# Methodology for Neural Network-Based Material Card Calibration Using LS-DYNA *MAT_187_SAMP-1* Considering Failure with GISSMO

**DOI:** 10.3390/ma15020643

**Published:** 2022-01-15

**Authors:** Paul Meißner, Jens Winter, Thomas Vietor

**Affiliations:** Institute for Engineering Design, Technische Universität Braunschweig, Hermann-Blenk-Strasse 42, 38108 Brunswick, Germany; jens.winter@tu-braunschweig.com (J.W.); t.vietor@tu-braunschweig.de (T.V.)

**Keywords:** parameter identification, machine learning, hyperparameter optimization, LS-DYNA, *MAT_187_SAMP-1*, GISSMO failure model

## Abstract

A neural network (NN)-based method is presented in this paper which allows the identification of parameters for material cards used in Finite Element simulations. Contrary to the conventionally used computationally intensive material parameter identification (MPI) by numerical optimization with internal or commercial software, a machine learning (ML)-based method is time saving when used repeatedly. Within this article, a self-developed ML-based Python framework is presented, which offers advantages, especially in the development of structural components in early development phases. In this procedure, different machine learning methods are used and adapted to the specific MPI problem considered herein. Using the developed NN-based and the common optimization-based method with LS-OPT, the material parameters of the LS-DYNA material card *MAT_187_SAMP-1* and the failure model *GISSMO* were exemplarily calibrated for a virtually generated test dataset. Parameters for the description of elasticity, plasticity, tension–compression asymmetry, variable plastic Poisson’s ratio (VPPR), strain rate dependency and failure were taken into account. The focus of this paper is on performing a comparative study of the two different MPI methods with varying settings (algorithms, hyperparameters, etc.). Furthermore, the applicability of the NN-based procedure for the specific usage of both material cards was investigated. The studies reveal the general applicability for the calibration of a complex material card by the example of the used *MAT_187_SAMP-1*.

## 1. Introduction

In recent years, the application of FE analyses has become an indispensable tool in the product development process, especially when it comes to the design and dimensioning of structural components with complex materials such as polymers or fiber-reinforced plastics [1,2,3]. Due to the increasing computational capacity and the progress in the software development of FE programs, the level of detail in calculations has increased in recent years and the generated data have become more accurate [4]. As a result, increasing efforts are being made to use this growth in data quantity and quality to apply machine learning (ML) methods for component development to further reduce computation times and improve the quality of results [4,5].

Nevertheless, the required computing resources, as well as application-dependent high computing time and necessary user expertise often represent a barrier to the use of CAE software in the development process. To perform high-quality FE analyses, material cards are required for FE solvers which are able to represent the respective material behavior of the evaluated model with sufficient quality. Currently, state-of-the-art FE solvers use a large number of various different and mostly phenomenological material models [6,7,8,9].

In addition to the selection of an appropriate material card to take into account the respective material behavior, it is necessary to calibrate the material parameters (MPs) of the chosen material card. Since the phenomenological material models only describe the empirical relationships of the material behavior and thus the MPs do not always have a direct physical background, it is usually not possible to directly determine them from experimental material tests. Furthermore, even physical MPs determined from experimental tests do not usually lead to sufficiently good reproduction quality in the simulation, since FE model discretization, settings and numerical effects can lead to deviations. In order to achieve the best possible agreement between simulation and reality, the identification of the MPs in a calibration process is necessary. The material card calibration process is usually accomplished either by a trial-and-error approach or by a semi-automatic, iterative optimization procedure. This requires comprehensive expertise in continuum mechanics, materials science, FE software and numerical optimization [10,11,12]. Additionally, the conventional iterative method, in which the optimization is usually performed on a metamodel, is computationally and time-intensive and hence expensive. In the early stages of product development, different materials for the design are often compared with each other and sometimes the material selection is adjusted in the further design process. For the purpose of design and safety verification using numerical simulations, this requires the repeated execution of the MPI process. Within the conventional parameter identification (PI) method, the entire process has to be repeated, even if only minor changes are applied to the material being calibrated—e.g., by changing the amount of a material additive. This restricts the possibility of shortening the product development time.

In contrast to this common method, a rather novel method for PI based on machine learning can alternatively be used in a direct inverse process to determine the MPs that can best reproduce the experimentally determined material behavior [13,14,15]. An essential advantage of this approach comprises the storage of information in the form of the weights and biases in the neural network structure during the training process, allowing the reusability of trained NNs to determine MPs for modified materials with similar material characteristics. Thereby, an efficient MPI for specific material groups as well as for different process settings and machines is possible. Since the prediction of MPs requires only a few seconds in contrast to the one-time training process, the development time could be decreased in this way [15]. Furthermore, no expert knowledge is required for the actual application of the NN to a specific experimental dataset, potentially enabling the application of CAE methods for product development to a wider range of users.

Different ML techniques have been presented in the literature [16,17]. In contrast to other approaches, exemplarily presented in [16], the aim of the present method is not to completely replace the FE simulations using the ML algorithms and to directly predict the material response to the external load. In the presented approach, an NN is trained based on simulation data and a prediction is performed for unknown datasets (usually experimental test data—in the present case, unknown virtual test data) in order to estimate MPs for numerical simulations.

In the often used optimization-based MPI procedures, a specific error metric between the experimental and the numerical dataset is established (e.g., stress–strain curves, eigenvalues or eigenfrequencies). The objective of the MPI process is then to minimize this error by varying the MPs [18] in an analytical or numerical calculation using various optimization techniques such as gradient-based methods (GBMs) [10,11] or genetic algorithms (GAs) [19]. However, the resulting accuracy for GBMs is significantly affected by the choice of the location of the starting points, which are typically not known [12]. For GAs, the large number of iterations required is often a disadvantage. Finding suitable parameters for sophisticated material models usually requires many iterations, which increases computational costs and often leads to receiving exclusively local optima. Additionally, the entire parameter identification process has to be restarted, if material cards have to be calibrated for marginally differing materials. For this commonly used MPI procedure, user-friendly software tools (e.g., *LS-OPT*, *Valimat*) or self-created optimization frameworks are usually applied. A global survey of various methods for PI is given in [20].

In comparison, Yagawa et al. [21] first described the direct procedure of training a feedforward artificial neural network (FFANN) using a squared loss function for the MP prediction of the material model. In this process, the neural network learns the inverse relationship between the simulation results and the corresponding MPs used. Because of this reverse relation, it is also called a direct inverse model [22]. Since the initial MPI of a viscoplastic material model by Yagawa et al., this approach has been applied to other materials, respectively, material models. Huber and Tsakmakis used this procedure for MPI for pure kinematic hardening in plasticity laws [23] and plasticity with nonlinear isotropic and kinematic hardening [24]. Furthermore, Nardin et al. identified the parameters of a constitutive law for soils by training the ANN with the results from micro- and macro-mechanical numerical models [25]. Aguir et al. [26] demonstrated the efficiency of the NN-based method in terms of low computation times by identifying parameters for the Hill’48 criterion under the associative and non-normality assumption and Voce law hardening parameters of AISI 304 stainless steel. To additionally examine the accuracy of the identified MP and their correlation, Unger and Könke [27] used a Bayesian neural network [28] for the direct inverse MPI. Furthermore, Adeli et al. [29] used a Bayesian approach to determine the parameters of a viscoplastic damage model. Meißner et al. [15] showed the applicability of the NN-based method for the prediction of a yield curve for the simulation of a thermoplastic polymer as well as examined influences on the prediction accuracy.

In this paper, the NN-based procedure for PI using a self-developed Python framework is presented and different network structures and network settings are compared regarding the prediction accuracy. Different established ML and PI methods from the scientific literature are combined in our framework. The method is applied to a virtually generated base dataset, which is derived from the experimental investigations of an additively processed acrylonitrile butadiene styrene (ABS) [15]. For the description of the material behavior in the FE-software LS-DYNA, the material card *MAT_187_SAMP-1* (Semi-Analytical Model for Polymers) was selected, with which the representation of the highly complex specific material behavior of thermoplastics is basically possible. The LS-DYNA card *MAT_ADD_DAMAGE_GISSMO* was also used for simulation, since additionally to the material characteristics—elasticity, plasticity, tension–compression asymmetry, variable transverse contraction and strain rate dependency—the failure should also be taken into account. For the calibration of the material parameters, different FE models of quasi-static and dynamic tests were created. The results of the structural simulations of the various tests provided the input for the FFANN to learn the relationship between the simulation results and the MPs during the training process. The NNs were built using the Python libraries *Tensorflow* and *Keras* and integrated into the custom framework. After optional hyperparameter optimization (HPO) with the library *Kerastuner*, the FFANN was finally applied to a validation (VAL) dataset as well as to the previously derived virtual base dataset and thus predicted corresponding MPs for the structural simulation. Using the predicted MPs, the different tests were simulated again and the results were compared with the initial simulations. Different settings of the FFANNs were investigated. Furthermore, a self-programmed custom loss function (CLF) was implemented in the network to increase the prediction accuracy.

For comparison, different optimization models for the MPI were built with the conventional iterative optimization-based method using the LS-OPT software and applied to the different datasets. Both PI approaches are explained and compared with each other in more detail in Section 2. In Section 3, the results of the different runs with varying settings (different meta-models, optimization algorithms, etc.) are compared with the results of the NN-based method. Finally, in Section 4, the results are summarized and the applicability of the NN-based method is evaluated. Furthermore, an outlook on additional research gaps and improvement capabilities of the introduced method is given.

## 2. Materials and Methods

In this section, the applied methods, models and procedures are briefly explained to improve the understanding of the used material parameter identification methods. Since the focus of this paper is on the NN-based PI method, it is discussed more precisely. Furthermore, the data generation process and the structure of the virtual investigations conducted are explained.

### 2.1. Artificial Neural Networks

The ANN used in this work is a so-called feed forward artificial neural network. It is composed of neurons arranged in layers, where the outputs of one layer serve as inputs for the following layer. The input layer (IL) obtains the information from the external environment. The hidden layer(s) (HLs) consist of the neurons responsible for the extraction of patterns related to the process or system to be analyzed. The output layer (OL) neurons are responsible for generating the final network outputs resulting from the processing of the previous layer data [30,31].

Hyperparameters (HPs) can be used to adapt or control the behavior of ML algorithms. These are not adjusted by the learning algorithm itself [32]. These HPs can occasionally have a significant impact on the predictive accuracy of an ML model [33]. The synaptic weights as well as the biases comprise the parameters of an FFANN, which have to be obtained within the training process [17,30,31,34]. However, the choice of ANN architecture is seldom transferable and significantly depends on the actual use case. Further in-depth information on the structure and architecture of ANNs can be found in [17,30,31,32,34].

The application-specific determination of the best interacting HP set is usually a difficult challenge, since usually, only the general influences on model performance are known. For overcoming this problem, approaches exist for objectively searching different values for model HP and selecting a single set, which leads to a model that performs best on a particular dataset. Those approaches are referred to as HP tuning or HP optimization and there exist a variety of different applicable libraries (e.g., *scikit-learn*, *Hyperas*) with different algorithms. More detailed information on these approaches can be found in [32].

Different HP search and optimization strategies were investigated and compared in Meißner et al. [15]—for a use case related to the one presented in this paper. In HPO, the retrieval of eligible HPs can be expressed as an optimization task. Here, the decision variables are the HPs and the objective function to be optimized is the validation set error resulting from training with these parameters. Since both random search and HP optimization using the Bayesian optimization algorithm (using *Keras Tuner*) provided good results in the study of Meißner et al., these strategies using the library *Keras Tuner* (V. 1.0.1) [35] were also used for some investigations within this paper. The Bayesian optimization provides a method based on the *Bayes theorem* to guide the search of a global optimization problem which can be effective and efficient. Thus, a so-called surrogate model is created, which represents a probabilistic model of the objective function. This in turn is used to calculate a so-called acquisition function, which can predict how promising an arbitrary point is. The maximization of this function thus provides new candidates for evaluation on the actual objective function [36]. For more detailed information on the optimization algorithms, we refer the reader to [32,35,36,37,38,39]. The HPs for a defined default NN and the investigated HP search range in this paper are listed in Appendix A, Table A4 and Table A5.

In the present case, the goal of the training process is to find a model $M^{INV}$ that matches the examples at best. The cost function quantifies the error between the predicted output and the labeled output and presents it in the form of a single real number, which should be minimized during the training process. Often, the mean square error (MSE) [32], also used in this paper, is used for this task. The backpropagation algorithm comprises the minimization of this cost function using the gradient descent (GD) optimization for the FFANN. In this process, the synaptic weights and biases are automatically updated in several iterations, successively decreasing the error generated by the FFANN. Some of the various specific GD optimization algorithms were tested within this work (see Section 2.5.2) [30,31].

During the training of NNs, the problem of overlearning often occurs. However, there are various methods of counteracting this, with one of them being *early stopping*. This method allows to specify an arbitrarily large number of training epochs and stops training once the model performance stops improving on a hold-out validation dataset. The investigations of Meißner et al. in the context of the paper [15] have already shown that this problem exists in the methodology used, which is why *early stopping* was used in the presented work on this paper. Nevertheless, preliminary work has also shown that the increase in error between the experiment (EXP) and final simulation with predicted parameters is not necessarily linked to an increase in validation error during NN training (see Section 3). Therefore, a variant of *early stopping* was chosen in this paper, in which the training process is stopped as soon as the validation error has not decreased for a defined number of epochs [30,32].

Another method of preventing overlearning and improving the performance of the NN for unknown datasets (generalization)—which are not used for training—is dropout. At each training stage, individual nodes are either dropped out of the net with a probability of 1−μ or kept with probability μ, so that a reduced network is left. Dropout has the effect of making the training process noisy and forcing nodes within a layer to probabilistically take on more or less responsibility for the inputs, which is why this was also used for some of the network architectures examined in this work [32,40].

### 2.2. Description of the Material Behavior with LS-DYNA MAT_187_SAMP-1 and GISSMO

The focus of this work is the evaluation and assessment of the NN-based MPI method described in Section 2.5.2 for subsequent structural simulations. The use of this method is not limited to any specific FE-solver, material model or material card. However, to extensively and profoundly compare the NN-based method with the conventional iterative optimization-based method and thus to reveal problems as well as to show its advantages, a material with complex characteristics in the form of a thermoplastic polymer was chosen as an application example based on the work in [15]. The following material characteristics or effects of this material class were selected to be identified by the two PI methods and should be considered in the subsequent structure simulation:

- Elasticity;
- Plasticity;
- Tension–compression asymmetry;
- Variable plastic Poisson’s ratio;
- Strain rate dependency;
- Failure.

At this point, it should be mentioned that thermoplastic polymers possess further characteristic properties, which, however, are not considered in this work and are also often neglected in a structural simulation at a macroscopic level. In the FE software LS-DYNA, various material models are implemented in a multitude of material cards, whereby the description of thermoplastic polymers is basically possible with diverging accuracy with some of them. Principally *MAT_187_SAMP-1* and *MAT_187_SAMP_Light* are applicable material cards for the consideration of the various characteristics of thermoplastics in numerical simulation, with the latter being a simplification of the first variant [6,41,42]. Specifically, thermoplastic polymers do not possess a constant modulus of elasticity and the different flow criteria under tension and compression preclude the use of a von Mises-type flow surface. Furthermore, the strain hardening of thermoplastics is anisotropic and plastic deformation does not occur at constant volume. This lack of plastic incompressibility requires a flow rule that allows permanent volumetric deformation. To account for these various characteristics, it requires a complex material model, which is why *MAT_187_SAMP-1* was used in this work. However, it should be mentioned that *MAT_187_SAMP-1* can describe other characteristics as well, whereas only the ones considered for this work will be presented in more detail in the following [6,42].

In *MAT_187_SAMP-1*, it is possible to directly import experimental data in the form of a defined curve or a table into the material card. However, these provided data do not usually lead to the desired agreement between experimental and simulative results, which is why optimization or a trial-and-error process is usually necessary. In these cases, it is therefore required to reproduce the input curve by the value pairs of a mathematical formulation from the literature or by a spline. The MPs are then the parameters of these mathematical formulations. In contrast to conventional material parameters, such as Young’s modulus (*emod*) and bulk modulus (*bulk*), these are only indirectly included in the material card.

Three different yield curve formulations are obtainable with *MAT_187_SAMP-1*, which are defined depending on the input provided for the material card. The von Mises yield surface formulation is achieved by only utilizing tension test data as input in *MAT_187_SAMP-1*. The implementation of only either compression and tension test data or shear and tension test data results in the Drucker–Prager yield surface definition. Whereas the provision tension, compression, and shear test data to *MAT_187_SAMP-1* generates the yield surface definition of *MAT_187_SAMP-1* known as the SAMP-1 yield surface. In the following, some theoretical formulations are presented, on which the material model is based. Please refer to [6,42] for complete explanations.

The SAMP-1 yield surface can be expressed by the following formula:

(1) $$f=\sigma_{vm}^{2}-A_{0}-A_{1p}-A_{2p^{2}}\leq 0,$$

with $\sigma_{vm}$ being the von Mises stress which can be formulated as follows:

(2) $$\sigma_{vm}=\sqrt{\frac{3}{2}\left[{\left(\sigma_{xx}+p\right)}^{2}+{\left(\sigma_{yy}+p\right)}^{2}+{\left(\sigma_{zz}+p\right)}^{2}+2\sigma_{xy}^{2}+2\sigma_{yz}^{2}+2\sigma_{xz}^{2}\right]},$$

and *p* being the first stress invariant which can be expressed as

(3) $$p=-\frac{\sigma_{zz}+\sigma_{yy}+\sigma_{zz}}{3}.$$

The unknown constants of $A_{0}$, $A_{1}$, and $A_{2}$ can be calculated through using tension, compression, and shear test data as functions of the test results:

(4) $$A_{0}=3\sigma_{s}^{2},$$

(5) $$A_{1}=9\sigma_{s}^{2}\left(\frac{\sigma_{c}-\sigma_{t}}{\sigma_{c}\sigma_{t}}\right),$$

(6) $$A_{2}=9\left(\frac{\sigma_{c}\sigma_{t}-3\sigma_{s}^{2}}{\sigma_{c}\sigma_{t}}\right).$$

Since the results of the two different shear tests performed were not used as input for the material card, but only as a target curve during calibration, only tensile and compression test data were provided to generate the yield surface, and thus a linear yield surface was generated. Hence, the Drucker–Prager yield surface was used and the remaining curve was calculated internally from the two available curves:

(7) $$\sigma_{s}=\frac{2\sigma_{c}\sigma_{t}}{\sqrt{3}\left(\sigma_{t}+\sigma_{c}\right)}.$$

Furthermore, *MAT_187_SAMP-1* provides the capability to define two different flow rules, named associated and non-associated, depending on the definition of the plastic Poisson’s ratio (PPR) used. If the constant PPR is used in the material card, the yield rule is considered as associated yield. Otherwise, if the change of the PPR is defined with the plastic strain, the non-associated yield rule is generated, which is used in the simulations. This is the case in the present work since the plastic Poisson’s ratio changing under loading should be considered in the simulation. The expression of the yield rule can be given as follows:

(8) $$g=\sqrt{q^{2}+\alpha p^{2}},$$

where α is the angle between the hydrostatic axis and plastic potential and thus a function of plastic strain. α can be specified by the following expression:

(9) $$\alpha =\frac{9}{2}\cdot \frac{1-2\nu_{p}}{1+\nu_{p}}.$$

If curves from dynamic tests are available, then the load curve defining the yield stress in uniaxial tension is replaced by a table definition. This table then contains multiple load curves corresponding to different values of the plastic strain rate. Additionally, in *MAT_187_SAMP-1*, the rate effect in compression and shear are assumed to be similar to the rate effect under tensile load [6].

Although *MAT_187_SAMP-1* has an integrated failure model, the stand-alone failure model *Generalized Incremental Stress-State Dependent Damage Model* (*GISSMO*) is very flexible and widely applied in combination with many different material cards; hence, this has been used to consider failure within this work. Using *GISSMO*, damage prediction can be represented by taking into account material instability as well as localization and failure. The failure model formulation of *GISSMO* allows an incremental description of damage accumulation, including softening and failure. It offers the advantage of defining an arbitrary triaxiality-dependent failure strain. The damage accumulation is based on an incremental formulation, which consists of [42,43,44,45,46]:

(10) $$\dot{D}=\frac{n}{\epsilon_{f}}D^{\left(1-\frac{1}{n}\right)}\dot{\epsilon_{p}},$$

with *D* being the current value of damage, $\dot{\epsilon_{p}}$ the plastic strain rate, *n* the damage exponent and $\epsilon_{f}$ the equivalent plastic strain at failure (EPSF). The onset of necking is considered by the forming intensity parameter *F* (instability measure):

(11) $$\dot{F}=\frac{n}{\epsilon_{crit}\left(\eta \right)}F^{\left(1-\frac{1}{n}\right)}\dot{\epsilon^{p}},$$

where the critical strain curve, $\epsilon_{crit}\left(\eta \right)$, is also a function of the triaxiality (TRI) and is intended to act as a trigger for coupling damage and stress under proportional loading. Hence, this curve can be interpreted as an instability criterion, i.e., from this point on, material degradation becomes critical and the material point experiences an accelerated, localized strain behavior up to fracture. The main difference between functions *D* and *F*, is the type of limiting strain, which depends on the triaxiality forms used, $\epsilon_{f}$ or $\epsilon_{crit}\left(\eta \right)$. The damage in this formulation is coupled to the stress tensor, using the concept of effective stress of Lemaitre [47,48], when the instability is reached F=1:

(12) $$\sigma_{eff}=\sigma \left(1-{\left(\frac{D-D_{crit}}{1-D_{crit}}\right)}^{m}\right).$$

where $D_{crit}$ is used as an indicator for reaching the onset of necking. The exponent *m* is called the fading exponent, which is used for the regularization of fracture strain and the energy consumed during post-instability deformation. Further detailed information on *GISSMO* is provided in [42,43,44,45,46].

**Figure 1:**
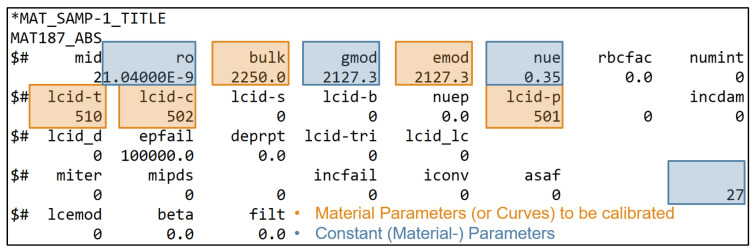
Exemplary illustration of the material card *MAT_187_SAMP-1* used in this work.

**Figure 2:**
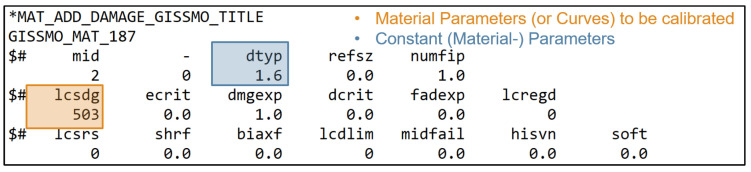
Exemplary illustration of the material card *MAT_ADD_DAMAGE_GISSMO* used in this work.

Figure 1 and Figure 2 show the *MAT_187_SAMP-1* and *MAT_ADD_DAMAGE_GISSMO* material cards used in this work, with the corresponding input parameters and input curves highlighted. The complete material parameter setup (start values, minimum and maximum values, target values, etc.) are provided in Section 2.4, Table 1.

The Young’s modulus *emod* and the bulk modulus *bulk* are variables in the present study which are to be identified by the two MPI methods. In contrast, the shear modulus *gmod*, the density *ro* and the elastic Poisson ratio *nue* are assumed to be constant for all investigated datasets, as can be seen in Section 2.3. The constant value *27* in the last column of the material card *MAT_187_SAMP-1* (Figure 1) allows the output of the equivalent plastic strain (EPS) on the *history variable 27*, which is not provided by default.

As mentioned before, in *MAT_187_SAMP-1*, it is possible to import curves for the definition of material characteristics. To achieve the best possible match between simulation and reality by an optimization process or by an NN-based prediction, it is necessary to parameterize these curves by analytical functions or by splines. Thus, the MPs are the parameters of the analytical curve. In the present case, *lcid-t* represents the yield curve (more precisely, the strain rate-dependent yield curves at different strain rates, presented in a table). In order to reproduce the significant softening of the thermoplastic polymer while keeping the number of MPs small (without considering damage), the approach of Meißner et al. with a combination of second-degree polynomial and root function [15] was used in this work:

(13) $$\sigma =a\cdot \epsilon_{pl}^{2}+b\cdot \epsilon_{pl}+c+d\cdot \epsilon_{pl},$$

with the material parameters *a*, *b*, *c*, *d* and the plastic strain $\epsilon_{pl}$. This formulation is similarly used for the description of plasticity under compressive loading (*lcid-c*). To take into account the tension–compression asymmetry, this results in four MPs for the description of plasticity under tensile load and four MPs for the description under compressive load.

The curve *lcid-p* describes the PPR as a function of plastic strain in uniaxial tension and uniaxial compression, where the plastic strain on the abscissa is negative for compression and positive for tension. This curve is usually determined from Digital Image Correlation (DIC) measurements during experimental tests [49]. The typical behavior of unreinforced thermoplastic polymers [50] is described in this paper with the formula:

(14) $$\nu_{p}=\nu_{p,plat}-\left(\nu_{p,plat}-\nu_{p,press}\right)\cdot e^{min\left(\frac{-5\cdot \epsilon_{pl}}{\epsilon_{p,plat}};0\right)},$$

where $\nu_{p}$ is the plastic Poisson’s ratio. The model is used to describe an exponential decay from the pressure side to a plateau on the tension side, where $\nu_{p,plat}$ is the PPR value of the tension side, $\epsilon_{p,plat}$ defines the value where 99% of the difference between compression and tension is subtracted and $\nu_{p,press}$ is the constant PPR of the compression side.

To take strain rate dependency into account in *MAT_187_SAMP-1*, instead of a single yield curve *lcid-t*, a table with multiple yield curves at different strain rates must be provided [42], as can be seen in Figure A2 in Appendix A. In this work, the Cowper–Symonds analytical approach [51] was used to scale the quasi-static yield curve:

(15) $$\sigma =\sigma_{0}\left(\epsilon \right)\left[1+{\left(\frac{\dot{\epsilon }}{C}\right)}^{\frac{1}{P}}\right].$$

This has to be included in the expression of the yield curve (Equation (13)), resulting in the equation:

(16) $$\sigma =\left(a\cdot \epsilon_{pl}^{2}+b\cdot \epsilon_{pl}+c+d\cdot \epsilon_{pl}\right)\left[1+{\left(\frac{\dot{\epsilon }}{C}\right)}^{\frac{1}{P}}\right],$$

where $\dot{\epsilon }$ is the strain rate resulting from the experimental test and the two additional material parameters *C* and *P*, which have to be estimated in the PI process.

The failure is implemented using the curve *lcsdg* in *MAT_ADD_DAMAGE_GISSMO*, where the curve defines EPSF vs. TRI [42]. The (stress) triaxiality is defined as the ratio of hydrostatic stress $\sigma_{m}$ divided by von Mises equivalent stress $\sigma_{eq}$ (Equation (18)), which is equal to the ratio of negative hydrostatic pressure (Equation (3)) divided by the von Mises equivalent stress [43,52,53]:

(17) $$\eta =\frac{\sigma_{m}}{\sigma_{eq}}=-\frac{p}{\sigma_{eq}},$$

with:

(18) $$\sigma_{eq}=\sqrt{\sigma_{x}^{2}+\sigma_{y}^{2}+\sigma_{z}^{2}-\sigma_{x}\sigma_{y}-\sigma_{y}\sigma_{z}-\sigma_{z}\sigma_{x}+3\left(\tau_{xy}^{2}+\tau_{yz}^{2}+\tau_{zx}^{2}\right)}.$$

**Figure 3:**
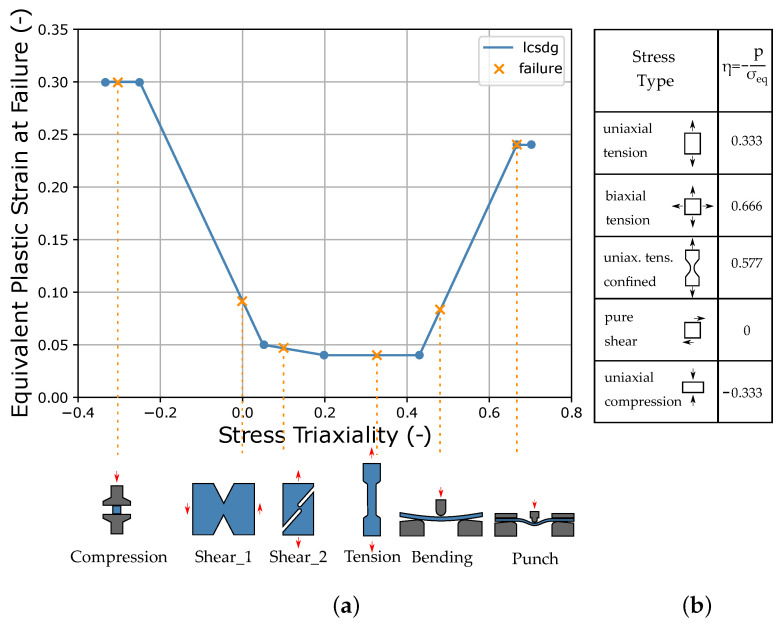
Equivalent plastic strain at failure vs. triaxiality curve with corresponding tests used in this paper (**a**) and stress state-dependent triaxialities (**b**).

The triaxiality is a scalar quantity, which allows an exact characterization of the element stress state in the case of the plane stress state. Different loading conditions lead to different stress triaxialities (see Figure 3b).

In this paper, the curve *lcsdg* is defined by a discontinuous linear function consisting of six straight lines (see Figure 3a), which is derived from the failure behavior of unreinforced thermoplastics known from the literature [54]. The exemplary LS-DYNA input curve is shown in Figure A3, Appendix A, where it is defined by seven points at different triaxialities in the range of −0.333–0.7. Here, four values for the equivalent plastic strain at failure ((r)epsf_0 to (r)epsf_3) were defined, which in this work represent the four MPs for the description of the failure. However, the definition of this curve is flexible and could also be done by a different or also finer resolution of the points of the curve. A continuous function could also be advantageous. The EPS was chosen as the driving quantity for the failure (dtyp=1.6). During the simulation of the test, once an element reaches an EPS greater than or equal to the threshold defined by the curve *lcsdg*, failure occurs and simultaneously the element is deleted.

In order to calibrate the points on the ESRP vs. TRI curve of the failure model sufficiently for the real application under complex loading conditions, different tests for the determination of varying sections are necessary. However, these do not always provide the same point on the curve, since the triaxiality changes during the test due to the different stress components. Depending on the material properties and corresponding deformation, they may also fail under a different triaxiality. In Figure 3a, the tests used in this work (see Section 2.3) are assigned to the respective segment to be calibrated.

Furthermore, there are additional input options for *MAT_187_SAMP-1* and *MAT_ADD_DAMAGE_GISSMO*, which can be used to implement and consider more complex properties. However, these are not considered here and therefore we refer to [42] for further information.

**Figure 4:**
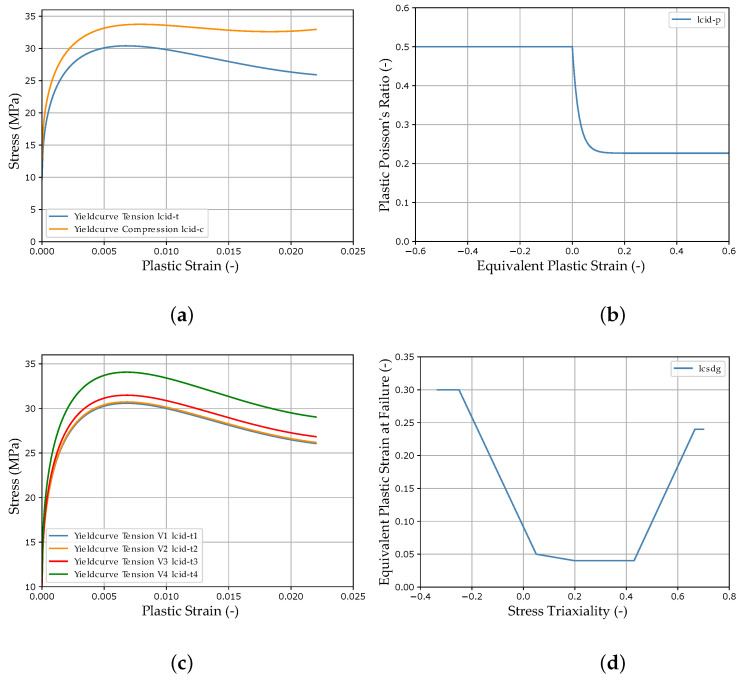
Material card analytical input curves, calculated with the target parameters of the virtual experimental dataset: (**a**) lcid-t and lcid-p for plasticity and tension–compression asymmetry; (**b**) lcid-p for variable plastic Poisson’s ratio; (**c**) lcid-t1 to lcid-t4 for strain rate dependency and (**d**) lcsdg for failure.

The imported analytical curves, calculated with the target parameters of the virtual experimental dataset (see Section 2.4), for the *MAT_187_SAMP-1* and *MAT_ADD_DAMAGE_GISSMO* material cards are shown in Figure 4. The material parameters to be identified, the respective material card input curves (MCIC) and parameter ranges can be found in Table 1 above.

### 2.3. Finite Element Models

As explained in Section 2.2, the calibration of an extensive material card such as *MAT_187_SAMP-1* (and *MAT_ADD_DAMAGE_GISSMO*) requires different experiments. In this work, the investigations are performed using virtual test data resulting from FE simulations. The FFANN is also trained using virtually generated data, which has a significant advantage over other methods based on experimental data concerning the necessary exp. tests. Hence, the creation of the FE models used is briefly discussed below.

**Figure 5:**
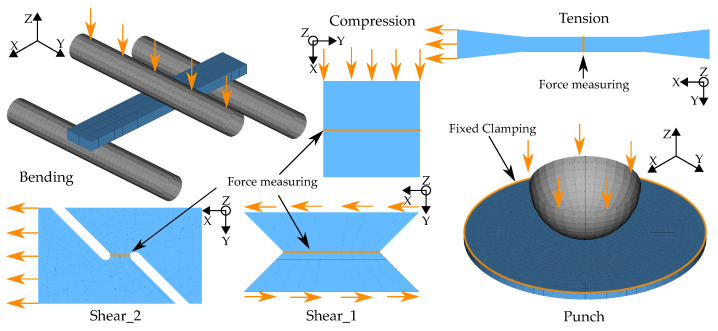
Created FE-models for the PI methods used.

In the course of calibration with the two MPI methods used in this work, a total of ten different FE models were created: a quasi-static tensile test, four strain rate-dependent tensile tests (velocities 1–4), a compression test, a three-point bending test, a punch test, and two different shear tests (see Figure 5).

For all models, the discretization with the shell formulation ELFORM 16—fully integrated shell element—and five integration points were chosen. The relatively coarse discretization of the tensile test model results from the requirement to drastically reduce the simulation time of the strain rate dependent tensile tests, which exhibit very high computation times at low loading rates. In order to reduce the computation time of the explicit simulation, mass-scaling was applied for all models using the parameter *DT2MS*. In addition, time-scaling was used, which can be applied to the reduction in simulation times. However, since this cannot be employed for strain rate-dependent tests, only the four dynamic tensile tests tension V1–V4 were calculated with the strain rate-dependent material card *MAT_187_SAMP-1*. Thus, the strain rate-dependent behavior of the virtual material used is exclusively reflected in the simulation results of these four dynamic tests and time scaling could thus be used for all other tests. The respective values for time and mass scaling were determined for each test in a preliminary study. The criteria chosen for the selection of the respective values were the ratio of kinetic to internal energy and the ratio of maximum force from unscaled to scaled model. These should be below 1% to be able to exclude a significant influence of the scaling factors. The strain rate-dependent tensile test velocities V1–V4 are: 0.0166 mm·s${}^{-1}$, 0.1666 mm·s${}^{-1}$, 16.666 mm·s${}^{-1}$ and 1666.6 mm·s${}^{-1}$. With the specimen geometry, the resulting strain rates are: $1\times 10^{-4}$ s${}^{-1}$, $1\times 10^{-3}$ s${}^{-1}$, $1\times 10^{-1}$ s${}^{-1}$ and $1\times 10^{1}$ s${}^{-1}$.

**Figure A1:**
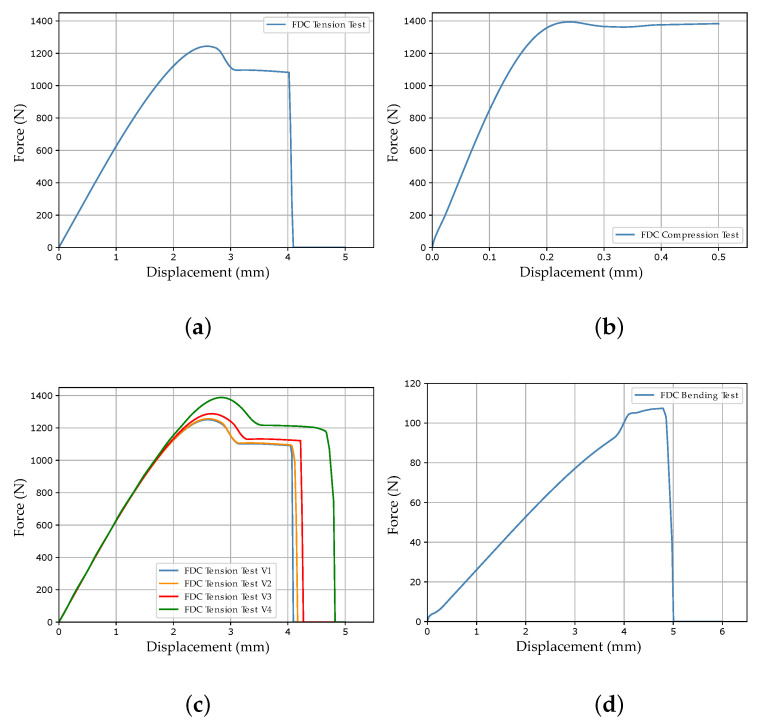

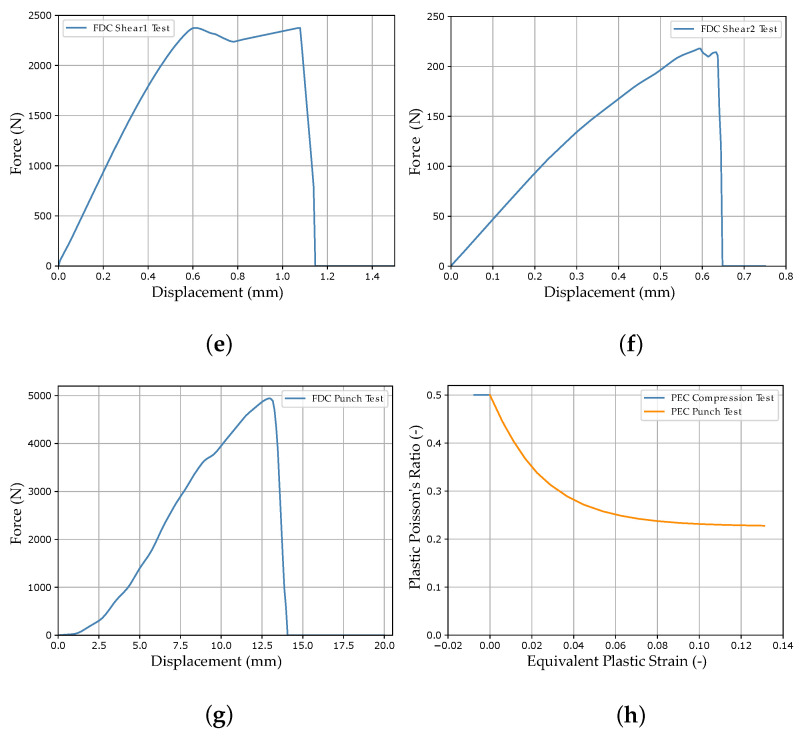
Simulation output curves of the virtual experimental test dataset: (**a**) FDC tension test; (**b**) FDC compression test; (**c**) FDC tension tests V1–V4; (**d**) FDC bending test; (**e**) FDC shear1 test; (**f**) FDC shear2 test; (**g**) FDC punch test; and (**h**) PEC compression and punch test.

**Figure A2:**
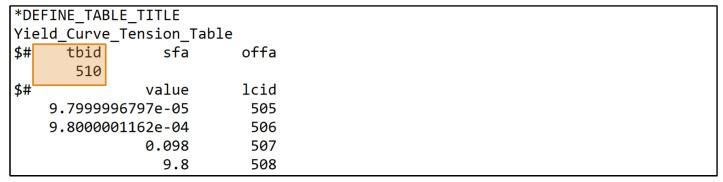
Table definition of strain rate-dependent yield curves (yield curve at specific strain rate) used for *MAT_187_SAMP-1*.

**Figure A3:**
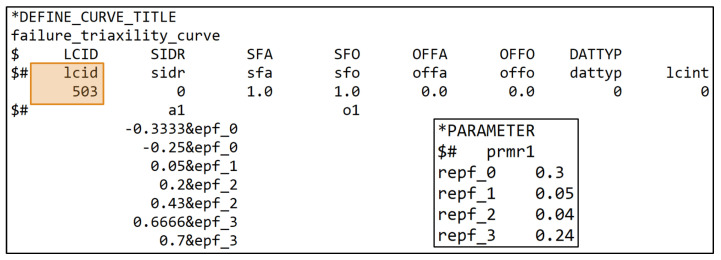
Equivalent plastic strain at failure vs. triaxiality curve for *MAT_ADD_DAMAGE_GISSMO*, with (r)epsf_0 to (r)epsf_3 (equivalent plastic strain at failure) being parameters which need to be calibrated.

For the application of the load, a velocity-controlled load curve *DEFINE_CURVE_ SMOOTH was used for all tests in order to reduce influences such as vibrations caused by abrupt load application. For additionally required objects, such as supports in the bending test, the material card *MAT_RIGID was used. For all models, the force and displacement were evaluated over the history of the tests (see Figure 5). Additionally, for the compression and punch test, the equivalent plastic strain and plastic Poisson’s ratio were evaluated using *History Variables* 2 and 27, respectively, always at the same integration point of the identical element. This results in a total of twelve simulation output curves (SOCs), which are shown in Appendix A, Figure A1, as an example for the virtually generated experimental dataset. In the NN-based method, the ordinate values of these curves represent the input of the NN (see Section 2.5.2). In addition to the variable MP, which should be determined by the applied approach, the following MPs were used: shear-modulus gmod=2127.3 MPa; elastic Poisson’s ratio nue=0.035; density ro=1.04 cm${}^{3}$/g; equivalent plastic strain at failure $epfail=1\times 10^{5}$ (default value for latest possible failure).

### 2.4. Material Parameter Setup and Configuration of the Virtual Investigations

As mentioned previously, the necessary data for training the FFANN in the NN-based MPI method are generated by numerical simulations. Nevertheless, the final target is to predict the MPs for an experimental dataset in order to subsequently perform structural simulations. Within this work, a virtual experimental dataset was generated from numerical simulations for the validation of the two investigated MPI methods. The material properties of the virtual material were adapted to the data described in Meißner et al. [15], which was based on the additively processed thermoplastic ABS. Using the MPs defined in Table 1 and the analytical formulas presented in Section 2.2, the corresponding MCIC (see Figure 4) were calculated, producing the corresponding *MAT_187_SAMP-1* and *MAT_ADD_DAMAGE_GISSMO* material cards. These material cards were then imported into the FE models presented in Section 2.3 and the corresponding (true) SOCs were evaluated. Figure A1 in Appendix A shows the twelve SOCs of the virtual experimental dataset and in Figure A6, the noise-affected SOCs of training dataset two are presented (see Supplementary Materials for range plots of all datasets).

The two MPI methods are subsequently used for searching the MPs to obtain the minimum error between the true SOC and the SOC calculated from the optimized/predicted MP. This error is evaluated with both methods using the dynamic time warping distance (see Section 2.5.1), whereby in general, other distance measures would also be possible. In the iterative optimization-based method, the MPs are changed in order to directly minimize this error. In contrast, the NN-based method is applied to minimize the error of the true/predicted MPs or the material card input curves (see Figure 4) during NN training (see Section 2.5.2). For both MPI methods, an MP range has to be defined in which the search is performed. To ensure comparability, the same MP range was used for both MPI methods (see Table 1). This range was also used for the generation of the training and validation datasets of the NN-based method. For example, the noise-affected SOCs of training dataset two are shown in Appendix A, Figure A6. The design of the studies conducted using the two MPI methods is explained in Section 2.5.1 and Section 2.5.2, respectively.

### 2.5. Material Parameter Identification Process

Using a PI procedure, the required material parameters for the material models are obtained, resulting in calibrated material cards that are subsequently used to perform structural simulations. The aim of model calibration is to obtain an unknown parameter, knowing the experimentally measured response of a system to the applied loads. Nevertheless, this presupposes the material model employed to be fundamentally suitable for representing the material response with appropriate accuracy due to its mathematical expression. Combined with the FE model, which must be capable of correctly representing the EXP, the efficient and reliable PI method is crucial for structural modeling and safety evaluation. Two main methods exist to solve this identification problem. The most widely used PI approach commonly involves an error minimization approach in which the distance between the parameterized model predictions and the EXP test results is minimized [20]. Unfortunately, this error minimization technique often results in challenging optimization tasks that are heavily nonlinear and multimodal. In contrast, in the second direct inverse NN-based method, the presence of an inverse relationship between the output and input is assumed. The final determination of the required inputs takes only a few seconds and can be easily repeated once such a relationship is established. Both approaches are described more detailed as follows.

#### 2.5.1. Iterative Optimization-Based Procedure Using LS-OPT

For PI, an iterative optimization procedure is described as the minimization of an error function F(x) stated as the difference between the model outputs $y^{M}$ and the output of the experiment $y^{E}$ (measurements in form of stress–strain curves, etc.), meaning that:

(19) $$minF\left(x\right)=min||y^{E}-M\left(x\right)\left|\right|;$$

(20) $$y^{M}=M\left(x^{M}\right),$$

where *M* represents the material model with its constitutive relations which characterizes the stress–strain relations and *x* the unknown MP for the material model. A solution $x^{M}$ results from the minimum of this function and if $F(x^{M})>0$, the residual error is caused by the inaccuracy of a model or by some noise in the measured data [18].

As mentioned above, Equation (19) is commonly solved by gradient-based optimization methods or genetic algorithms, although some drawbacks arise in the context of this method. As already presented in Meißner et al. [15], these disadvantages include:

- The results of the optimization process are highly dependent on the choice of starting point, while the ideal location is unknown;
- Finding suitable parameters for sophisticated material models requires many iterations, which lead to high computational costs;
- Even with minor modifications in the experimental setup or in the investigated material, the computationally and time-consuming process has to be restarted [12,18].

**Figure 6:**
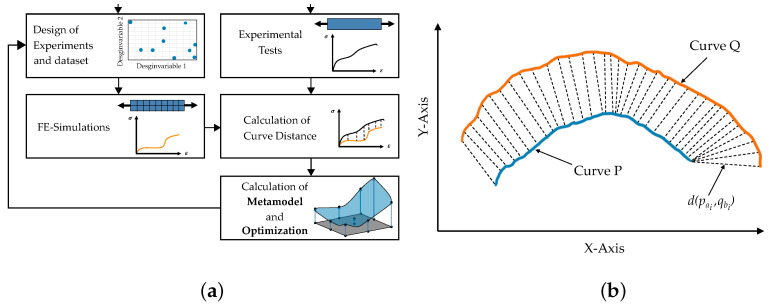
Metamodel-based iterative optimization methodology for PI identification (**a**) and curve distance measurement algorithm dynamic time warping (**b**).

Different solutions exist to apply the iterative optimization-based method, which includes commercial software as well as often used in-house developed software. As mentioned before, the commercial software LS-OPT (Version 6.0) [55] was used in this work, which is commonly applied for PI tasks [56,57] but can also be used in other fields such as structural design [58]. A brief overview of the used optimization-based methodology is shown in Figure 6a. For a more detailed description of this inverse PI, we refer to [59,60,61].

For the so-called distance measurement of experimental and simulated curves, different algorithms exist [62] depending on the application. In the field of MPI, mean squared error, partial curve mapping [63], discrete Frechét distance [64] and dynamic time warping (DTW) [65,66] should especially be mentioned.

Especially steep noisy curves with different lengths complicate the determination of a reasonable distance measure as well as a suitable objective function for optimization. As failure was included as a material characteristic in the present work, steep curves with different lengths had to be handled. Therefore, MSE could not be used as in Meißner et al. [15]. Instead, the dynamic time warping algorithm was used in this work for both the iterative optimization-based method and the final comparison with the NN-based method (see Figure 6b. To calculate the DTW distance, a matrix is set up in which the distances of each point of a curve to the points of another curve are listed. From this matrix, a corresponding warping path $W=(w_{1},\ldots ,w_{l})$ can be determined that maps the curves to each other. Mathematically, the measurement procedure can be expressed as follows:

(21) $$DTW\left(P,Q\right)=\frac{1}{l}\min_{w}\left\{\sum_{i=1}^{l}\delta \left(w_{i}\right)\right\},$$

with $\delta \left(w_{i}\right)=d\left(p_{h},q_{k}\right)$, if $w_{i}=\left(h,k\right)$, h∈1,…,n and k∈1,…,m [55]. With this method, the Euclidean distance between all points is determined and combined in a matrix. The combinations with minimum Euclidean distance are then selected from this matrix. For the calculation of the DTW distance with the NN-based method, the Python library *similaritymeasures V 0.4.4* was used. In addition to the FE models, self-programmed Python scripts had to be integrated into the optimization process to automatically generate the corresponding input curves/tables (e.g., yield curve) from the MPs using the formulas from Section 2.2. The final target value of the optimization to be minimized results from the sum of the normalized distances of the respective curves. In the present case, the target value *F* to be minimized results from the sum of the normalized error of the respective FDC of the ten simulated tests and the normalized error of the plastic Poisson’s ratio-equivalent plastic strain-curves (PEC) of the compression and the punch test:

(22) $$\begin{array}{cc} F= & \frac{1}{12}(DTW_{FDC_{t}}+DTW_{FDC_{t1}}+DTW_{FDC_{t2}}+DTW_{FDC_{t3}}+DTW_{FDC_{t4}}\\ \; & +DTW_{FDC_{c}}+MSE_{PEC_{c}}+DTW_{FDC_{b}}+DTW_{FDC_{p}}+DTW_{PEC_{p}}\\ \; & +DTW_{FDC_{s1}}+DTW_{FDC_{s2}}) \end{array}$$

In this case, the DTW distance was always used to calculate the error, except when calculating the error of the PEC of the compression test. Since the evaluation of the PEC under compression load (see Figure 4b) results in a horizontal straight line and in the DTW algorithm, one curve is normalized to the other, which resulted in NAN values. Therefore, the MSE was used for this specific case. To prevent oscillations and related optimization problems, the curves calculated by the simulations were truncated after failure at a force of ∼0 using a *lookup* function integrated into LS-OPT.

In the present work, the *sequential strategy with domain reduction* was used to apply automated metamodel-based optimization. Similarly to the *sequential strategy*, sampling is done sequentially, whereas for each iteration, a small number of points is chosen and multiple iterations are requested. In contrast, in each iteration, the domain reduction strategy is used to reduce the size of the subregion. During a particular iteration, the subregion is used to fix the positions of the new points [55].

**Figure 7:**
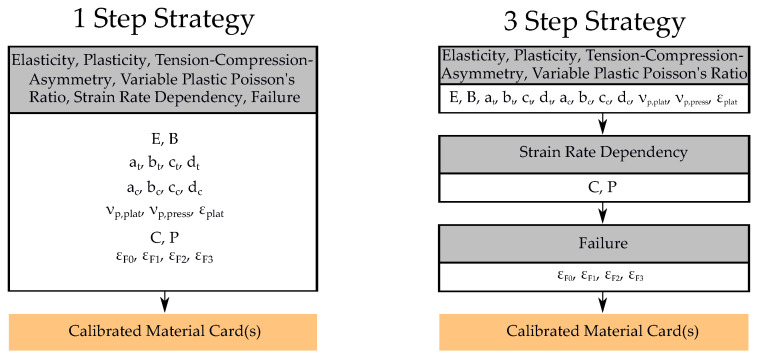
Used 1 Step Strategy and 3 Step Strategy for iterative optimization-based calibration.

In this work, two basic strategies were implemented using this method to calibrate the MPs for the virtual experimental dataset (see Figure 7). The sum of the number of simulations per iteration was oriented to the dataset size of the training set used for the NN, in order to improve the comparability of the two methods.

**Figure 8:**
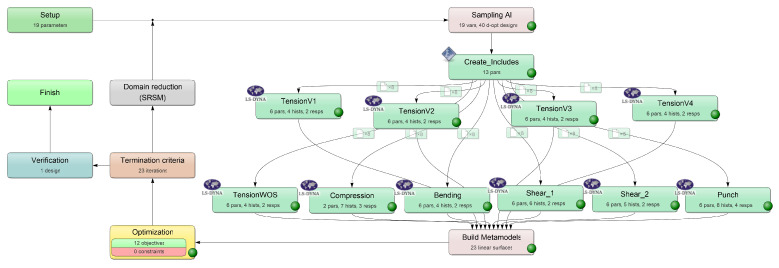
LS-OPT flowchart of material card calibration for the virtual experiment dataset (1 Step Strategy).

In a “1 Step Strategy”, all parameters were determined in a single optimization run (see Figure 8). In contrast, in a 3 Step Strategy, the parameters for elasticity, plasticity, tension–compression asymmetry and variable PPR were initially determined in a first optimization run. In this step, no strain rate dependency was integrated, and thus, the FE models of the tension tests V1–V4 were not imported into the optimization process. Furthermore, failure was not considered in this step and therefore the *MAT_ADD_DAMAGE_GISSMO* card was not used. Subsequently to the second step, the parameters for the strain rate dependency were exclusively calibrated in an optimization with the four strain rate-dependent tension tests. The previously calibrated parameters were assumed to be constant. In the final step, only the four MPs for failure were estimated and all other MPs were kept constant. Therefore, each FE model was used in this step, except for the compression model, since no failure occurs for this test. For each of the two strategies, different runs were performed with varying optimization algorithms and metamodel settings for the same dataset, in order to make a valid comparison to the NN-based method. The settings of the respective runs are provided in Table A1 and Table A2, Appendix A.

#### 2.5.2. Direct Neural Network-Based Procedure Using Self-Implemented Framework

**Figure 9:**
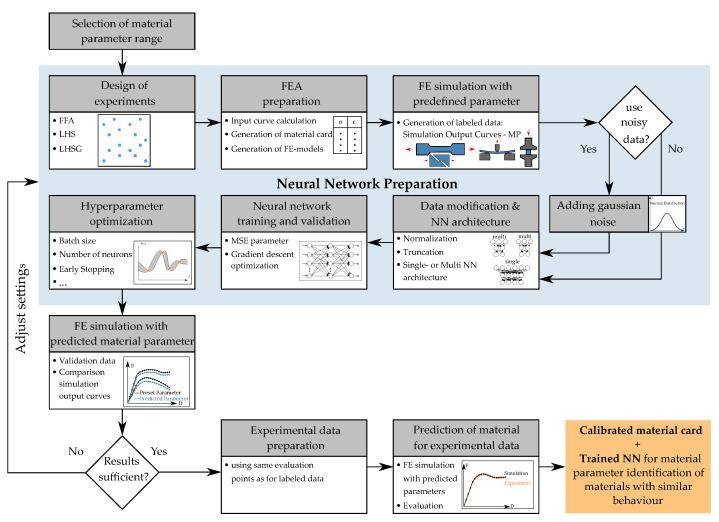
Workflow of the used direct NN-based parameter identification procedure applied to the problem of identifying the defined material parameters for *MAT_187_SAMP-1* and *MAT_ADD_DAMAGE_GISSMO*.

The direct inverse neural network-based procedure assumes there exists an inverse model $M^{INV}$ related to model *M*, which fulfills the following equation:

(23) $$x=M^{INV}\left(y\right)$$

for all possible *y* [18]. Aside from the large amount of data required, one of the disadvantages of this method is the time-consuming retrieval for the inverse relationship. In contrast, the major benefit lies in the storage of the generated knowledge and the associated possibility to retrieve the desired input within seconds. During the training process, all previously generated information (input–output relationship) is fed into the ANN. Unlike iterative optimization approaches with gradients or genetic algorithms, choices are consistently made based on actual designs. The core of this direct inverse method is to train an ANN to directly predict the MPs for a given input (e.g., force–displacement curves). In the context of the work in Meißner et al. [15], a corresponding method was established and further developed in the work for this paper. Figure 9 displays the workflow of this method, applied to the present dataset for the identification of the defined MPs (see Section 2.4, as can be seen in Table 1) of the material cards *MAT_187_SAMP-1* and *MAT_ADD_DAMAGE_GISSMO*. The workflow illustrated was implemented in a self-developed Python (V.3.7) environment. The libraries *Tensorflow* (V.2.1.0) and *Keras* (V.2.3.1) were used to create the NNs and implemented into the workflow. A GPU was used to execute the NN-based calculations.

In this method, the NN learns the relationship between simulation output curves (input: FDC or PEC) and the corresponding material parameters (output). After training the NN, the goal is to import unseen experimental data into the NN and then predict corresponding MPs for the subsequent structural simulation. In the work for this paper, a virtual experimental dataset was generated instead (see Section 2.4), which was used for performance evaluation and comparison with the iterative optimization-based method.

Similarly to the iterative optimization-based method, an MP range has to be defined initially, which ensures the sufficient coverage of the resulting material characteristics depending on the application scenario. However, with the NN-based method, this range solely needs to be defined by the creator of the NN and the actual end-user does not need to have this expert knowledge. In the following step, representative MP combinations are generated using sampling methods, which should cover the input space as sufficiently as possible. In Meißner et al. [15], the influence of some DOE methods on a dataset for the MPI of the parameters of a yield curve was investigated. The LH sampling revealed good results and was therefore also used for the investigations within this thesis using the library *PyDOE* (V.0.3.8).

Since most of the used MPs are only indirectly included in the material cards *MAT_187_SAMP-1* and *MAT_ADD_DAMAGE_GISSMO*, in our case, the automated calculation of the input curves/tables for the sampled MP sets using the formulas presented in Section 2.2 was required. The automatically evaluated simulation results (FDC with self-created code and PEC using the library lsreader V.0.1.37 from ls-prepost) with the corresponding MPs comprised the database for the training of the FFANN. The identical abscissa values (displacements or EPS) were analyzed for all the datasets of the respective SOC to ensure the comparability of the data. To ensure a sufficient database for the NN, 200 equidistantly distributed abscissa positions were defined for each SOC. With a total of twelve SOCs, this results in 2400 input values for the NN. The input layer of the NN thus always contains 2400 neurons. Each input dataset contains the corresponding 19 material parameters (Section 2.2, Table 1), which represent the output of the NN. Consequently, the output layer of the NN always possesses 19 neurons.

As the outcome of the FE simulations has no random errors, the resulting data (FDC and PEC) are clean and noise-free. For the robustness of the NN to measurement errors that always occur in physical experiments, and thus to increase its generalizability, Gaussian noise with zero mean and standard deviation σ is applied to the resulting normalized ordinate values (see Figure A6, Appendix A):

(24) $$Y_{noise}=Y\left(1+N\left(\mu =0,\sigma =0.001\right)\right)\;\mathrm{for}\;\mathrm{force},$$

and:

(25) $$Y_{noise}=Y\left(1+N\left(\mu =0,\sigma =0.0005\right)\right)\;\mathrm{for}\;\mathrm{plastic}\;\mathrm{poission}’s\;\mathrm{ratio}.$$

The standard deviation here represents an HP, which also influences the prediction accuracy. This value should be adjusted to the variation of experimental data or even be optimized.

During the simulation, oscillations often occur after the abrupt failure of the test specimens, which are reflected in heavy oscillations of the evaluated force–displacement curves (FDCs). The unsteady occurrence of these oscillations would significantly complicate the training process of the NN and decrease the prediction accuracy. Therefore, the force values of the FDCs are truncated after failure, using a specially created *failure-cut-function* and all subsequent force values are set to F=0. Additionally, filters for smoothing such as a moving-average filter are applied if necessary. For subsequent prediction based on unknown experimental data, the same modification must be applied to the input curves to ensure proper prediction quality.

Afterwards, the datasets are normalized to values between 0 and 1 (using scikit-learn V.0.22.1), since the algorithms employed for training the NN achieved improved results within this range. The following data types were normalized separately from each other:

- Material parameter (NN output);
- Ordinate values of material card input curves;
- Ordinate values of simulation output curves (NN input).

Since both the MPs and the different input and output curves have different ranges, these were also normalized separately from each other. To prevent the contamination of the validation dataset with actually unknown information, the normalization was always performed on the range of the training dataset. Additionally, the FDC or PEC can also lie outside the range used for training.

In preliminary studies for the work on this paper, different network architectures for the prediction of MPs of a tension yield curve and a compression yield curve with the formulation (Equation (13)) for LS-DYNA *MAT_024* were carried out. Simulations of a tension and a compression test were performed and NNs were trained identically to the present procedure with the resulting simulation results. Thereby, two independent NNs were trained in a *multi*-NN structure, each allowing the prediction of the four MPs of the yield curve of the specific load case. In addition, a large *single*-NN was trained, with combined input from the simulation results of the tensile and compression tests, which was able to predict all eight MPs. Afterwards, the resulting MSE of the two architectures was compared, which was obtained after back-simulation with the predicted parameters and in comparison with the original simulation curves. The evaluation of the results showed an equal or even better prediction quality for the *single*-network. For this reason, this network architecture was also used for the work in this paper. In addition, the *single*-network architecture offers the advantage of enabling the NN to learn MPs from different trials, which may allow for the future prediction of MPs when the dataset is incomplete. Nevertheless, the *multi* or even mixed network structures should also be investigated in the future for the current data. In the present case of the *single*-network architecture, the 200 ordinate values of each simulation result were concatenated and provided to the NN in this form.

**Figure 10:**
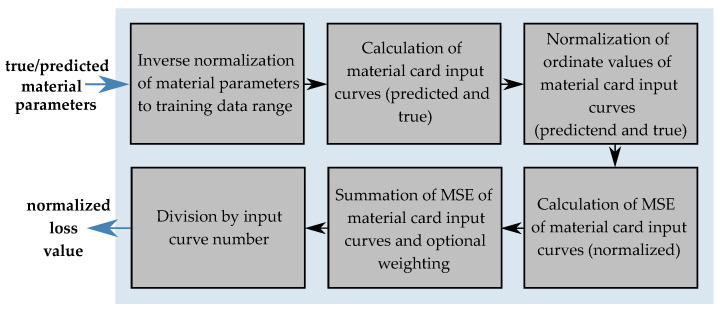
Process of the custom loss function calculation.

**Figure 11:**
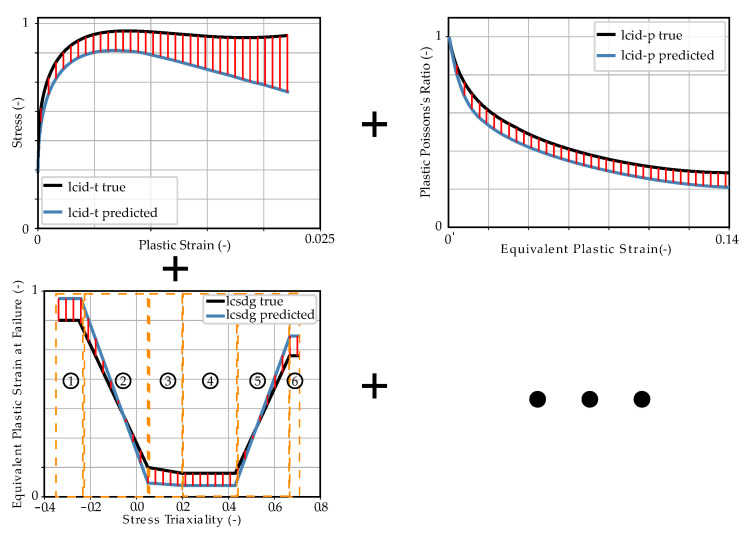
Exemplary calculation of the MSE of the material card input curves from true and predicted values and summation to the loss value (for EPSF vs. TRI separate curve segments).

To improve the assessment of the NN’s performance and to identify potential overfitting, a training/validation data separation is applied and additionally a cross-validation is performed. Afterwards, an FFANN is created and trained with the generated data by reversing the role of the inputs and outputs. During training, the outcome of the loss function is minimized in a usually gradient descent optimization. In the paper by Meißner et al. [15], the MSE resulting from true and predicted MPs was minimized. However, this is only appropriate to a limited extent, since, for instance, different MPs can lead to very similar or even the same analytical input curves of the material cards (e.g., yield curve). In addition, different yield curves can lead to very similar or even the same results of the FE simulation (e.g., FDCs). Unfortunately, the actual optimization objective, i.e., the error between the EXP and output of the simulation cannot be used here, since the results of the simulation with the predicted MPs are not available at the time of the GD optimization. However, preliminary experiments based on the dataset of Meißner et al. have shown that there is a higher correlation between the error of the simulation output curves and the error of the analytic input curves between the error of the SOC and the error of the MP. Therefore, in addition to the conventional loss function of the MSE of the parameters, a custom loss function was developed in this work and implemented in the NN. Using this, a summed normalized MSE of the analytical input curves of the NN is calculated for each MP dataset. The calculation process of the CLF is shown in Figure 10. The analytical input curves are calculated from the respective true and predicted MPs using tensor operations (see Figure 11).

In the first step, the respective MPs were transformed back to the original sizes by inverse normalization. Consequently, the analytical MCIC can be calculated using the formulas presented in Section 2.2. The ordinate values of these analytical curves are then normalized and the MSE between the respective true and predicted curves are calculated. In the present case, this was done for all input parameters, except for the two modules emod and bulk. For these two parameters, only the MSE of the normalized parameters were calculated. The MCIC *lcid-p* for consideration of the variable PPR was separated at EPS=0, since the negative strain range is calibrated from the compression test and the positive range is determined from the punch test. Thus, two curves are included in the calculation of the total loss. A similar procedure was used for the EPSF vs. TRI curve (see Figure 11), with the curve being divided into six individual segments which were included in the calculation of the final loss. Afterwards, the errors were summed up, whereby optionally a weighting of the respective segments can be made by adding constant factors. The final loss value for each input batch was obtained by dividing by the number of calculated MSE of the input curves (or parameters). In the present case, the final custom loss (CL) is given by

(26) $$\begin{array}{cc} CL= & \frac{1}{16}(MSE_{EMod}+MSE_{BMod}+MSE_{lcid-t}+MSE_{lcid-t1}+MSE_{lcid-t2}\\ \; & +MSE_{lcid-t3}+MSE_{lcid-t4}+MSE_{lcid-c}+MSE_{lcid-p_{c}}+MSE_{lcid-p_{t}}\\ \; & +MSE_{lcsdg1}+MSE_{lcsdg2}+\ldots +MSE_{lcsdg6}). \end{array}$$

Preliminary tests revealed an increase in the error of the SOC while the error of the MPs or of the CL remained constant, which indicates the overlearning of the NN that is difficult to detect. Hence, *early stopping* was implemented into the method, stopping the training of the NN at a defined number of trained epochs without any improvement in the validation loss.

Since the performance of the NN regarding the prediction accuracy significantly depends upon the selected HPs (e.g., kernel initializer and activation function), an optimization of these can be carried out in the following. Since the final aim is to achieve the highest possible agreement between the simulation and the real material behavior, the corresponding SOC of the validation set are then obtained by back simulation with the predefined and predicted MPs, and their error is compared using, e.g., DTW (as can be seen in Section 2.5.1). After training, the NN can finally be used to predict MPs for experimental input curves. Here, the virtually generated experimental dataset was used for validation and compared with the results of the iterative optimization-based method.

Similarly to the procedure of the iterative optimization-based method, different runs with varying settings of the NNs or the boundary conditions (dataset size) were performed (see Appendix A, Table A3). Three datasets of different sizes (see Table 2) were used for the training and evaluation of the NN_Runs. One dataset consists of the ten FE simulations of the tests and the respective twelve evaluated SOCs as well as the corresponding MPs of the material card(s).

In addition to the investigations with a predefined default NN (see Appendix A, Table A4), different HPOs (see Appendix A, Table A6) were conducted and investigations regarding the prediction accuracy were carried out using the resulting NNs (see Appendix A, Table A6). The corresponding HP search range is presented in Appendix A, Table A5. Some HP combinations in connection with the used CLF resulted in unstable behavior during the training. These were: the activation functions *Linear* (not in OL), *Selu*, *Elu*, *Tanh*, *Exponential* as well as the kernel initializer *Zero* and the GD optimizer *Adadelta*. Often, these HPs led to abrupt increases in the loss value during training, which became so large that they caused the training process to stop due to NAN values. Therefore, these HPs were excluded from HP optimization, resulting in a stable training process without any problems.

## 3. Results and Discussion

**Figure A4:**
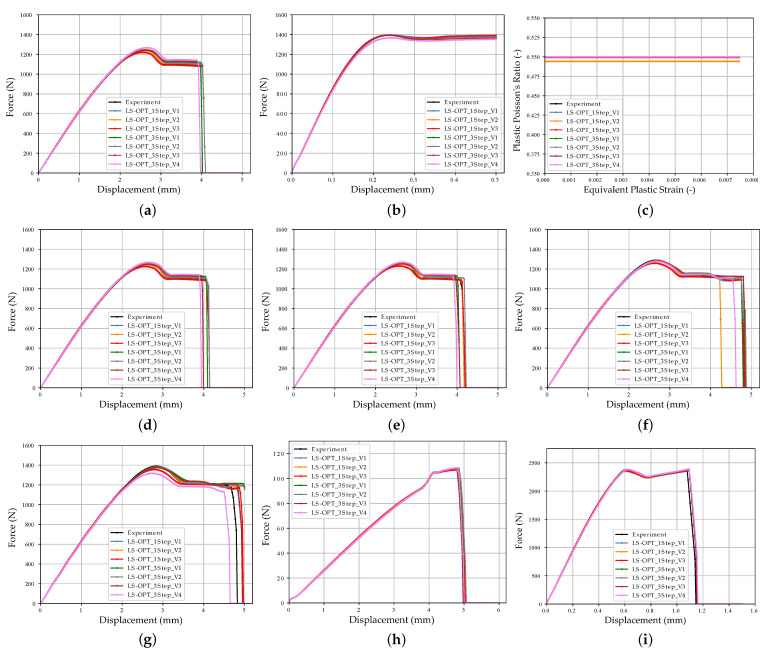

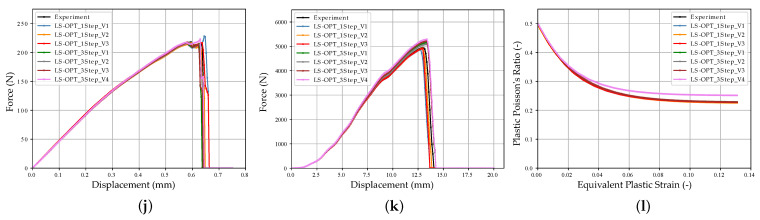
Simulation output curves of the LS-OPT runs with the virtual experimental test dataset: (**a**) FDC tension test; (**b**) FDC compression test; (**c**) PEC compression test; (**d**) FDC tension tests V1; (**e**) FDC tension tests V2; (**f**) FDC tension tests V3; (**g**) FDC tension tests V4; (**h**) FDC bending test; (**i**) FDC shear1 test; (**j**) FDC shear2 test; (**k**) FDC punch test; and (**l**) PEC punch test.

For both MPI methods used, a systematic experimental scheme was created with varying settings. The experimental design of the optimization-based method including the results is shown for the 1 Step Strategy in Appendix A, Table A1 and for the 3 Step Strategy in Table A2. As already mentioned in Section 2.5.1 and Section 2.5.2, the dimensionless dynamic time warping distance was used as evaluation criteria in addition to the qualitative SOC curve shapes. This distance was calculated for the virtual experimental and the validation dataset between true and predicted curves. The mean value was calculated for each of the twelve SOC. Nevertheless, the DTW distance is rather to be taken as a qualitative dimensionless comparative metric. A doubling of the DTW does not necessarily result in twice as bad reproduction quality compared to the reference curve. The SOCs for the optimization-based method are shown in Figure A4, Appendix A.

**Figure 12:**
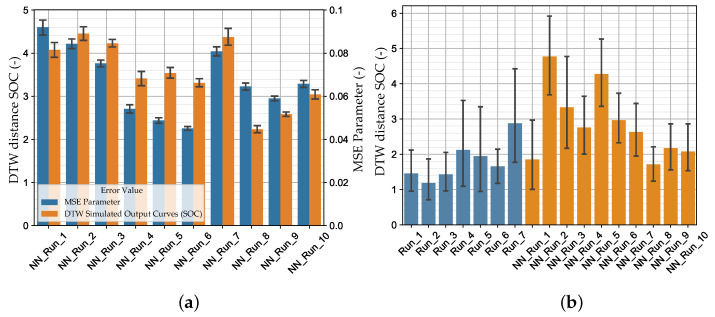
Comparison of the distance measures of (**a**) the NN-based method for the validation dataset and (**b**) the optimization-based and the NN-based method for the virtual experimental dataset.

Generally, the runs of the 1 Step Strategy achieved better results than the runs of the 3 Step Strategy. The maximum number of specified iterations of the 1 Step Strategy was fully exploited, whereas with the 3 Step Strategy, the optimization was partially prematurely terminated. The best results were achieved by Run_2 with an elliptic metamodel order and the optimization algorithm ASA/LFOP. The best results with the 3 Step Strategy were obtained with a linear metamodel order and the GA/LFOP optimization algorithm. The worst results using the optimization-based MPI method were achieved with the 3 Step Strategy and the MSE chosen as the Distance Measure in the first two optimization steps. The results indicate an advantage for the optimization algorithm to have information about the full MP range within each iteration, as in the 1 Step Strategy. Furthermore, in a multiple steps strategy, error-prone calibrated MPs from previously passed steps cannot be recalibrated, which leads to error propagation. However, the advantages of this strategy can be a reduction in computation time as well as a reduction in the tendency to determine local optima. Nevertheless, with all runs, MPs could be calibrated, allowing the reproduction of the nonlinear material behavior of the thermoplastic polymer investigated. In Figure 12b, the results of the optimization-based method are compared with the NN-based method for the virtual experimental dataset (in all bar charts, a 95% confidence interval is shown).

In Figure 12a, the MSE of the MPs and the DTW distance of the SOCs of the NN_Runs for the validation dataset are compared. As expected, the NN_Runs executed with HPO (NN_Run_8–NN_Run_10) achieved the best results (lowest validation error DTW). The best results were obtained with NN_Run_8, for which the medium-sized dataset (2) and the HPO with the Bayesian optimizer were used. Contrary to expectations, using the larger datasets and the CLF (NN_Run_3 and NN_Run_9) resulted in a comparatively high or constant error. Only when using the standard MSE of the MPs as a loss function, a larger dataset resulted in a slight decrease in the mean DTW distance of the VAL set. However, with the increasing dataset size, both loss types used (MSE and CL, see Table A3) and the standard deviation of the error values decreased (Figure 12a).

Using the default MSE, even lower mean DTW distances of the VAL set were obtained compared to using the CLF with a default NN. Thereby, a significant reduction in the MSE of the MPs using the MSE as loss function can be seen. As already described in [15], in the present investigations, a reduction in the MSE of the MPs also does not necessarily lead to a reduction in the actual optimization objective (mean DTW SOC VAL set). Contrary to the preliminary investigations based on the MPs for tensile and compressive plasticity, this also applies to the CLF, which can be identified in the example of NN_Run_8 and NN_Run_9 (see Table A3) by the reduction in the CL value with a simultaneous increase in the DTW SOC. A reason for this is assumed to be the varying sensitivity of the MP. The normalized MPs feed into the material card depending on the frequency of occurrence in the MCIC. For example, the MPs and/or the MCIC describing the variable PPR have less influence on the mean DTW SOC than the MPs of the tension plasticity. Finally, the normalized total loss is minimized during optimization. However, some MPs may have a stronger influence on the actual optimization objective (mean DTW SOC) than others. Since during training this information is not available due to the missing results from the back simulation, it cannot be considered in the GD optimization. Thus, a separate prediction of the MPs of the different material characteristics using an appropriate *multi*-network architecture would be worth investigating in the future. Although this is not possible without further consideration, since MPs of different material characteristics are included in the same MCIC (e.g., strain rate-dependent lcid-t). However, the significantly lower DTW distances of SOCs could be obtained when using the CLF and the HPO. This indicates the high influence of the network parameters on prediction accuracy. Therefore, the advantage of the CLF is assumed to only come into effect in combination with appropriate network settings (HP). Furthermore, the introduction of scaling factors in the NN_Run_7 did not reduce the mean DTW SOC of the VAL set.

**Figure A5:**
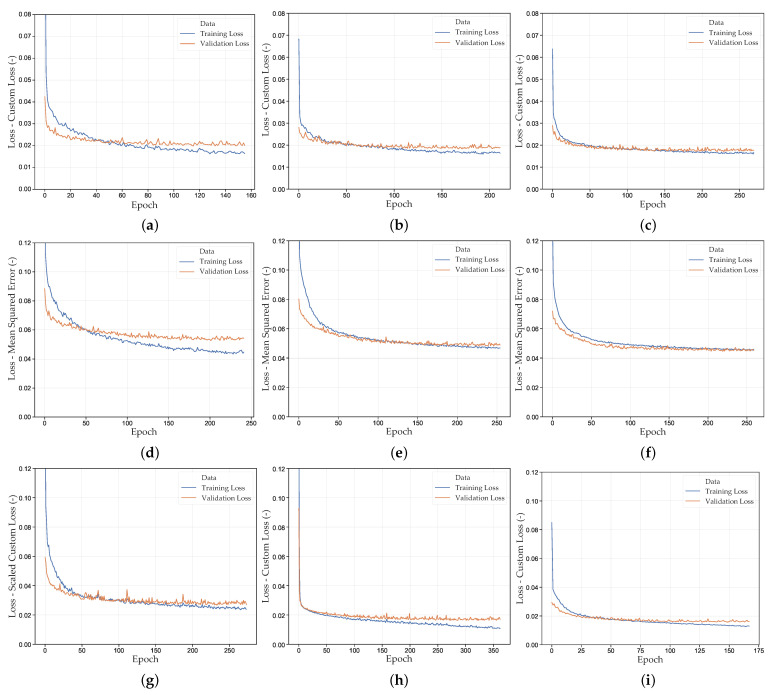

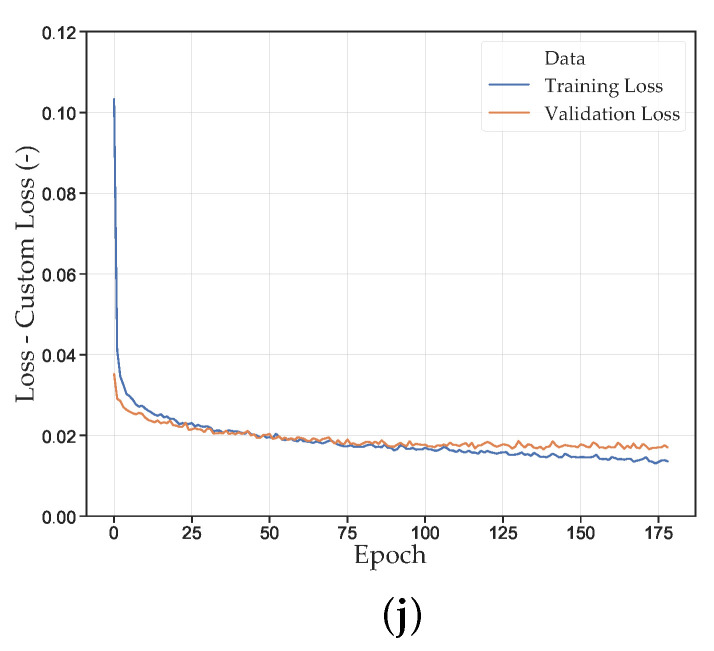
NN_Run learning curves for the training and validation dataset: (**a**) NN_Run_1; (**b**) NN_Run_2; (**c**) NN_Run_3; (**d**) NN_Run_4; (**e**) NN_Run_5; (**f**) NN_Run_6; (**g**) NN_Run_7; (**h**) FDC NN_Run_8; (**i**) FDC NN_Run_9; and (**j**) NN_Run_10.

In Appendix A, Figure A5, the learning curves of the NN_Runs are shown. Again, an increasing dataset size shows a decrease in the fluctuation of the respective loss value. Furthermore, with increasing dataset size, the lowest validation error tends to be reached later. This also applies to the validation error plateau, from which the error is only marginally reduced. Presumably, this is due to the ability of the NN to learn the relationship between input and output more precisely with a larger amount of data. This is also evident in the ratio of training loss to validation loss, with a smaller gap between the two curves for larger datasets. With smaller datasets, the training loss is usually further minimized, whereas the validation loss remains constant or even increases again. This again emphasizes the necessity of *early stopping* to reduce the overlearning tendency. Furthermore, a significantly earlier achievement of a low loss value can be observed when using the HP optimized FFANNs. However, for these networks, the overlearning risk increases, which can be seen in the further decreasing training loss at constant validation loss. Notably, in preliminary studies, NNs with up to 500,000 neurons were trained, which had a rather negative effect on the prediction accuracy. Thereby, the fluctuation of the loss value also increased and the validation optimum was reached at earlier epochs. However, the strong fluctuation and larger validation loss with a high number of neurons could be counteracted with an increasing dropout rate.

**Figure 13:**
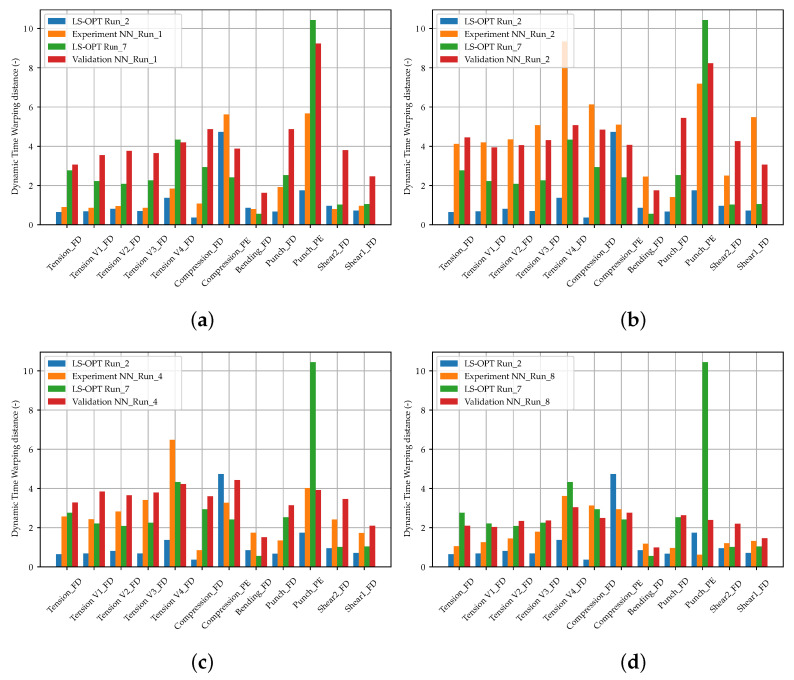
Bar plot comparison of the results of selected LS-OPT_Runs and NN_Runs for the SOCs of the different tests for the EXP dataset. The best and worst LS-Opt_Run were compared with (**a**) NN_Run_1 (second best NN_Run); (**b**) NN_Run_2 (worst NN_Run); (**c**) NN_Run_4 (best NN_Run with MSE Loss); and (**d**) NN_Run_8 (best NN_Run).

In Figure 12b, the DTW distances of the NN_Runs for the virtual experimental dataset are shown and compared with the results of the optimization-based method. In Figure 13, the results of selected LS-OPT_Runs and NN_Runs for the SOCs of the different tests are compared and in Appendix A, Figure A7, the corresponding SOCs are shown (see Supplementary Materials for plots of all NN_Runs).

The best NN_Runs results for the virtual experimental dataset were obtained from NN_Run_8, followed by NN_Run_1. This matches only partially the expectations because, in contrast to NN_Run_8, NN_Run_1 exhibits a rather high validation error. However, only one experimental dataset was investigated here. Hence, in future studies, verifications should be performed using larger experimental datasets. In contrast, the NN_Run_2 exhibited the highest DTW distance for the EXP set. As for the validation dataset, the NN_Runs with MSE as the loss value resulted in a lower DTW distance in contrast to runs with CL without HPO. Accordingly, the best results were obtained using CLF with hyperparameter optimization.

**Figure A6:**
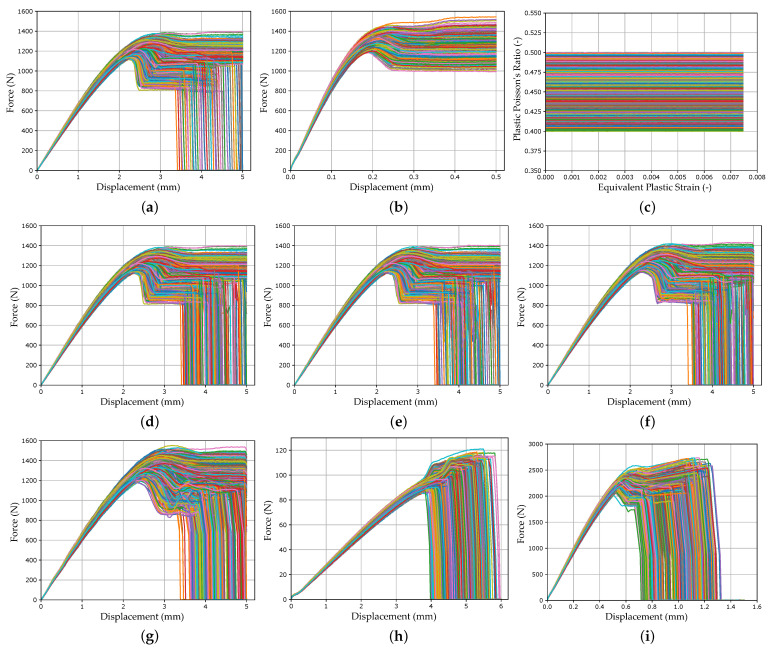

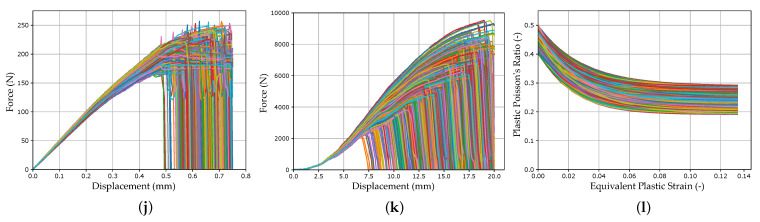
Simulated output curves of the tests for training data of dataset 2: (**a**) FDC tension test; (**b**) FDC compression test; (**c**) PEC compression test; (**d**) FDC tension tests V1; (**e**) FDC tension tests V2; (**f**) FDC tension tests V3; (**g**) FDC tension tests V4; (**h**) FDC bending test; (**i**) FDC shear1 test; (**j**) FDC shear2 test; (**k**) FDC punch test; and (**l**) PEC punch test.

**Figure A7:**
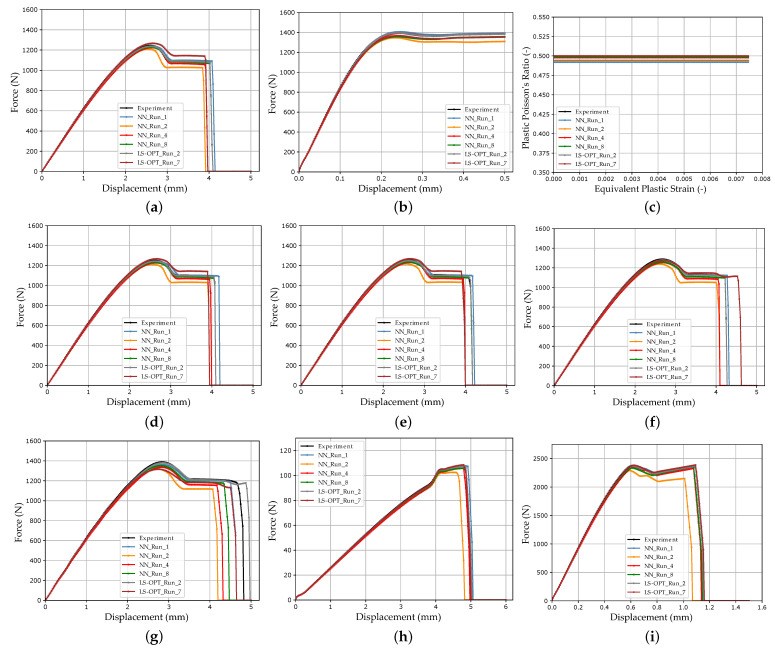

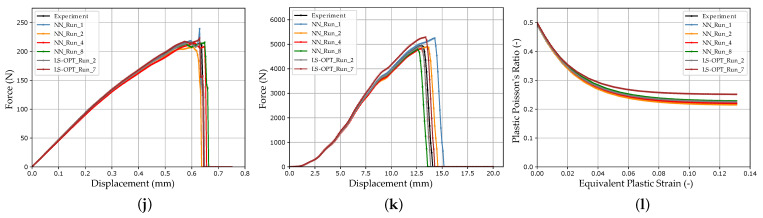
Comparison of the simulation output curves of selected LS-OPT and NN runs with the virtual experimental test dataset: (**a**) FDC tension test; (**b**) FDC compression test; (**c**) PEC compression test; (**d**) FDC tension tests V1; (**e**) FDC tension tests V2; (**f**) FDC tension tests V3; (**g**) FDC tension tests V4; (**h**) FDC bending test (**i**) FDC shear1 test; (**j**) FDC shear2 test; (**k**) FDC punch test; and (**l**) PEC punch test.

The tendency of the VAL DTW distance to be larger than the EXP set is noticeable. One reason for this could be the tendency of the DTW distance to be higher in the peripheral areas of the MP ranges, which increases the VAL DTW on average. In contrast, the EXP set is more centrally located in the MP range. Furthermore, in the NN-based method model, errors are learned from the FFANN. For instance, in the training dataset, not all oscillations or deflections after failure could always be filtered out in the shear test two (Figure A6j), causing them to be learned as well. In the prediction, the NN consequently has problems matching these MPs for these curves, which results in relatively high DTW values. These then lead to an increased VAL DTW distance. In future studies, the processing of the labeled data should therefore be further improved to enable the NN to better learn the input–output relationship and make the loss more representative.

The failure points of the tensile tests and the punch test exhibit an especially increased dispersion. The SOCs of the training data range of the tensile tests (see Appendix A, Figure A6a,d–g), show the failure of only a subset of the simulations performed within the maximum displacement. This reduction in information density regarding failure could lead to reduced prediction accuracy. Similarly, for MPI using LS-OPT, the sufficient calibration of the failure point proved to be difficult, with partially no failure calibrated for tensile test V4. On the other hand, the SOCs of the punch test training data range (see Appendix A, Figure A6k) show a very wide range of properties for the specified MP range. Hence, the reason for the difficulty in calibrating the punch test failure could be due to the relatively underrepresented information density of the failure time points. Once again, these insights highlight the importance of the appropriate choice of MP ranges. A key for better prediction accuracies in the future could be a sensitivity-based sampling, whereby a higher number of test points is placed in the MP range, which has a higher influence on the simulation results.

The comparison of the two MPI methods (Figure 12) reveals that the optimization-based method for the virtual experimental dataset still achieves better results than the NN-based method. However, with an appropriate choice of hyperparameters and network structure, the NN-based method also achieves very good results. For the investigated dataset, some NNs could achieve better results than some optimization-based runs. Of course, this depends on the choice of settings and the precision could surely be improved with the conventional method using LS-OPT. However, the results of the two methods are at a comparable level of precision, with the NN-based method having the distinct advantage of reusability. Nevertheless, future improvements could further increase the prediction accuracy of the NN-based method, which could help to apply the method in daily engineering work.

## 4. Conclusions and Outlook

In this paper, a NN-based method was presented to identify the material parameters of the *MAT_187_SAMP-1* and *MAT_ADD_DAMAGE_GISSMO* material cards in LS-DYNA for numerical simulation. The required labeled data were generated by a large number of numerical simulations and the FFANN was trained using these data. The feedforward artificial neural network was trained to learn the input–output relationship between test curves (e.g., FDCs) and the corresponding MPs for the numerical simulation. For verification purposes, an experimental dataset was virtually generated, which was based on an additively processed thermoplastic polymer (ABS) and exhibits pronounced nonlinearities. The FFANN was thus designed to predict a total of 19 MPs to account for elasticity, plasticity, tension–compression asymmetry, variable plastic Poisson’s ratio, strain rate dependency and failure. Various influential parameters, such as the choice of HPs, which influence the prediction accuracy of the FFANN, were investigated in studies. For comparison purposes, the conventional iterative optimization-based method was applied to the same dataset using the commercial software LS-OPT in different variants.

The results proved the general applicability of the presented NN-based method for the identification of MPs for highly complex material cards. Although better results could be obtained with certain variants of the conventional optimization-based method, good results could also be achieved with some NN variants. However, with the presented NN-based MPI method, material cards can be calibrated in a few seconds. This represents a significant time advantage compared to the conventional optimization-based method. Depending on the available hardware and software resources, a few seconds for the prediction of the material card stood in contrast to several days for the optimization-based calibration. The investigations showed the importance of the appropriate choice of the MP ranges as well as the network settings. Furthermore, an alternative custom loss function was presented, which has to be minimized during the NN training using gradient descent optimization. Within this function, the true and predicted MPs were used to compute the analytical functions that represent the input of the material cards. In connection with an HPO, the custom loss function showed promising results.

In order to further increase the prediction accuracy of the NN-based method and thus enable its application in everyday engineering work, further efforts and investigations have to be conducted. In addition to additional runs with varying optimizers and settings for evaluation, further network architectures and types should be reviewed and compared. Convolutional NN, as well as Bayesian NN, could be suitable options to further increase the prediction accuracy and counteract the network’s tendency to overlearn. In the present work, the application of Gaussian noise to the training data was already used to increase the generalization capability of the NN. In the future, different noise levels could also be applied, which would increase the number of training data without additional simulations. Furthermore, a continued investigation should be conducted regarding the optimization goal when training the NN in terms of the custom loss function. This could be a key factor to compensate for the advantage of the conventional MPI method consisting in the optimization on the final MPI objective. In contrast to minimizing the error between the output of the simulation and the experiment, only the error of the input of the material card between the true and predicted can be minimized with the NN-based method. Moreover, the applicability of the method to predict MPs for highly complex material cards should be verified using real experimental datasets. Since the NN also learns the boundary conditions and other settings of the FE model, the matching of the simulation model to the experimental test must be ensured in order to maintain prediction accuracy.

From the author’s point of view, the presented NN-based method has a high potential for the simulation-based product development process and is not limited to specific materials. Using a trained NN, MPs for the subsequent numerical simulation can be predicted in a few seconds and thus designs and materials can be compared as soon as the early concept phases. Often, this is not possible due to time-consuming and cost-intensive calculation efforts for each material. Another advantage results from the non-required expert knowledge regarding continuum mechanics, numerical optimization and the materials science of the actual users of the method, which include development and design engineers. Thus, the method represents an enabler to further incorporate structural simulations into the product development process. Moreover, the NN-based method can be used to determine suitable starting parameters for the conventional optimization-based method within a minimal amount of time. Thus, conventional development processes could also be accelerated and possibly lead to more accurate results.

## Figures and Tables

**Table 1:** Material parameters of the virtual experimental dataset and the corresponding MP ranges for the applied MPI methods. Additionally, the starting values for the optimization-based MPI method and the corresponding material card input curve names are listed.

MP Name	MP_exp_	MP_Min_	MP_Max_	MP_Start_	MCIC
emod (MPa)	2127.3	2000.0	2300.0	2100.0	-
bulk (MPa)	2250.0	2100.0	2400.0	2200.0	-
a_t_ (-)	43,416.1	40,000.0	46,000.0	44,000.0	lcid-t; lcid-t1-lcid-t4
b_t_ (-)	−3934.39	−4100.00	−3700.00	−4000.00	lcid-t; lcid-t1-lcid-t4
c_t_ (-)	9.702	8.000	10.500	9.000	lcid-t; lcid-t1-lcid-t4
d_t_ (-)	551.24	540.00	562.00	550.00	lcid-t; lcid-t1-lcid-t4
a_c_ (-)	51,000.0	47,000.0	52,000.0	50,000.0	lcid-c
b_c_ (-)	−3900.00	−4100.00	−3800.00	−4000.00	lcid-c
c_c_ (-)	12.500	9.500	13.000	11.500	lcid-c
d_c_ (-)	550.00	540.00	560.00	555.00	lcid-c
ν_p,plat_ (-)	0.2265	0.1900	0.2900	0.2500	lcid-p
ν_p,press_ (-)	0.5000	0.4000	0.5000	0.4500	lcid-p
ϵ_p,plat_ (-)	0.1232	0.1000	0.1500	0.1300	lcid-p
C ($\frac{1}{s}$)	27,572.1	15,000.0	50,000.0	30,000.0	lcid-t1-lcid-t4
P (-)	3.7482	2.7000	4.2000	3.2000	lcid-t1-lcid-t4
epsf_0_ (-)	0.3000	0.2500	0.4500	0.3500	lcsdg
epsf_1_ (-)	0.0500	0.0400	0.0550	0.0450	lcsdg
epsf_2_ (-)	0.0400	0.0340	0.0460	0.0350	lcsdg
epsf_3_ (-)	0.2400	0.2100	0.2800	0.2200	lcsdg

**Table 2:** Datasets for the training and validation of the investigations using the NN-based method.

Dataset	Sampling	Training Set Size	Validation Set Size	Complete Set Size
1	LHS	450	300	750
2	LHS	900	600	1500
3	LHS	1800	1200	3000

## Data Availability

Additional plots and graphics are provided in the Supplementary Materials of this paper.

## Supplementary Materials

The following supporting information can be downloaded at: https://www.mdpi.com/article/10.3390/ma15020643/s1, Additional plots and graphics of the results.

## Abbreviations

The following abbreviations are used in this manuscript:

ABS	Acrylonitrile Butadiene Styrene
ANN	Artificial Neural Network
ASA	Adaptive Simulated Annealing
CL	Custom Loss
CLF	Custom Loss Function
DIC	Digital Image Correlation
DOE	Design of Experiments
DTW	Dynamic Time Warping
EPS	Equivalent Plastic Strain
EPSF	Equivalent Plastic Strain at Failure
EXP	Experiment
FDC	Force–Displacement Curve
FE	Finite Element
GA	Genetic Algorithm
GBM	Gradient-Based Methods
GISSMO	Generalized Incremental Stress State-Dependent Damage Model
GD	Gradient Descent
HL	Hidden Layer
HP	Hyperparameter
HPO	Hyperparameter Optimization
IL	Input Layer
LFOP	Leap-Frog Algorithm
LH	Latin Hypercube
LHS	Latin Hypercube Sampling
MDPI	Multidisciplinary Digital Publishing Institute
ML	Machine Learning
MP	Material Parameter
MPI	Material Parameter Identification
MSE	Mean Squared Error
MCIC	Material Card Input Curve(s)
NAN	Not a Number
NN	Neural Network
OL	Output Layer
PEC	Plastic Poisson’s Ratio Equivalent Plastic Strain Curve
PI	Parameter Identification
PPR	Plastic Poisson’s Ratio
RSM	Response Surface Methodology
SOC	Simulation Output Curve(s)
TRI	Triaxiality
VAL	Validation
VPPR	Variable Plastic Poisson’s Ratio

## Appendix A

**Table A1:** LS-OPT settings of 1 Step Strategy for iterative optimization-based calibration and results (*—except for PEC Compression Test; **—when the global optimum is found, switch to LFOP to find best local optimum; ***—default).

Setting	Run_1	Run_2	Run_3
Sampling Point Selection	D-Optimal	D-Optimal	D-Optimal
Simulations per Iteration	40	100	40
Metamodel	Polynomial	Polynomial	Polynomial
Metamodel Order	Linear	Elliptic	Linear
Distance Measure	DTW *	DTW *	DTW *
Optimization Algorithm	ASA/LFOP **	ASA/LFOP **	GA/LFOP **
Maximum Iterations	23	9	23
Design Change Tolerance	0.01 ***	0.01 ***	0.01 ***
Objective Function Tolerance	0.01 ***	0.01 ***	0.01 ***
Required Iterations	23	9	23
Mean DTW SOC Exp. Set (-)	1.4526	1.1849	1.4301

**Table A2:** LS-OPT settings of 3 Step Strategy for iterative optimization-based calibration and results (*—except for PEC Compression Test; **—when global optimum is found, switch to LFOP to find best local optimum; ***—default).

Setting	Run_4	Run_5	Run_6	Run_7
Sampling Point Selection	D-Optimal	D-Optimal	D-Optimal	D-Optimal
Simulations per Iteration	40; 10; 20	45; 10; 20	40; 10; 20	40; 10; 20
Metamodel	Polynomial	Polynomial	Polynomial	Polynomial
Metamodel-Order	Linear	Elliptic	Linear	Linear
Distance Measure	DTW *; DTW *; DTW *	DTW *; DTW *; DTW *	DTW *; DTW *; DTW *	MSE; MSE; DTW *
Optimization Algorithm	ASA/LFOP **	ASA/LFOP **	GA/LFOP **	ASA/LFOP **
Maximum Iterations	20; 10; 15	18; 10; 15	20; 10; 15	20; 10; 15
Design Change Tolerance	0.01 ***	0.01 ***	0.01 ***	0.01 ***
Objective Function Tolerance	0.01 ***	0.01 ***	0.01 ***	0.01 ***
Required Iterations	18; 10; 14	15; 8; 13	20; 10; 15	20; 2; 15
Mean DTW SOC Exp. Set (-)	2.1246	1.9493	1.6545	2.8808

**Table A3:** Settings and results of the NN-based MPI runs (*—single segments of the custom loss function were scaled with a factor: $emod_{MSE}\times 1.5$, $bulk_{MSE}\times 1.5$, $lcid-t_{MSE}\times 2$, $lcid-c_{MSE}\times 6$, $lcid-p{\left(c\right)}_{MSE}\times 2$, $lcid-p{\left(t\right)}_{MSE}\times 2$, $lcsdg{\left(3\right)}_{MSE}\times 2$, $lcsdg{\left(4\right)}_{MSE}\times 4$, $lcsdg{\left(5\right)}_{MSE}\times 4$).

Run	Dataset	NN	Loss	Early Stopped Epoch	Loss Val. Set (-)	Mean DTW SOC Val. Set (-)	Mean DTW SOC Exp. Set (-)
NN_Run_1	1	Default	CL	157	0.0200	4.0773	1.8521
NN_Run_2	2	Default	CL	212	0.0189	4.4550	4.7747
NN_Run_3	3	Default	CL	270	0.0177	4.2278	3.3339
NN_Run_4	1	Default	MSE	242	0.0541	3.4139	2.7574
NN_Run_5	2	Default	MSE	255	0.0488	3.5369	4.2746
NN_Run_6	3	Default	MSE	260	0.0451	3.3119	2.9638
NN_Run_7	2	Default	CL (scaled *)	274	0.0269	4.3733	2.6311
NN_Run_8	2	HPO1	CL	363	0.0170	2.2341	1.7116
NN_Run_9	3	HPO2	CL	168	0.0161	2.5824	2.1712
NN_Run_10	2	HPO3	CL	179	0.0170	3.0430	2.0807

**Table A4:** Default Neural Net settings.

(Hyper-)Parameter	Setting
Batch Size	25
Maximum Epochs	400
Early Stopping Patience	40
Neurons (IL)	2400
Hidden Layers	1
Neurons (HL)	100
Kernel Initializer (HL)	He Uniform
Activation (HL)	Hard Sigmoid
Dropout (HL)	0.10
Neurons (Output Layer)	19
Kernel Initializer (OL)	He Uniform
Activation (OL)	Linear
Gradient Descent Optimizer	Adam

**Table A5:** Hyperparameter optimization search range (* = default parameter).

(Hyper-)Parameter	Search Range
Batch Size	20; 25 *; 30; *…*; 150
Number HL	1 *; 2; 3
Neurons (HL1)	30; 40; 50 *; *…*; 500
Kernel Initializer (HL)	Normal *; Uniform; Glorot Uniform; Lecun Uniform; Glorot Normal; He Normal; He Uniform
Activation (HL1)	Softmax; Softplus; Softsign; Relu *; Sigmoid; Hard Sigmoid
Dropout (HL1)	0.000; 0.025; 0.050 *; *…*; 0.250
Neurons (HL2)	30; 40; 50 *; *…*; 500
Kernel Initializer (HL2)	Normal *; Uniform; Glorot Uniform; Lecun Uniform; Glorot Normal; He Normal; He Uniform
Activation (HL2)	Softmax; Softplus; Softsign; Relu *; Sigmoid; Hard Sigmoid
Dropout (HL2)	0.000; 0.025; 0.050 *; *…*; 0.250
Neurons (HL3)	30; 40; 50 *; *…*; 500
Kernel Initializer (HL3)	Normal *; Uniform; Glorot Uniform; Lecun Uniform; Glorot Normal; He Normal; He Uniform
Activation (HL3)	Softmax; Softplus; Softsign; Relu *; Sigmoid; Hard Sigmoid
Dropout (HL3)	0.000; 0.025; 0.050 *; *…*; 0.250
Kernel Initializer (OL)	Normal *; Uniform; Lecun Uniform; Glorot Normal; He Normal; He Uniform
GD Optimizer	Adam *; Adagrad; Adamax; Nadam

**Table A6:** Neural Net settings from hyperparameter optimization (* = default settings).

(Hyper-)Parameter	HPO1	HPO2	HPO3
Batch Size	85	25	50
Maximum Epochs *	400	400	400
Early Stopping Patience *	40	40	40
Neurons (IL) *	2400	2400	2400
Number HL	1	2	2
Neurons (HL1)	460	470	450
Kernel Initializer (HL1)	He Uniform	He Uniform	He Normal
Activation (HL1)	Hard Sigmoid	Relu	Softplus
Dropout (HL1)	0.000	0.000	0.000
Neurons (HL2)	-	470	310
Kernel Initializer (HL2)	-	Uniform	Normal
Activation (HL2)	-	Softplus	Relu
Dropout (HL2)	-	0.025	0.150
Neurons (OL) *	19	19	19
Kernel Initializer (OL)	Lecun Uniform	He Uniform	Uniform
Activation (OL) *	Linear	Linear	Linear
Loss Function *	Custom Loss	Custom Loss	Custom Loss
GD Optimizer	Adamax	Adamax	Adamax
HP Optimizer	Bayesian	Bayesian	Random Search
Max Trials *	500	500	500
Executions per Trial *	2	2	2
