# Supplementary figures and images for: Methodology for Neural Network-Based Material Card Calibration Using LS-DYNA MAT_187_SAMP-1 Considering Failure with GISSMO (part 2 of 2)
Source: Materials (Basel). 2022 Jan 15;15(2):643. doi: 10.3390/ma15020643 (PMC8778971; doi:10.3390/ma15020643)

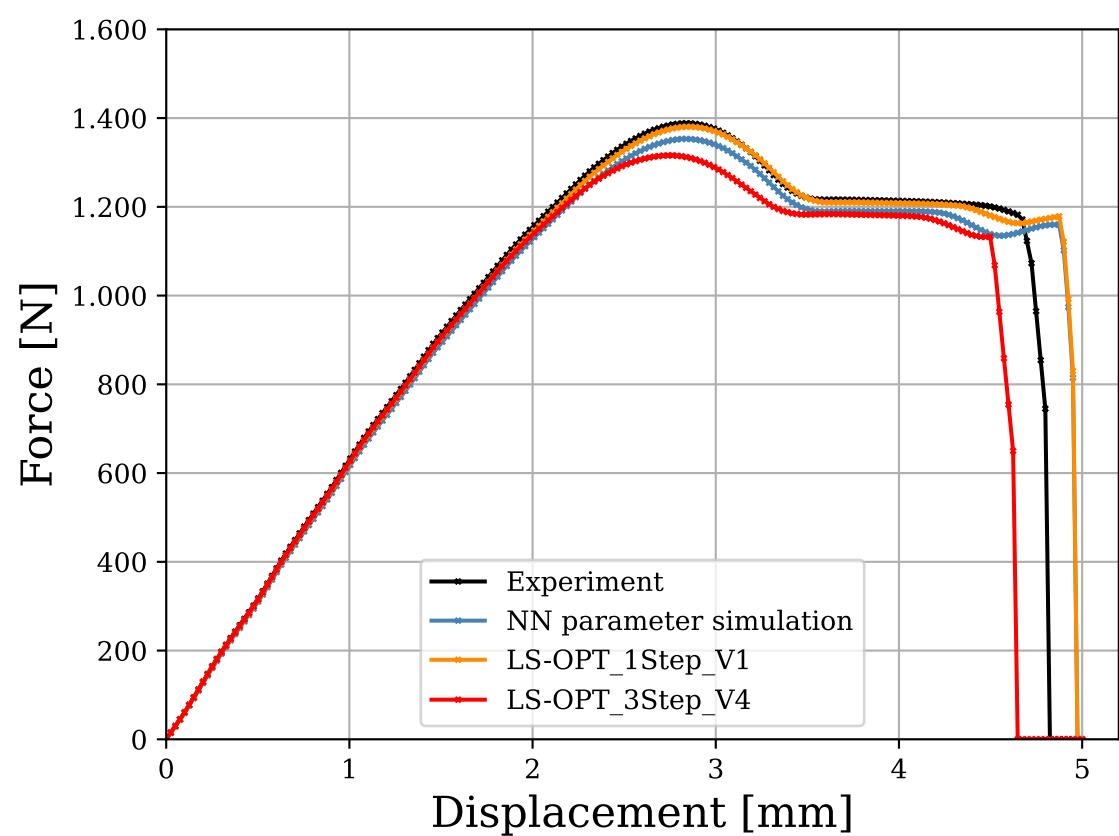

Supplement: Supplementary file 1 [file materials-15-00643-s001.zip › Supplementary_Material/SOC_NN_Pred_LSOPT_Complete/NN_Run_3/FD_Comparison_Tensile_Test_V4.pdf]

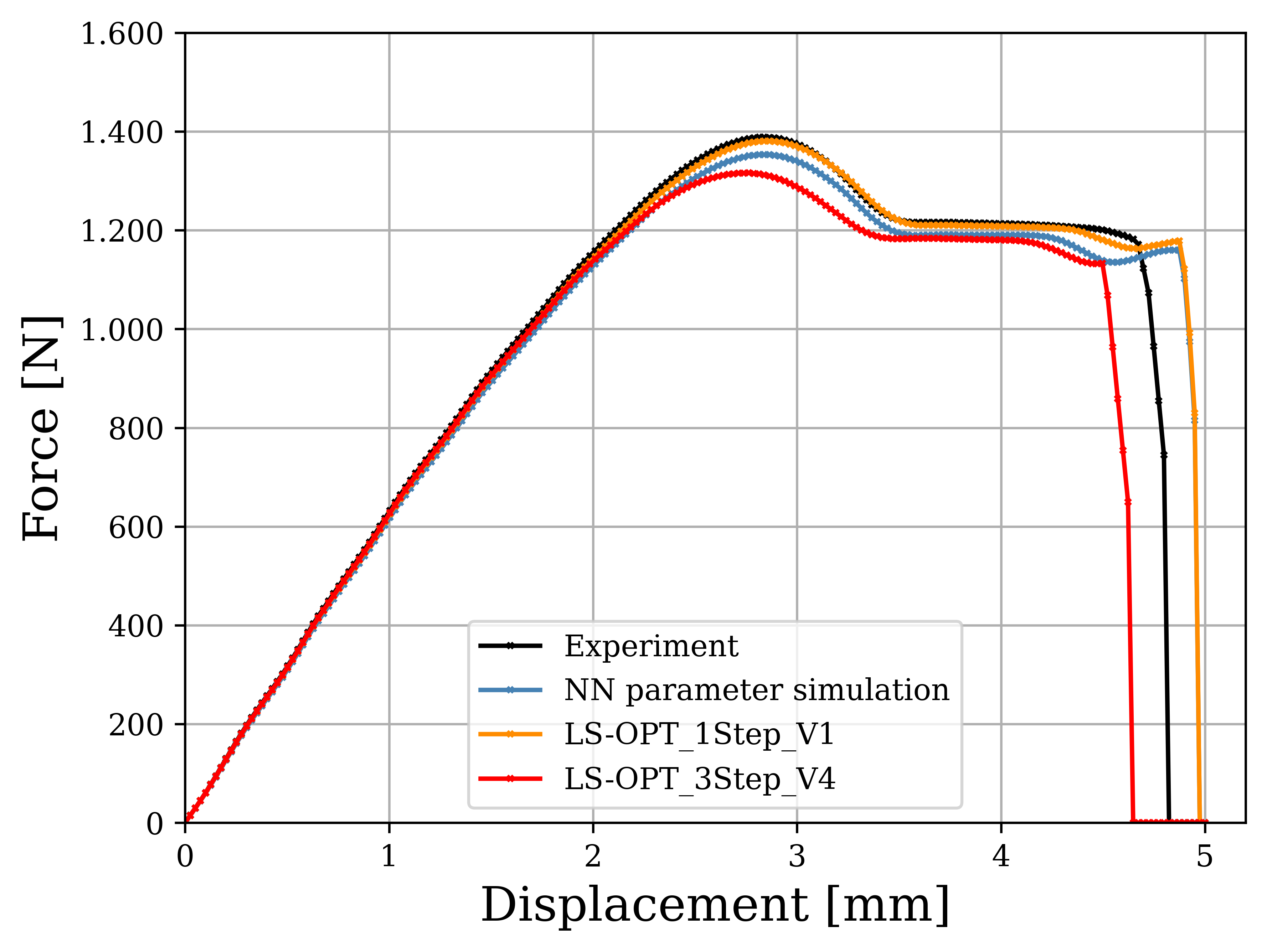

Supplement: Supplementary file 1 [file materials-15-00643-s001.zip › Supplementary_Material/SOC_NN_Pred_LSOPT_Complete/NN_Run_3/FD_Comparison_Tensile_Test_V4.png]

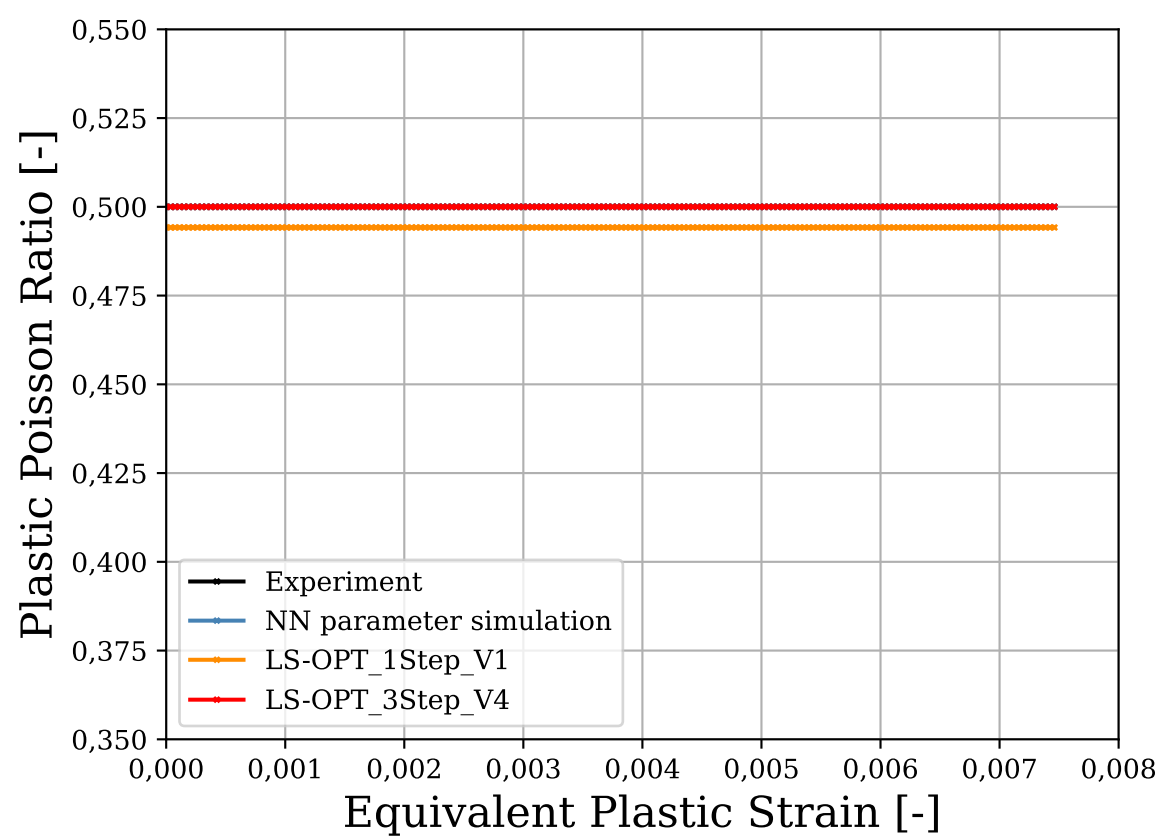

Supplement: Supplementary file 1 [file materials-15-00643-s001.zip › Supplementary_Material/SOC_NN_Pred_LSOPT_Complete/NN_Run_3/PE_Comparison_Compression_Test.pdf]

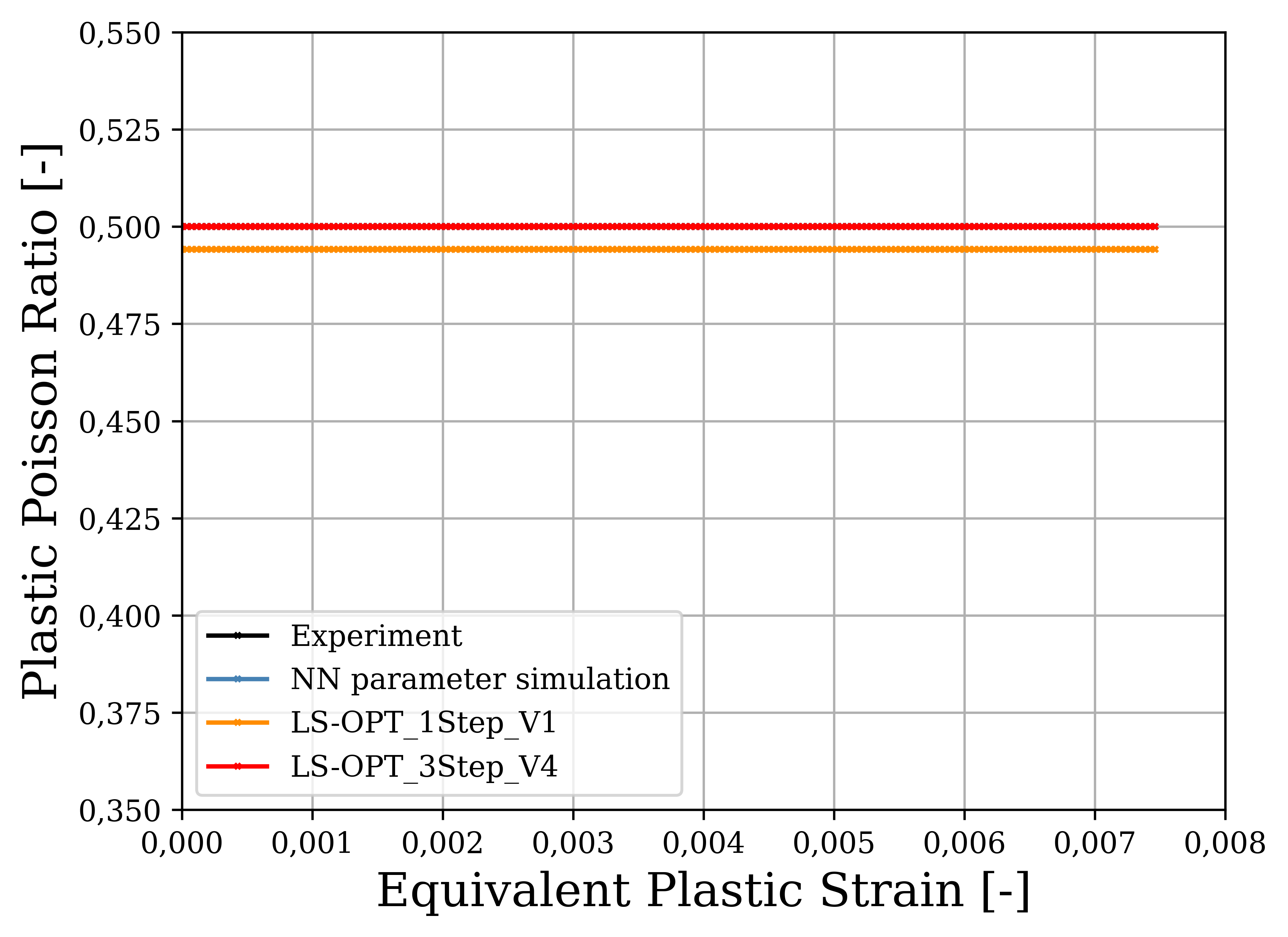

Supplement: Supplementary file 1 [file materials-15-00643-s001.zip › Supplementary_Material/SOC_NN_Pred_LSOPT_Complete/NN_Run_3/PE_Comparison_Compression_Test.png]

Plastic Poisson Ratio [-]

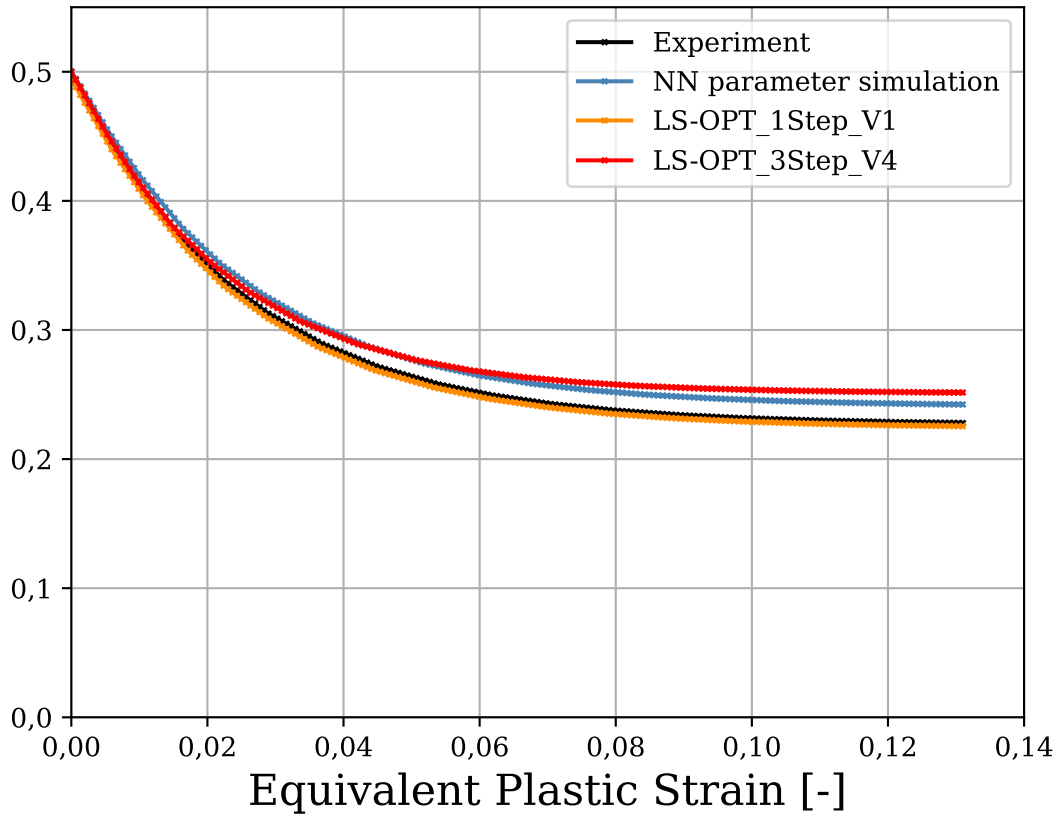

Supplement: Supplementary file 1 [file materials-15-00643-s001.zip › Supplementary_Material/SOC_NN_Pred_LSOPT_Complete/NN_Run_3/PE_Comparison_Punch_Test.pdf]

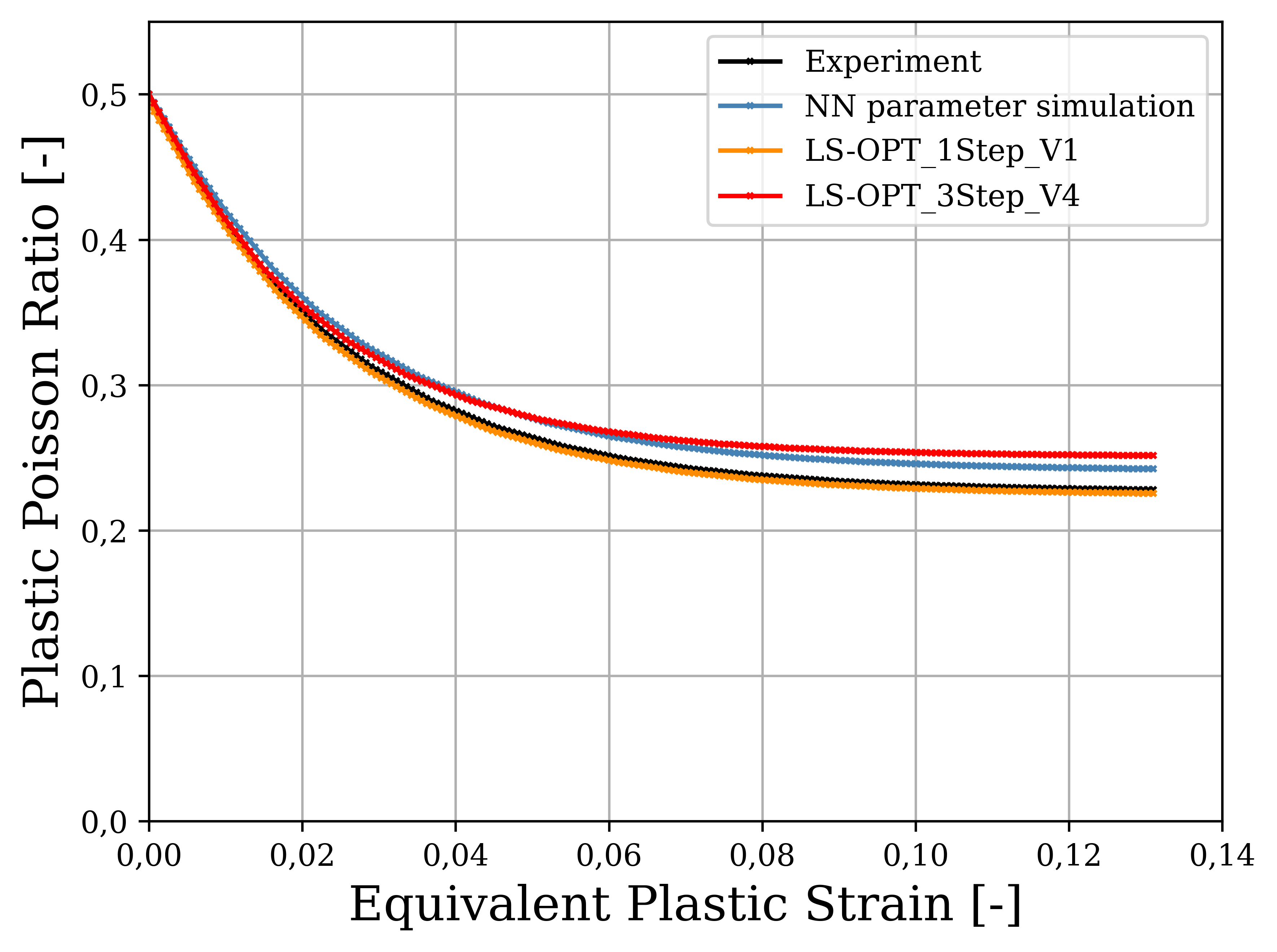

Supplement: Supplementary file 1 [file materials-15-00643-s001.zip › Supplementary_Material/SOC_NN_Pred_LSOPT_Complete/NN_Run_3/PE_Comparison_Punch_Test.png]

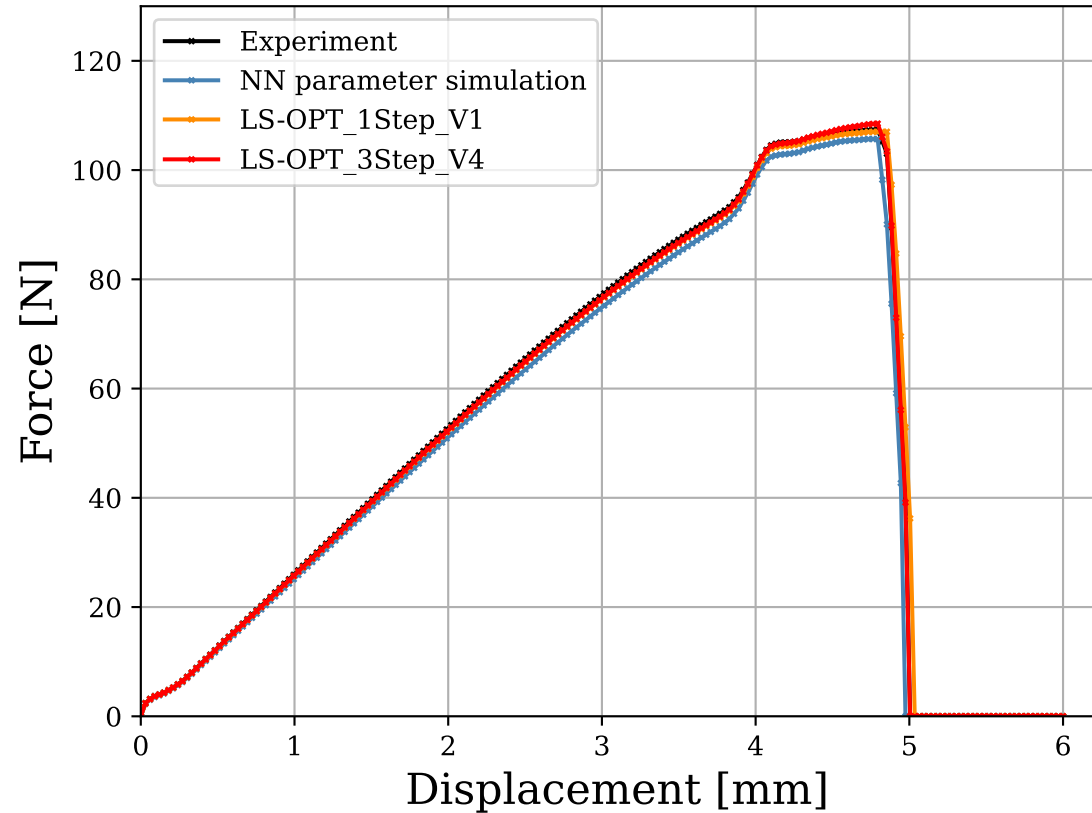

Supplement: Supplementary file 1 [file materials-15-00643-s001.zip › Supplementary_Material/SOC_NN_Pred_LSOPT_Complete/NN_Run_4/FD_Comparison_Bending_Test.pdf]

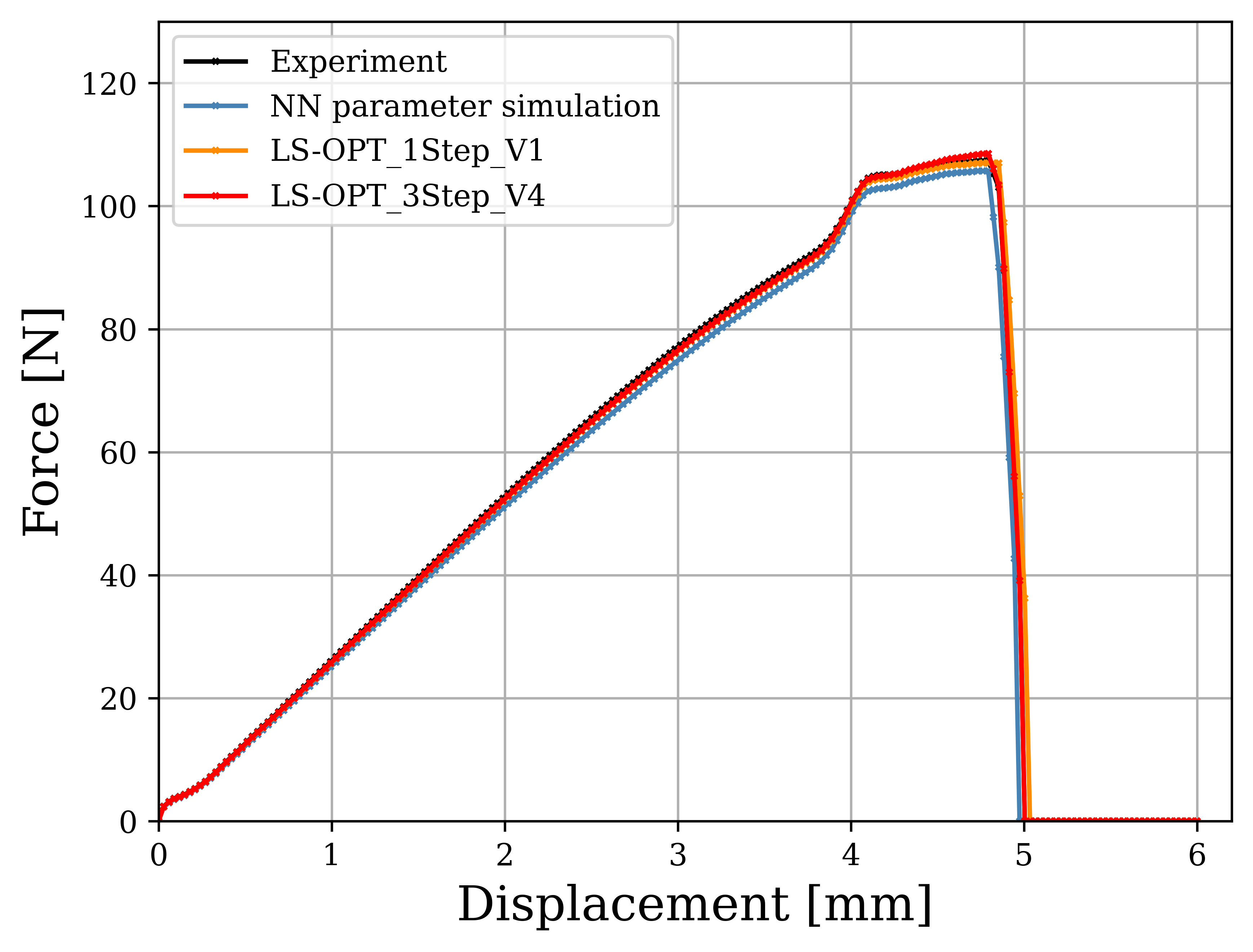

Supplement: Supplementary file 1 [file materials-15-00643-s001.zip › Supplementary_Material/SOC_NN_Pred_LSOPT_Complete/NN_Run_4/FD_Comparison_Bending_Test.png]

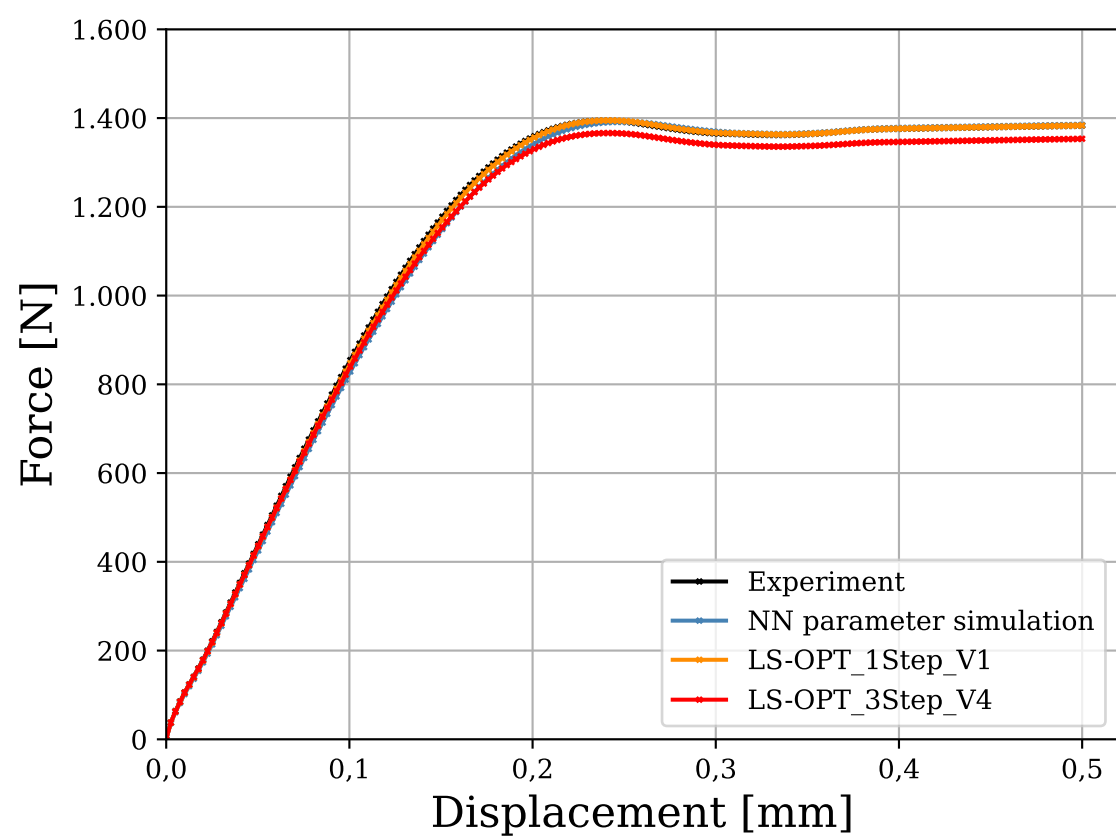

Supplement: Supplementary file 1 [file materials-15-00643-s001.zip › Supplementary_Material/SOC_NN_Pred_LSOPT_Complete/NN_Run_4/FD_Comparison_Compression_Test.pdf]

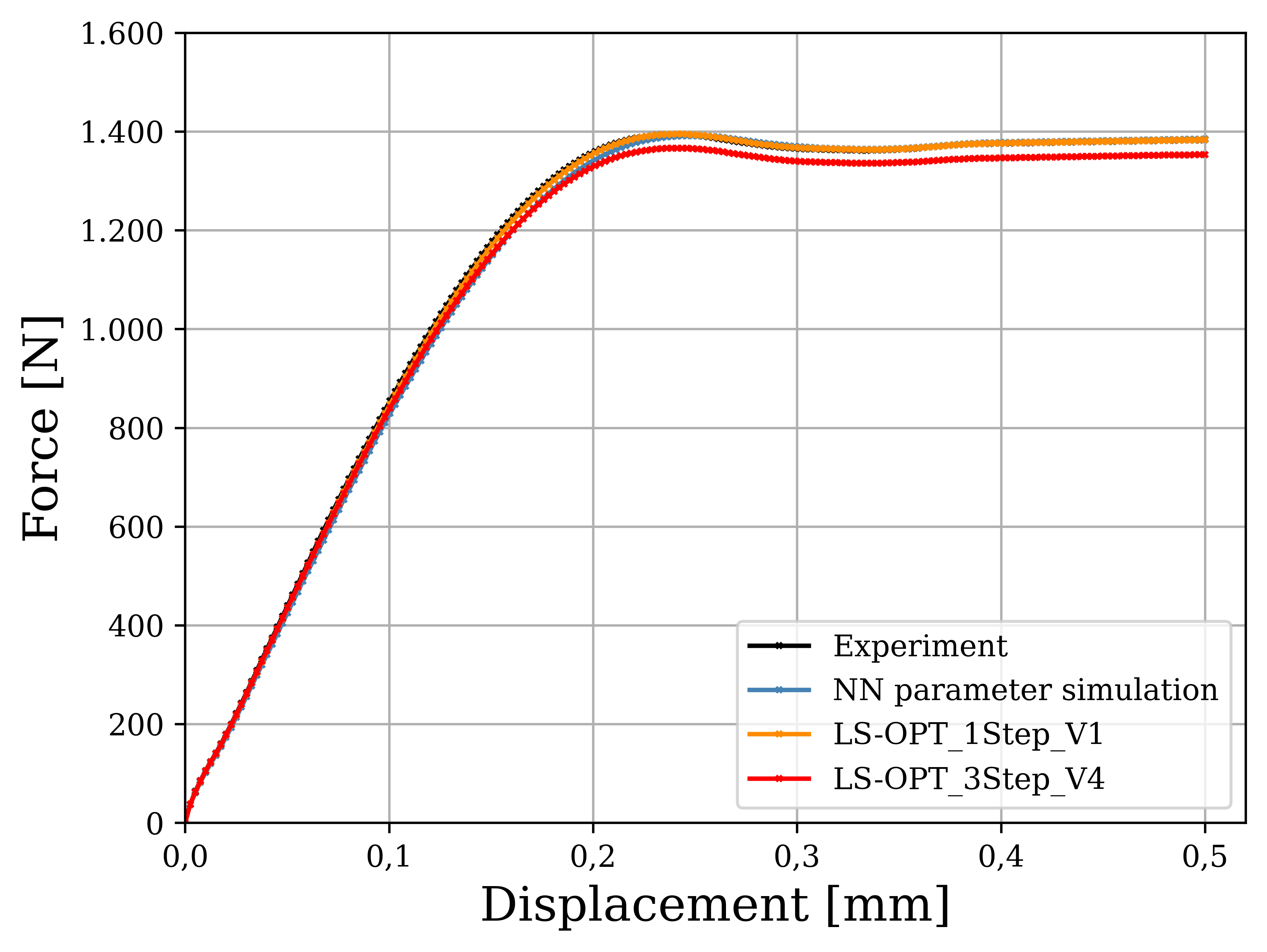

Supplement: Supplementary file 1 [file materials-15-00643-s001.zip › Supplementary_Material/SOC_NN_Pred_LSOPT_Complete/NN_Run_4/FD_Comparison_Compression_Test.png]

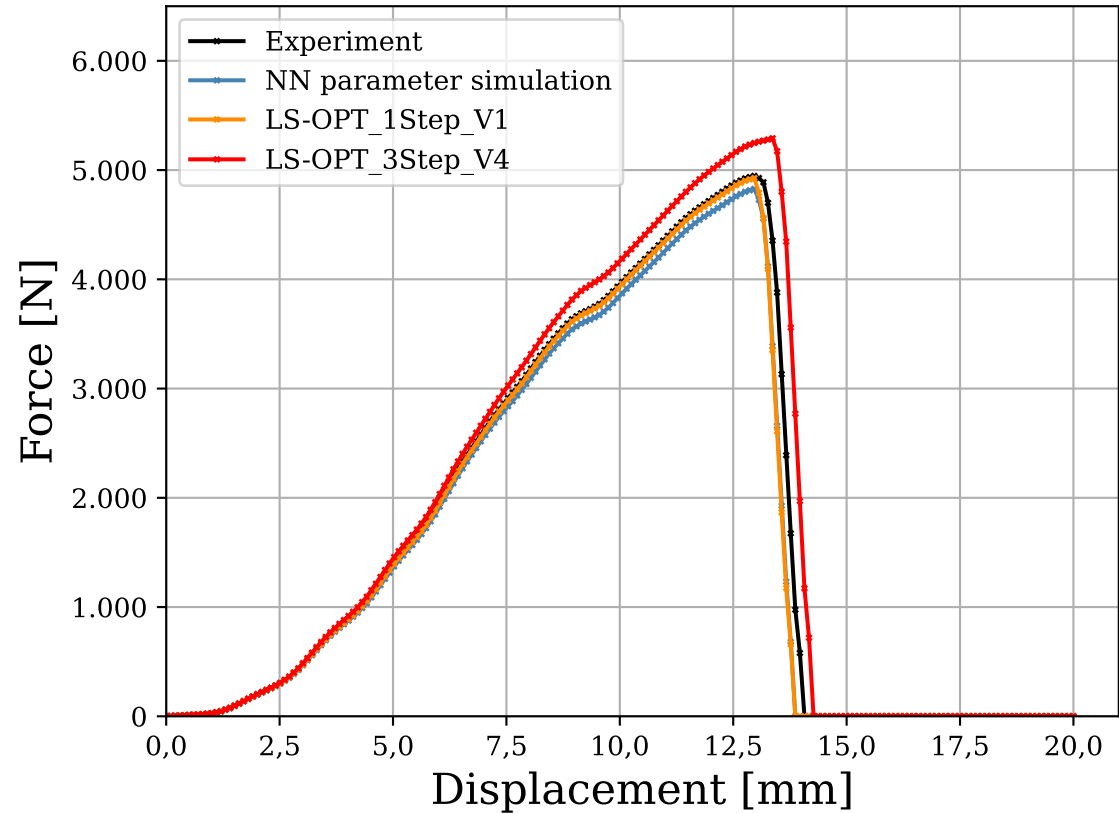

Supplement: Supplementary file 1 [file materials-15-00643-s001.zip › Supplementary_Material/SOC_NN_Pred_LSOPT_Complete/NN_Run_4/FD_Comparison_Punch_Test.pdf]

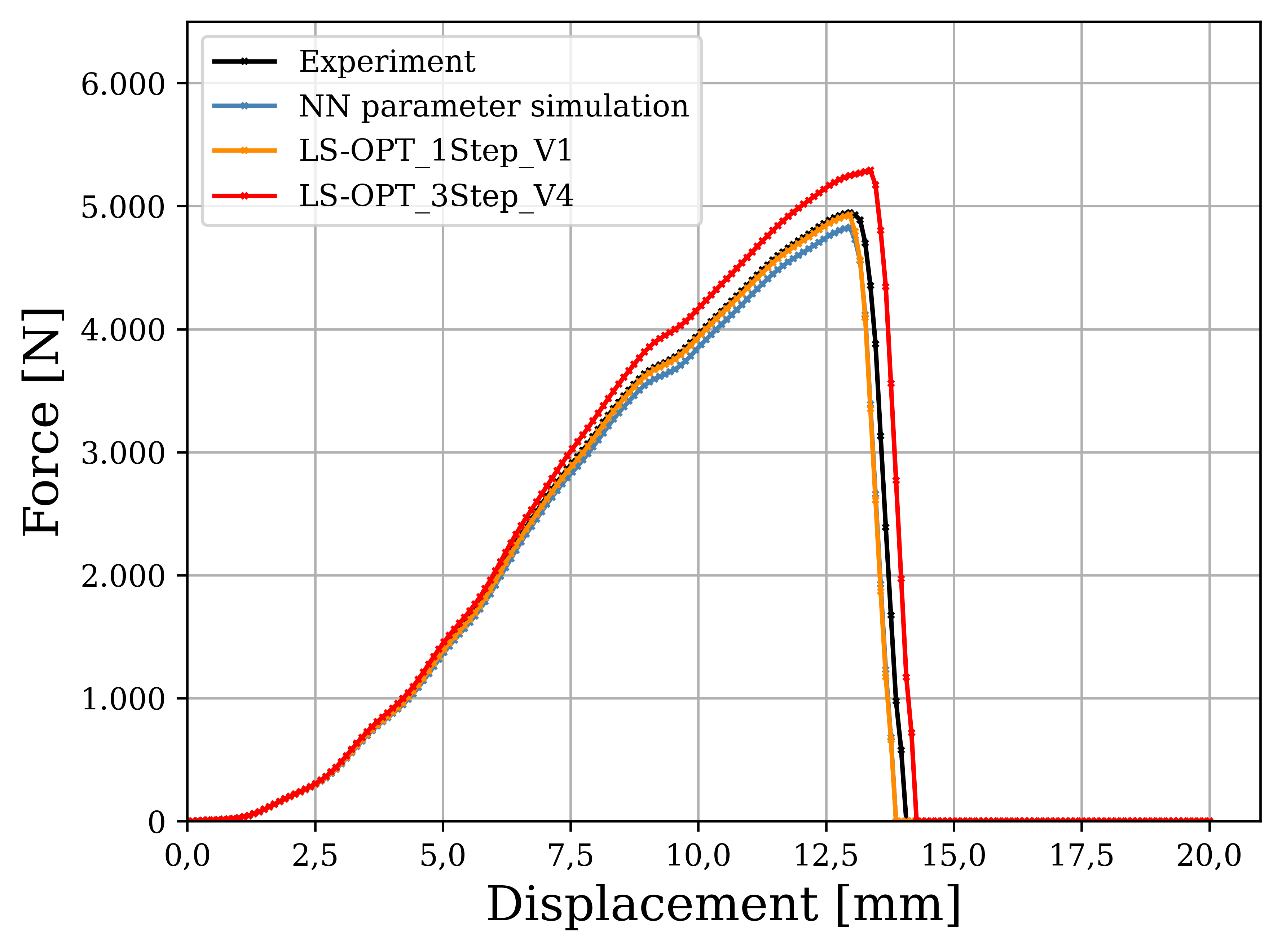

Supplement: Supplementary file 1 [file materials-15-00643-s001.zip › Supplementary_Material/SOC_NN_Pred_LSOPT_Complete/NN_Run_4/FD_Comparison_Punch_Test.png]

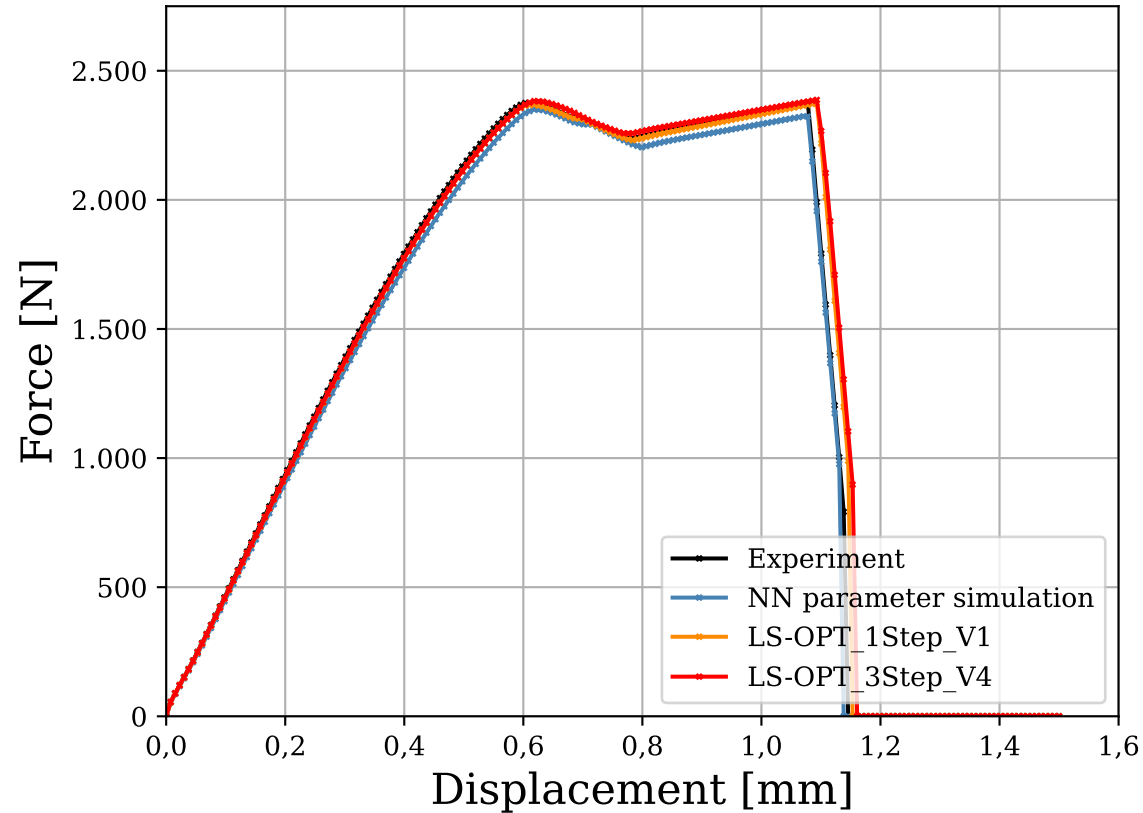

Supplement: Supplementary file 1 [file materials-15-00643-s001.zip › Supplementary_Material/SOC_NN_Pred_LSOPT_Complete/NN_Run_4/FD_Comparison_Shear_ASTM_Test.pdf]

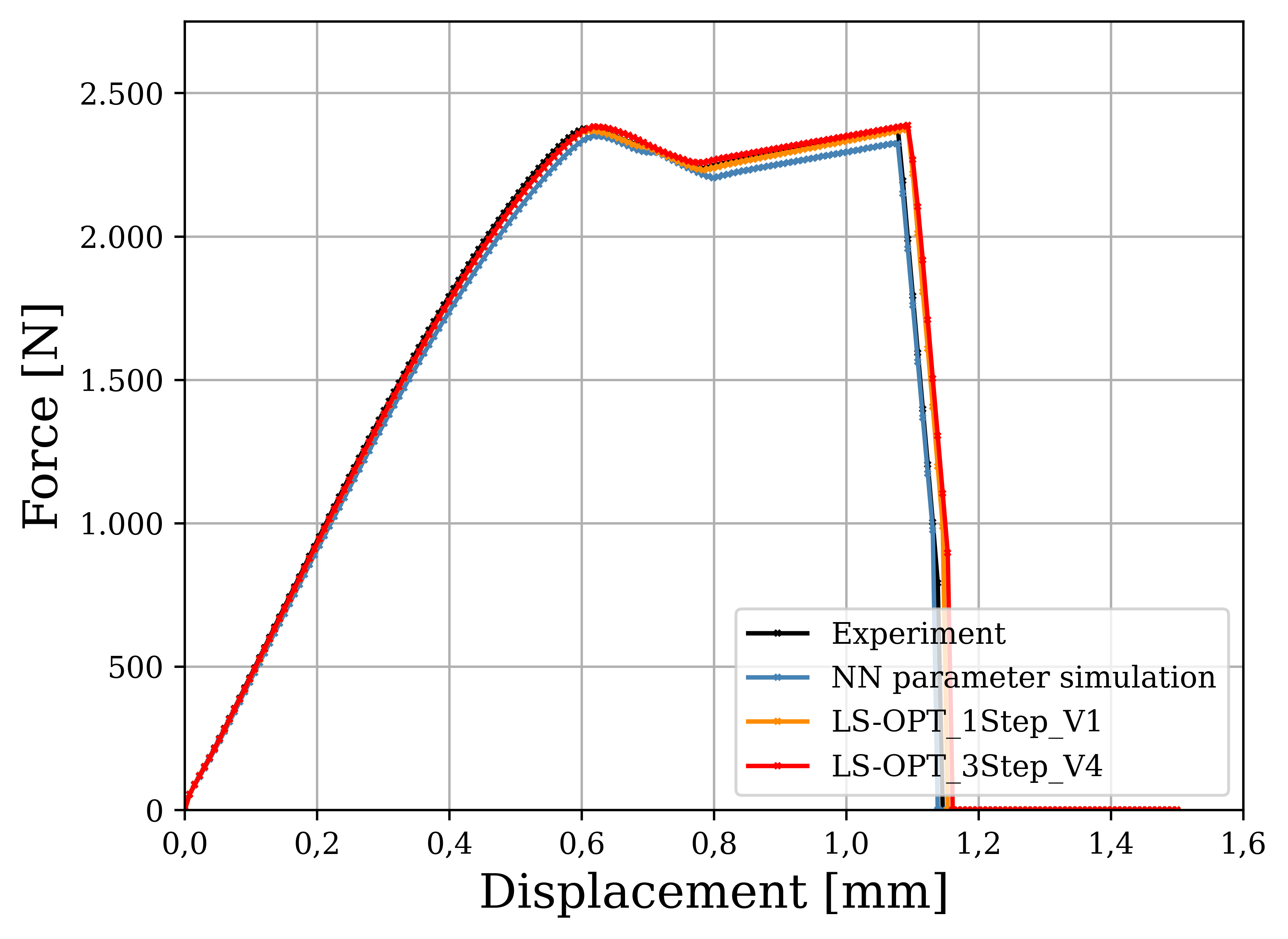

Supplement: Supplementary file 1 [file materials-15-00643-s001.zip › Supplementary_Material/SOC_NN_Pred_LSOPT_Complete/NN_Run_4/FD_Comparison_Shear_ASTM_Test.png]

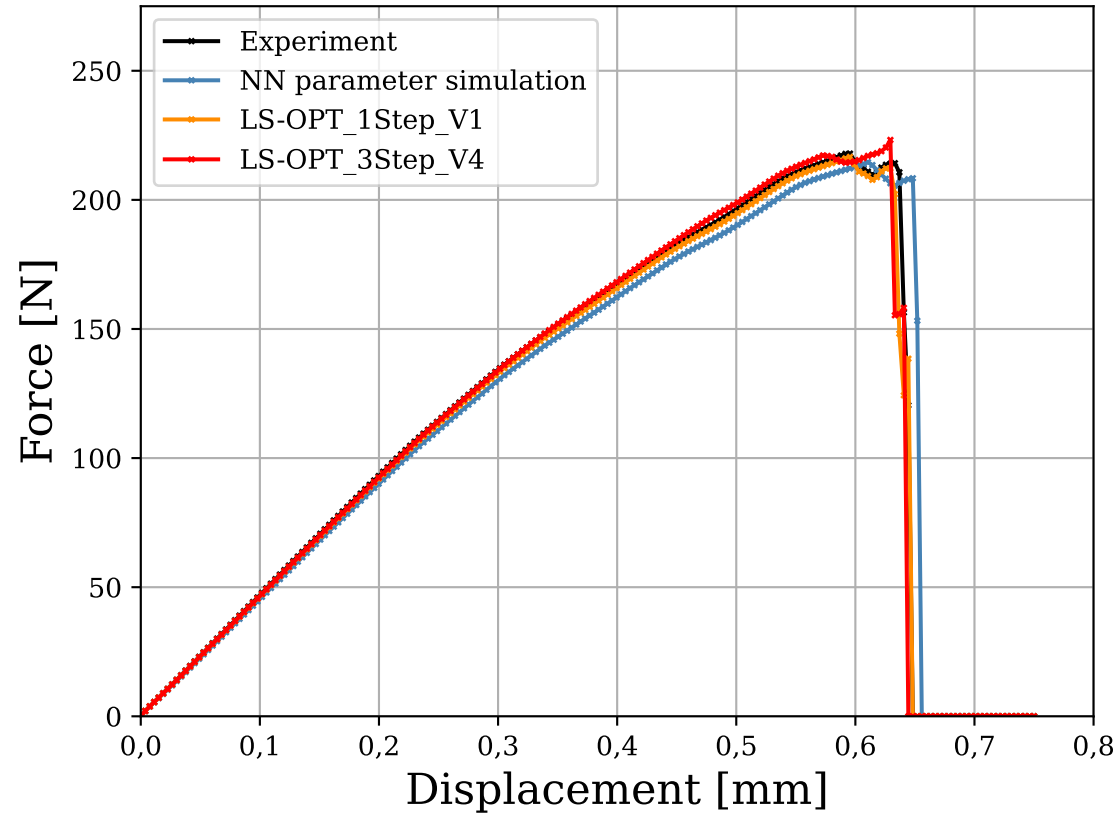

Supplement: Supplementary file 1 [file materials-15-00643-s001.zip › Supplementary_Material/SOC_NN_Pred_LSOPT_Complete/NN_Run_4/FD_Comparison_Shear_Dynamore_Test.pdf]

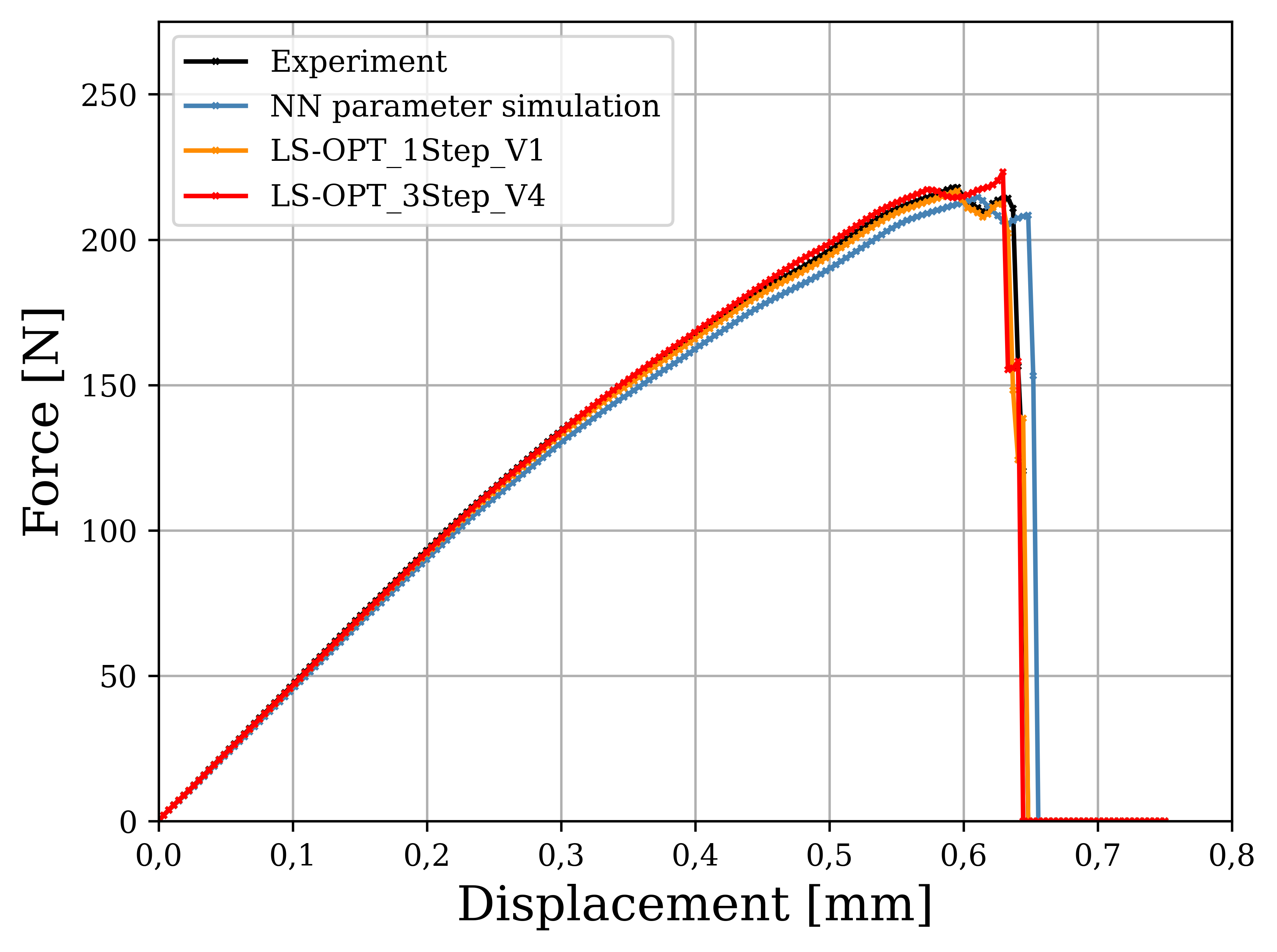

Supplement: Supplementary file 1 [file materials-15-00643-s001.zip › Supplementary_Material/SOC_NN_Pred_LSOPT_Complete/NN_Run_4/FD_Comparison_Shear_Dynamore_Test.png]

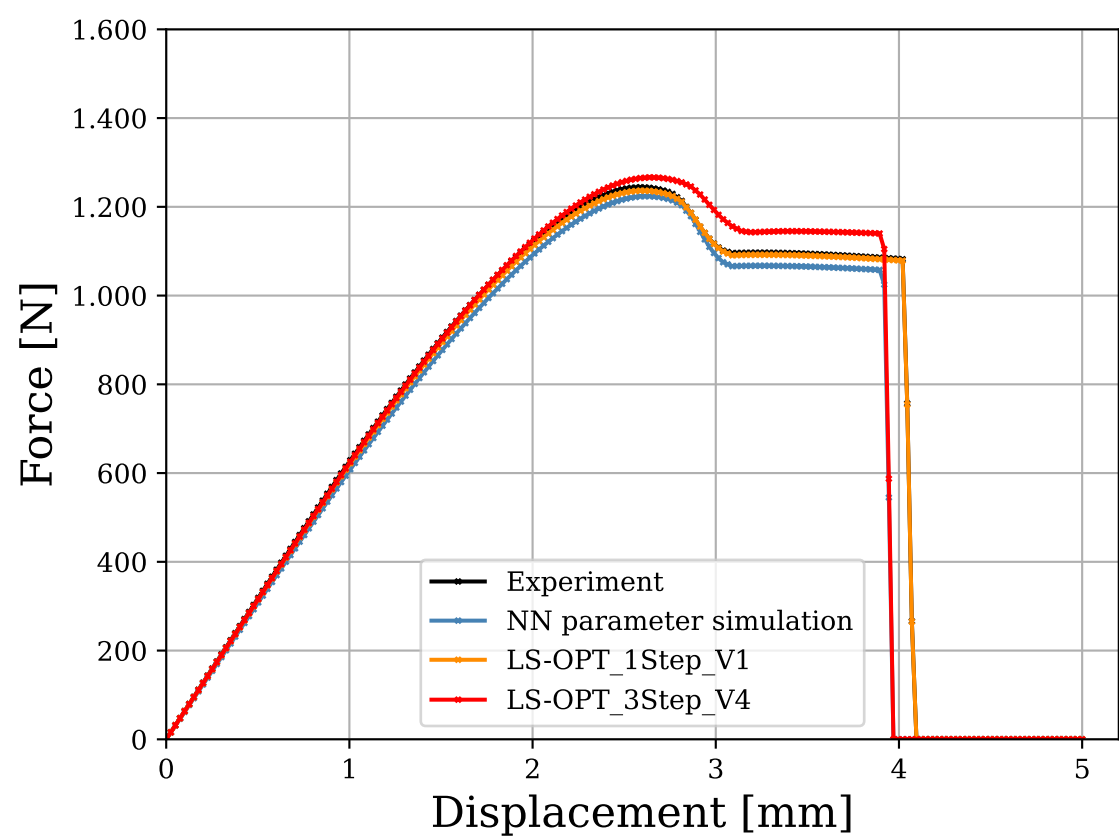

Supplement: Supplementary file 1 [file materials-15-00643-s001.zip › Supplementary_Material/SOC_NN_Pred_LSOPT_Complete/NN_Run_4/FD_Comparison_Tensile_Test.pdf]

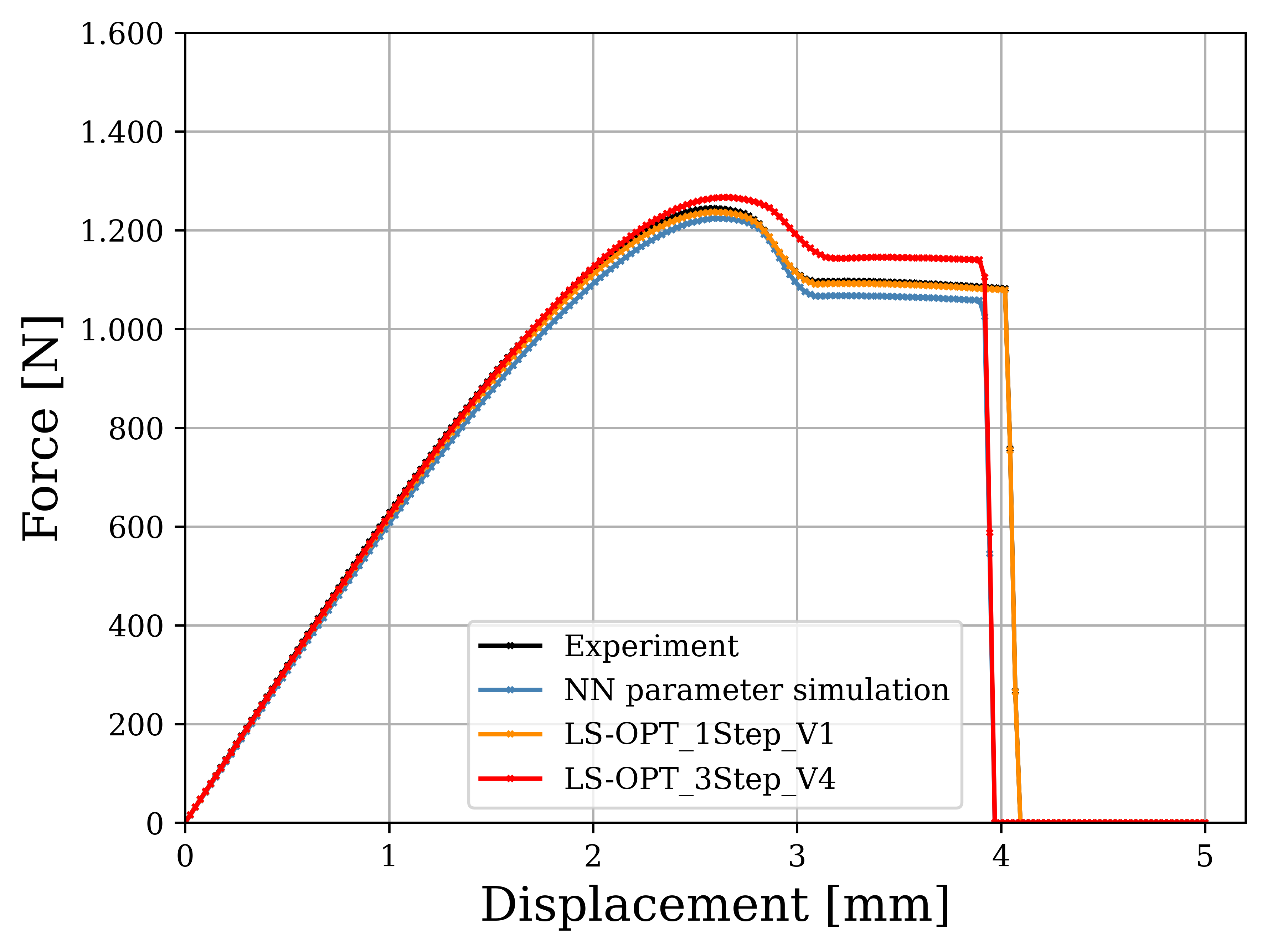

Supplement: Supplementary file 1 [file materials-15-00643-s001.zip › Supplementary_Material/SOC_NN_Pred_LSOPT_Complete/NN_Run_4/FD_Comparison_Tensile_Test.png]

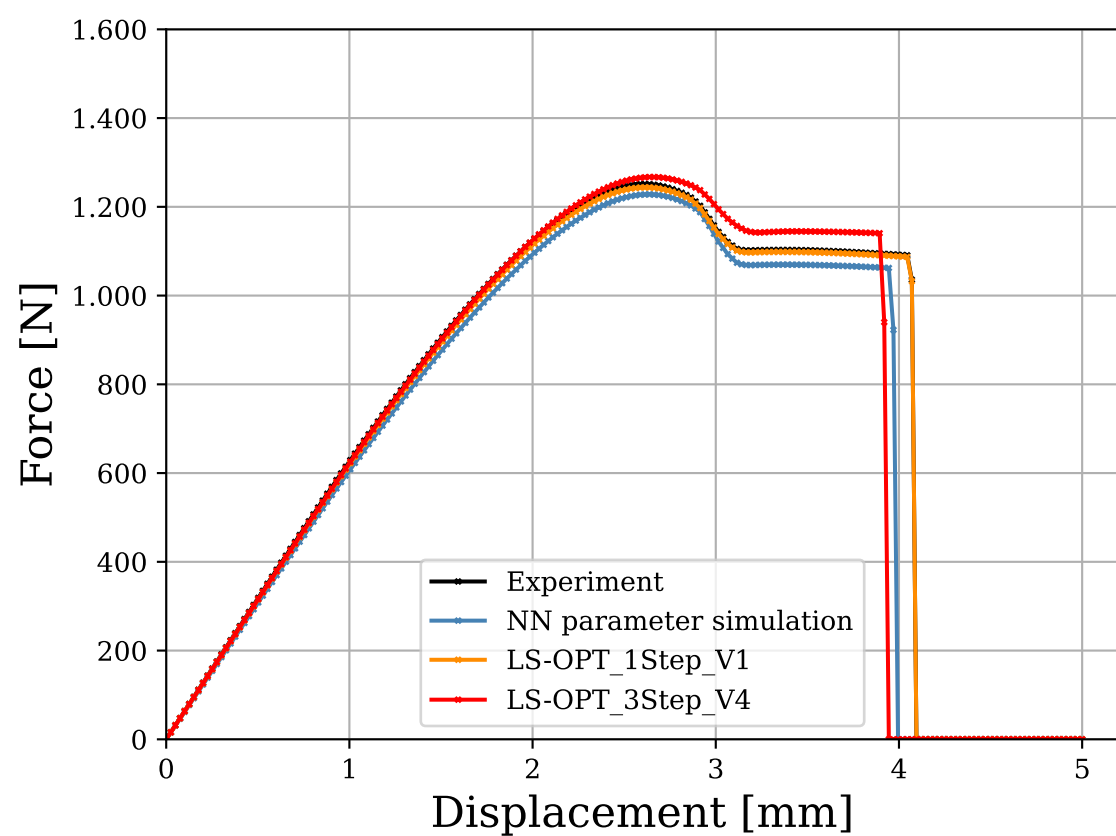

Supplement: Supplementary file 1 [file materials-15-00643-s001.zip › Supplementary_Material/SOC_NN_Pred_LSOPT_Complete/NN_Run_4/FD_Comparison_Tensile_Test_V1.pdf]

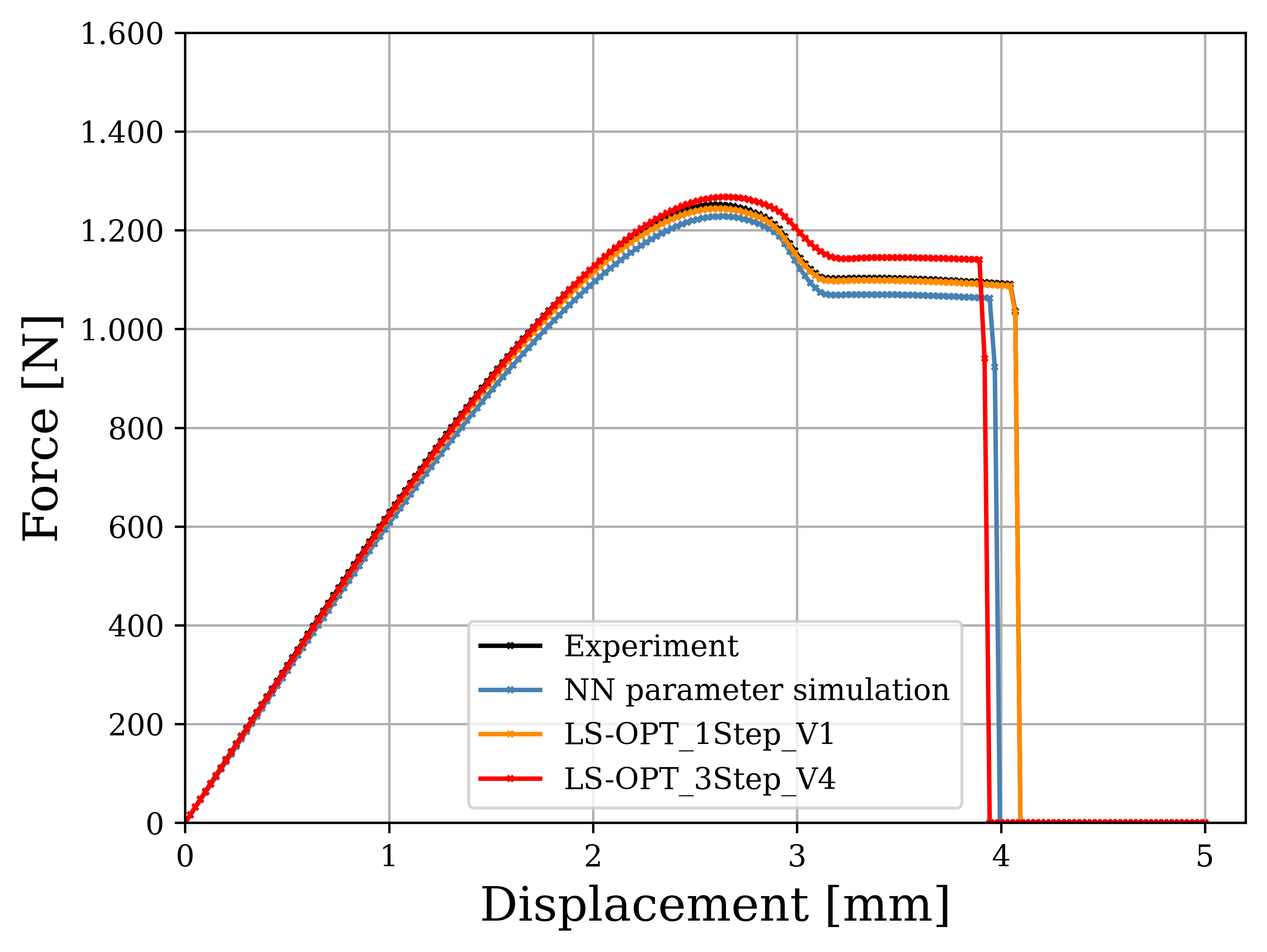

Supplement: Supplementary file 1 [file materials-15-00643-s001.zip › Supplementary_Material/SOC_NN_Pred_LSOPT_Complete/NN_Run_4/FD_Comparison_Tensile_Test_V1.png]

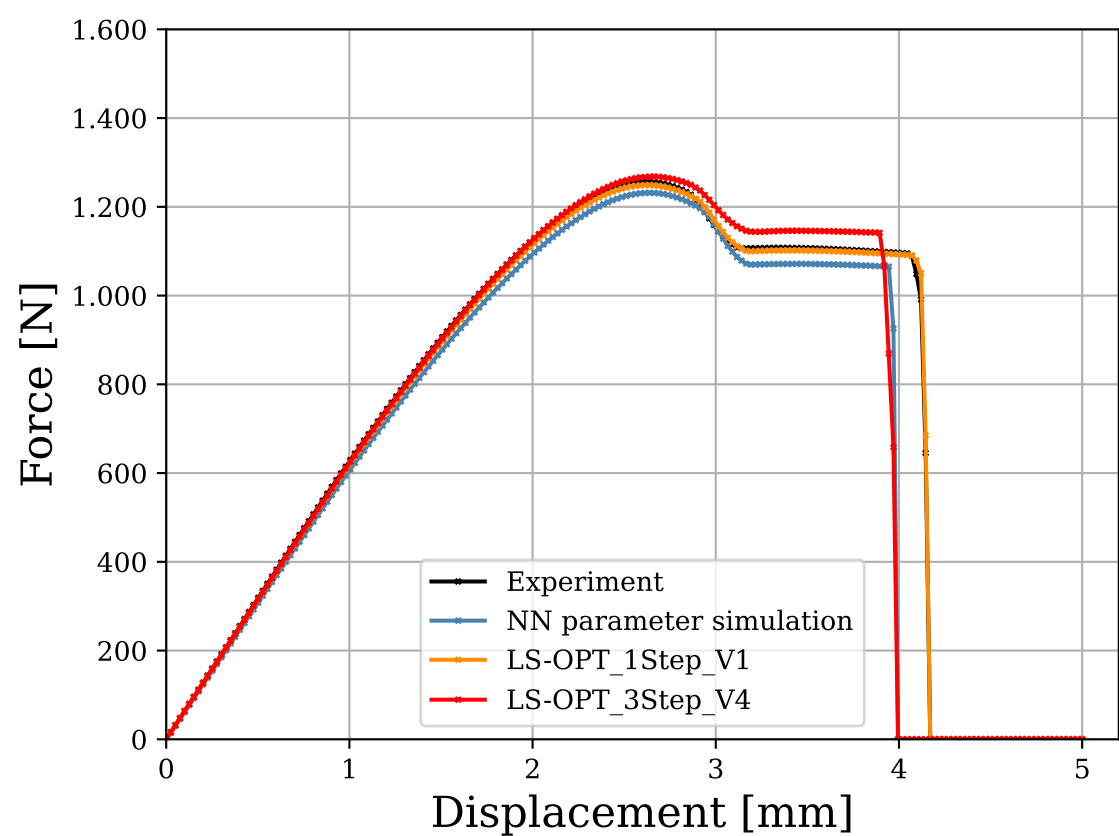

Supplement: Supplementary file 1 [file materials-15-00643-s001.zip › Supplementary_Material/SOC_NN_Pred_LSOPT_Complete/NN_Run_4/FD_Comparison_Tensile_Test_V2.pdf]

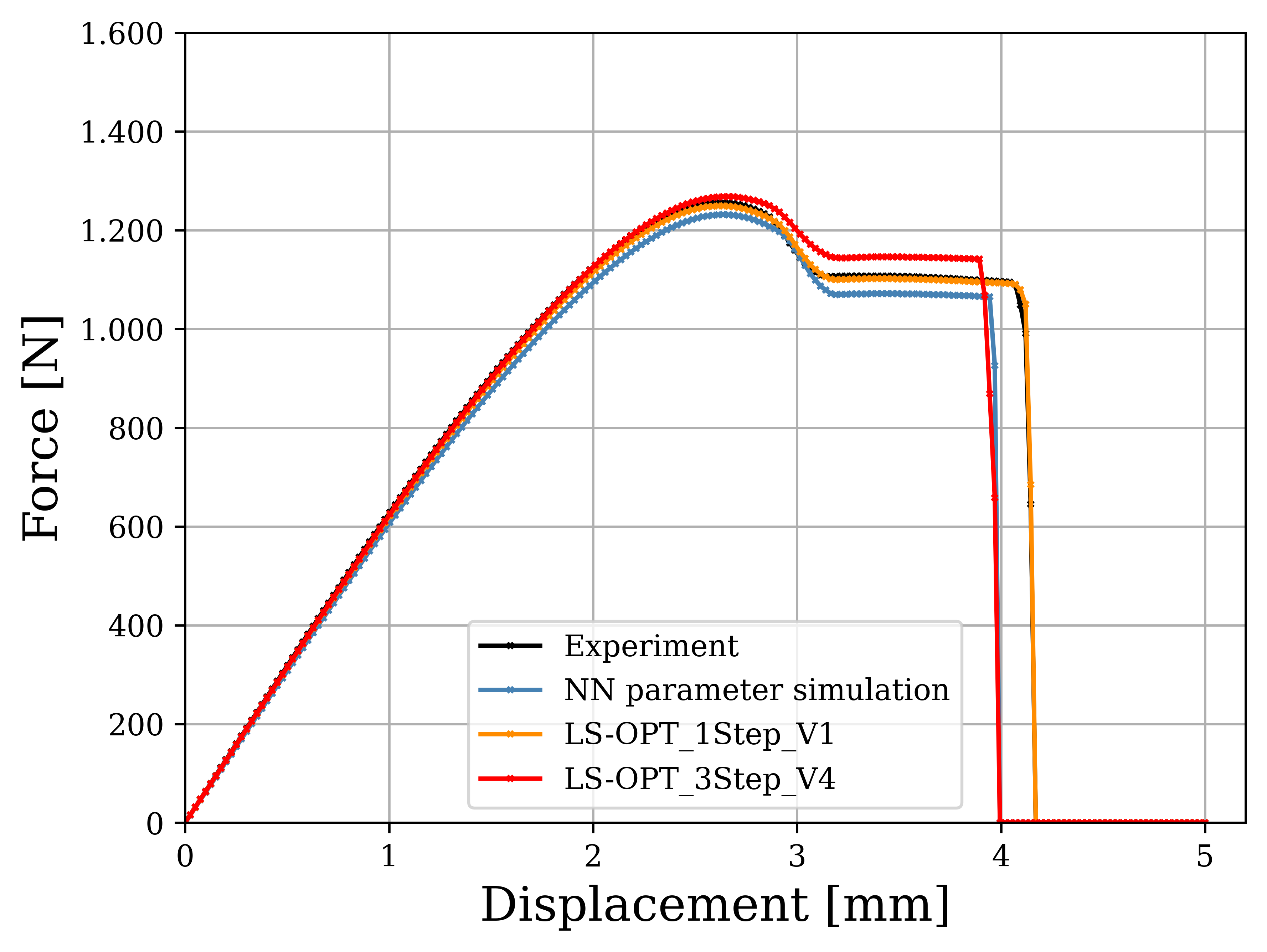

Supplement: Supplementary file 1 [file materials-15-00643-s001.zip › Supplementary_Material/SOC_NN_Pred_LSOPT_Complete/NN_Run_4/FD_Comparison_Tensile_Test_V2.png]

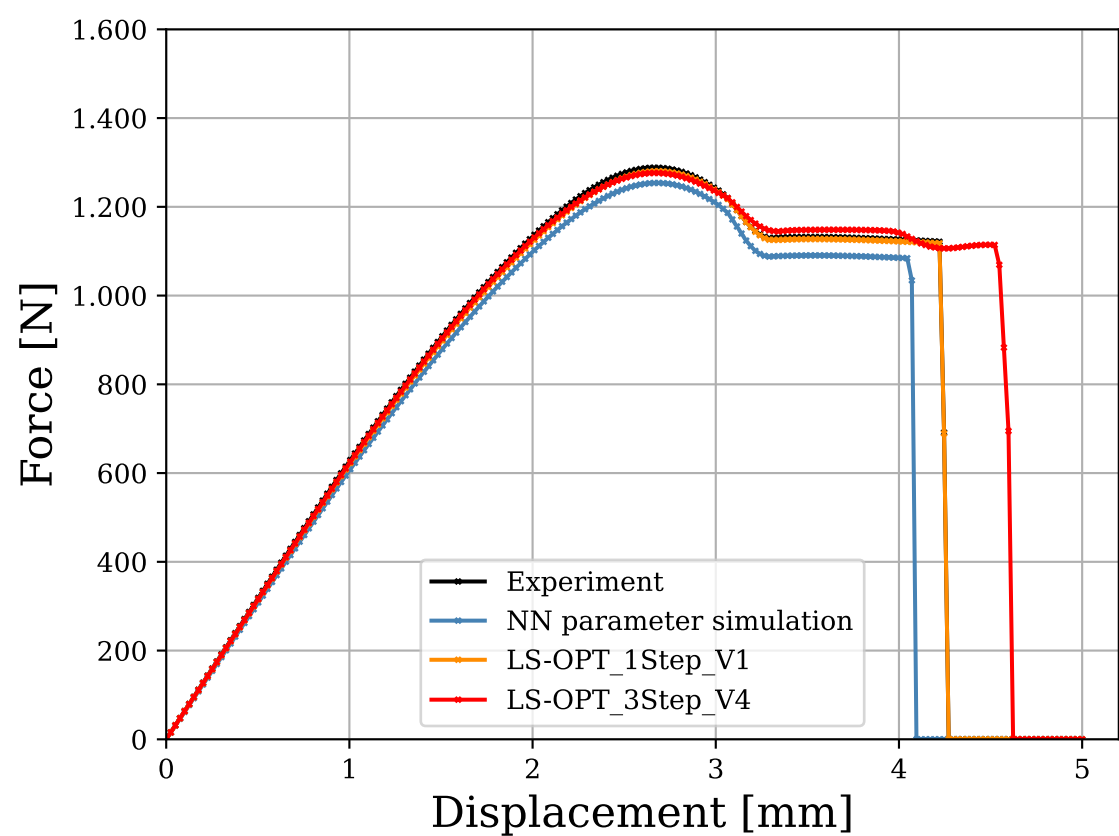

Supplement: Supplementary file 1 [file materials-15-00643-s001.zip › Supplementary_Material/SOC_NN_Pred_LSOPT_Complete/NN_Run_4/FD_Comparison_Tensile_Test_V3.pdf]

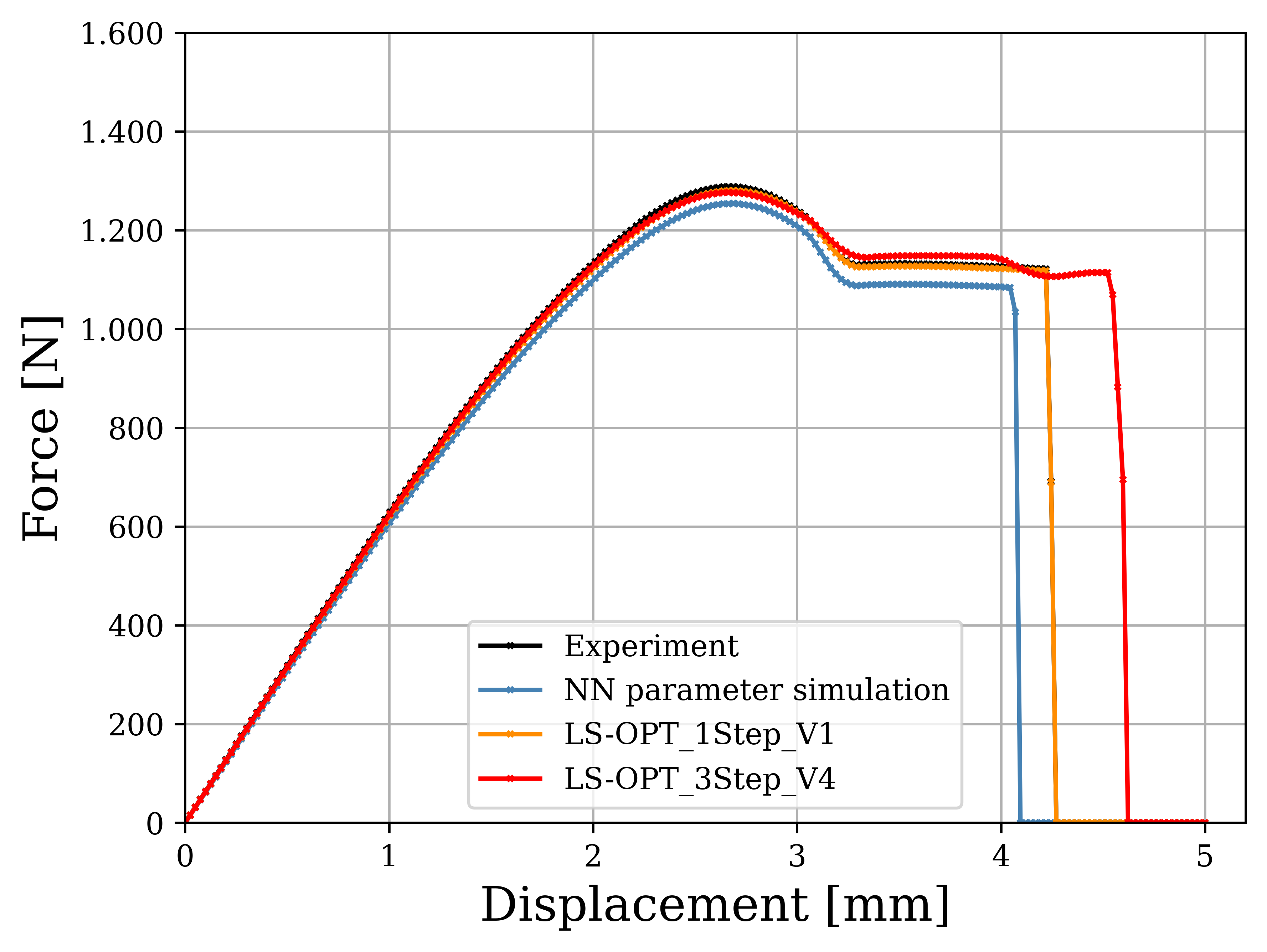

Supplement: Supplementary file 1 [file materials-15-00643-s001.zip › Supplementary_Material/SOC_NN_Pred_LSOPT_Complete/NN_Run_4/FD_Comparison_Tensile_Test_V3.png]

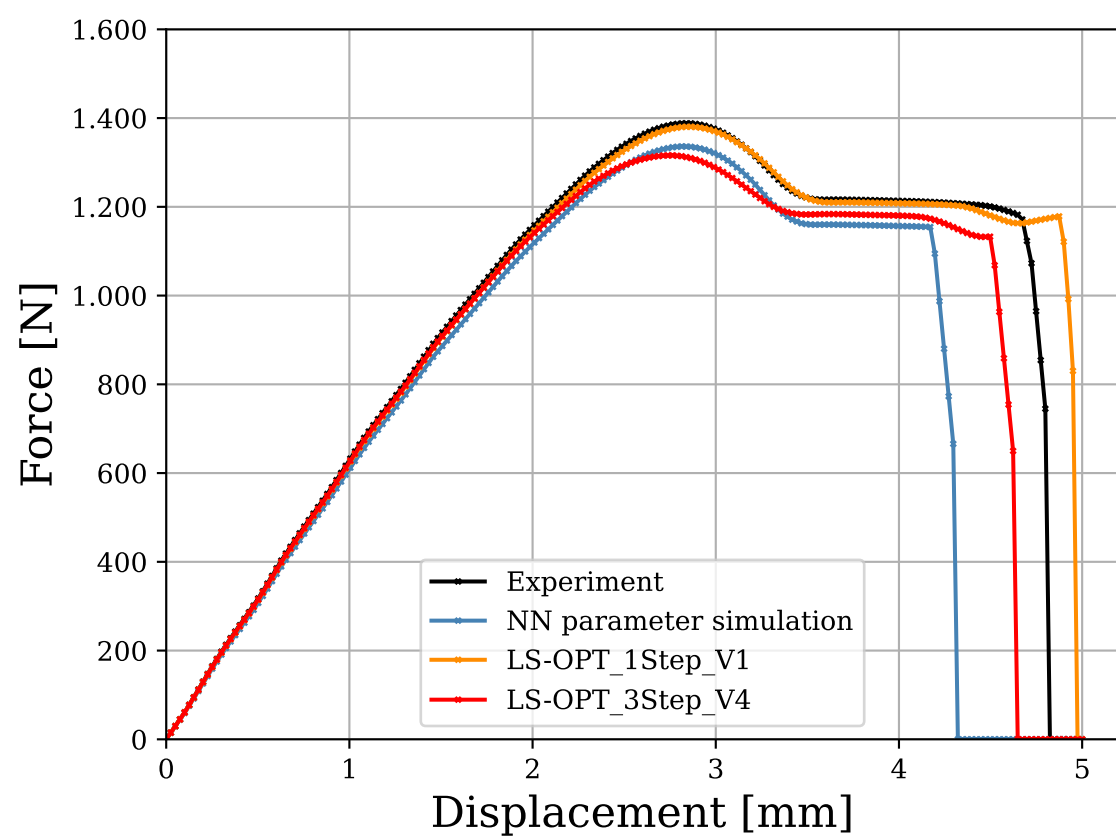

Supplement: Supplementary file 1 [file materials-15-00643-s001.zip › Supplementary_Material/SOC_NN_Pred_LSOPT_Complete/NN_Run_4/FD_Comparison_Tensile_Test_V4.pdf]

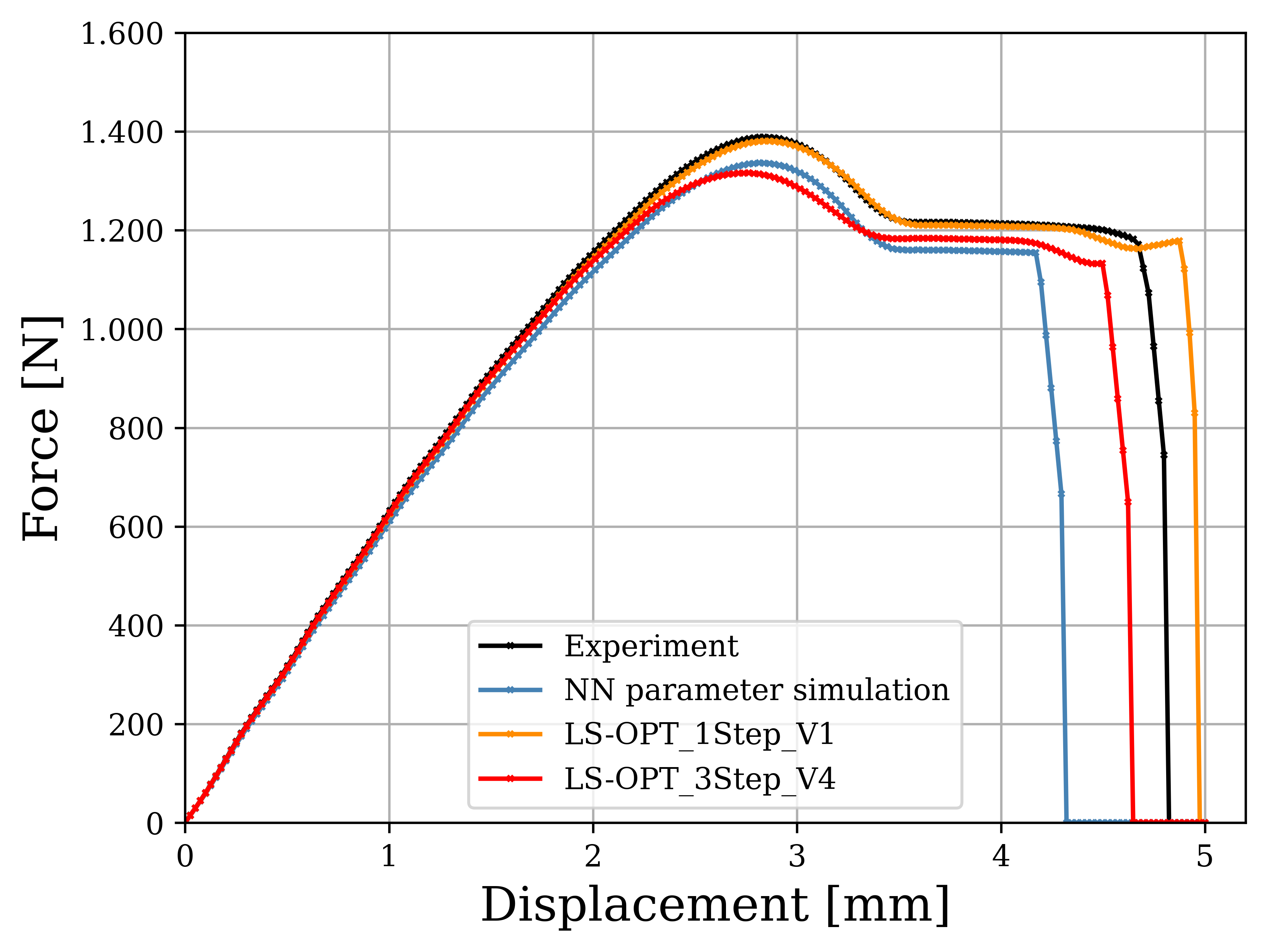

Supplement: Supplementary file 1 [file materials-15-00643-s001.zip › Supplementary_Material/SOC_NN_Pred_LSOPT_Complete/NN_Run_4/FD_Comparison_Tensile_Test_V4.png]

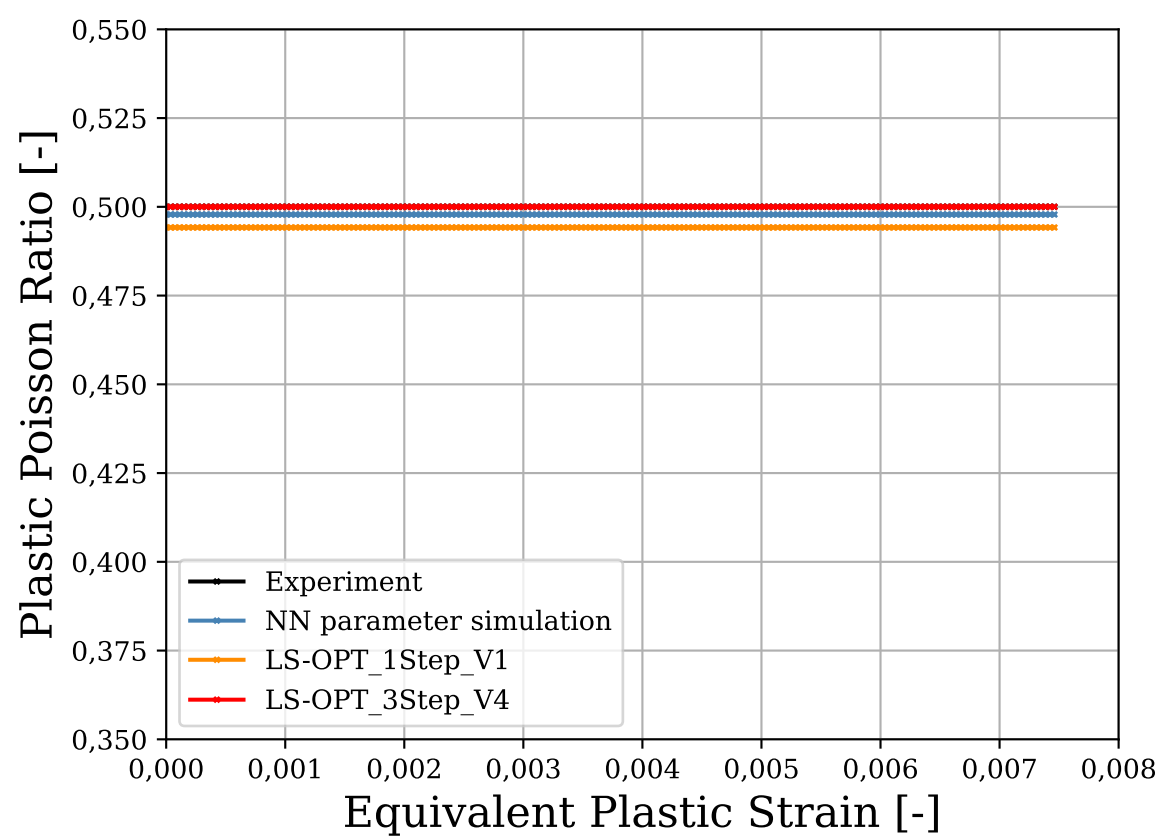

Supplement: Supplementary file 1 [file materials-15-00643-s001.zip › Supplementary_Material/SOC_NN_Pred_LSOPT_Complete/NN_Run_4/PE_Comparison_Compression_Test.pdf]

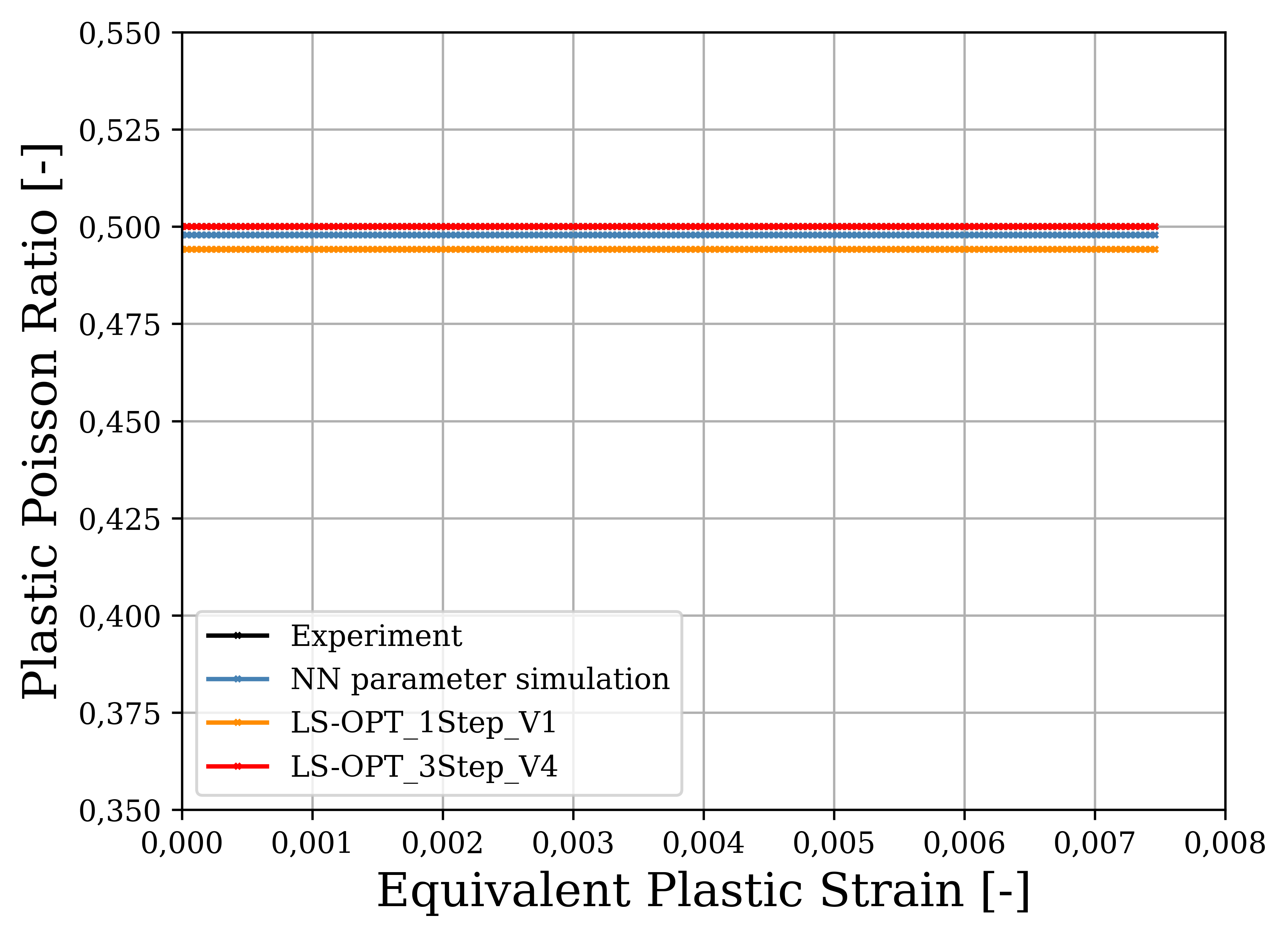

Supplement: Supplementary file 1 [file materials-15-00643-s001.zip › Supplementary_Material/SOC_NN_Pred_LSOPT_Complete/NN_Run_4/PE_Comparison_Compression_Test.png]

Plastic Poisson Ratio [-]

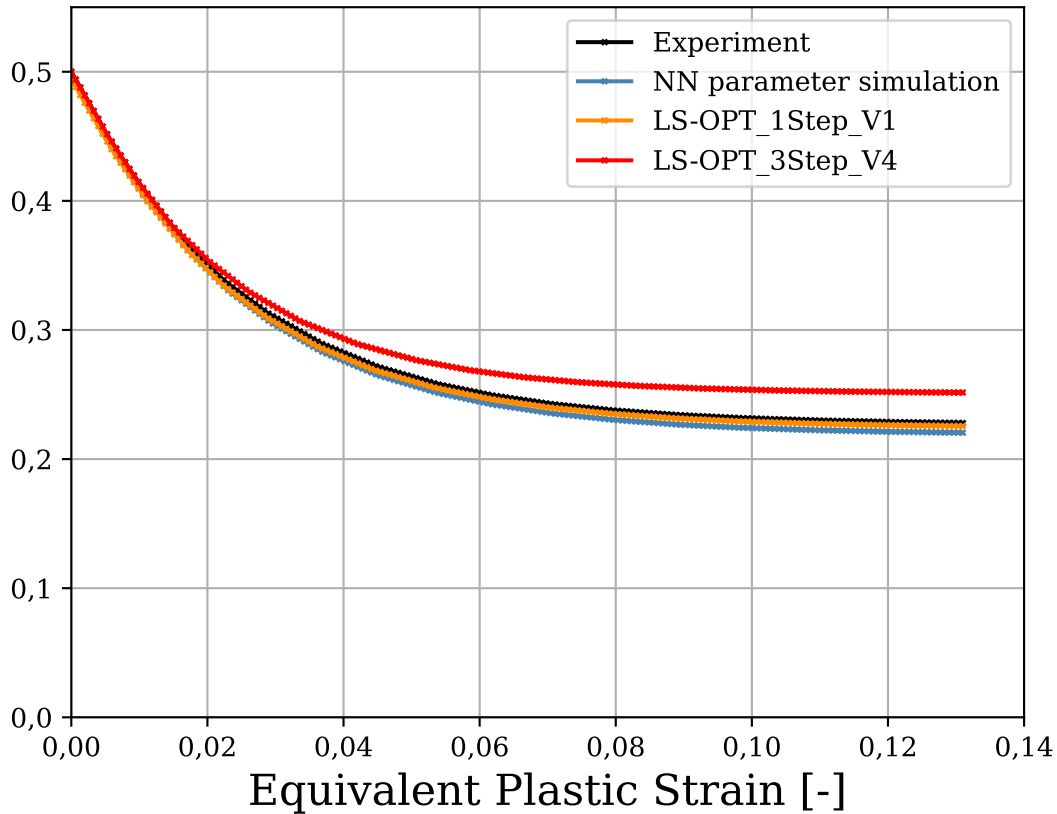

Supplement: Supplementary file 1 [file materials-15-00643-s001.zip › Supplementary_Material/SOC_NN_Pred_LSOPT_Complete/NN_Run_4/PE_Comparison_Punch_Test.pdf]

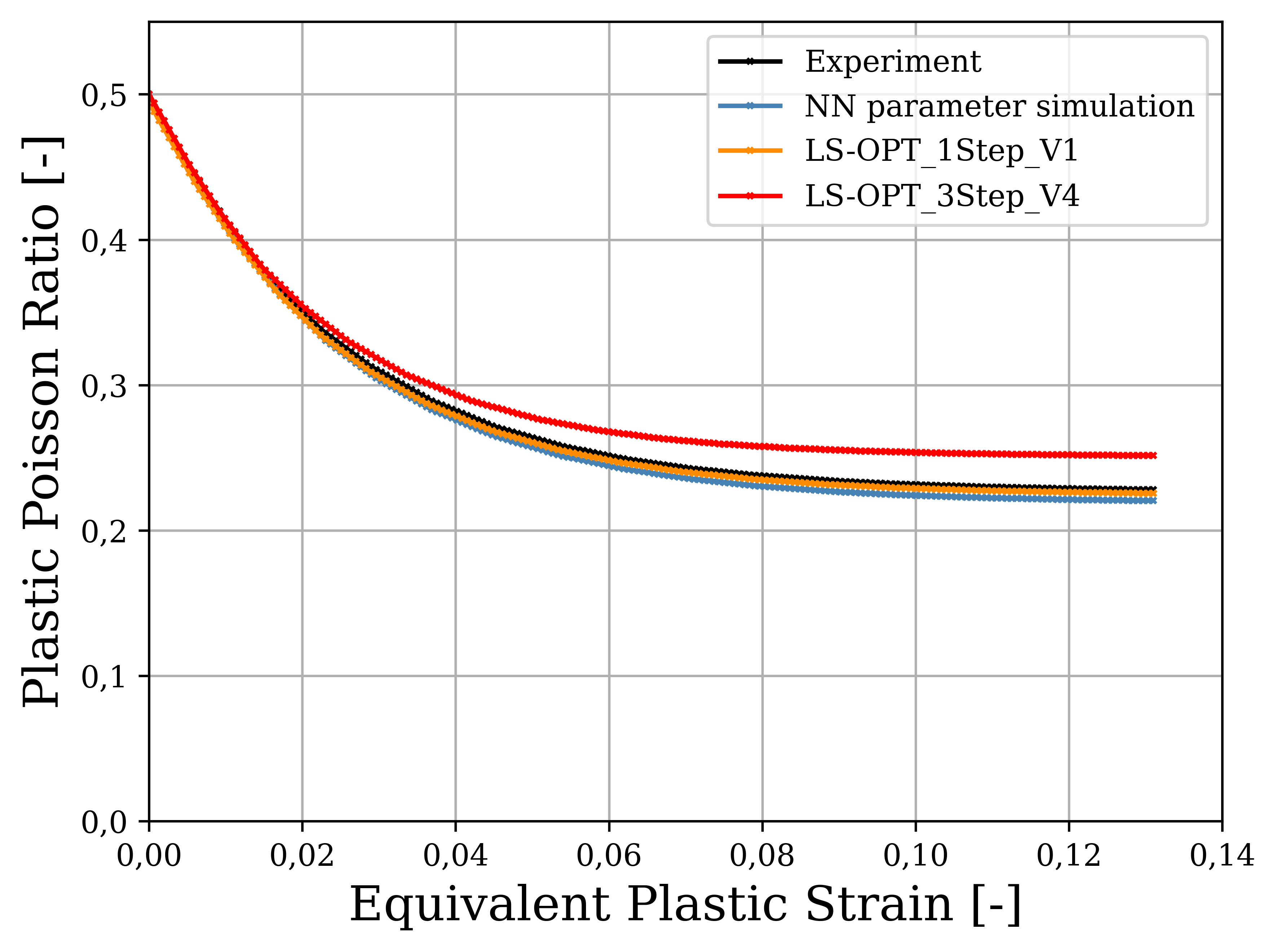

Supplement: Supplementary file 1 [file materials-15-00643-s001.zip › Supplementary_Material/SOC_NN_Pred_LSOPT_Complete/NN_Run_4/PE_Comparison_Punch_Test.png]

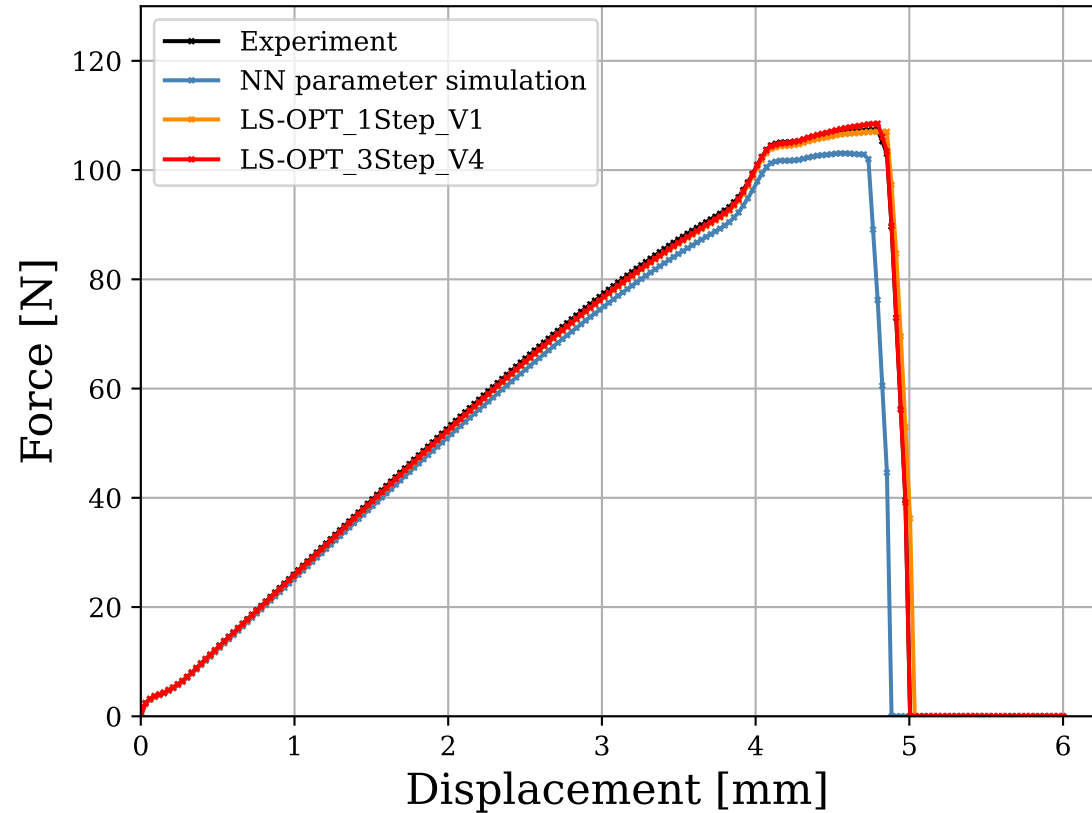

Supplement: Supplementary file 1 [file materials-15-00643-s001.zip › Supplementary_Material/SOC_NN_Pred_LSOPT_Complete/NN_Run_5/FD_Comparison_Bending_Test.pdf]

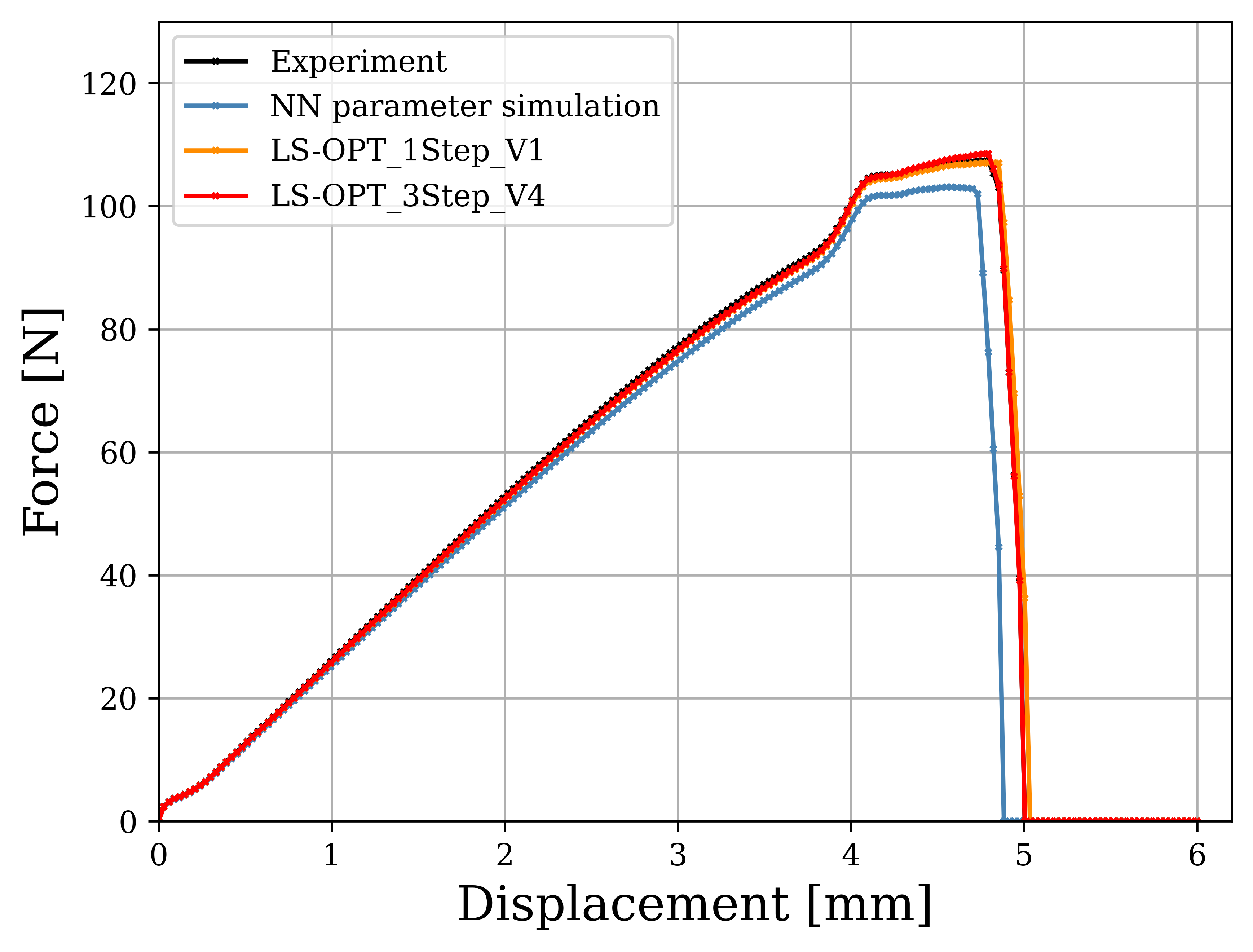

Supplement: Supplementary file 1 [file materials-15-00643-s001.zip › Supplementary_Material/SOC_NN_Pred_LSOPT_Complete/NN_Run_5/FD_Comparison_Bending_Test.png]

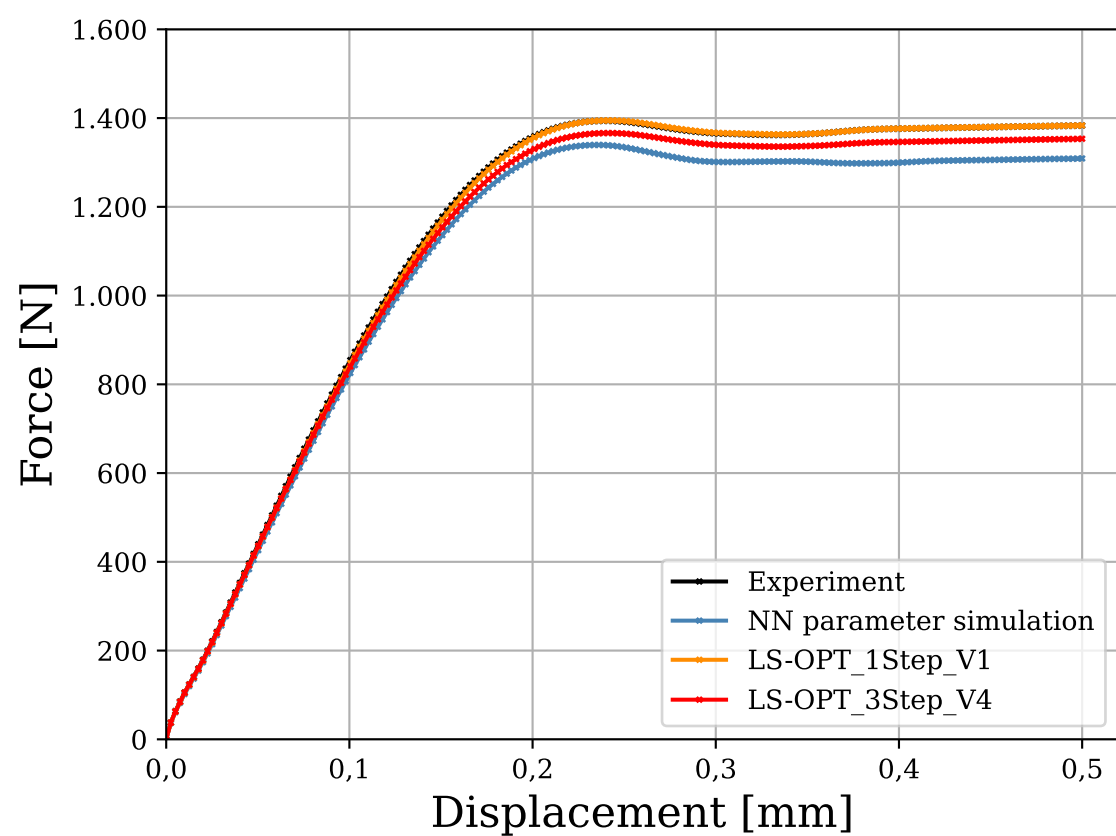

Supplement: Supplementary file 1 [file materials-15-00643-s001.zip › Supplementary_Material/SOC_NN_Pred_LSOPT_Complete/NN_Run_5/FD_Comparison_Compression_Test.pdf]

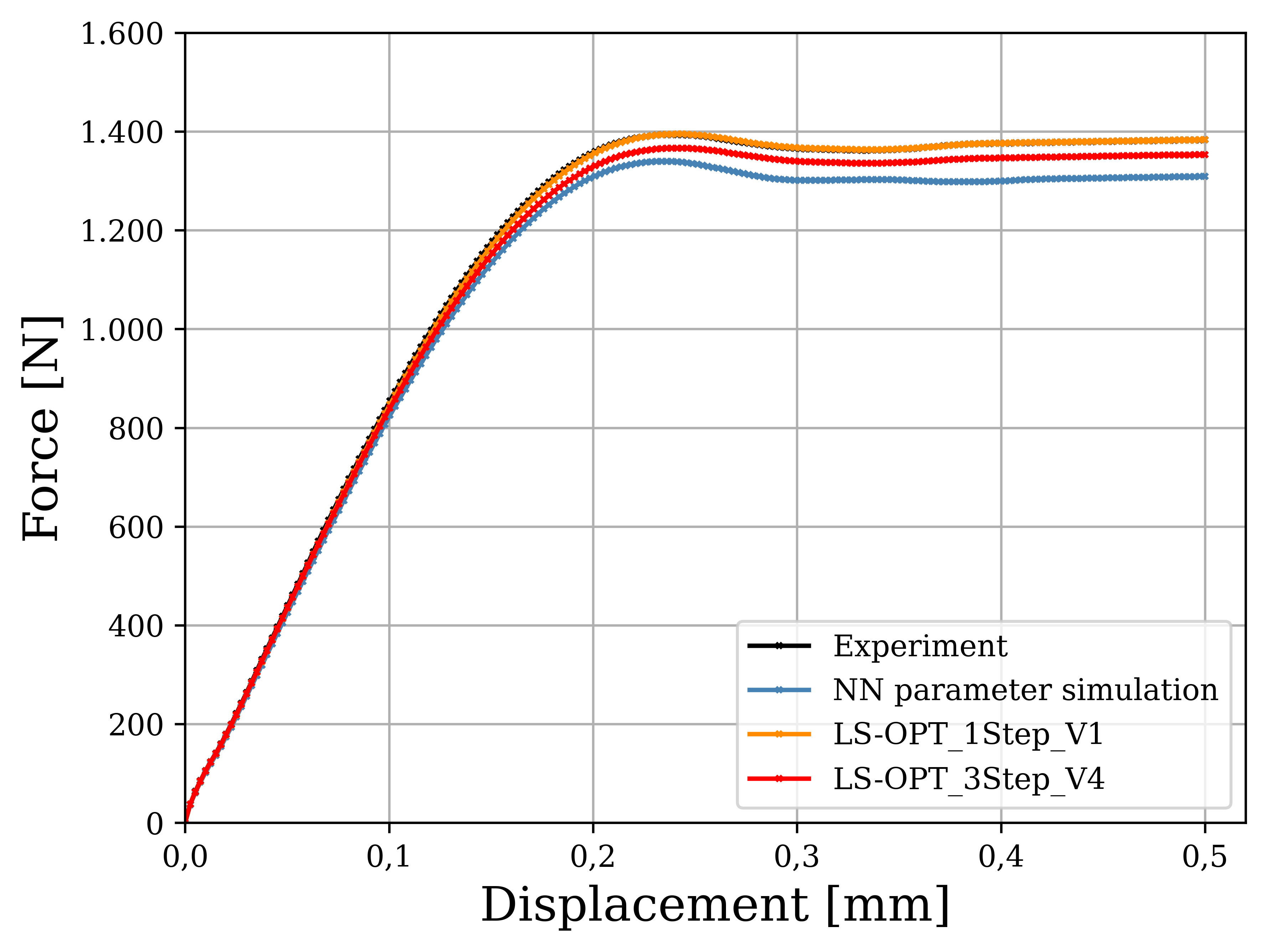

Supplement: Supplementary file 1 [file materials-15-00643-s001.zip › Supplementary_Material/SOC_NN_Pred_LSOPT_Complete/NN_Run_5/FD_Comparison_Compression_Test.png]

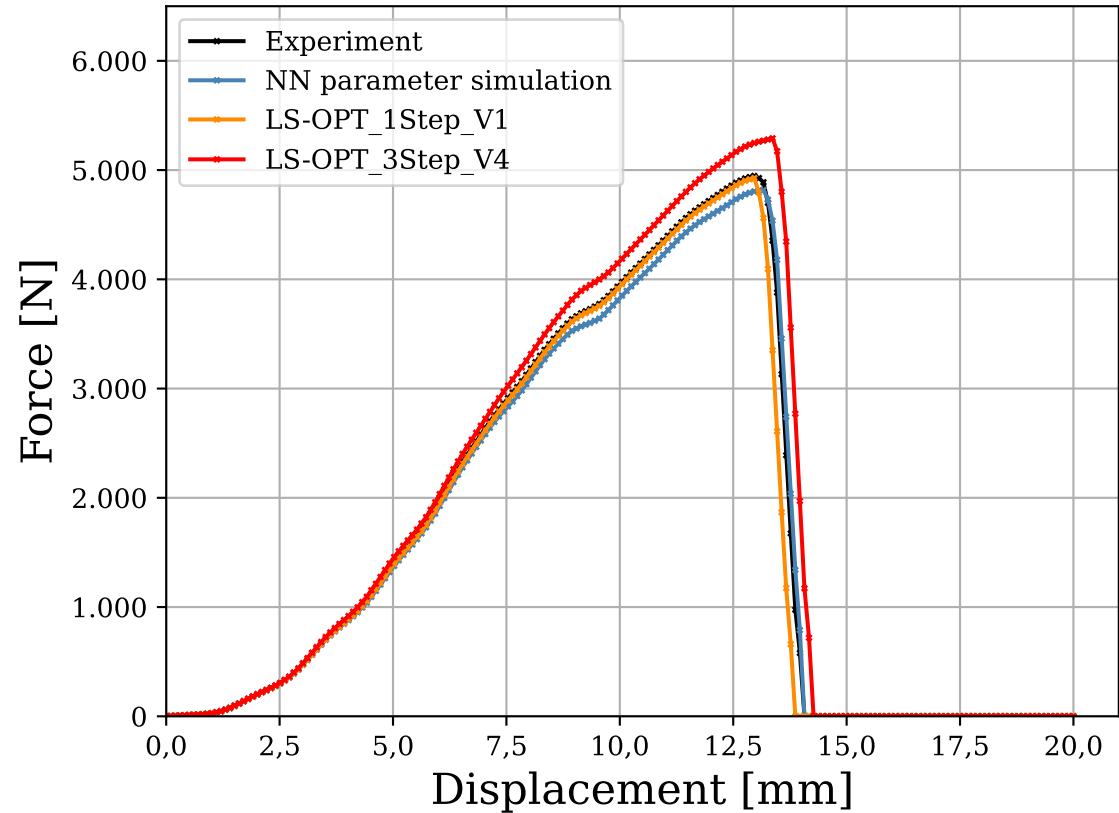

Supplement: Supplementary file 1 [file materials-15-00643-s001.zip › Supplementary_Material/SOC_NN_Pred_LSOPT_Complete/NN_Run_5/FD_Comparison_Punch_Test.pdf]

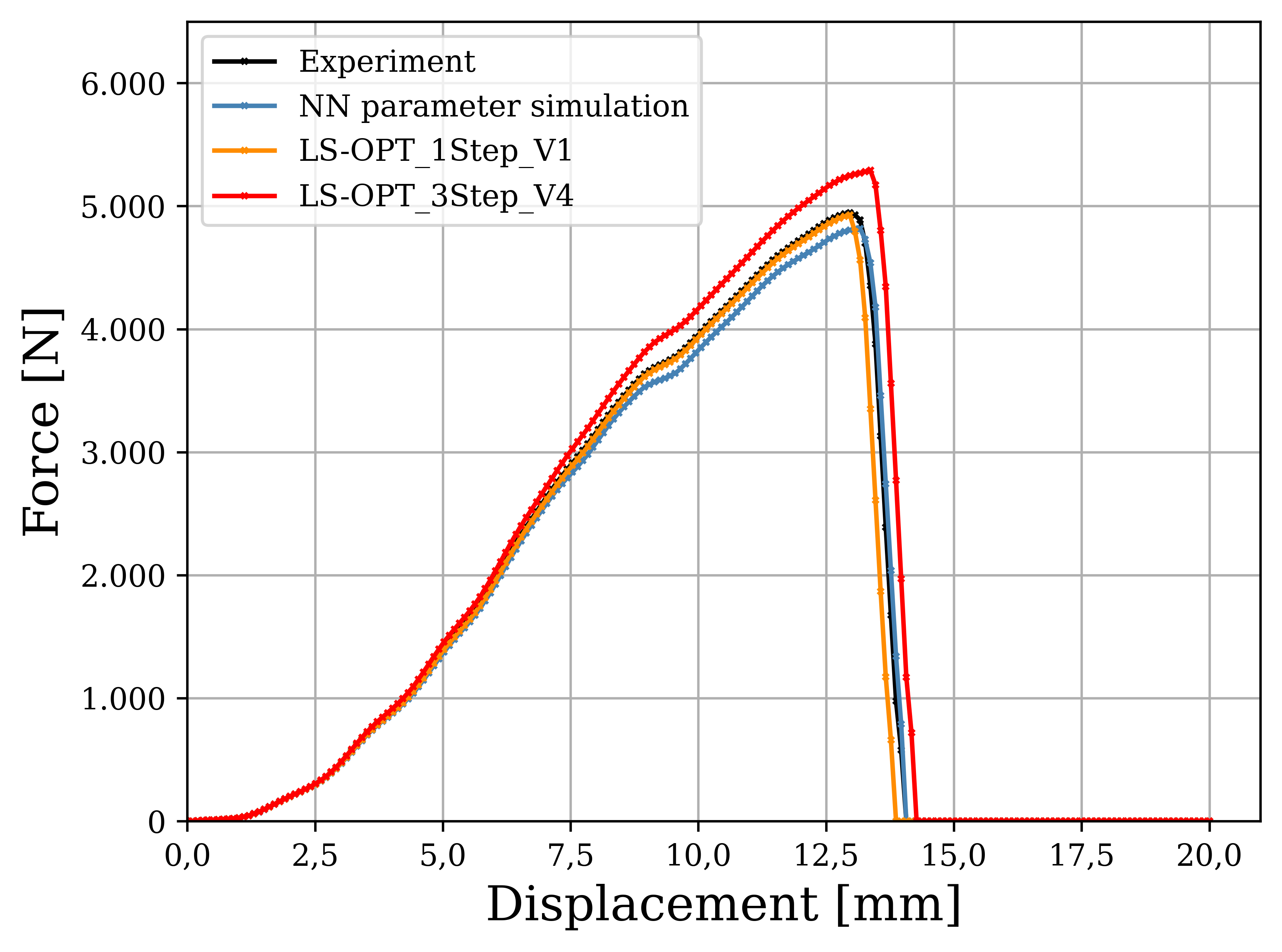

Supplement: Supplementary file 1 [file materials-15-00643-s001.zip › Supplementary_Material/SOC_NN_Pred_LSOPT_Complete/NN_Run_5/FD_Comparison_Punch_Test.png]

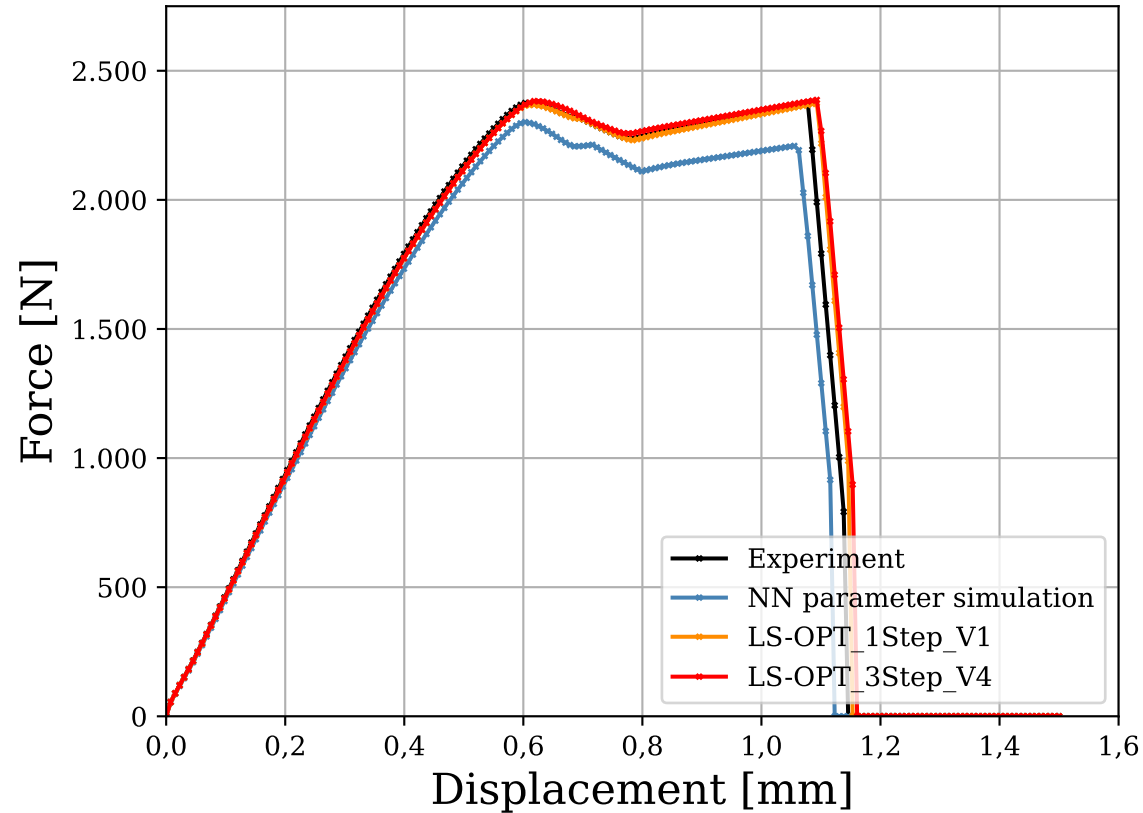

Supplement: Supplementary file 1 [file materials-15-00643-s001.zip › Supplementary_Material/SOC_NN_Pred_LSOPT_Complete/NN_Run_5/FD_Comparison_Shear_ASTM_Test.pdf]

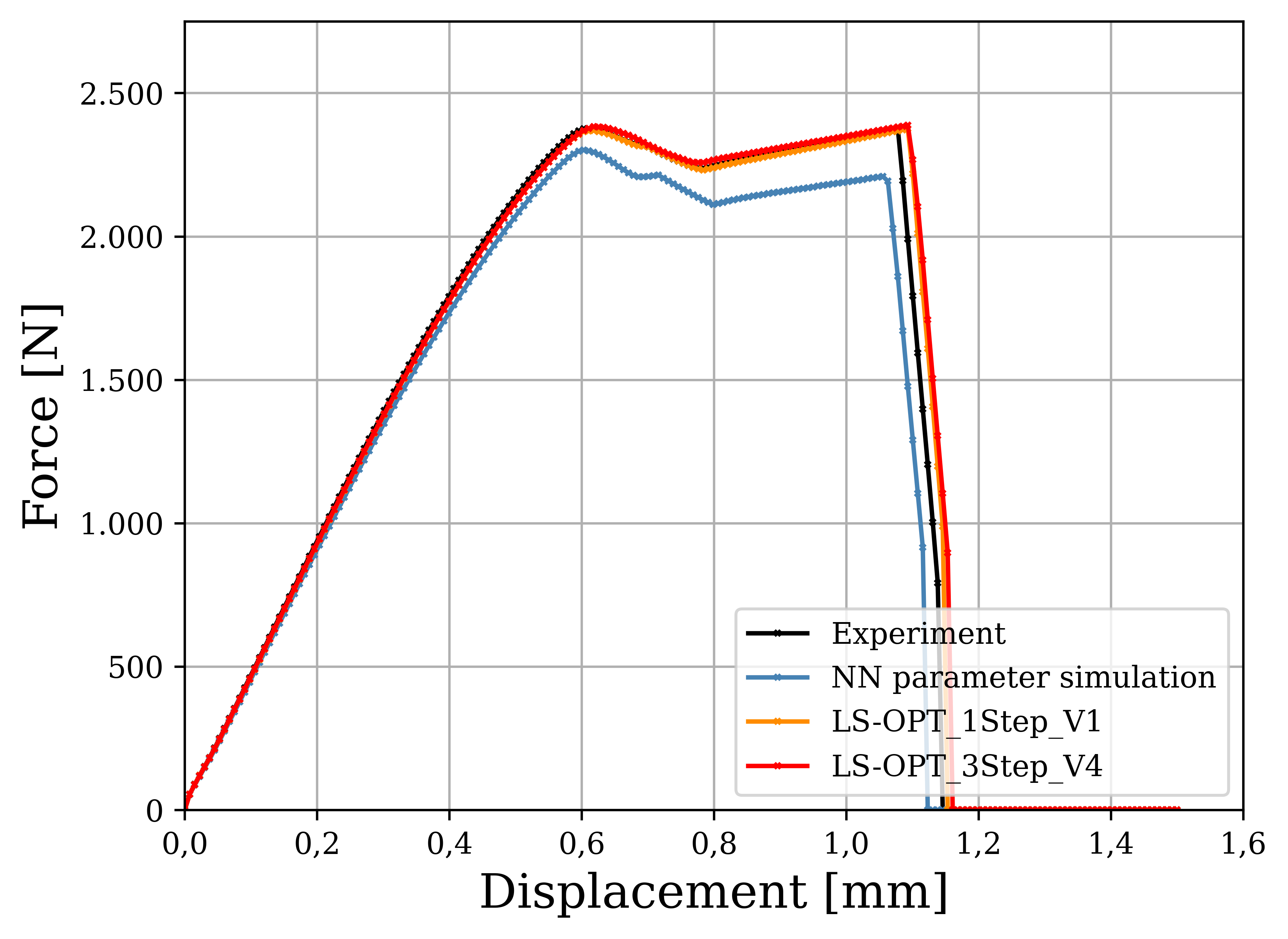

Supplement: Supplementary file 1 [file materials-15-00643-s001.zip › Supplementary_Material/SOC_NN_Pred_LSOPT_Complete/NN_Run_5/FD_Comparison_Shear_ASTM_Test.png]

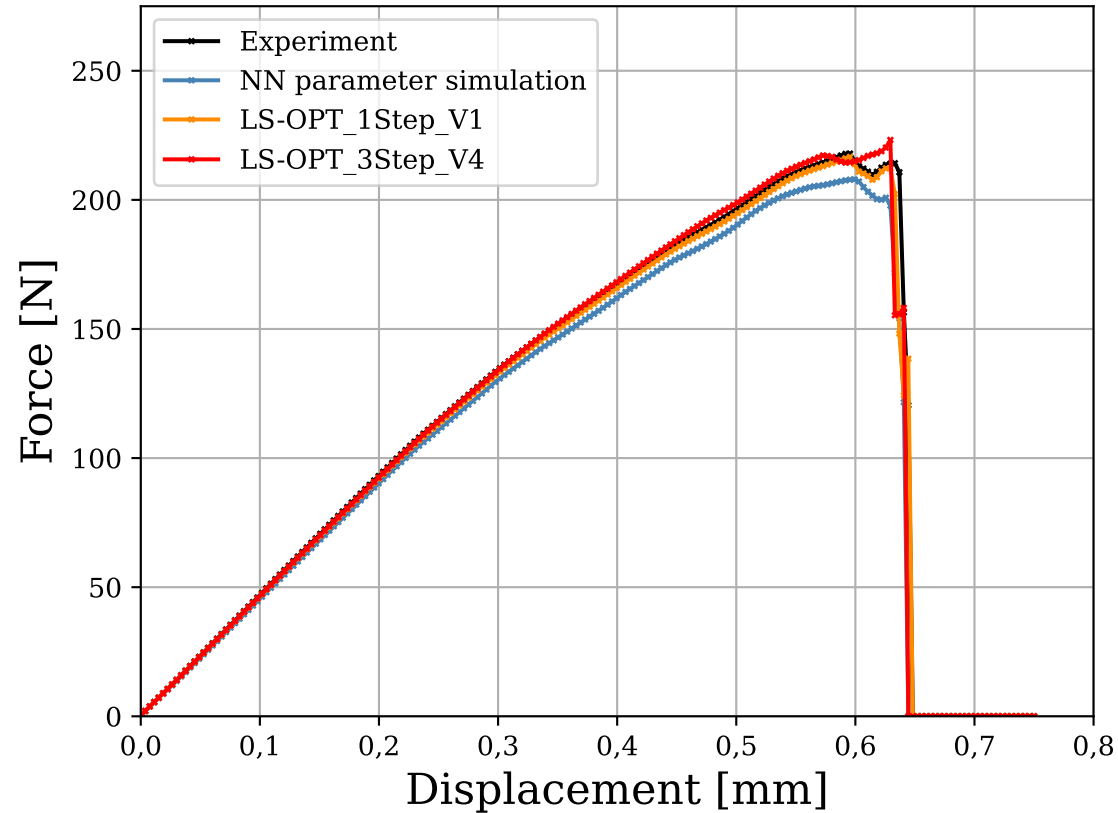

Supplement: Supplementary file 1 [file materials-15-00643-s001.zip › Supplementary_Material/SOC_NN_Pred_LSOPT_Complete/NN_Run_5/FD_Comparison_Shear_Dynamore_Test.pdf]

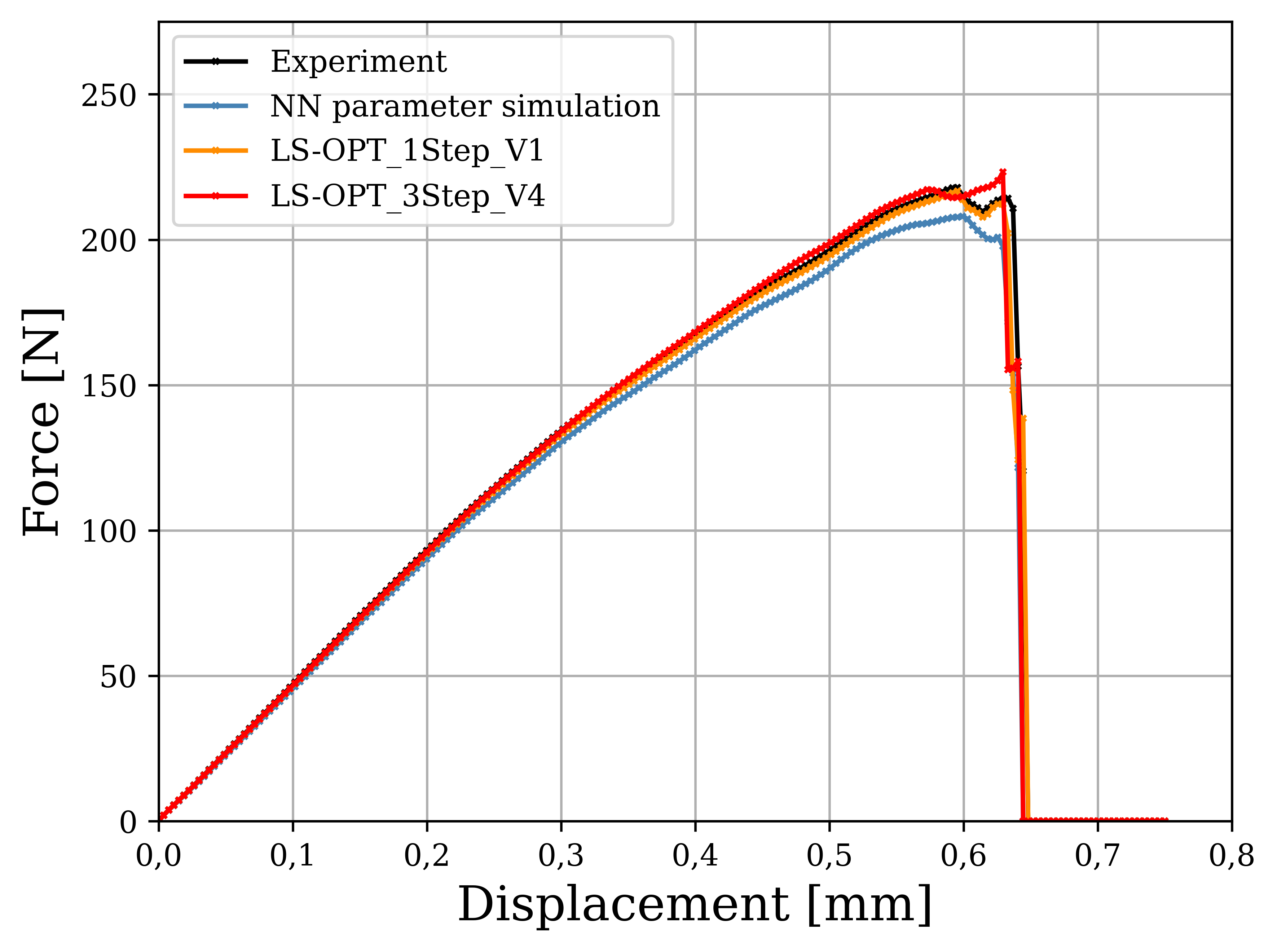

Supplement: Supplementary file 1 [file materials-15-00643-s001.zip › Supplementary_Material/SOC_NN_Pred_LSOPT_Complete/NN_Run_5/FD_Comparison_Shear_Dynamore_Test.png]

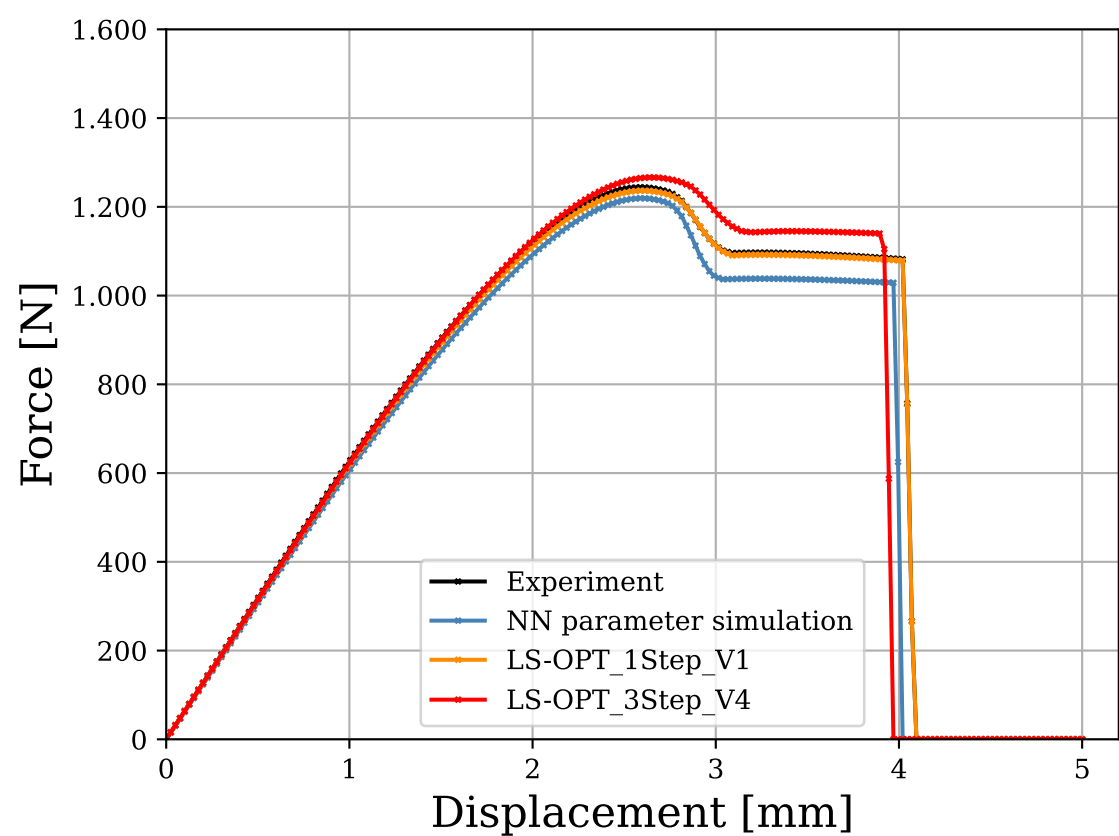

Supplement: Supplementary file 1 [file materials-15-00643-s001.zip › Supplementary_Material/SOC_NN_Pred_LSOPT_Complete/NN_Run_5/FD_Comparison_Tensile_Test.pdf]

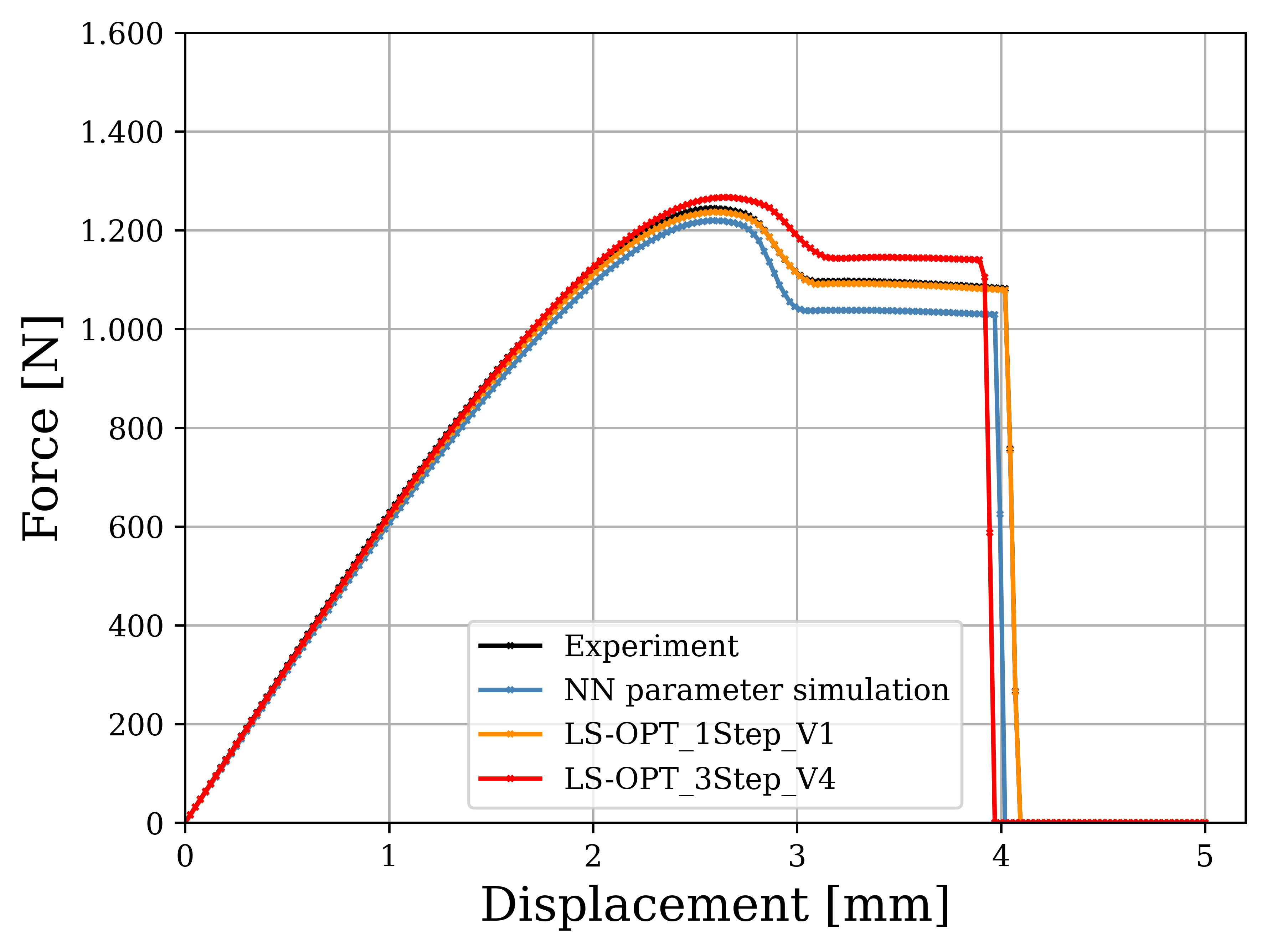

Supplement: Supplementary file 1 [file materials-15-00643-s001.zip › Supplementary_Material/SOC_NN_Pred_LSOPT_Complete/NN_Run_5/FD_Comparison_Tensile_Test.png]

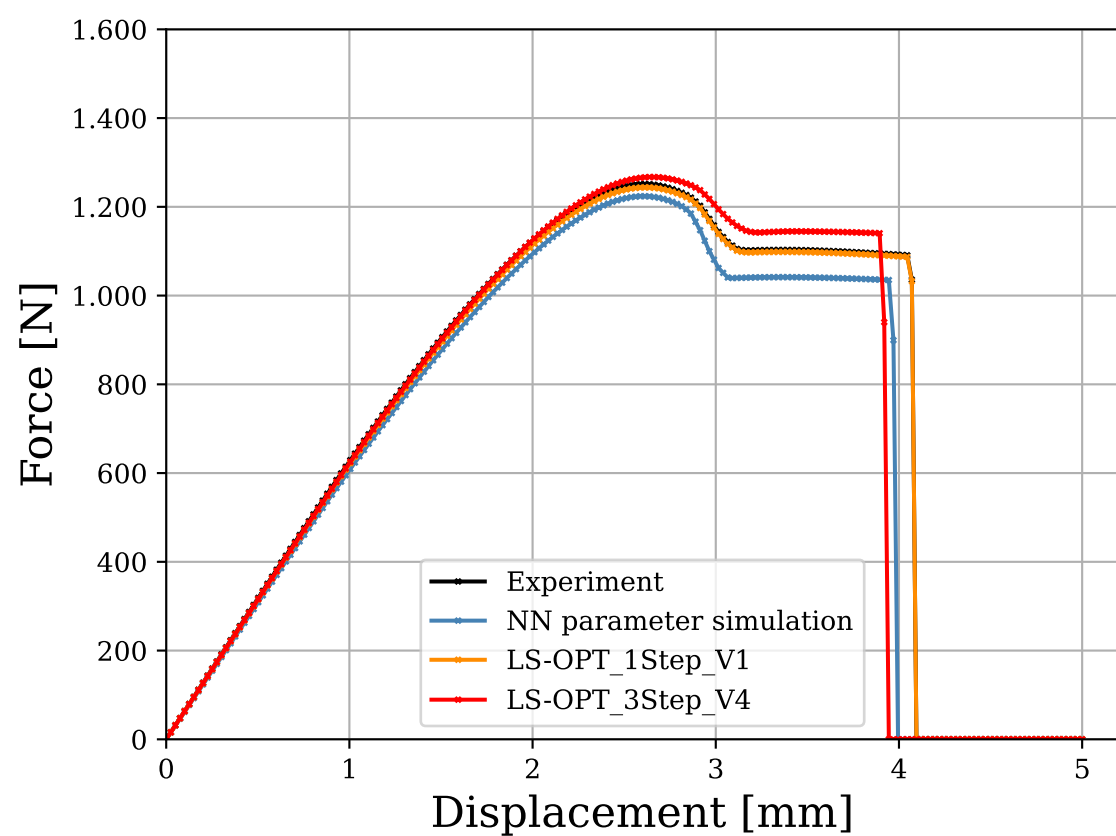

Supplement: Supplementary file 1 [file materials-15-00643-s001.zip › Supplementary_Material/SOC_NN_Pred_LSOPT_Complete/NN_Run_5/FD_Comparison_Tensile_Test_V1.pdf]

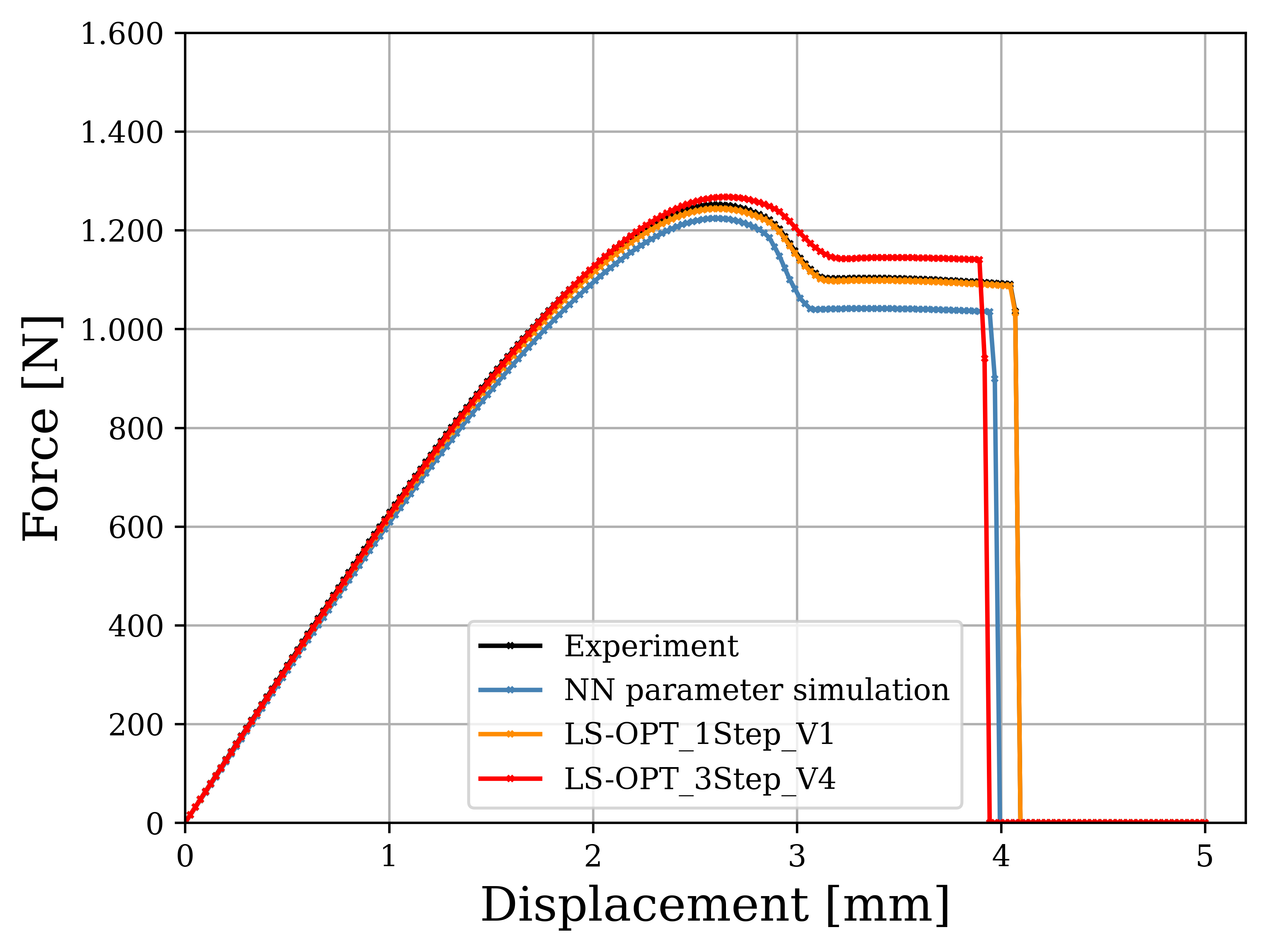

Supplement: Supplementary file 1 [file materials-15-00643-s001.zip › Supplementary_Material/SOC_NN_Pred_LSOPT_Complete/NN_Run_5/FD_Comparison_Tensile_Test_V1.png]

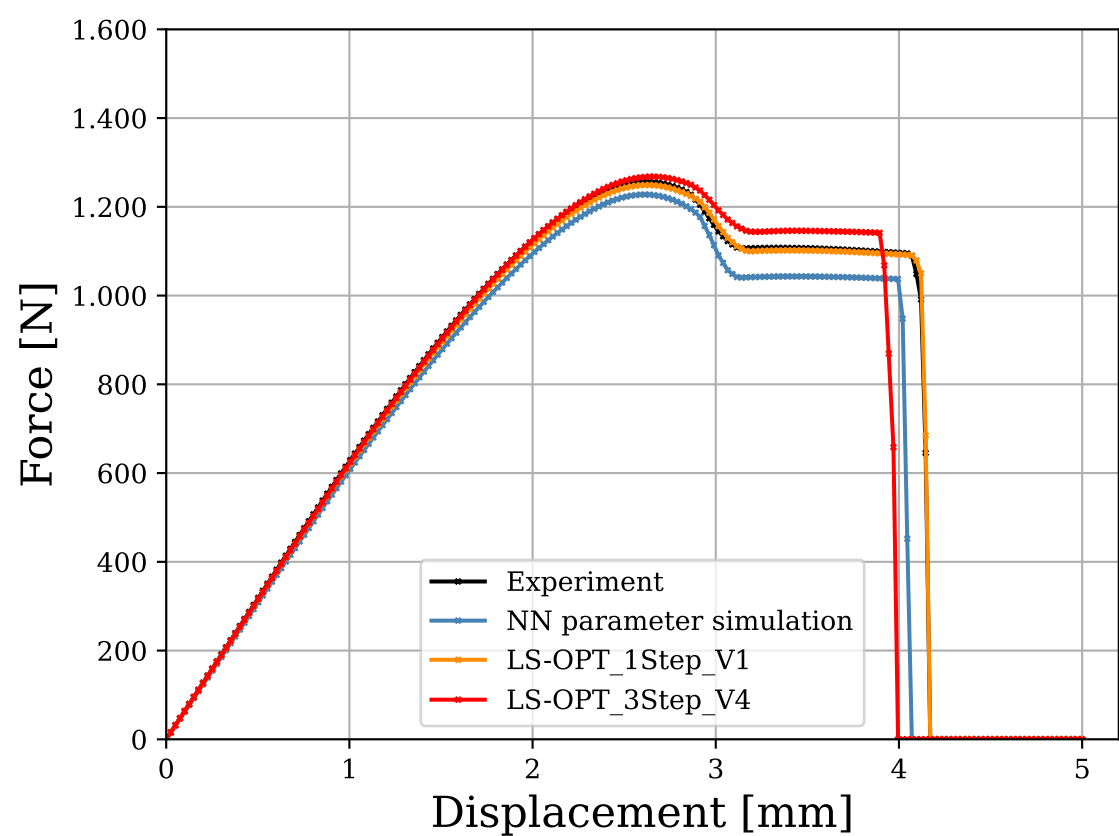

Supplement: Supplementary file 1 [file materials-15-00643-s001.zip › Supplementary_Material/SOC_NN_Pred_LSOPT_Complete/NN_Run_5/FD_Comparison_Tensile_Test_V2.pdf]

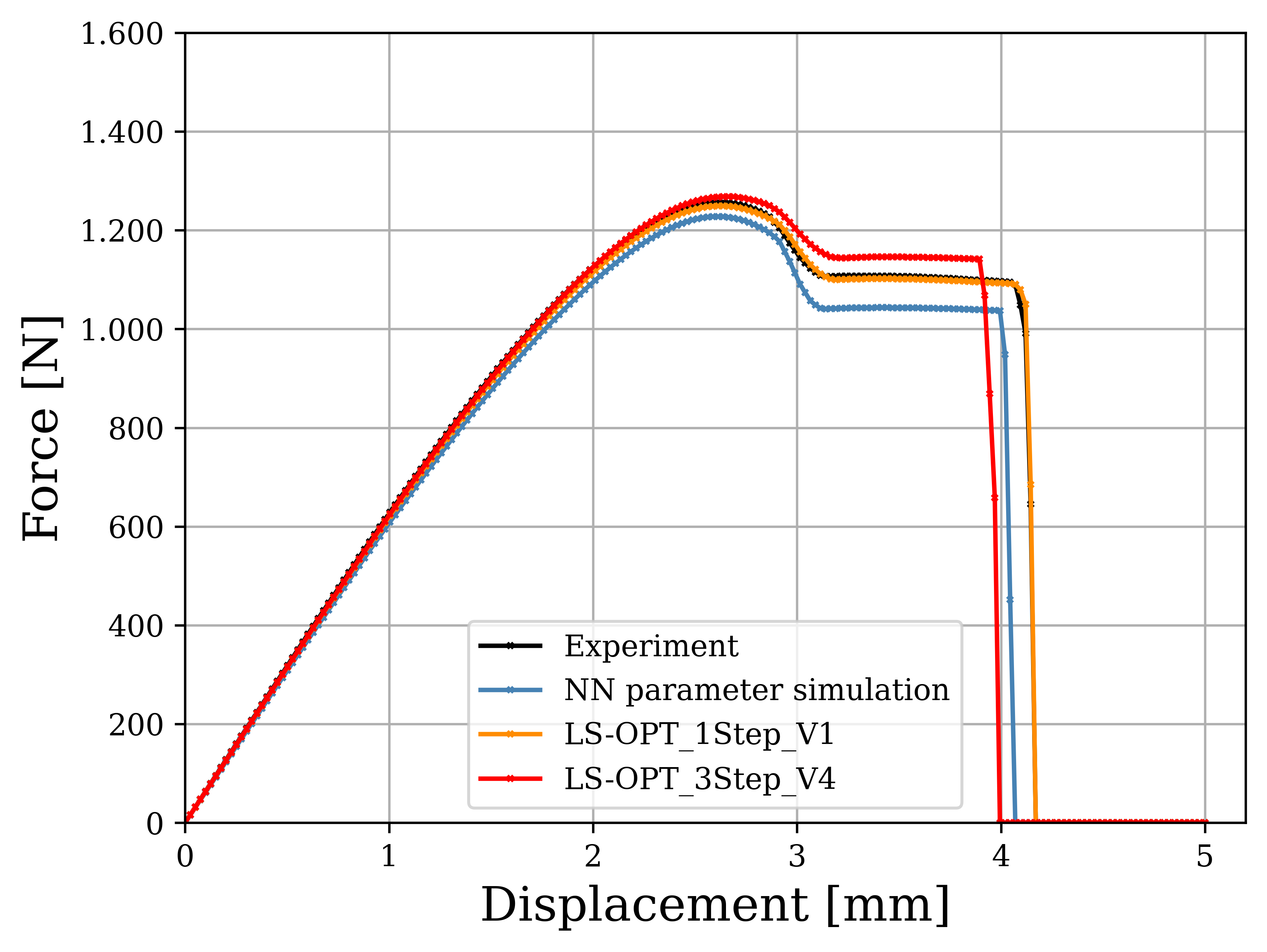

Supplement: Supplementary file 1 [file materials-15-00643-s001.zip › Supplementary_Material/SOC_NN_Pred_LSOPT_Complete/NN_Run_5/FD_Comparison_Tensile_Test_V2.png]

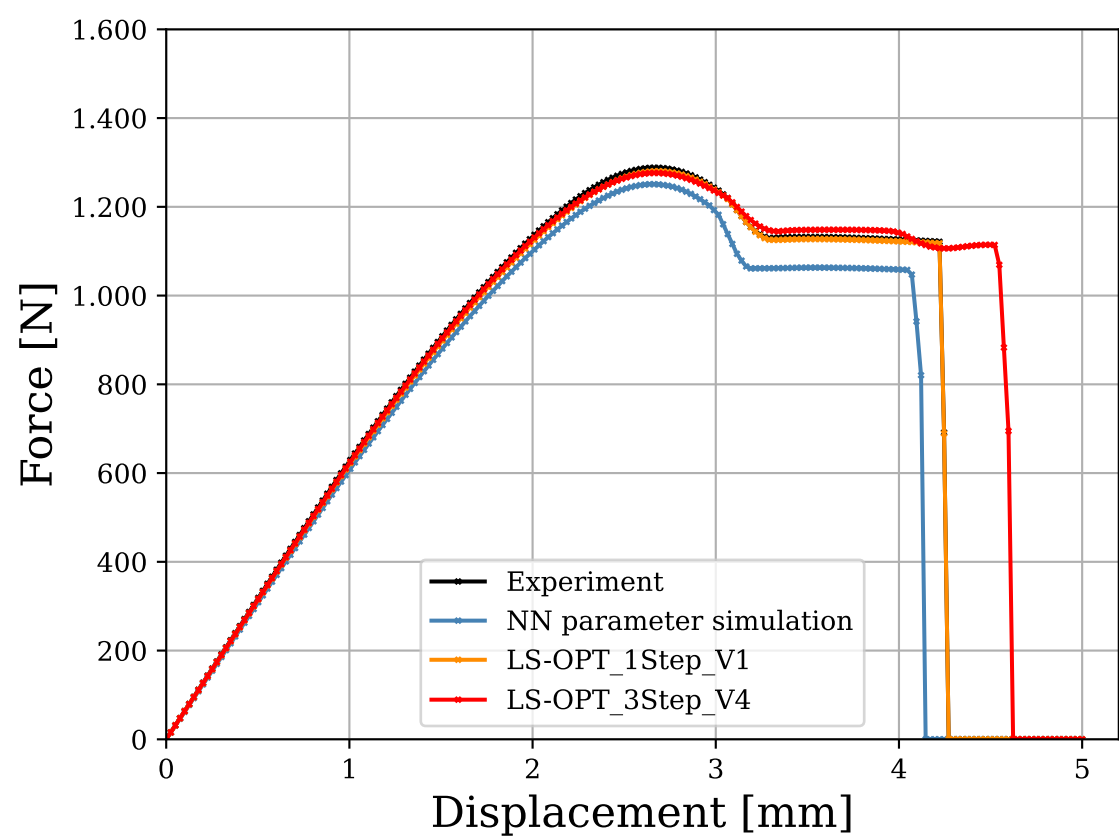

Supplement: Supplementary file 1 [file materials-15-00643-s001.zip › Supplementary_Material/SOC_NN_Pred_LSOPT_Complete/NN_Run_5/FD_Comparison_Tensile_Test_V3.pdf]

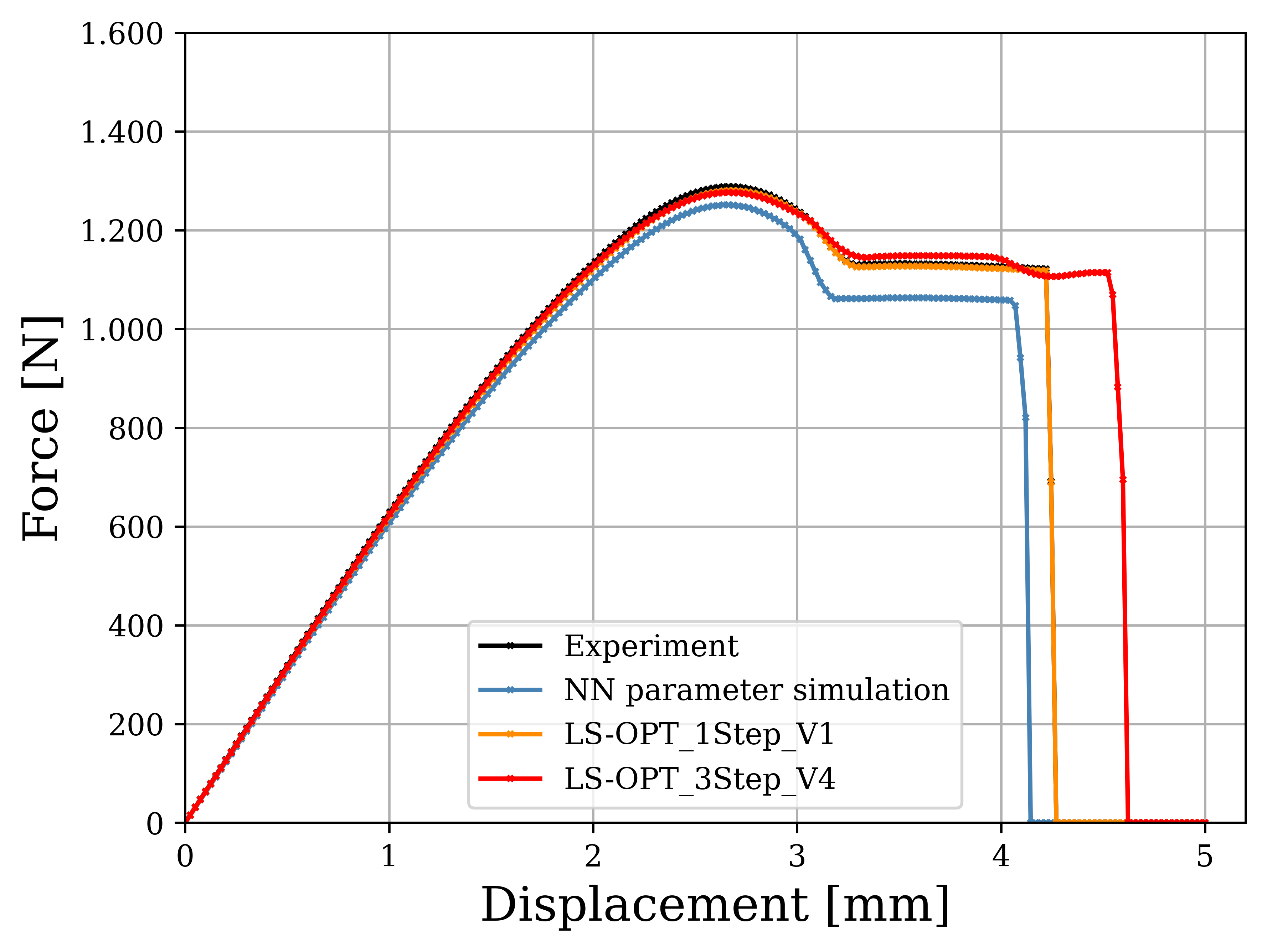

Supplement: Supplementary file 1 [file materials-15-00643-s001.zip › Supplementary_Material/SOC_NN_Pred_LSOPT_Complete/NN_Run_5/FD_Comparison_Tensile_Test_V3.png]

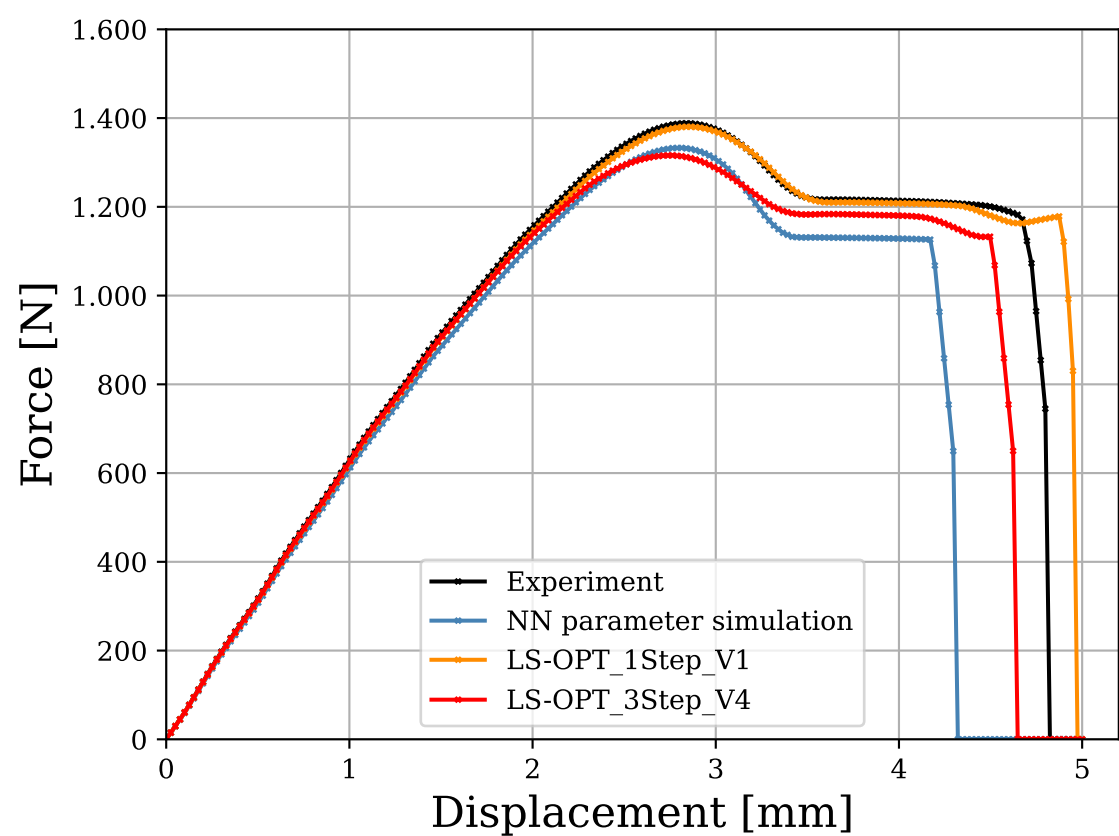

Supplement: Supplementary file 1 [file materials-15-00643-s001.zip › Supplementary_Material/SOC_NN_Pred_LSOPT_Complete/NN_Run_5/FD_Comparison_Tensile_Test_V4.pdf]

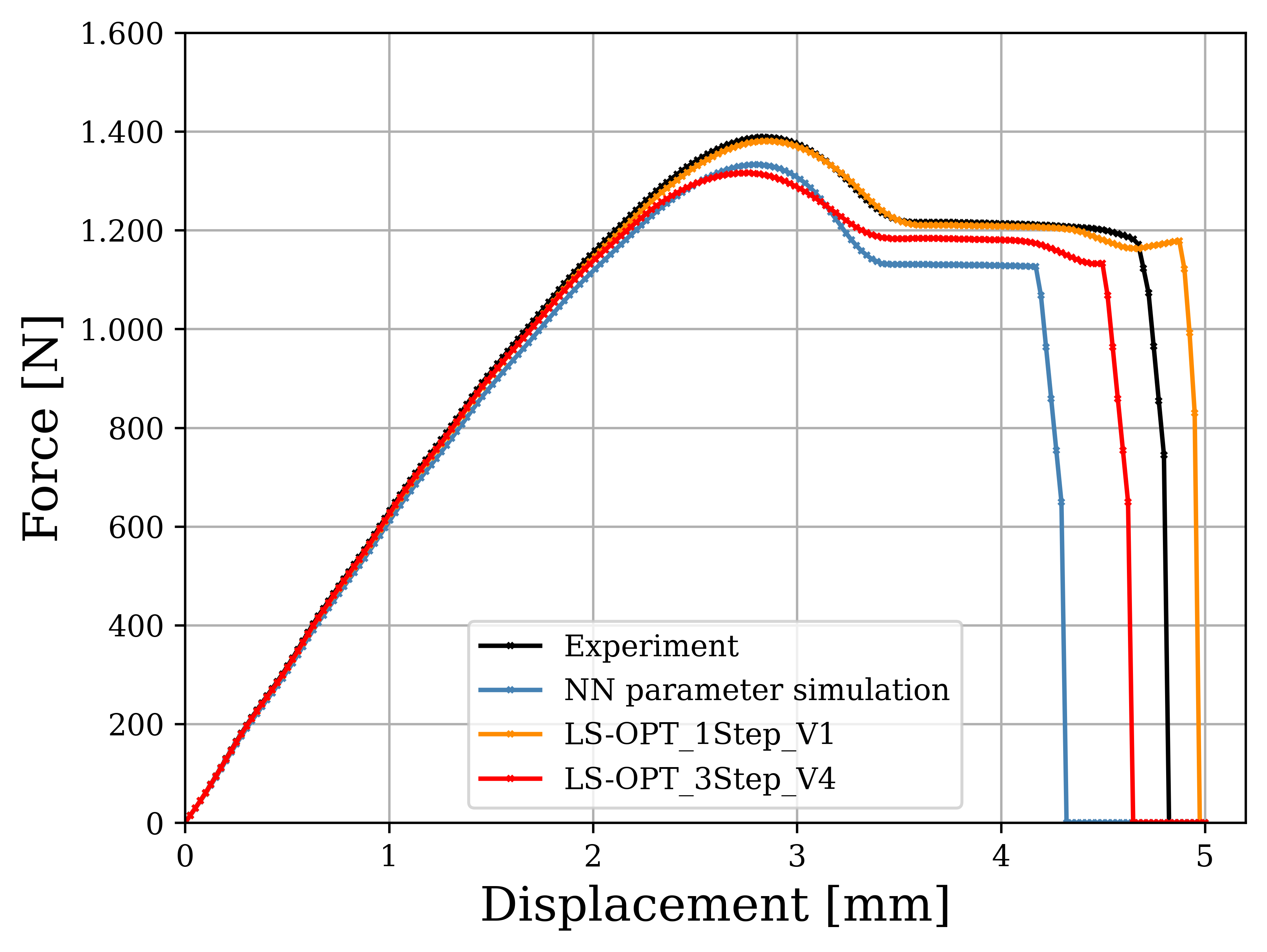

Supplement: Supplementary file 1 [file materials-15-00643-s001.zip › Supplementary_Material/SOC_NN_Pred_LSOPT_Complete/NN_Run_5/FD_Comparison_Tensile_Test_V4.png]

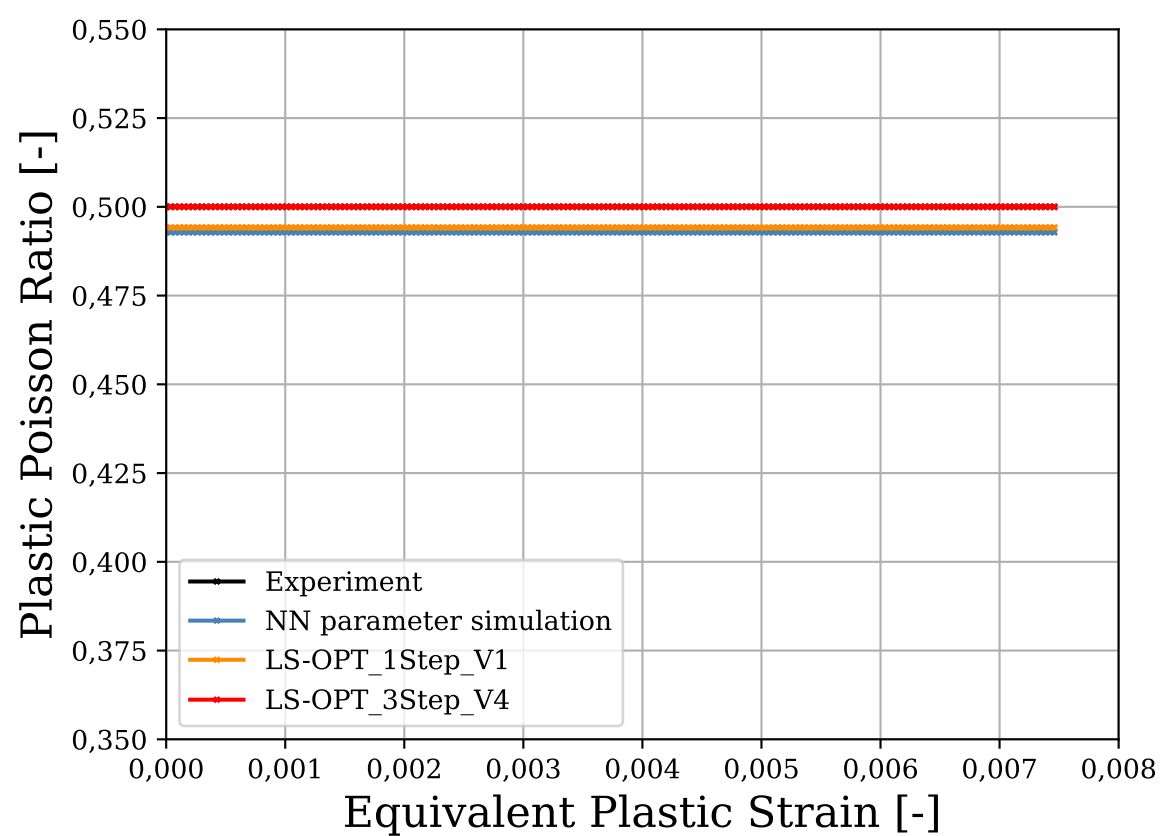

Supplement: Supplementary file 1 [file materials-15-00643-s001.zip › Supplementary_Material/SOC_NN_Pred_LSOPT_Complete/NN_Run_5/PE_Comparison_Compression_Test.pdf]

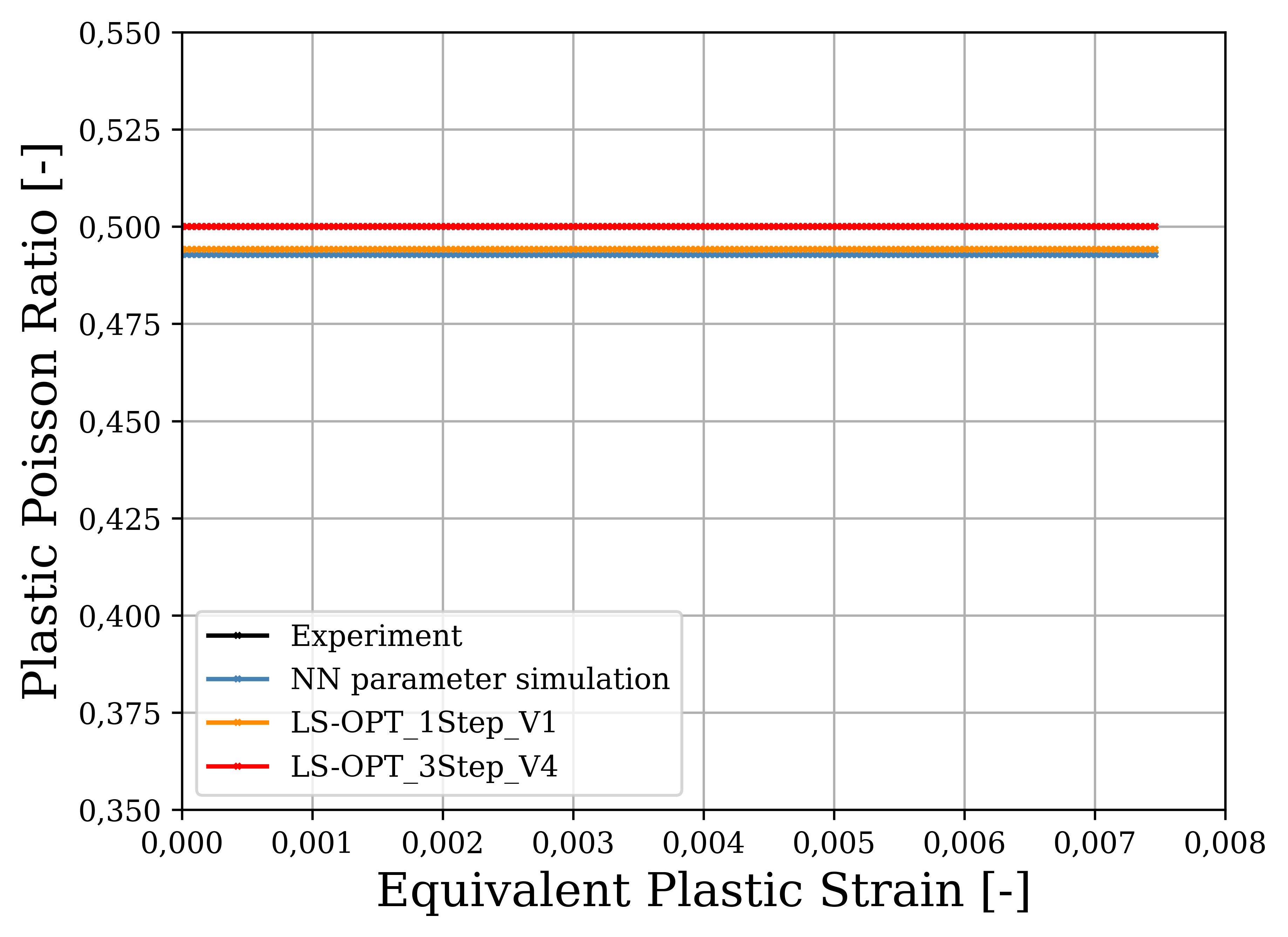

Supplement: Supplementary file 1 [file materials-15-00643-s001.zip › Supplementary_Material/SOC_NN_Pred_LSOPT_Complete/NN_Run_5/PE_Comparison_Compression_Test.png]

Plastic Poisson Ratio [-]

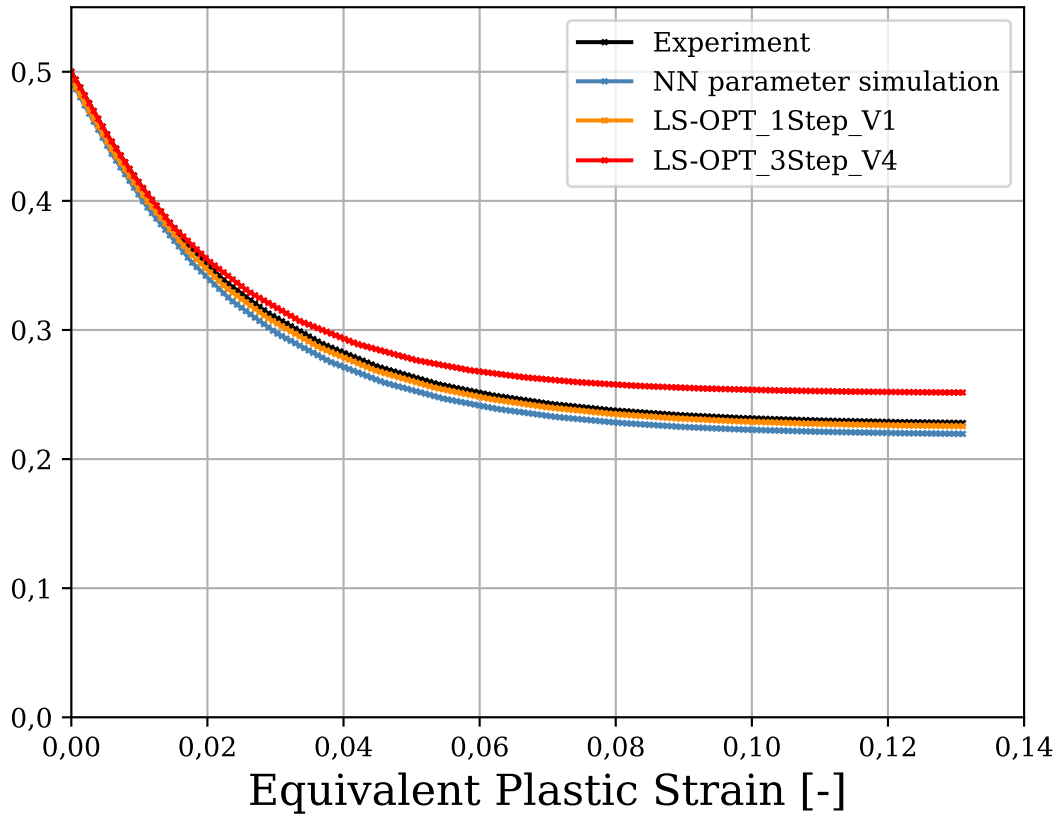

Supplement: Supplementary file 1 [file materials-15-00643-s001.zip › Supplementary_Material/SOC_NN_Pred_LSOPT_Complete/NN_Run_5/PE_Comparison_Punch_Test.pdf]

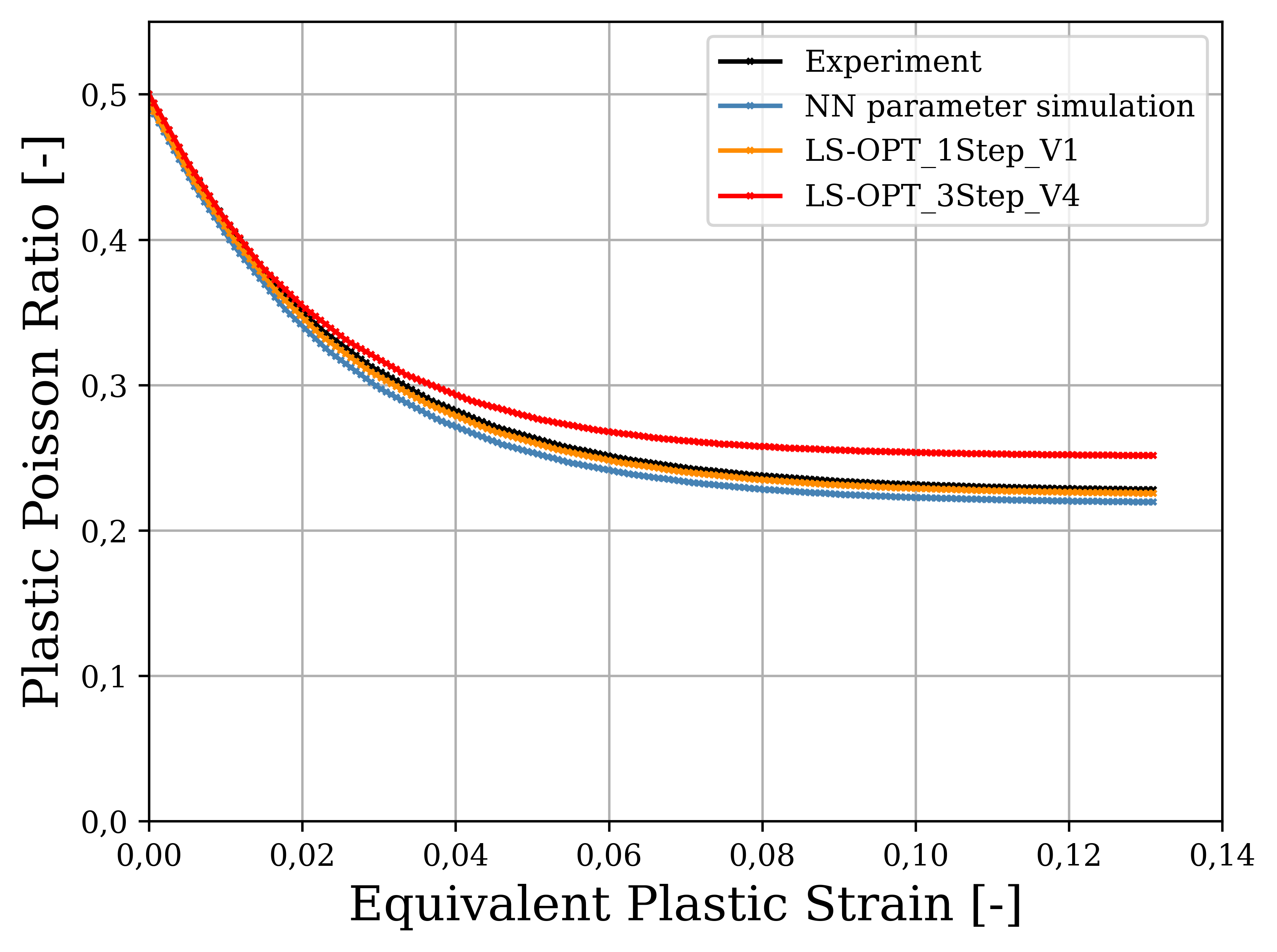

Supplement: Supplementary file 1 [file materials-15-00643-s001.zip › Supplementary_Material/SOC_NN_Pred_LSOPT_Complete/NN_Run_5/PE_Comparison_Punch_Test.png]

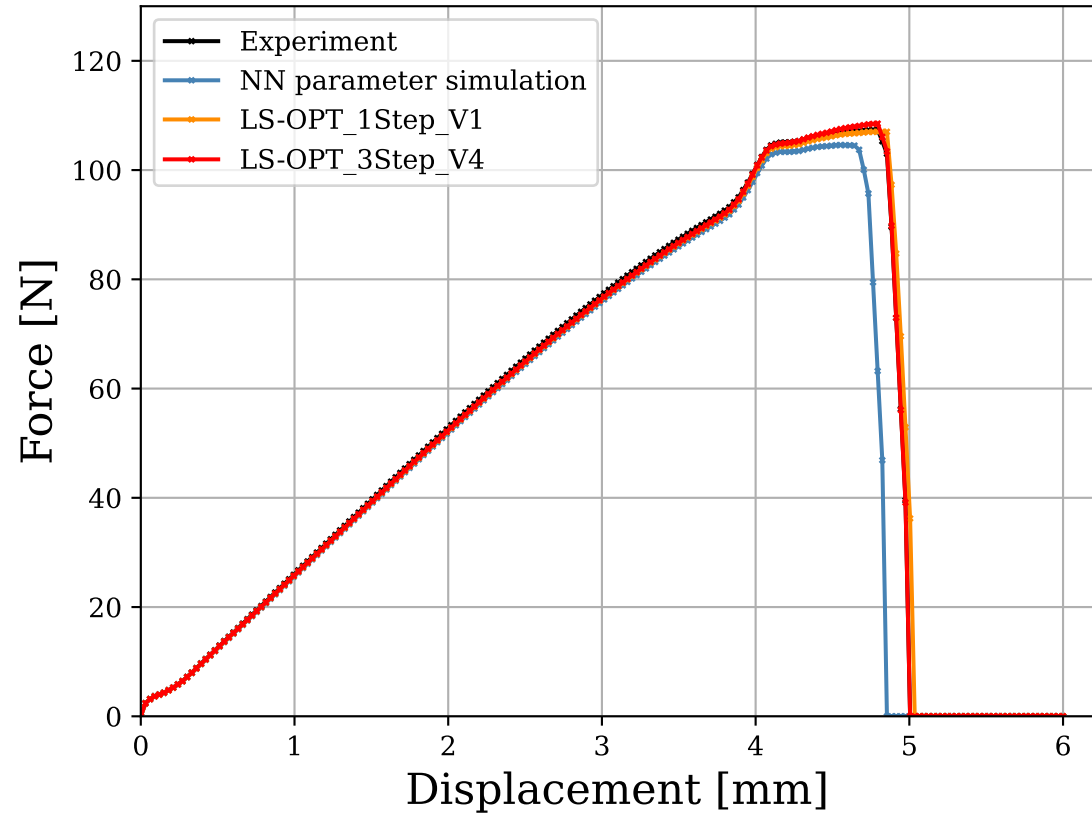

Supplement: Supplementary file 1 [file materials-15-00643-s001.zip › Supplementary_Material/SOC_NN_Pred_LSOPT_Complete/NN_Run_6/FD_Comparison_Bending_Test.pdf]

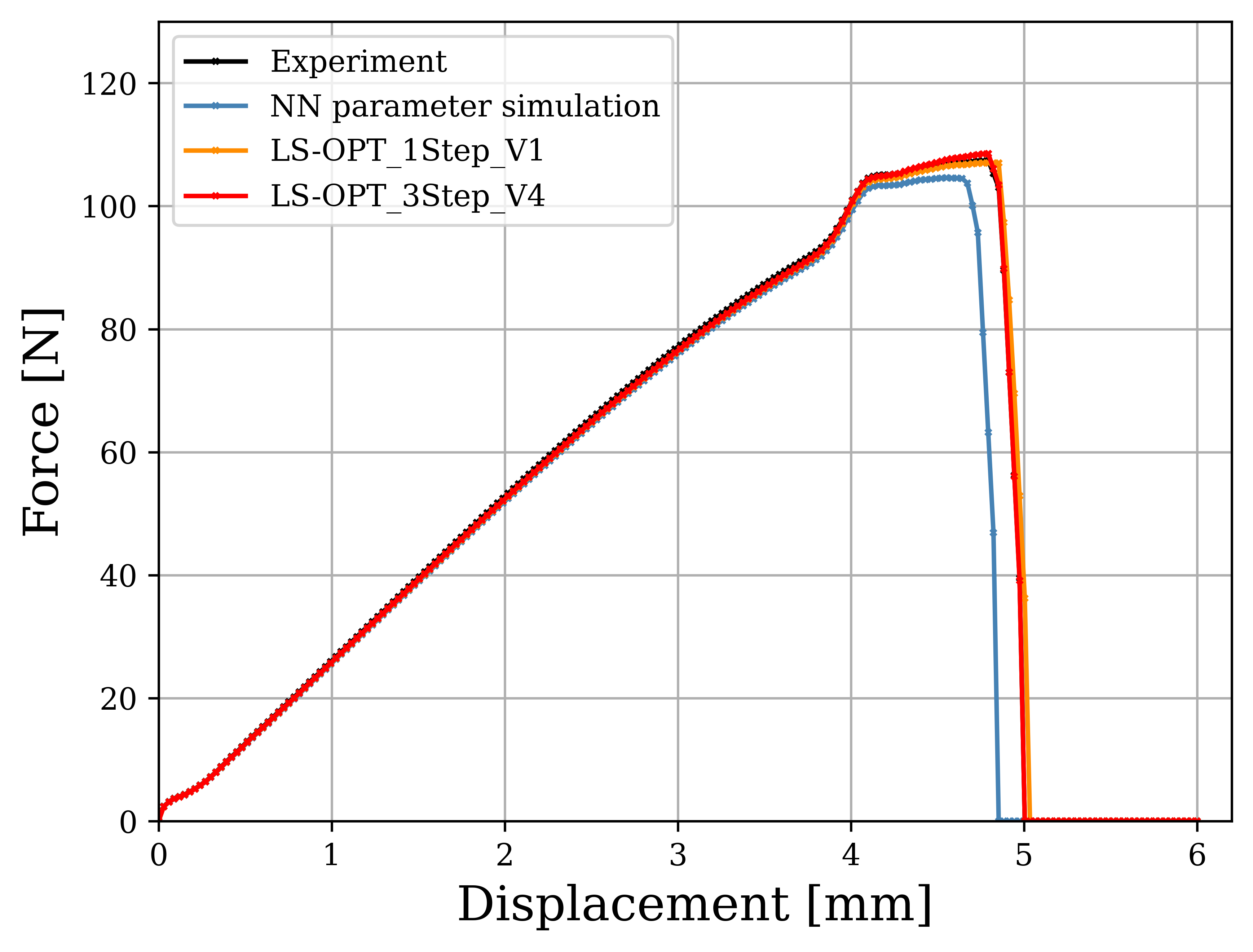

Supplement: Supplementary file 1 [file materials-15-00643-s001.zip › Supplementary_Material/SOC_NN_Pred_LSOPT_Complete/NN_Run_6/FD_Comparison_Bending_Test.png]

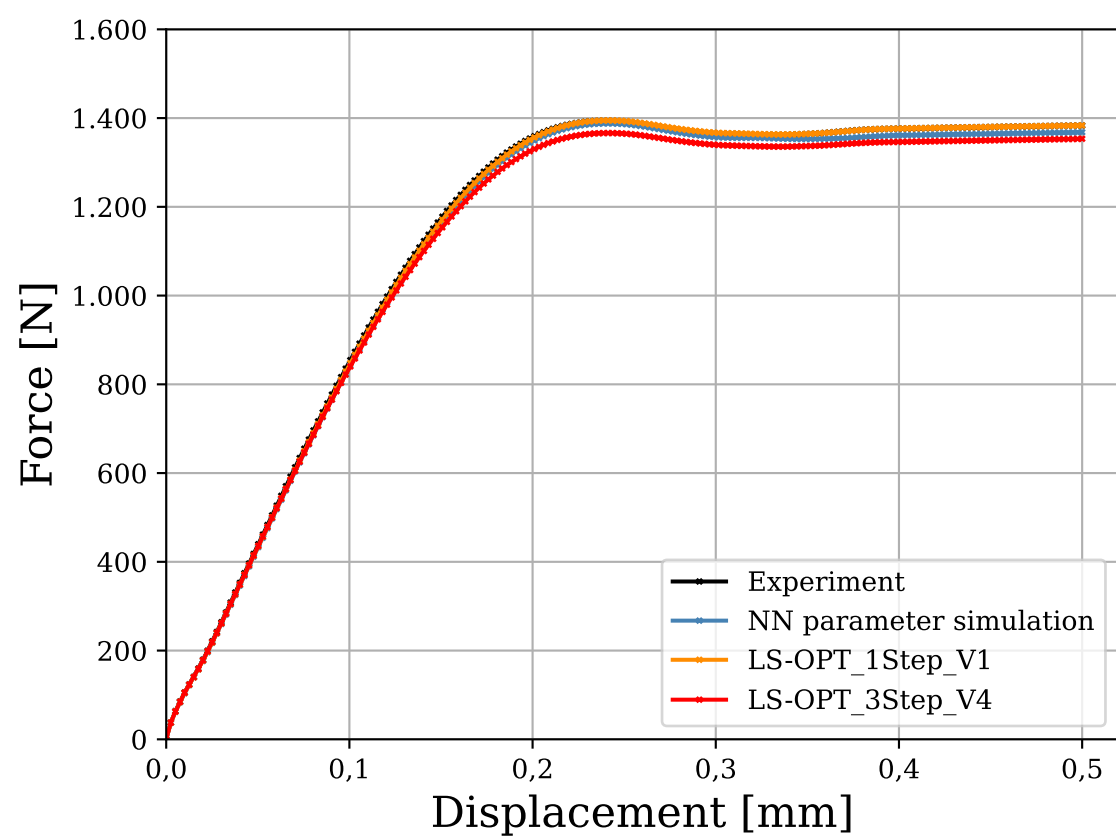

Supplement: Supplementary file 1 [file materials-15-00643-s001.zip › Supplementary_Material/SOC_NN_Pred_LSOPT_Complete/NN_Run_6/FD_Comparison_Compression_Test.pdf]

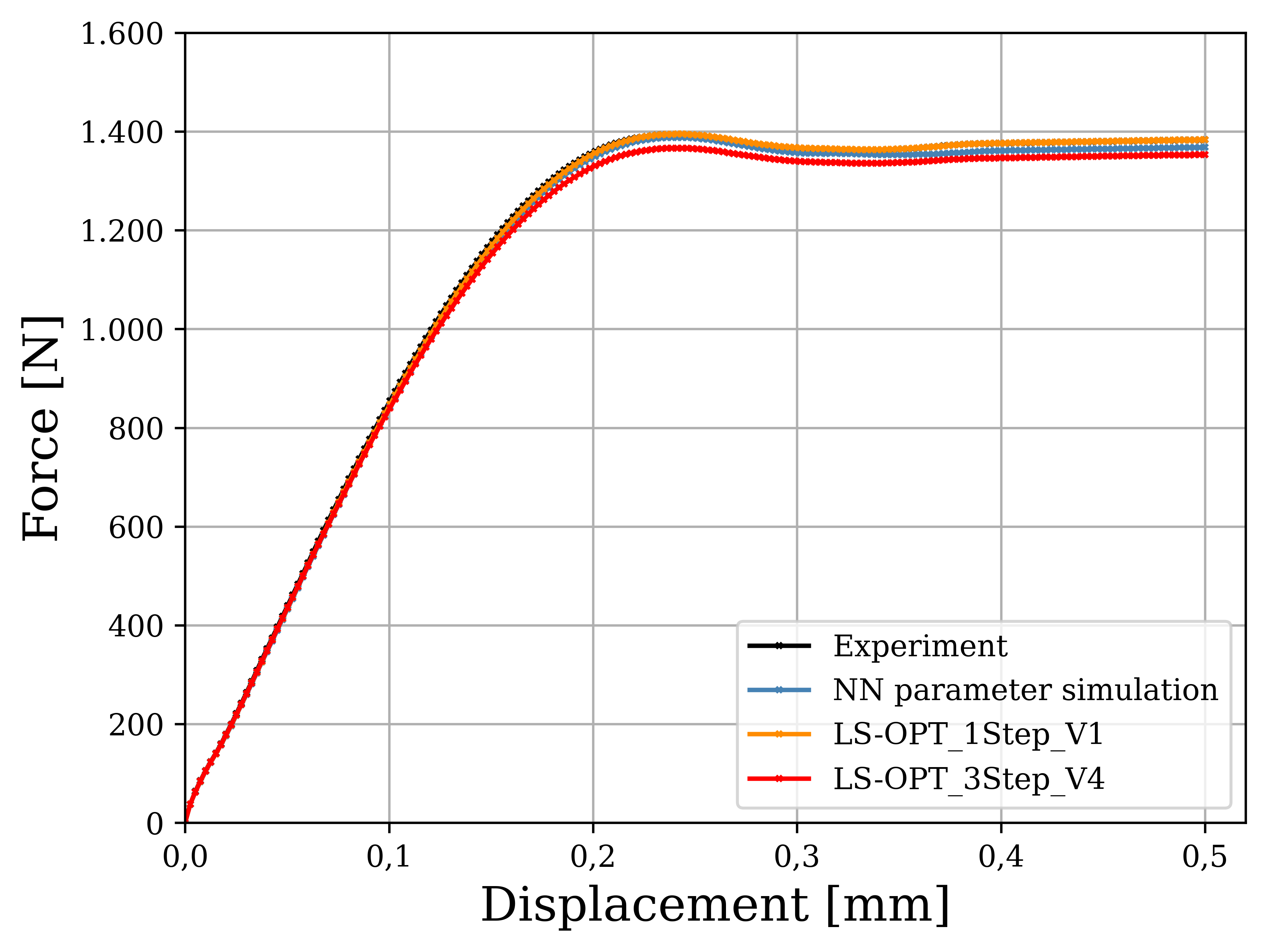

Supplement: Supplementary file 1 [file materials-15-00643-s001.zip › Supplementary_Material/SOC_NN_Pred_LSOPT_Complete/NN_Run_6/FD_Comparison_Compression_Test.png]

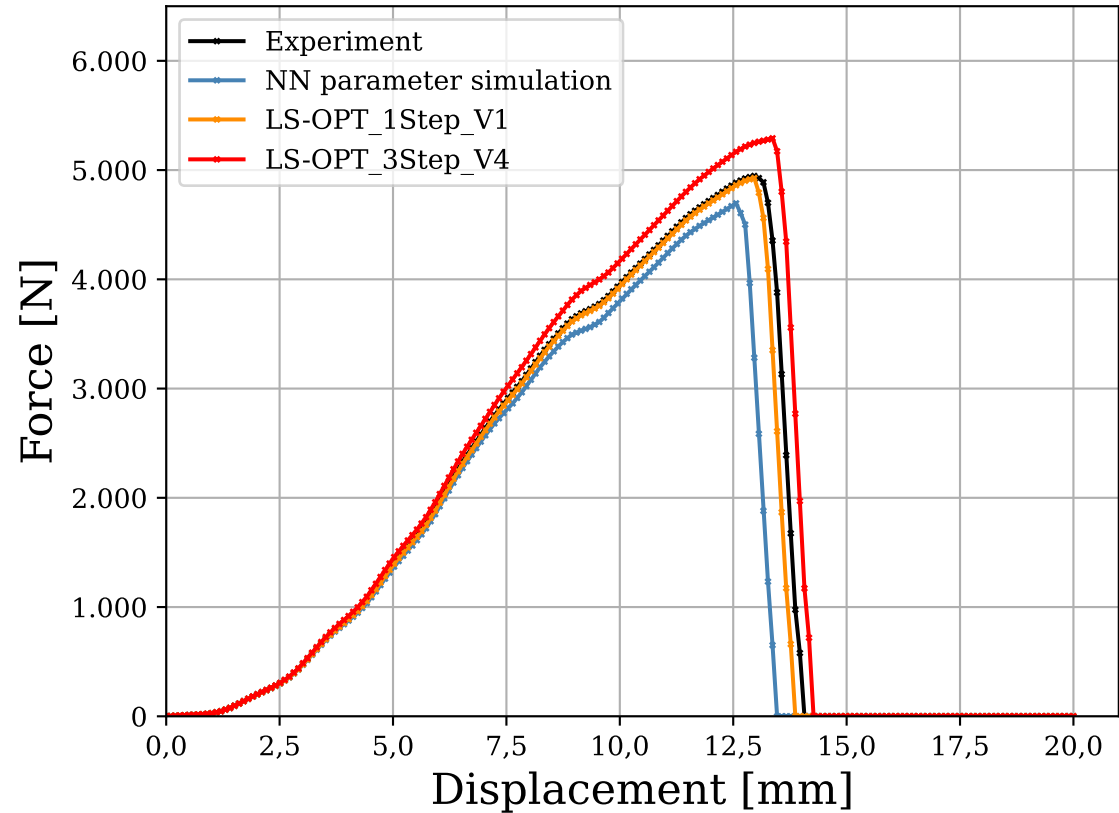

Supplement: Supplementary file 1 [file materials-15-00643-s001.zip › Supplementary_Material/SOC_NN_Pred_LSOPT_Complete/NN_Run_6/FD_Comparison_Punch_Test.pdf]

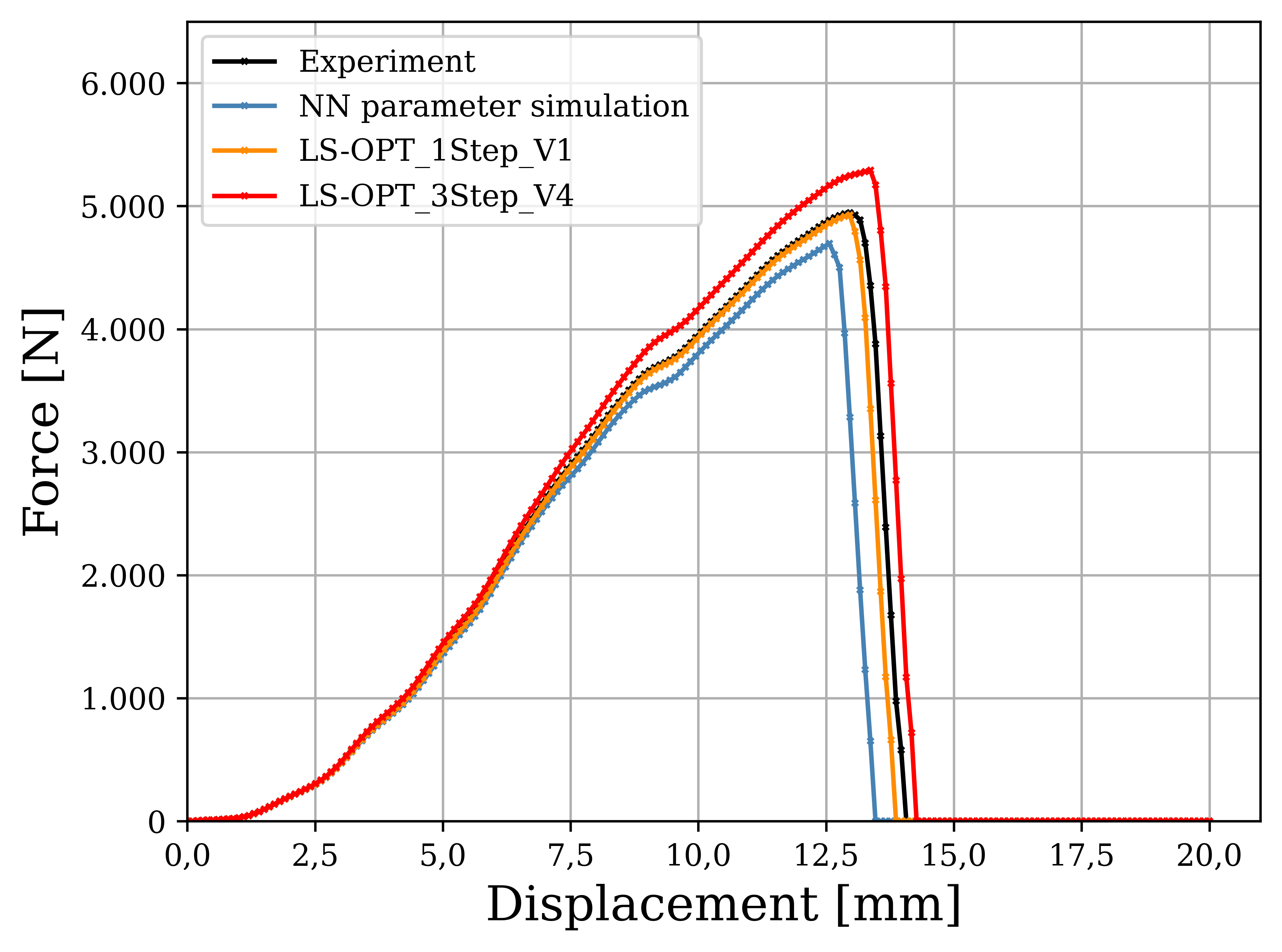

Supplement: Supplementary file 1 [file materials-15-00643-s001.zip › Supplementary_Material/SOC_NN_Pred_LSOPT_Complete/NN_Run_6/FD_Comparison_Punch_Test.png]

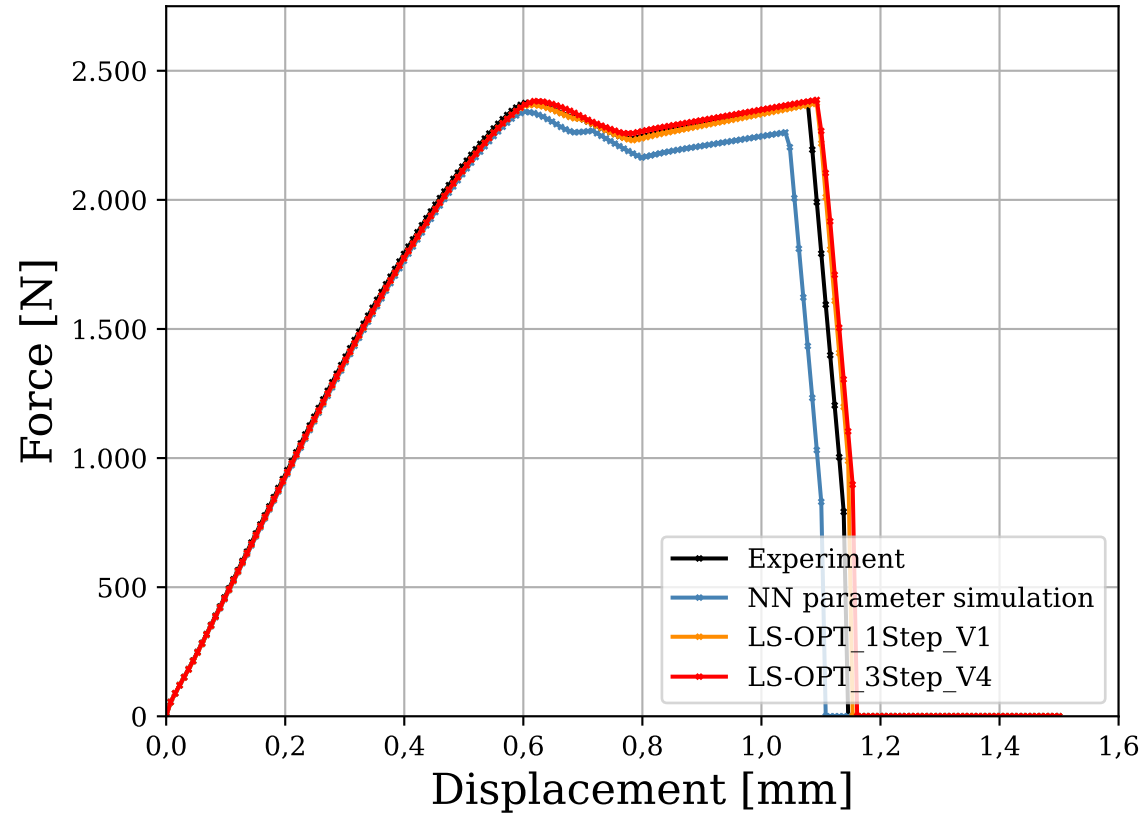

Supplement: Supplementary file 1 [file materials-15-00643-s001.zip › Supplementary_Material/SOC_NN_Pred_LSOPT_Complete/NN_Run_6/FD_Comparison_Shear_ASTM_Test.pdf]

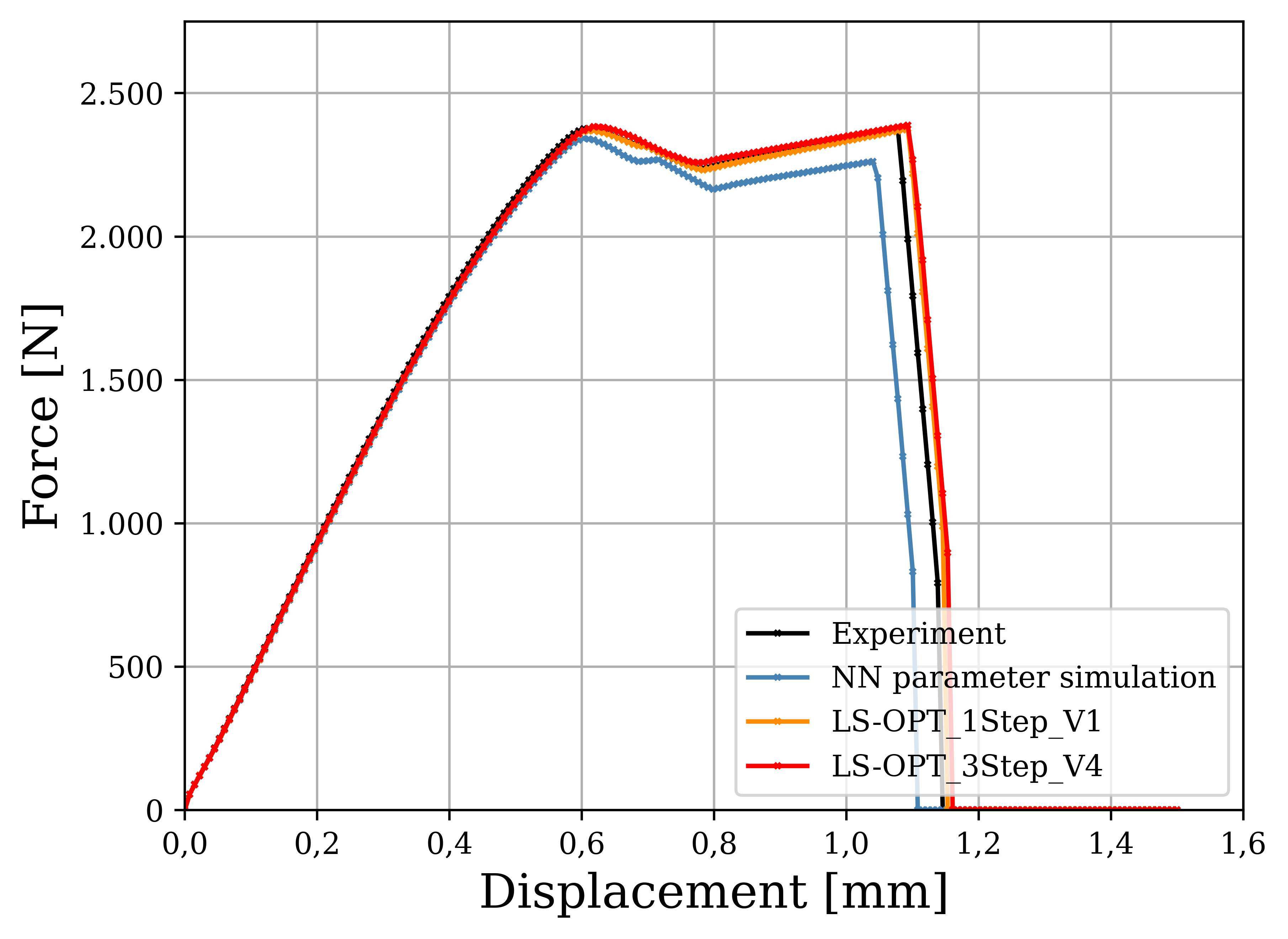

Supplement: Supplementary file 1 [file materials-15-00643-s001.zip › Supplementary_Material/SOC_NN_Pred_LSOPT_Complete/NN_Run_6/FD_Comparison_Shear_ASTM_Test.png]

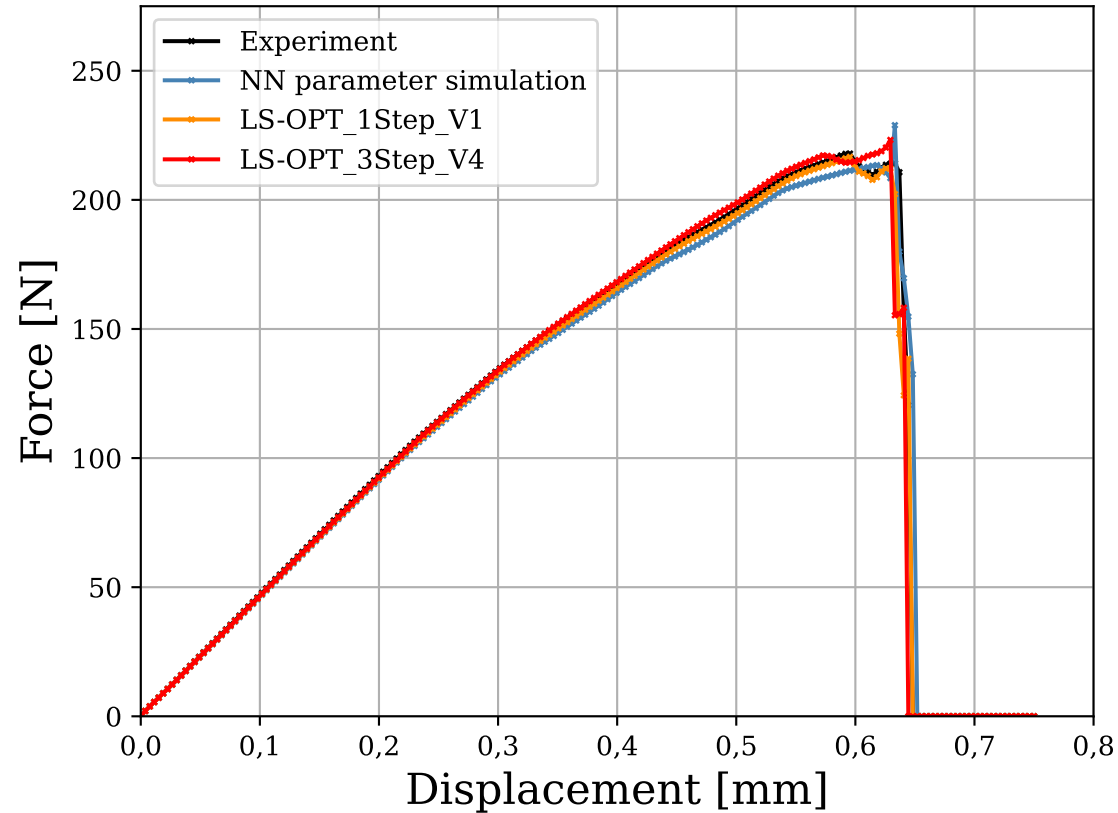

Supplement: Supplementary file 1 [file materials-15-00643-s001.zip › Supplementary_Material/SOC_NN_Pred_LSOPT_Complete/NN_Run_6/FD_Comparison_Shear_Dynamore_Test.pdf]

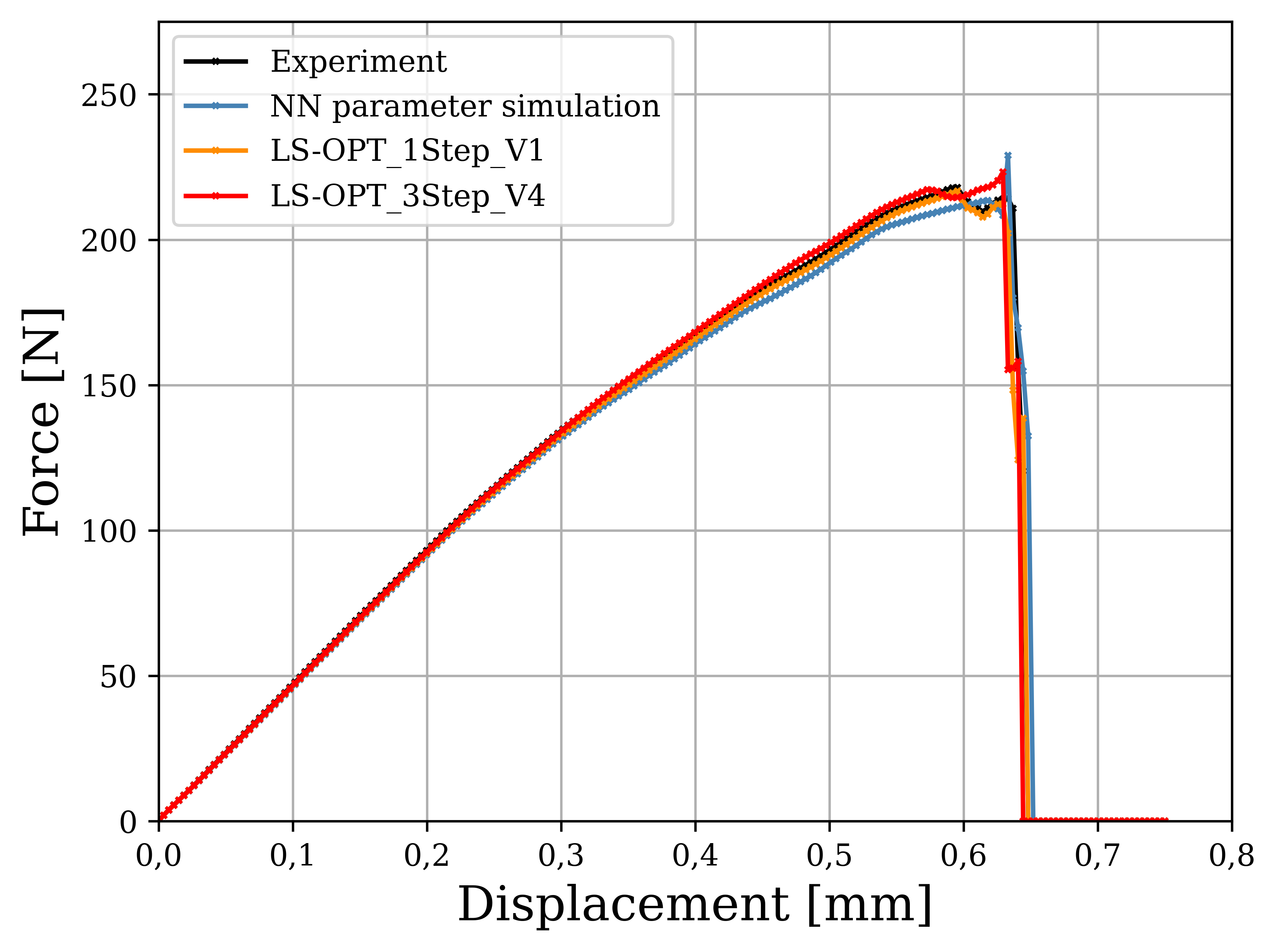

Supplement: Supplementary file 1 [file materials-15-00643-s001.zip › Supplementary_Material/SOC_NN_Pred_LSOPT_Complete/NN_Run_6/FD_Comparison_Shear_Dynamore_Test.png]

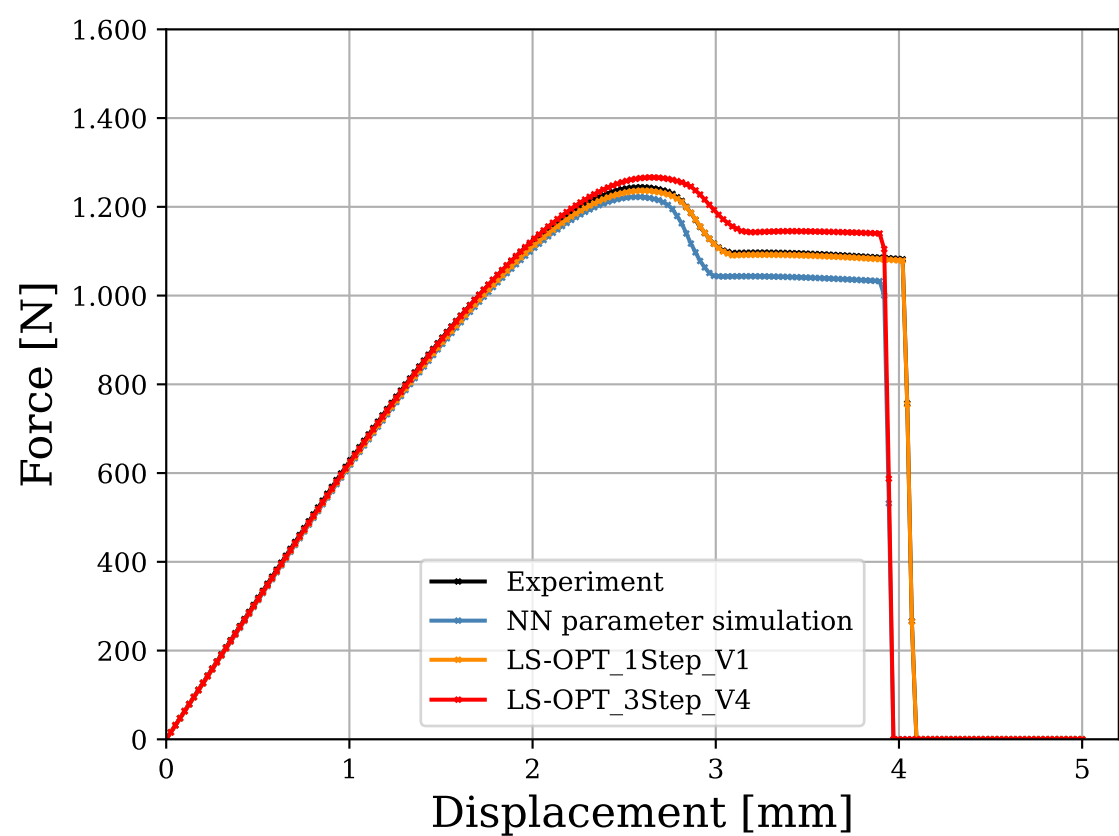

Supplement: Supplementary file 1 [file materials-15-00643-s001.zip › Supplementary_Material/SOC_NN_Pred_LSOPT_Complete/NN_Run_6/FD_Comparison_Tensile_Test.pdf]

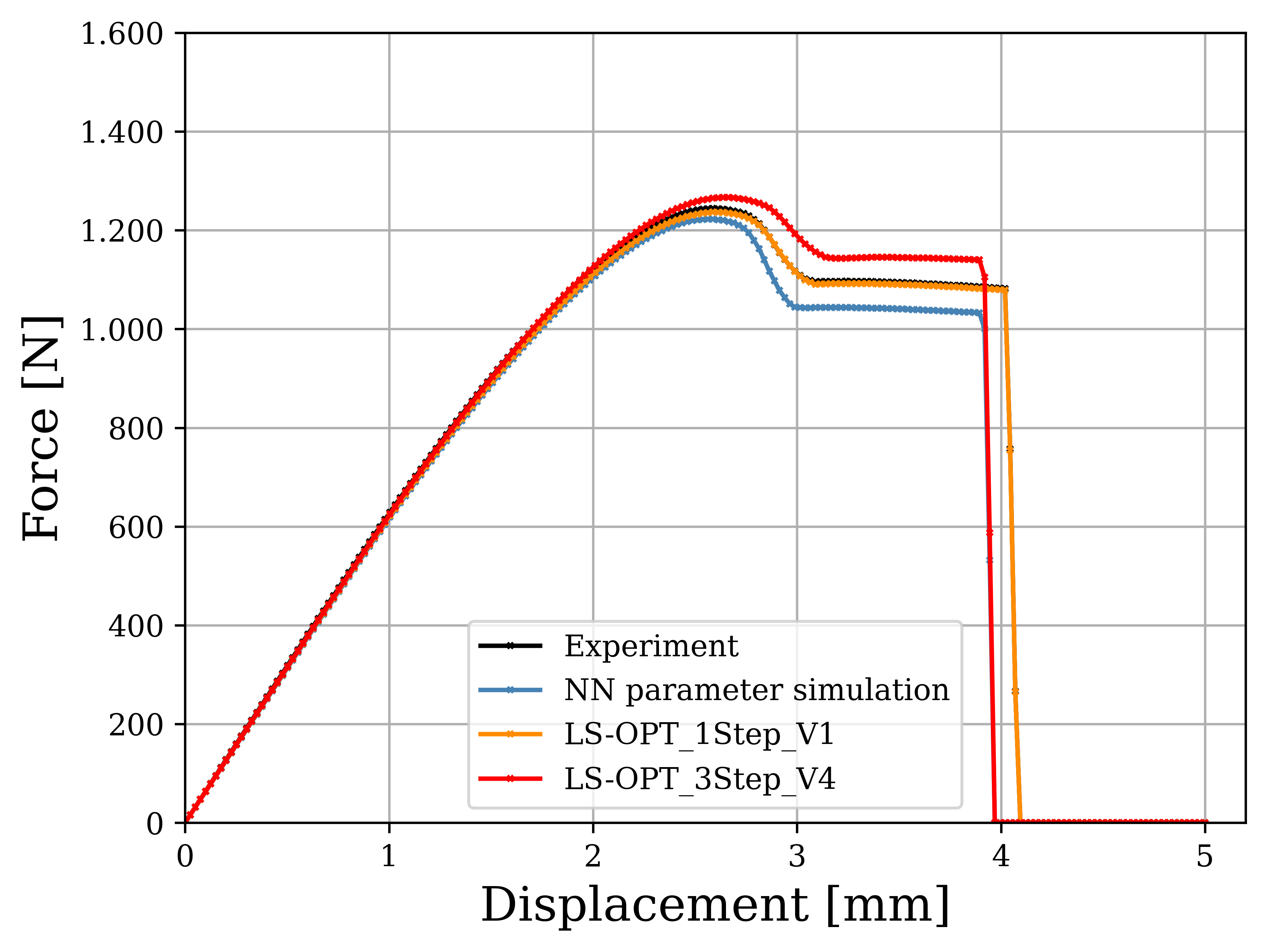

Supplement: Supplementary file 1 [file materials-15-00643-s001.zip › Supplementary_Material/SOC_NN_Pred_LSOPT_Complete/NN_Run_6/FD_Comparison_Tensile_Test.png]

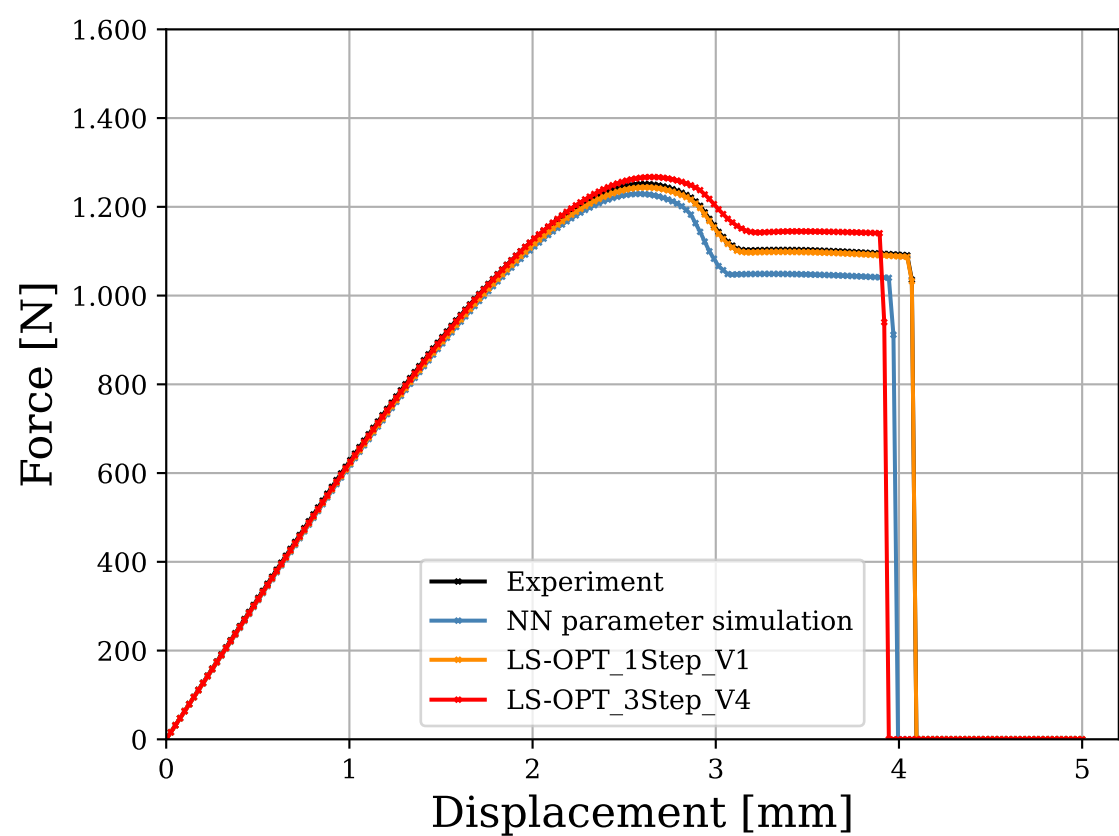

Supplement: Supplementary file 1 [file materials-15-00643-s001.zip › Supplementary_Material/SOC_NN_Pred_LSOPT_Complete/NN_Run_6/FD_Comparison_Tensile_Test_V1.pdf]

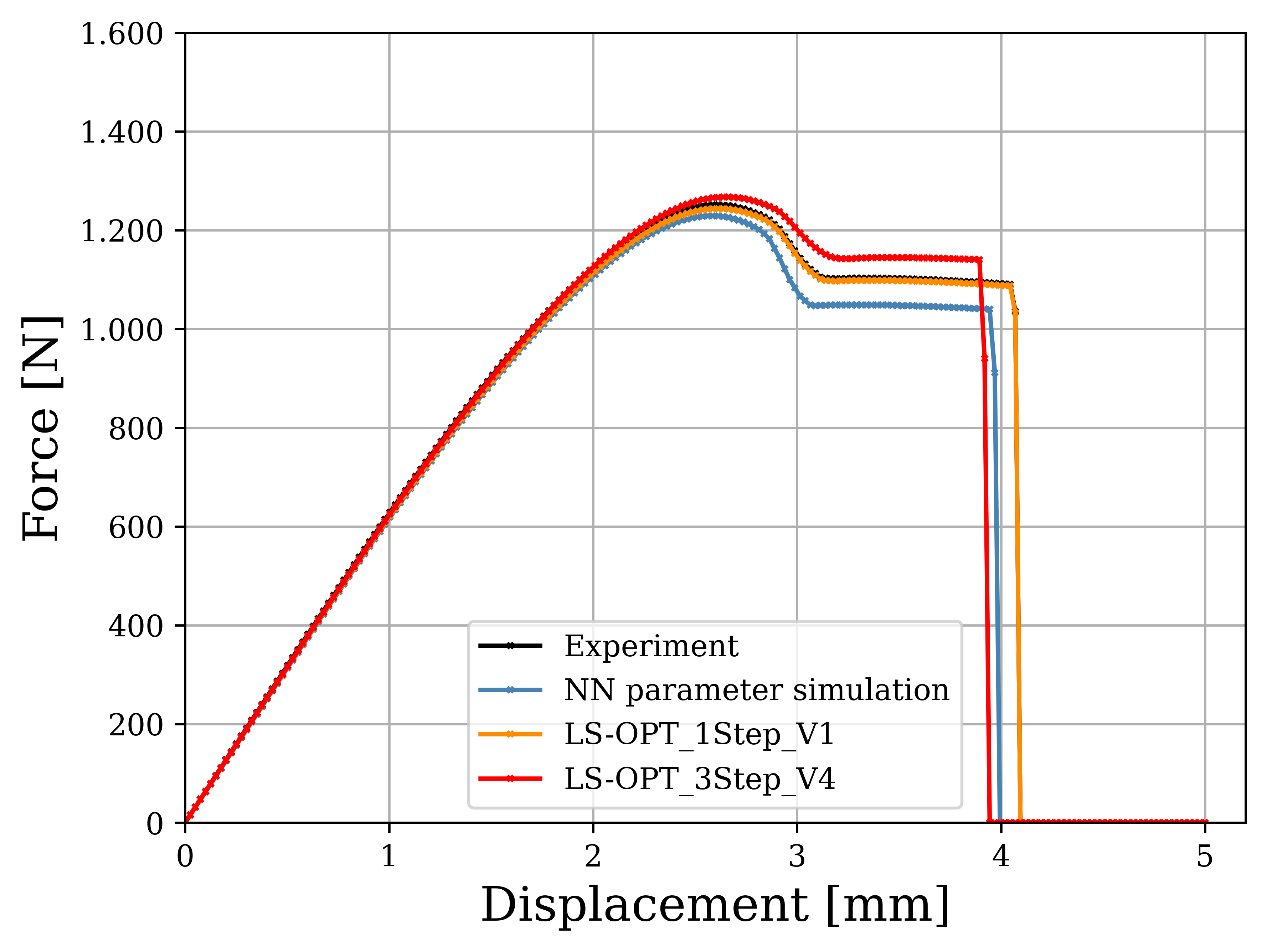

Supplement: Supplementary file 1 [file materials-15-00643-s001.zip › Supplementary_Material/SOC_NN_Pred_LSOPT_Complete/NN_Run_6/FD_Comparison_Tensile_Test_V1.png]

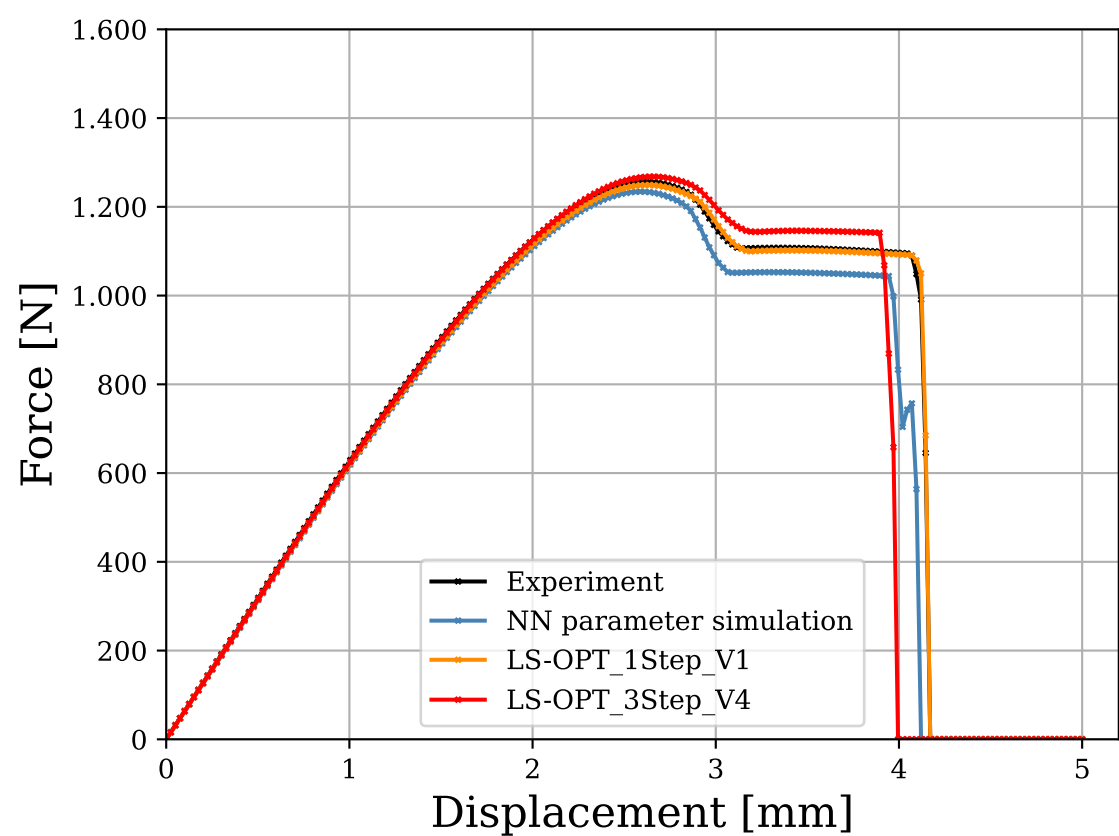

Supplement: Supplementary file 1 [file materials-15-00643-s001.zip › Supplementary_Material/SOC_NN_Pred_LSOPT_Complete/NN_Run_6/FD_Comparison_Tensile_Test_V2.pdf]

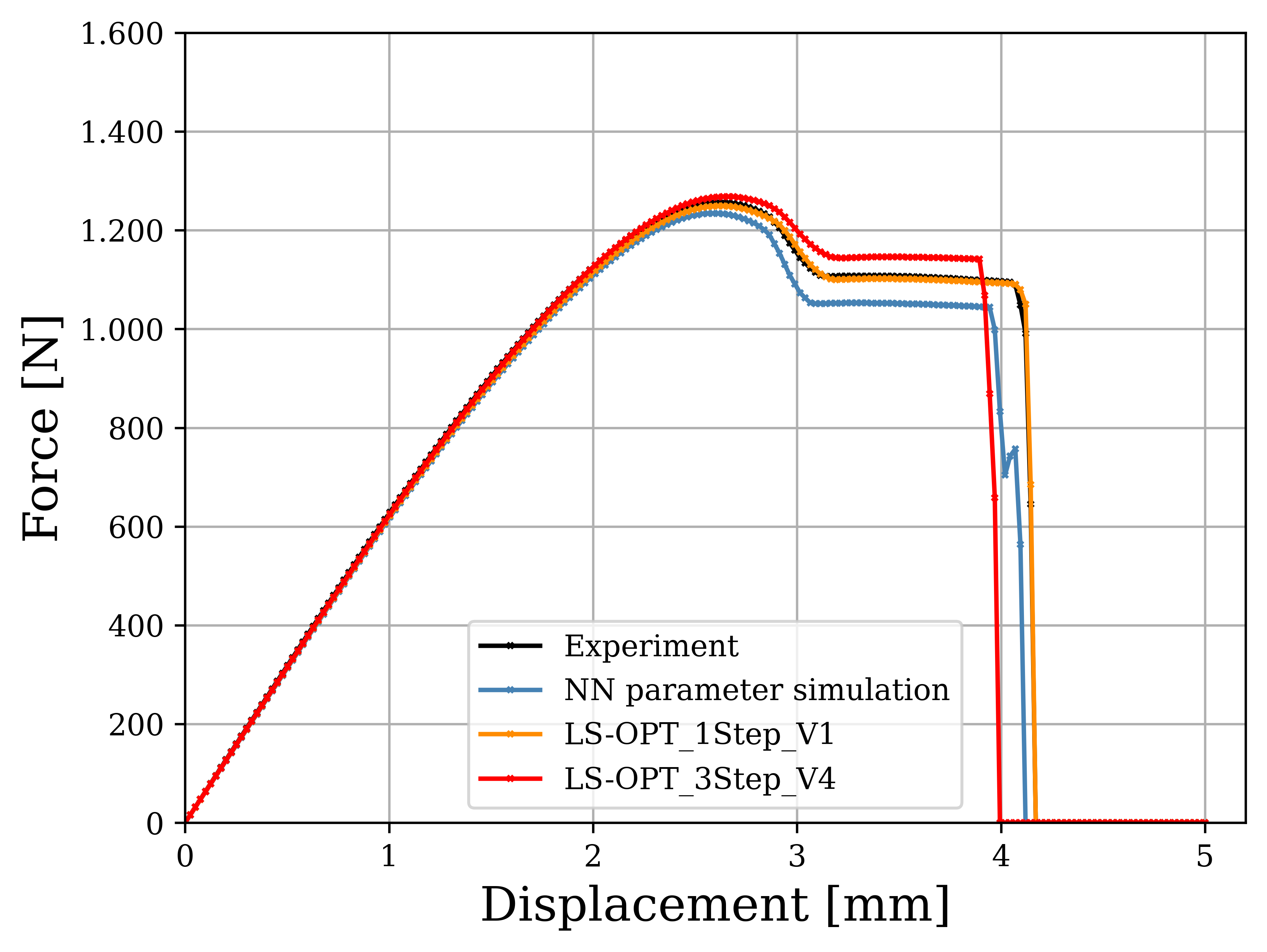

Supplement: Supplementary file 1 [file materials-15-00643-s001.zip › Supplementary_Material/SOC_NN_Pred_LSOPT_Complete/NN_Run_6/FD_Comparison_Tensile_Test_V2.png]

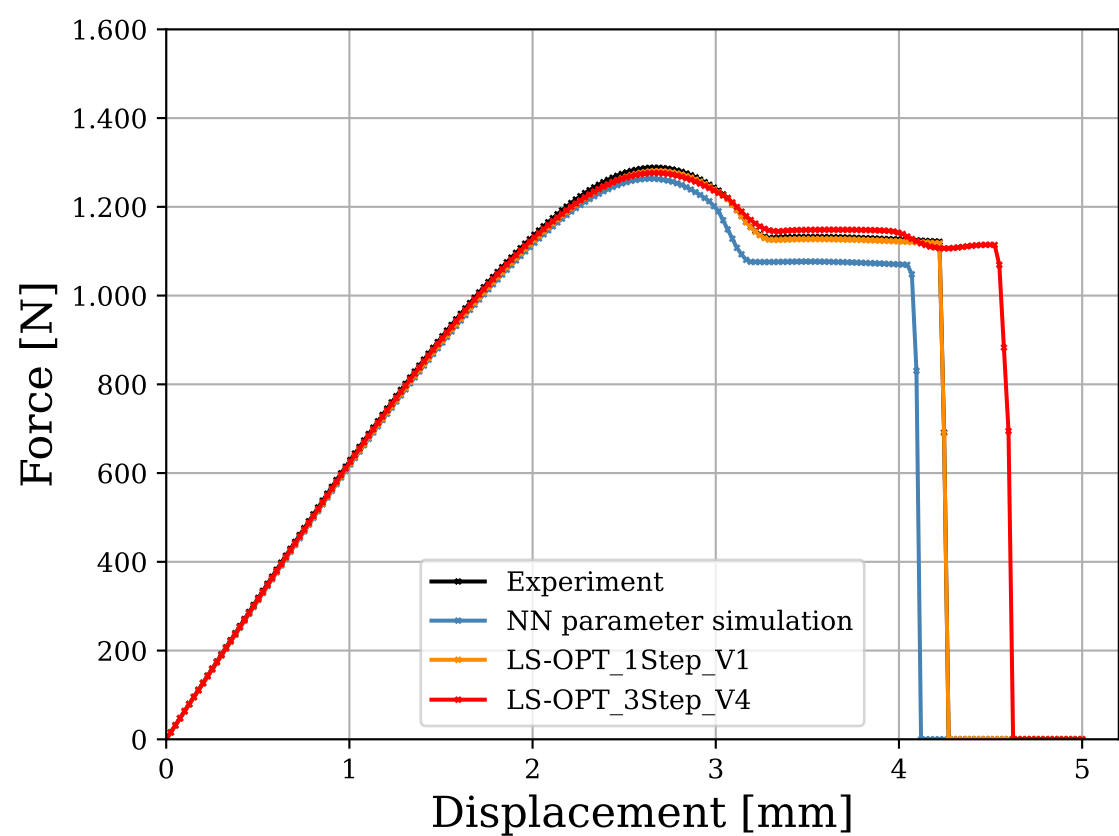

Supplement: Supplementary file 1 [file materials-15-00643-s001.zip › Supplementary_Material/SOC_NN_Pred_LSOPT_Complete/NN_Run_6/FD_Comparison_Tensile_Test_V3.pdf]

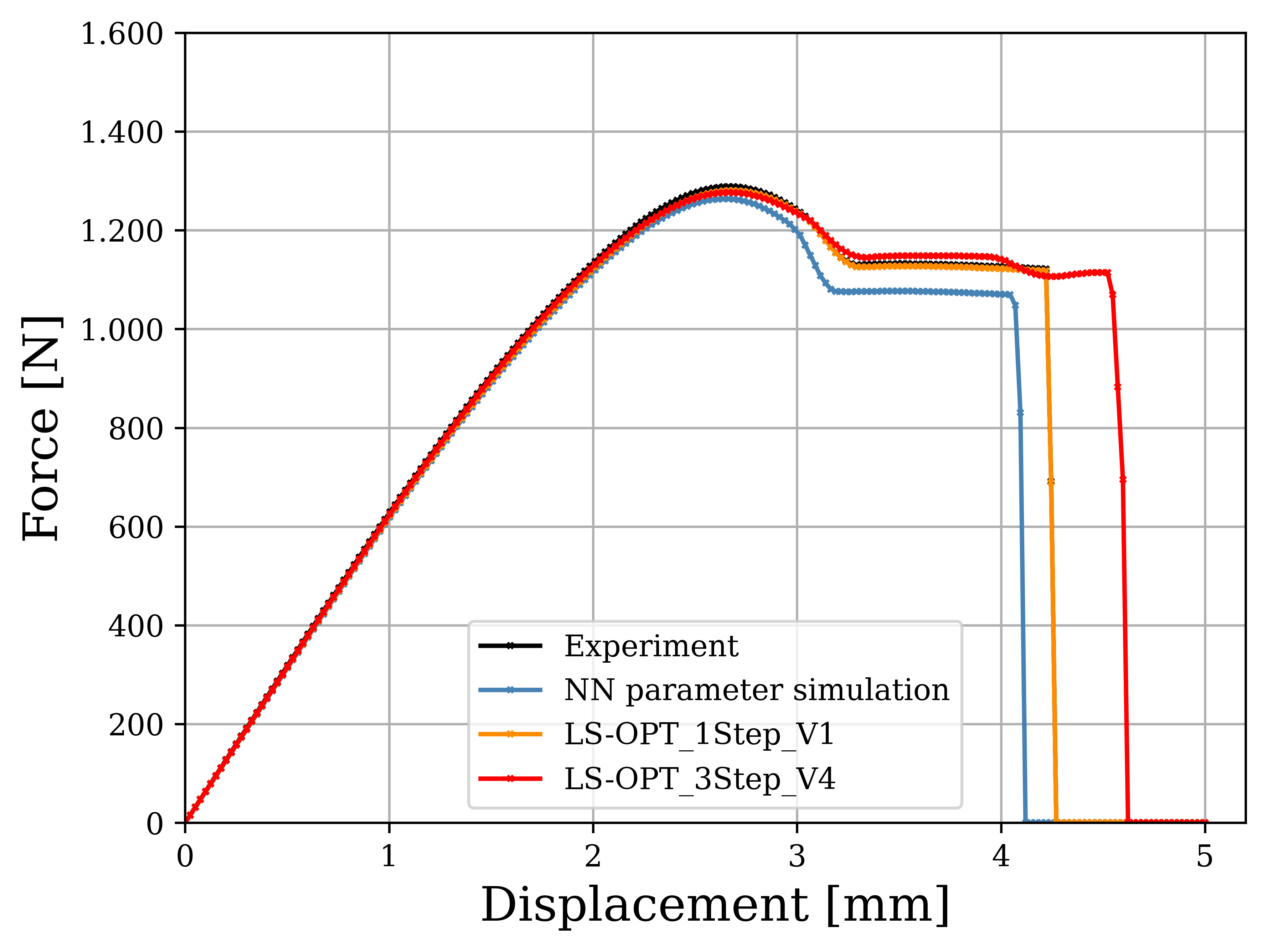

Supplement: Supplementary file 1 [file materials-15-00643-s001.zip › Supplementary_Material/SOC_NN_Pred_LSOPT_Complete/NN_Run_6/FD_Comparison_Tensile_Test_V3.png]

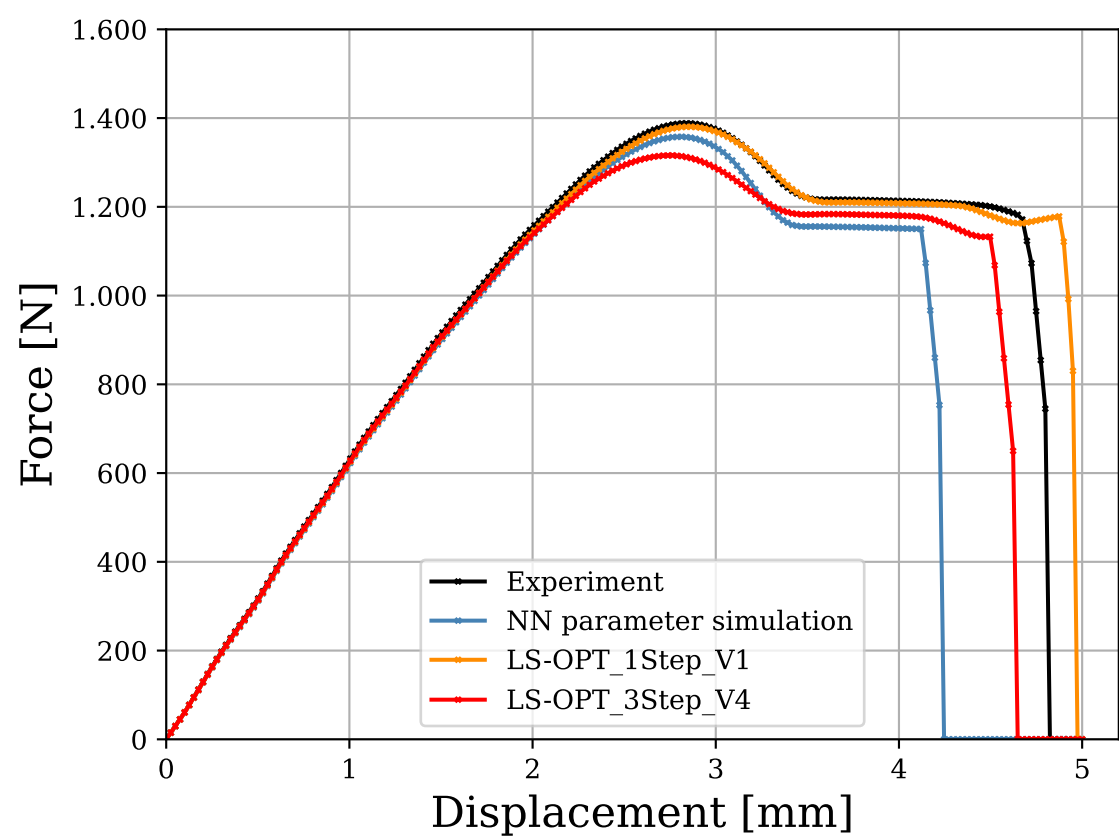

Supplement: Supplementary file 1 [file materials-15-00643-s001.zip › Supplementary_Material/SOC_NN_Pred_LSOPT_Complete/NN_Run_6/FD_Comparison_Tensile_Test_V4.pdf]

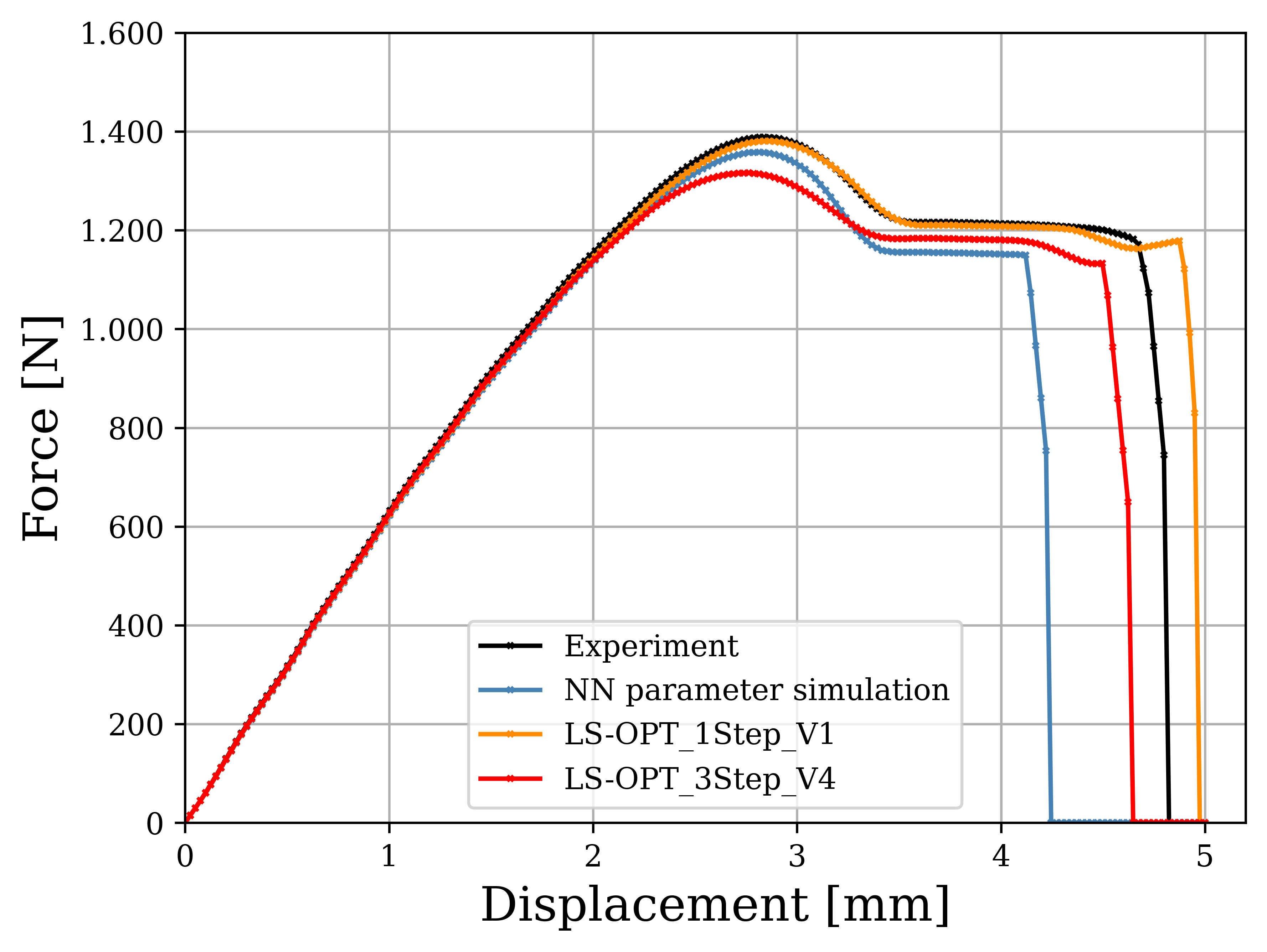

Supplement: Supplementary file 1 [file materials-15-00643-s001.zip › Supplementary_Material/SOC_NN_Pred_LSOPT_Complete/NN_Run_6/FD_Comparison_Tensile_Test_V4.png]

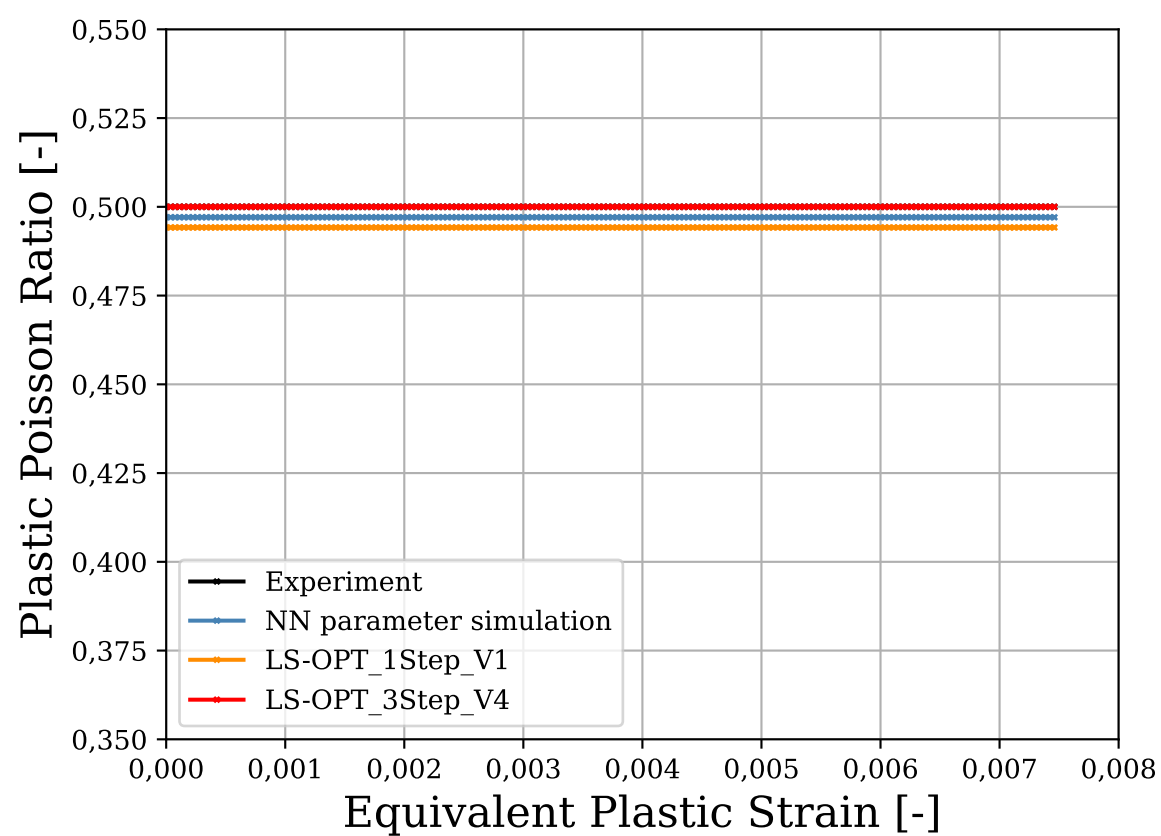

Supplement: Supplementary file 1 [file materials-15-00643-s001.zip › Supplementary_Material/SOC_NN_Pred_LSOPT_Complete/NN_Run_6/PE_Comparison_Compression_Test.pdf]

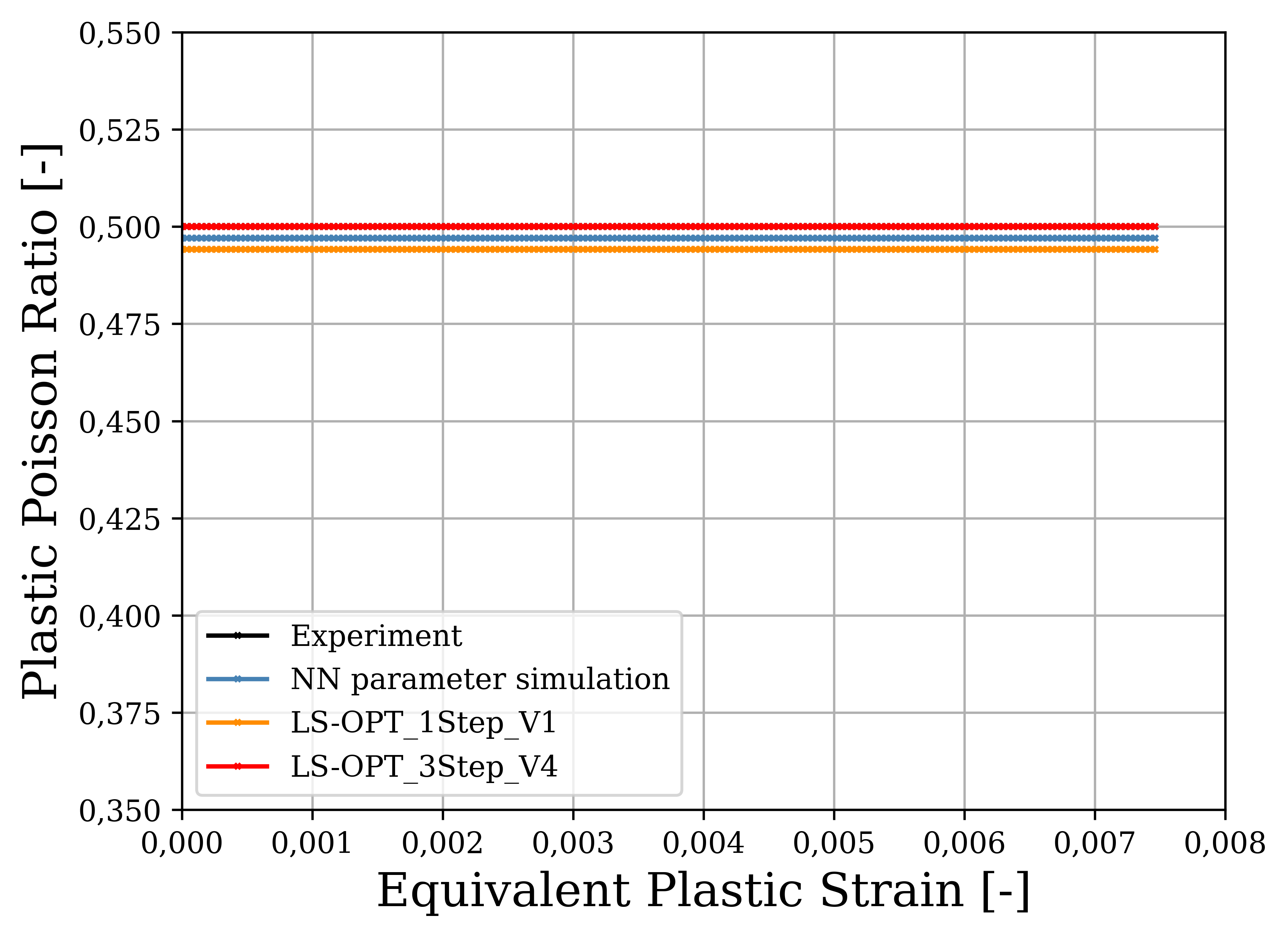

Supplement: Supplementary file 1 [file materials-15-00643-s001.zip › Supplementary_Material/SOC_NN_Pred_LSOPT_Complete/NN_Run_6/PE_Comparison_Compression_Test.png]

Plastic Poisson Ratio [-]

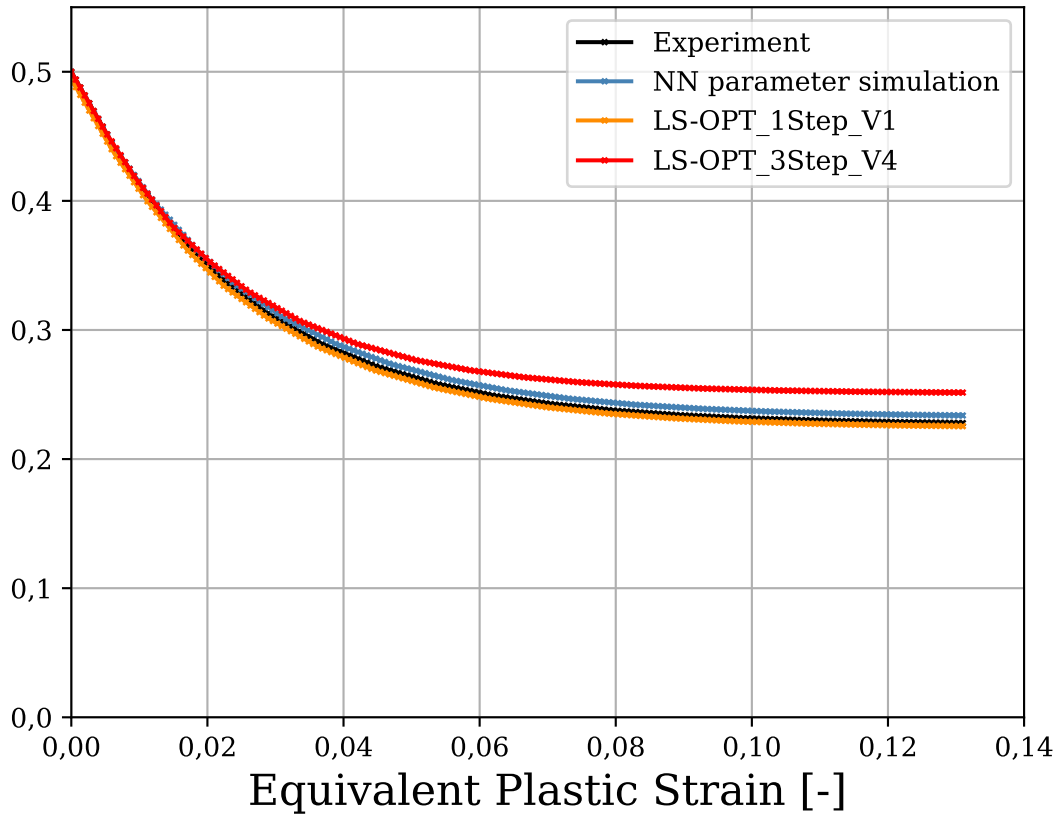

Supplement: Supplementary file 1 [file materials-15-00643-s001.zip › Supplementary_Material/SOC_NN_Pred_LSOPT_Complete/NN_Run_6/PE_Comparison_Punch_Test.pdf]

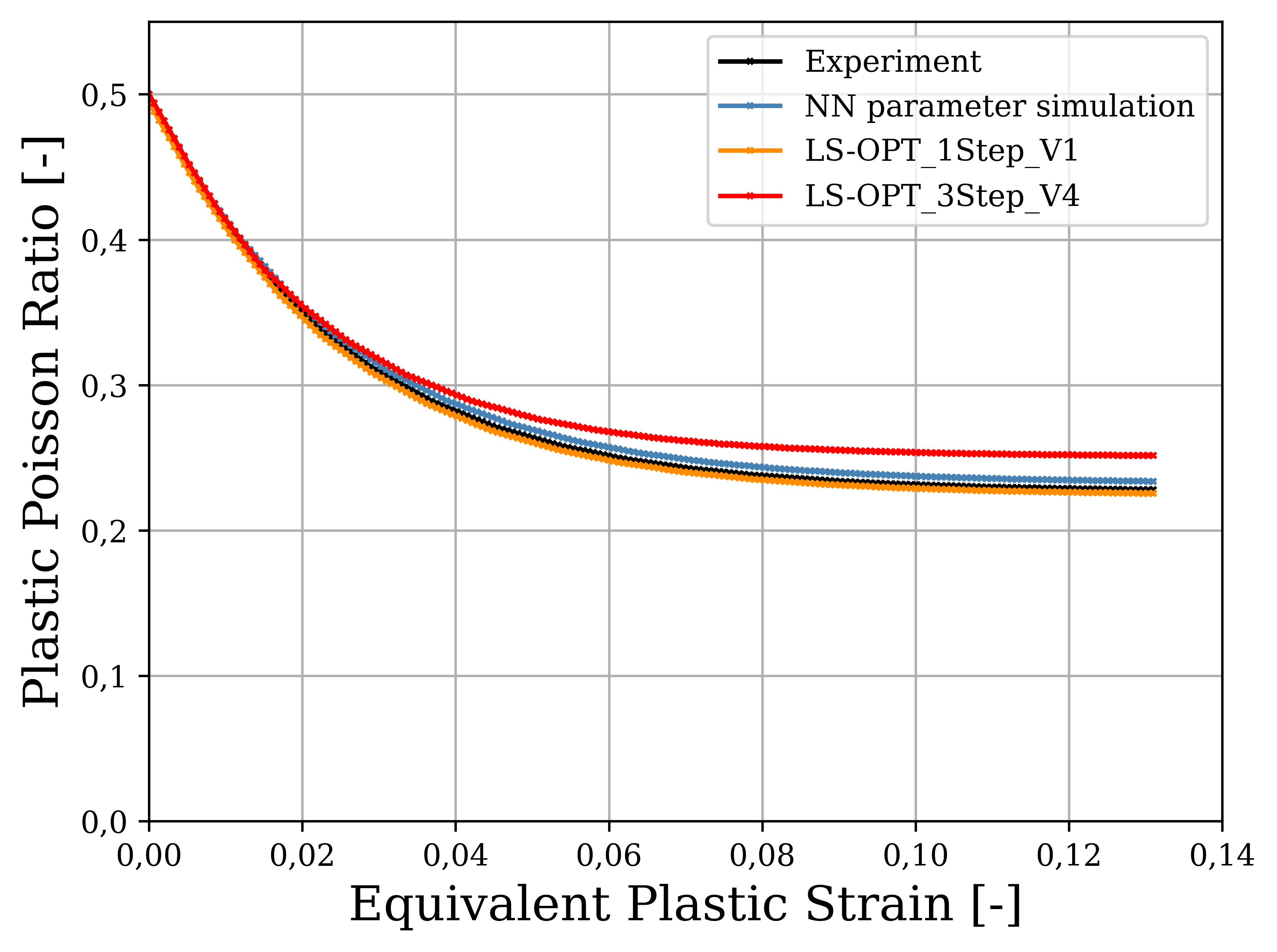

Supplement: Supplementary file 1 [file materials-15-00643-s001.zip › Supplementary_Material/SOC_NN_Pred_LSOPT_Complete/NN_Run_6/PE_Comparison_Punch_Test.png]

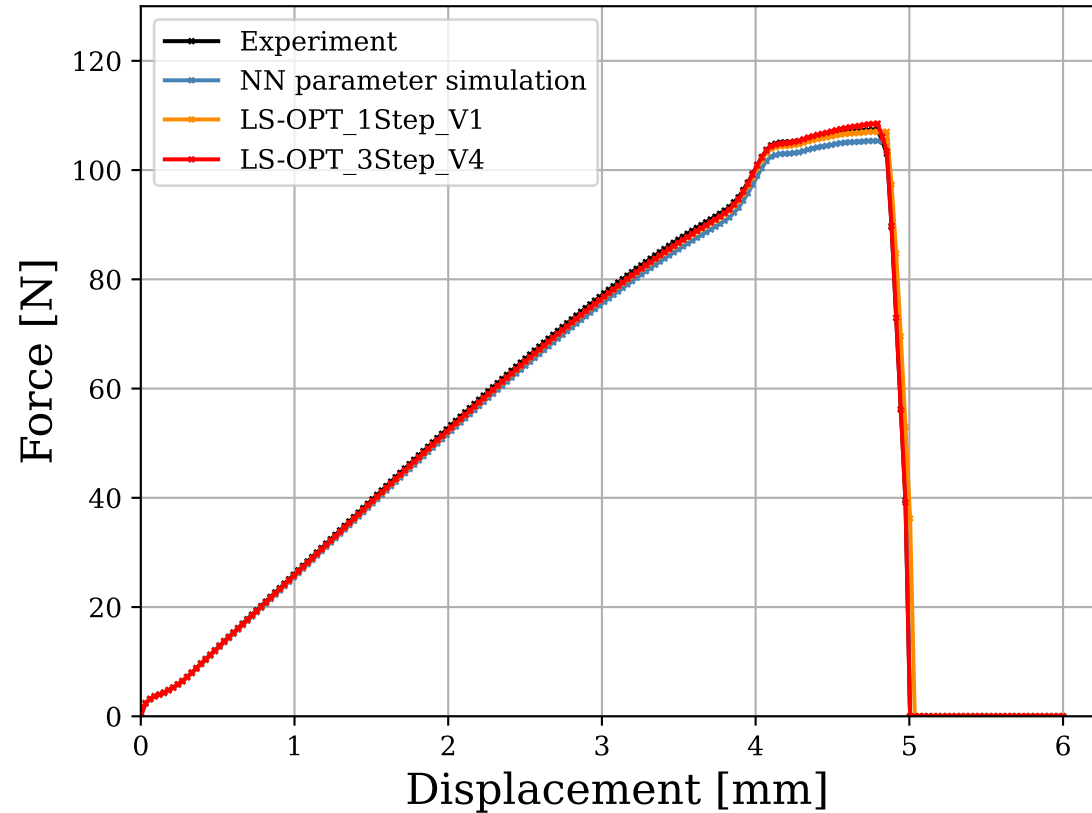

Supplement: Supplementary file 1 [file materials-15-00643-s001.zip › Supplementary_Material/SOC_NN_Pred_LSOPT_Complete/NN_Run_7/FD_Comparison_Bending_Test.pdf]

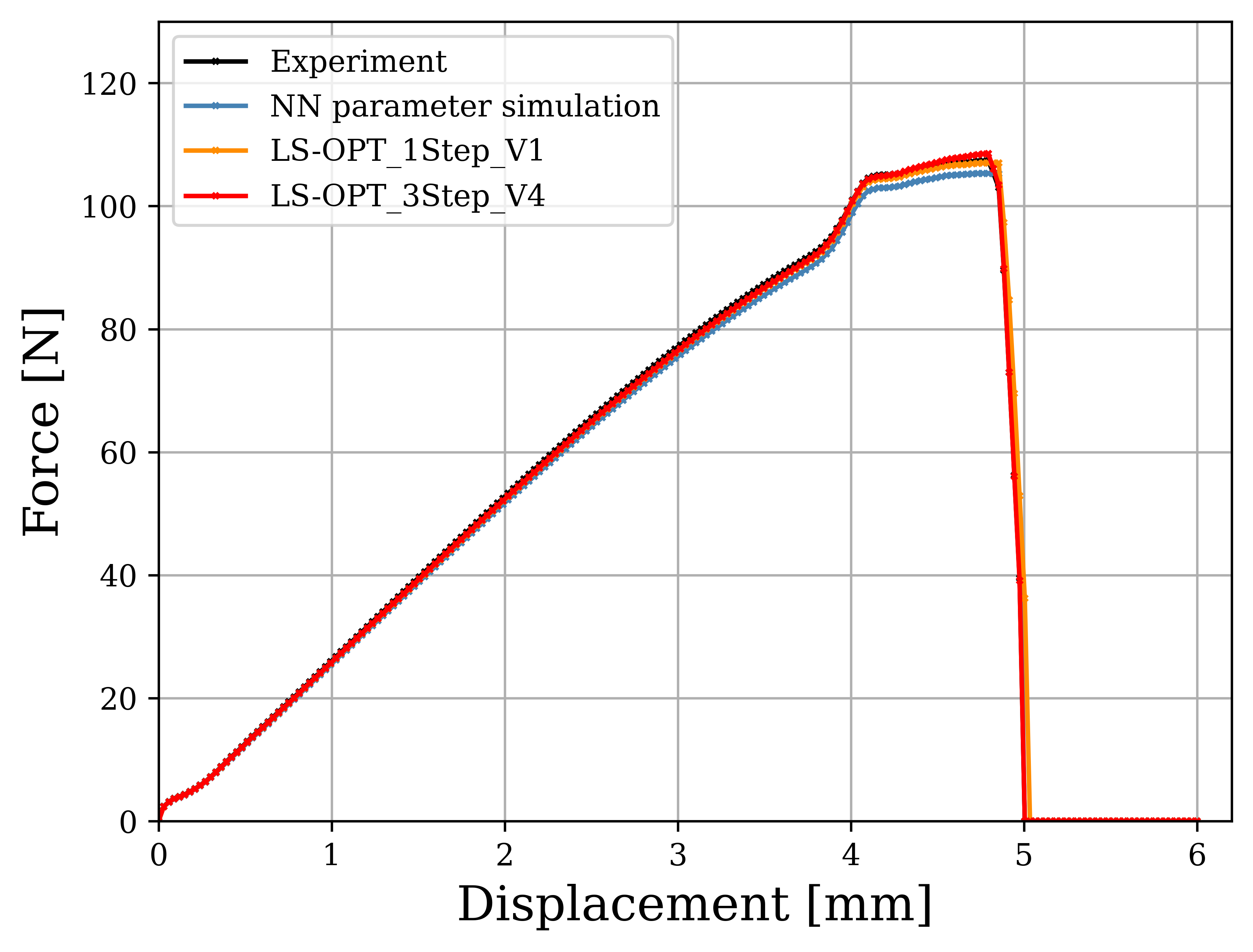

Supplement: Supplementary file 1 [file materials-15-00643-s001.zip › Supplementary_Material/SOC_NN_Pred_LSOPT_Complete/NN_Run_7/FD_Comparison_Bending_Test.png]

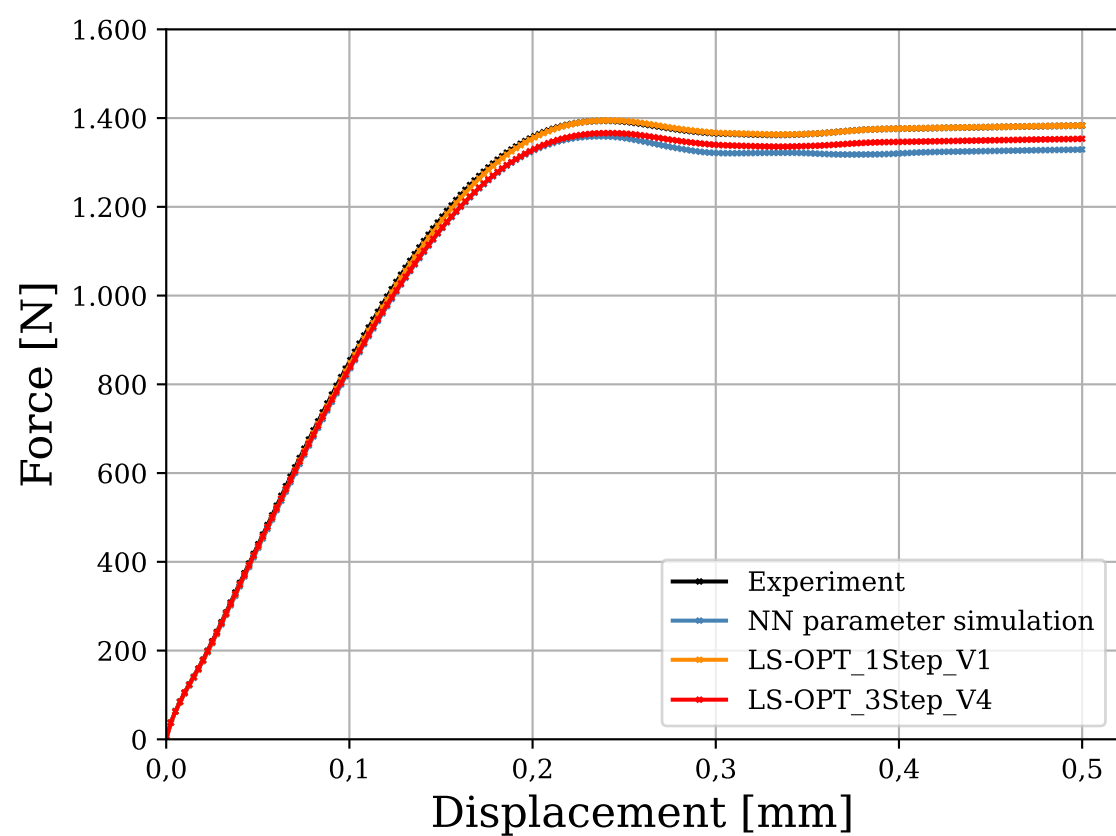

Supplement: Supplementary file 1 [file materials-15-00643-s001.zip › Supplementary_Material/SOC_NN_Pred_LSOPT_Complete/NN_Run_7/FD_Comparison_Compression_Test.pdf]

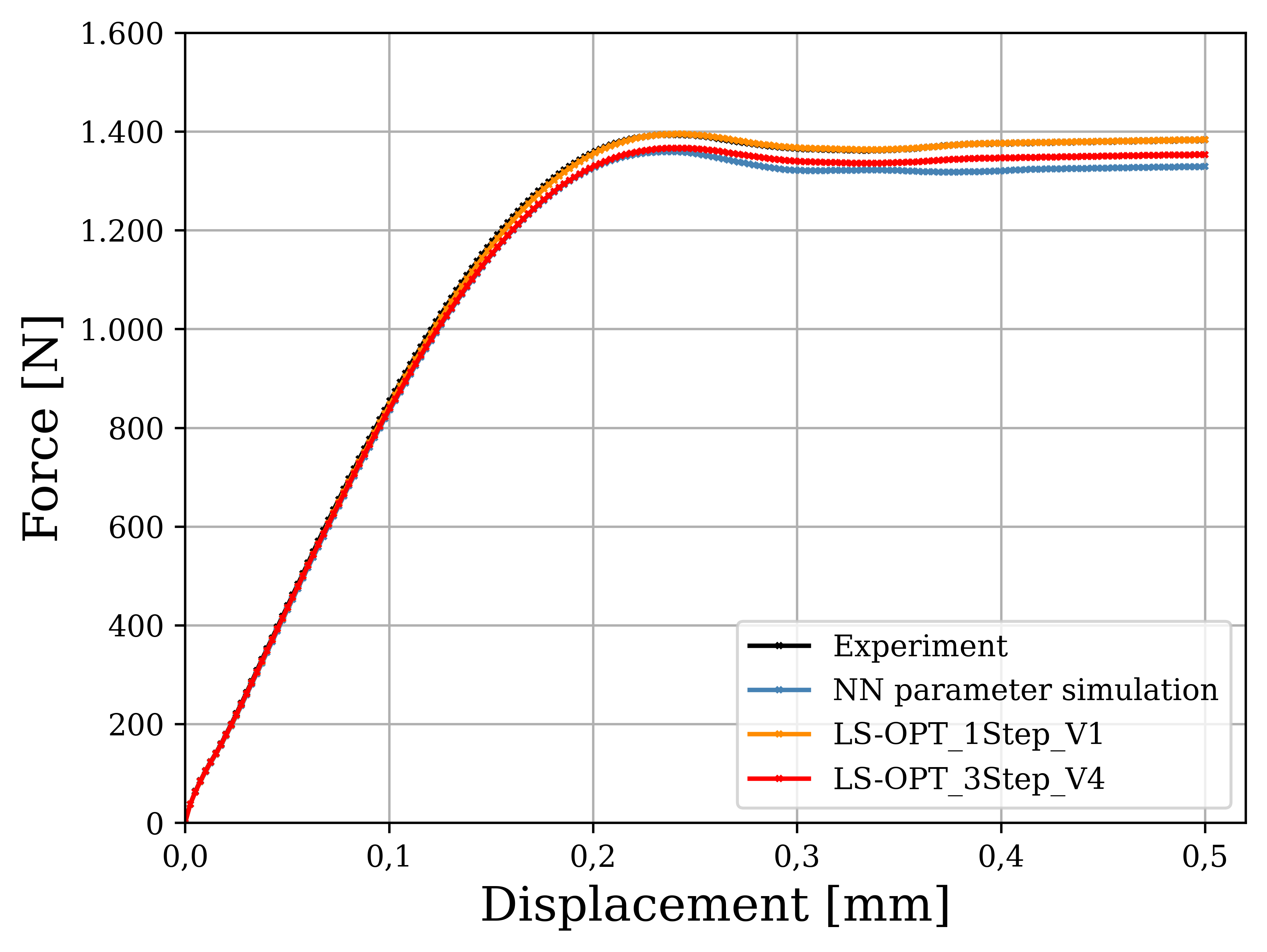

Supplement: Supplementary file 1 [file materials-15-00643-s001.zip › Supplementary_Material/SOC_NN_Pred_LSOPT_Complete/NN_Run_7/FD_Comparison_Compression_Test.png]

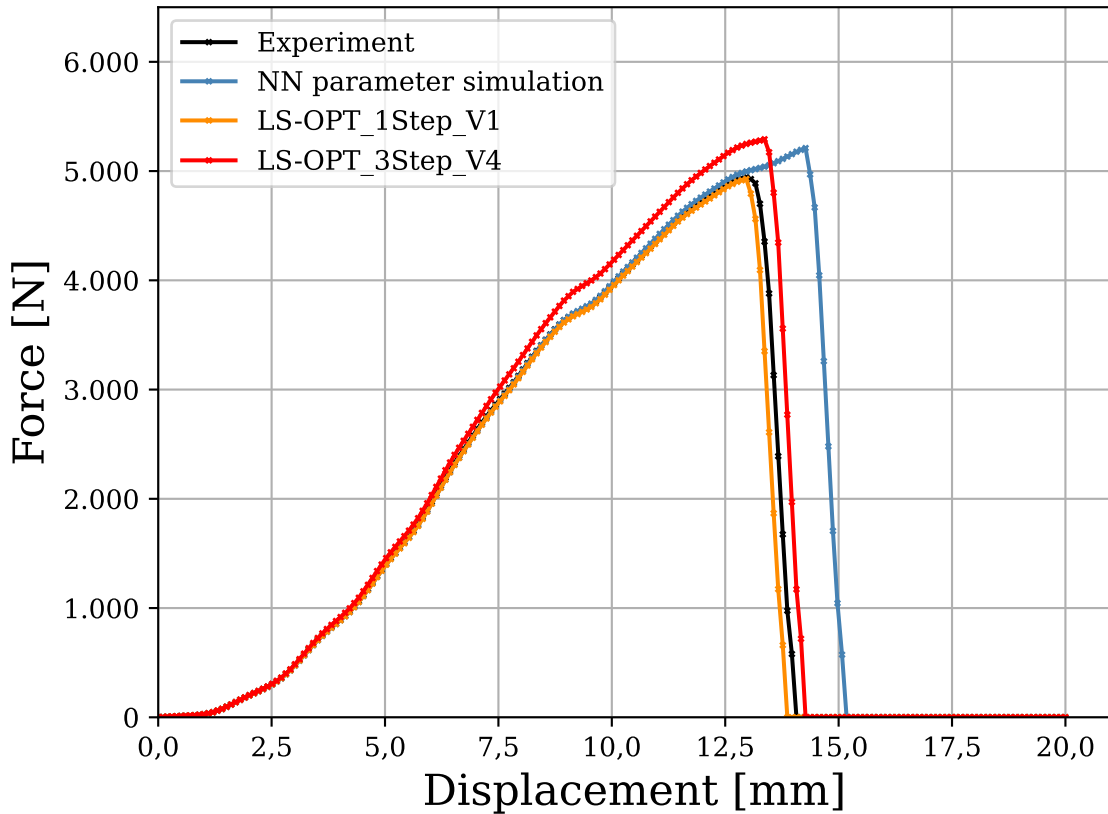

Supplement: Supplementary file 1 [file materials-15-00643-s001.zip › Supplementary_Material/SOC_NN_Pred_LSOPT_Complete/NN_Run_7/FD_Comparison_Punch_Test.pdf]

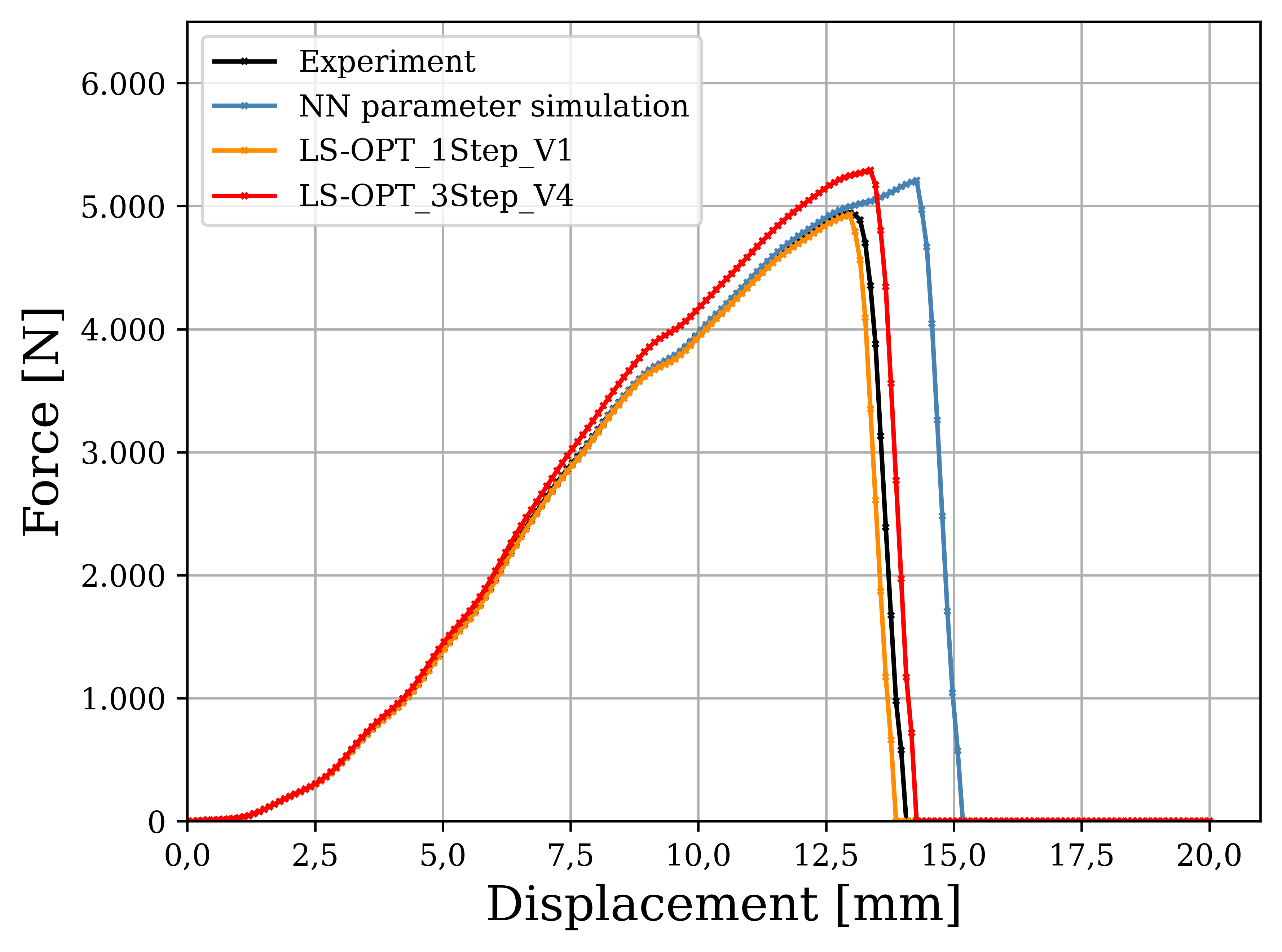

Supplement: Supplementary file 1 [file materials-15-00643-s001.zip › Supplementary_Material/SOC_NN_Pred_LSOPT_Complete/NN_Run_7/FD_Comparison_Punch_Test.png]

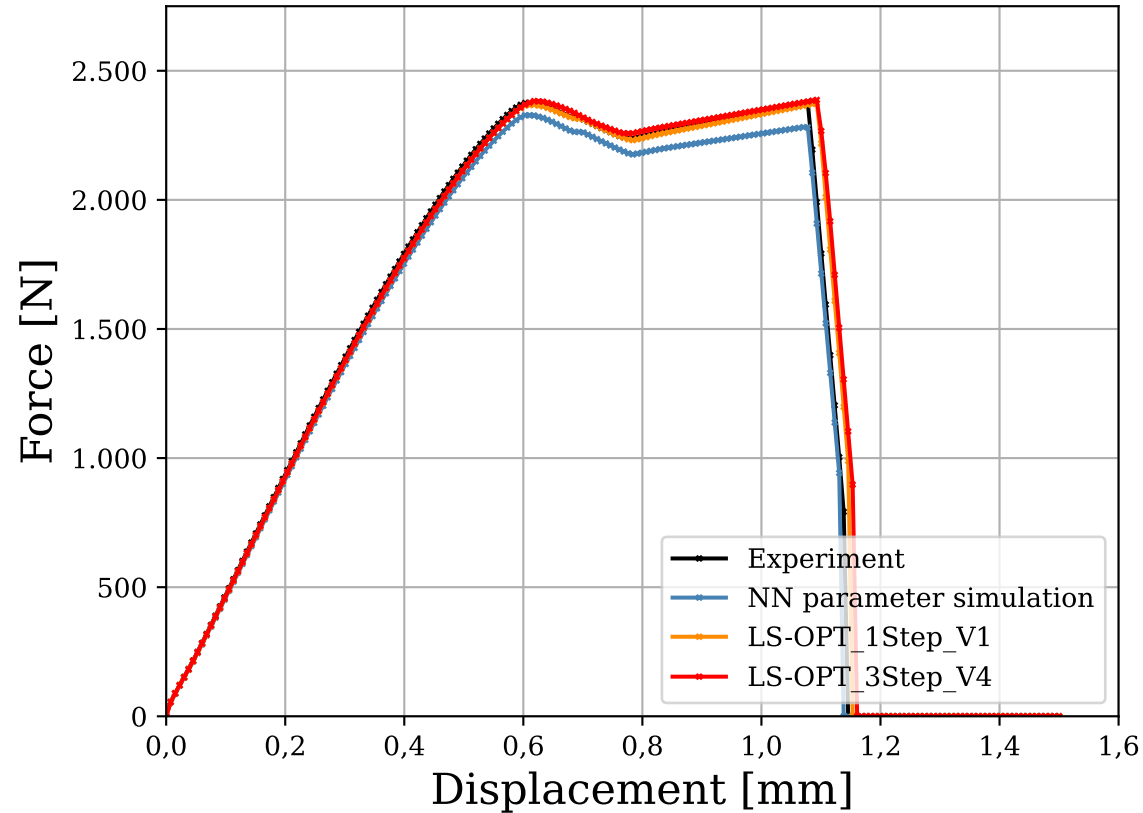

Supplement: Supplementary file 1 [file materials-15-00643-s001.zip › Supplementary_Material/SOC_NN_Pred_LSOPT_Complete/NN_Run_7/FD_Comparison_Shear_ASTM_Test.pdf]

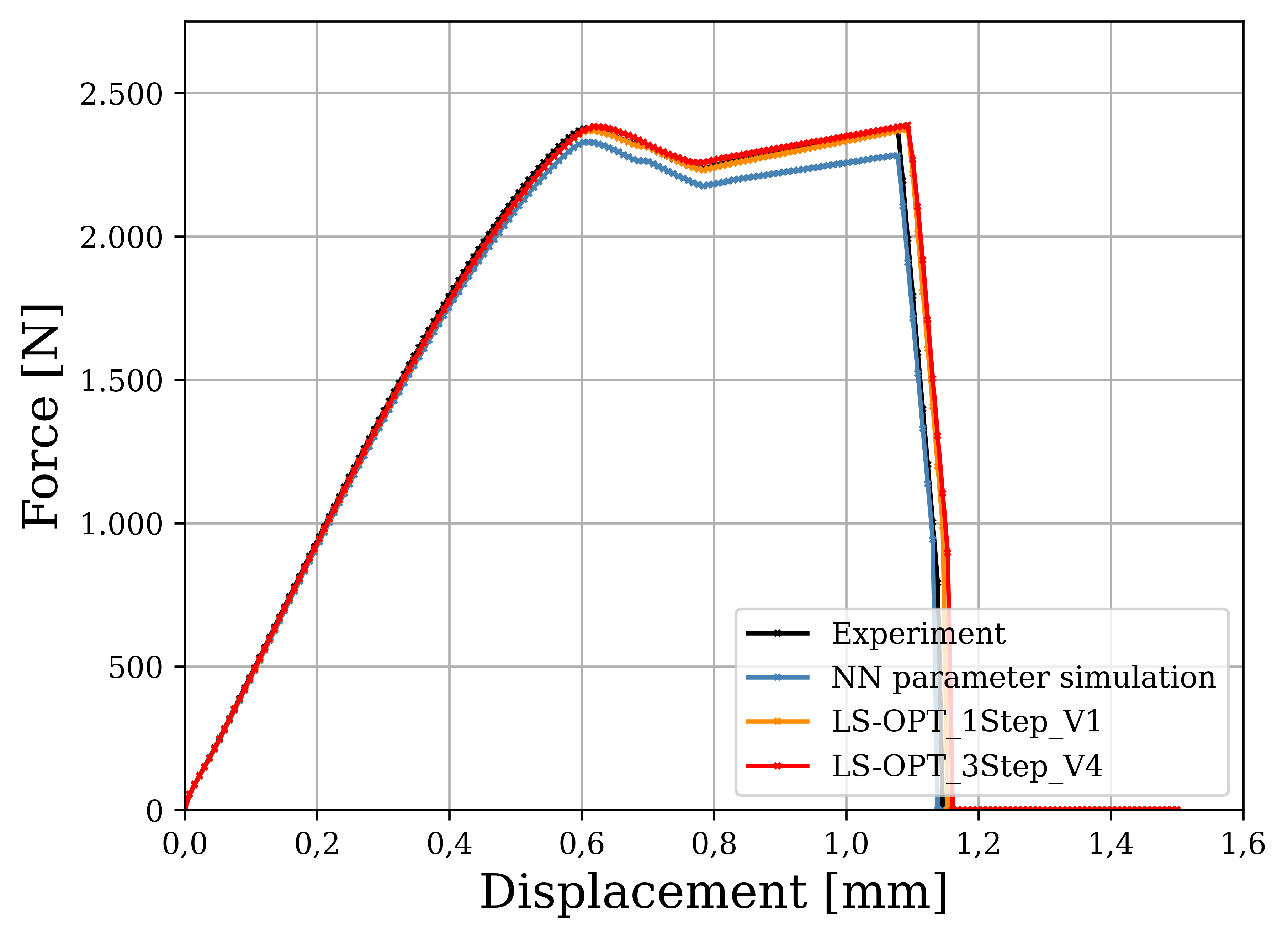

Supplement: Supplementary file 1 [file materials-15-00643-s001.zip › Supplementary_Material/SOC_NN_Pred_LSOPT_Complete/NN_Run_7/FD_Comparison_Shear_ASTM_Test.png]

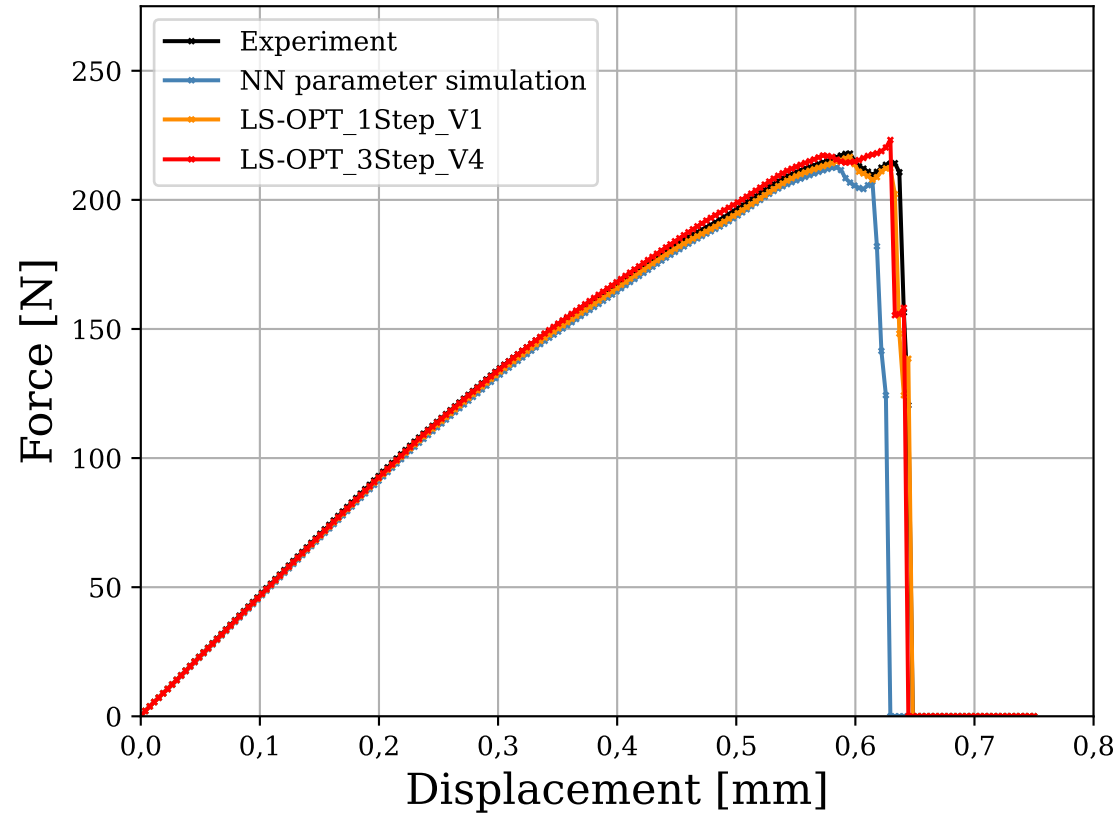

Supplement: Supplementary file 1 [file materials-15-00643-s001.zip › Supplementary_Material/SOC_NN_Pred_LSOPT_Complete/NN_Run_7/FD_Comparison_Shear_Dynamore_Test.pdf]

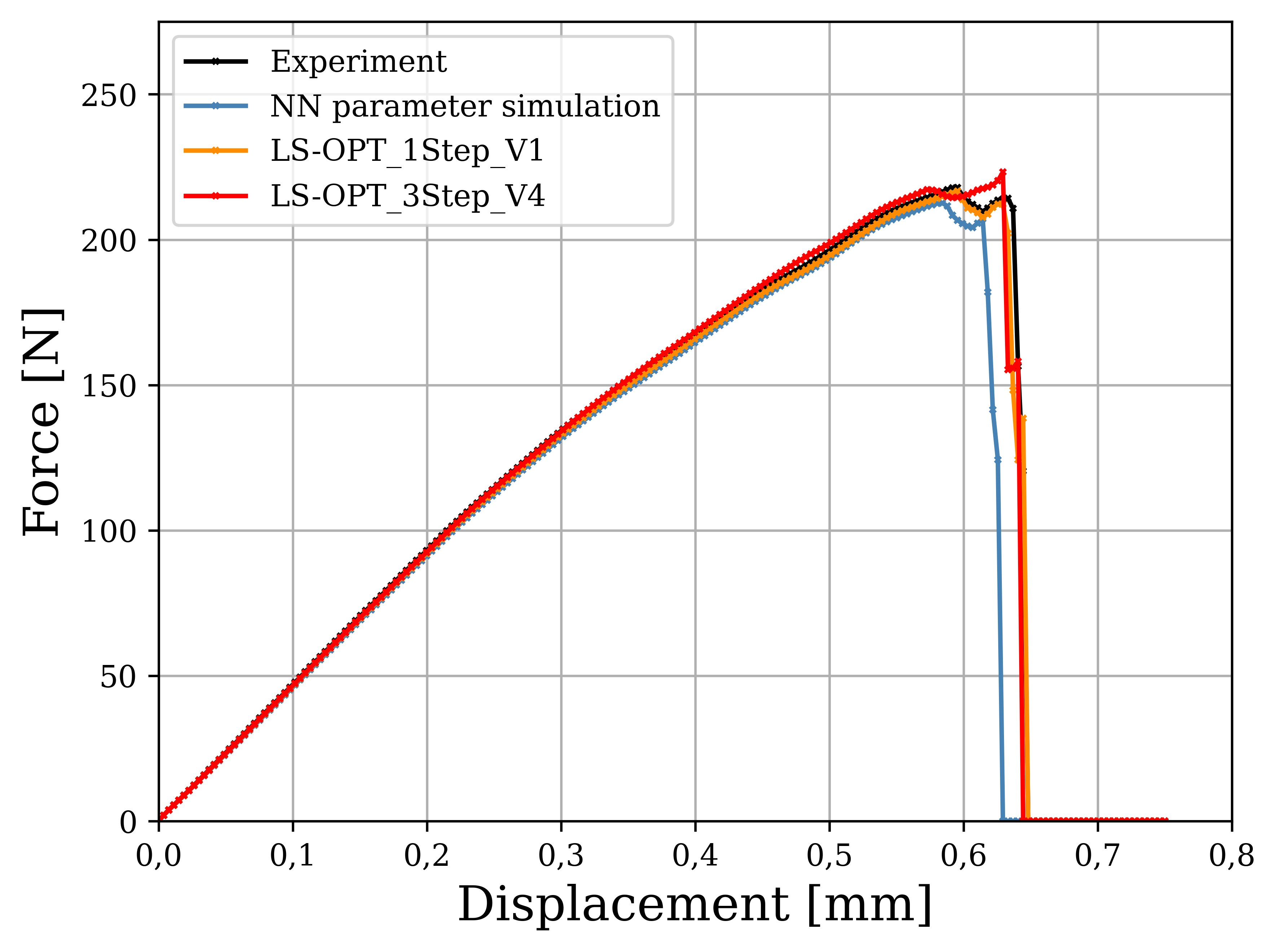

Supplement: Supplementary file 1 [file materials-15-00643-s001.zip › Supplementary_Material/SOC_NN_Pred_LSOPT_Complete/NN_Run_7/FD_Comparison_Shear_Dynamore_Test.png]

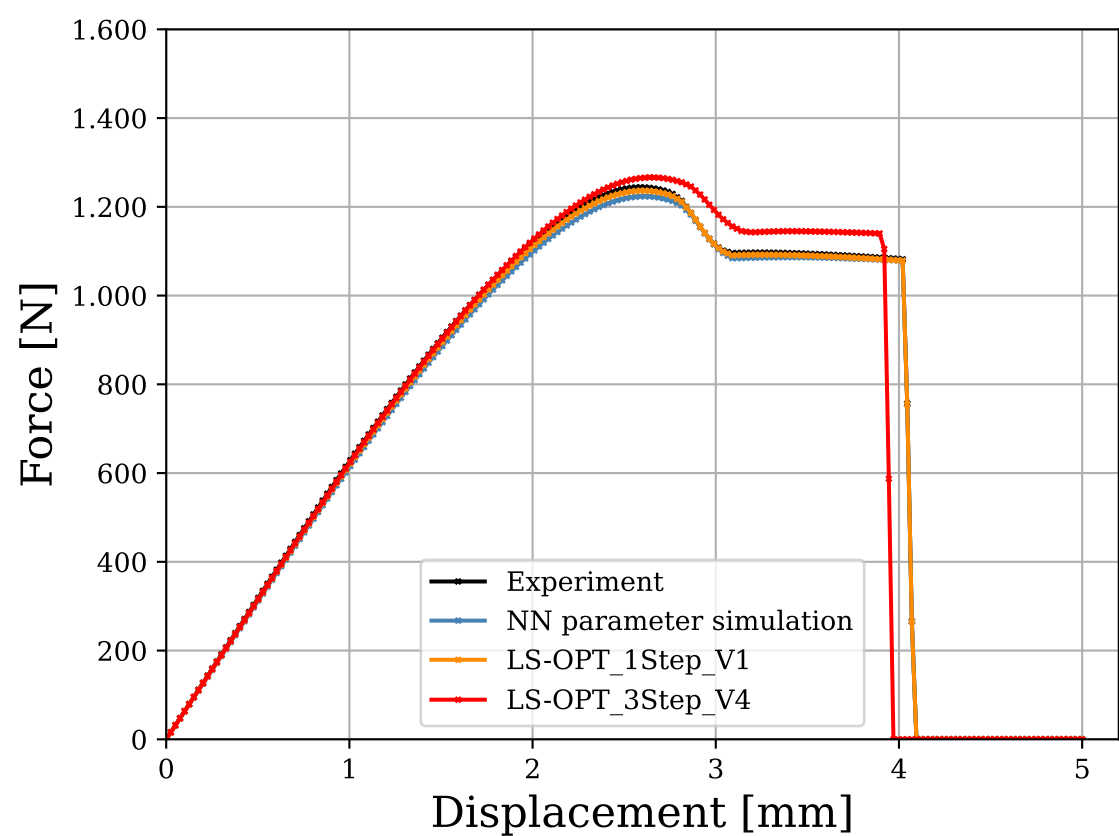

Supplement: Supplementary file 1 [file materials-15-00643-s001.zip › Supplementary_Material/SOC_NN_Pred_LSOPT_Complete/NN_Run_7/FD_Comparison_Tensile_Test.pdf]

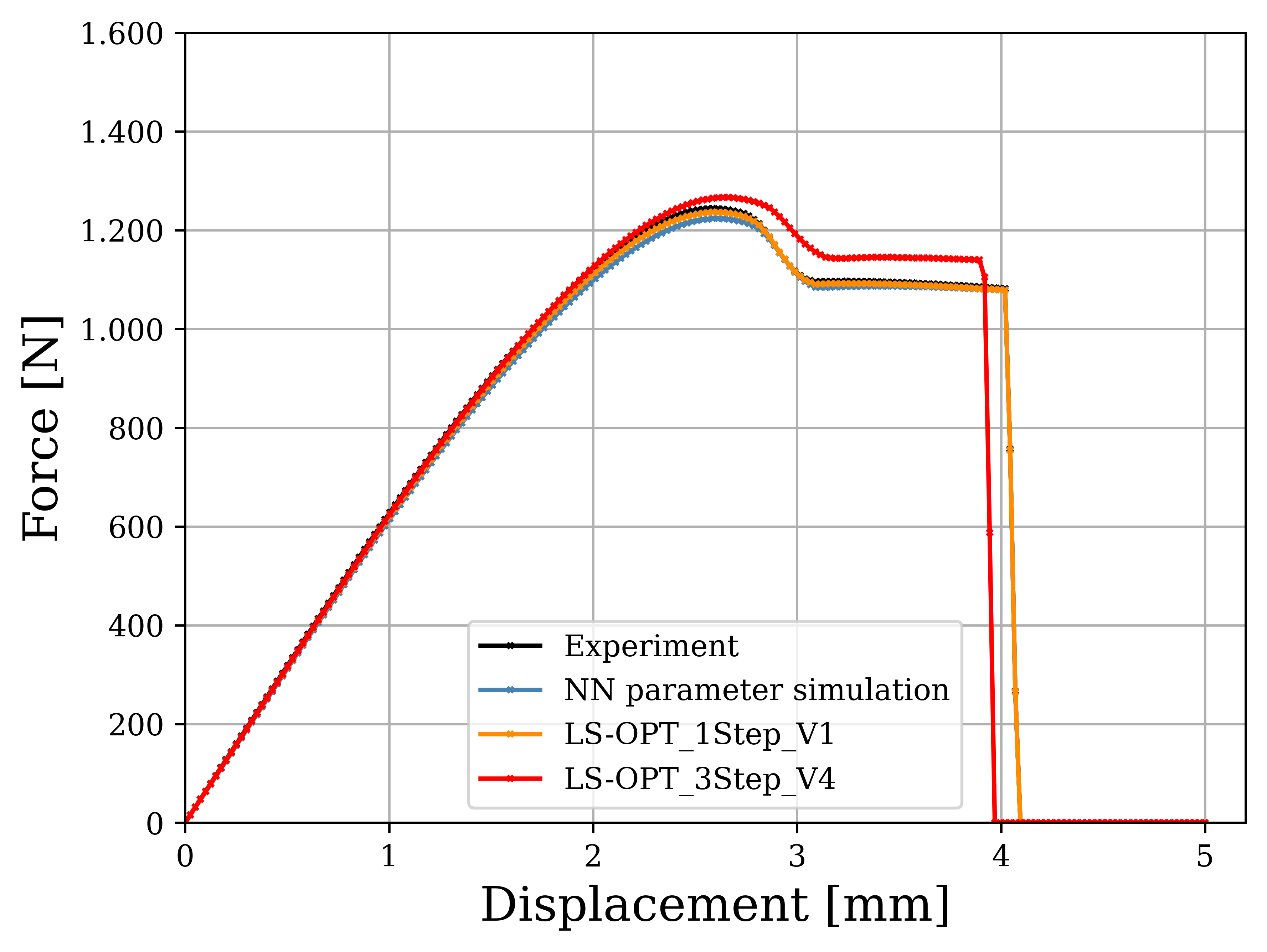

Supplement: Supplementary file 1 [file materials-15-00643-s001.zip › Supplementary_Material/SOC_NN_Pred_LSOPT_Complete/NN_Run_7/FD_Comparison_Tensile_Test.png]

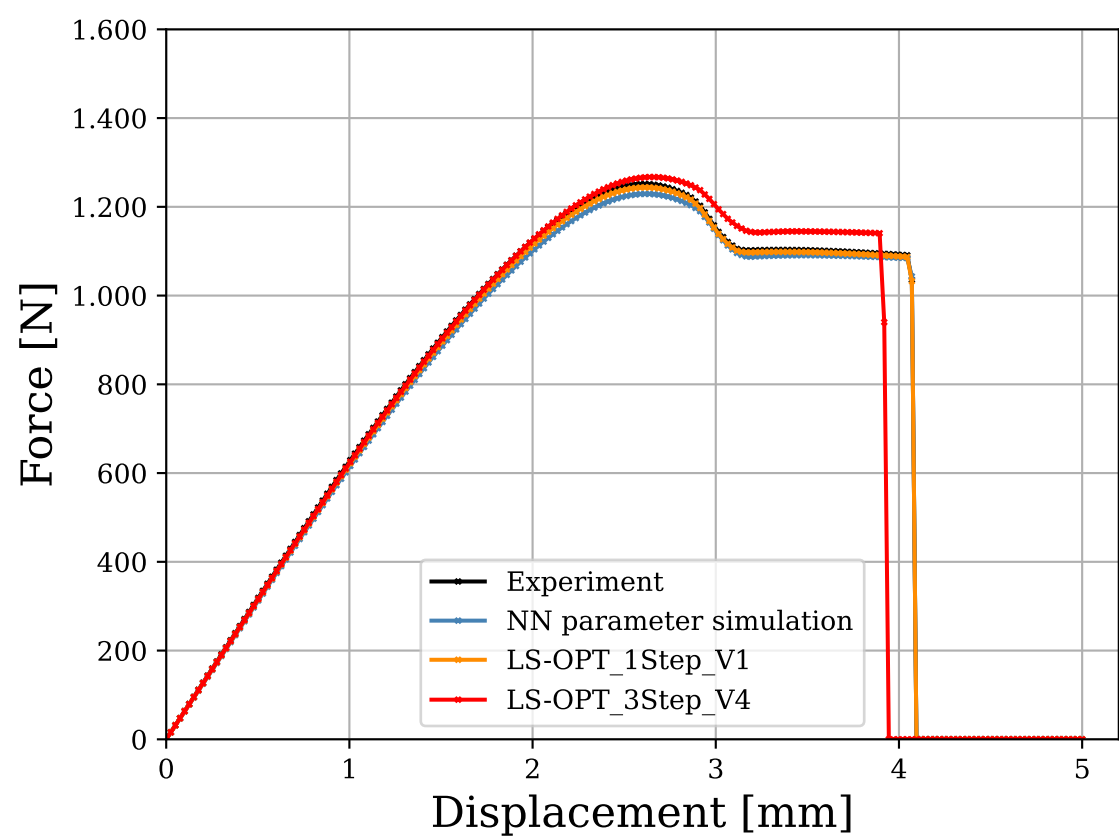

Supplement: Supplementary file 1 [file materials-15-00643-s001.zip › Supplementary_Material/SOC_NN_Pred_LSOPT_Complete/NN_Run_7/FD_Comparison_Tensile_Test_V1.pdf]

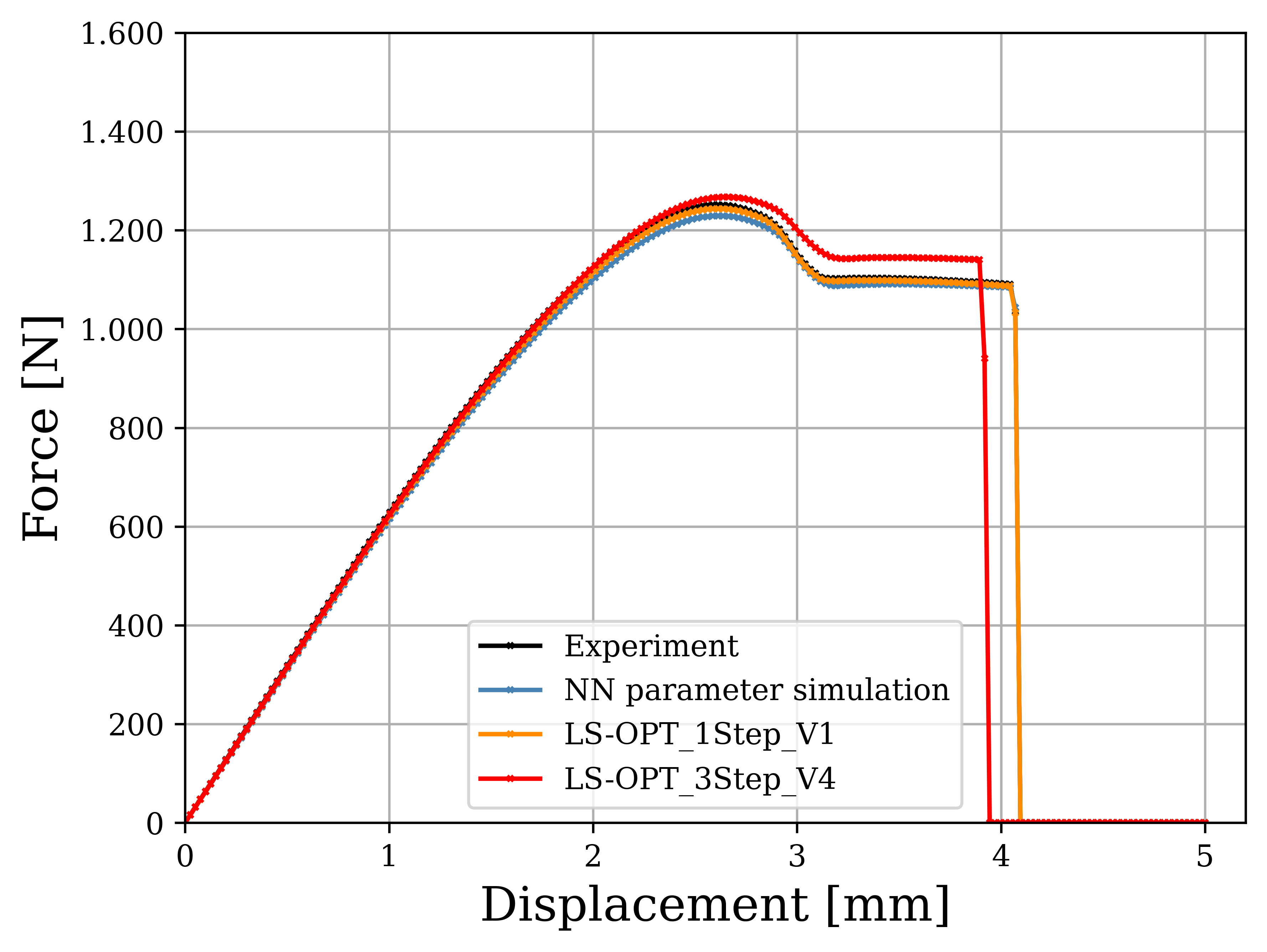

Supplement: Supplementary file 1 [file materials-15-00643-s001.zip › Supplementary_Material/SOC_NN_Pred_LSOPT_Complete/NN_Run_7/FD_Comparison_Tensile_Test_V1.png]

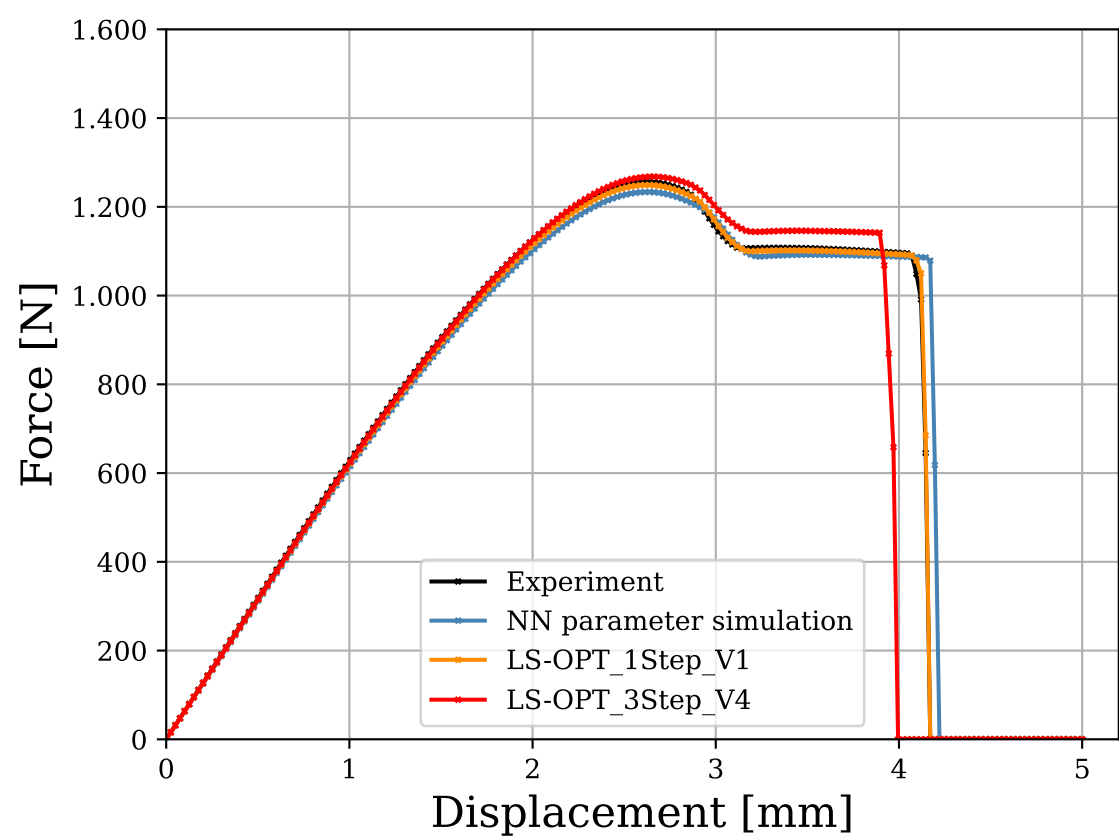

Supplement: Supplementary file 1 [file materials-15-00643-s001.zip › Supplementary_Material/SOC_NN_Pred_LSOPT_Complete/NN_Run_7/FD_Comparison_Tensile_Test_V2.pdf]

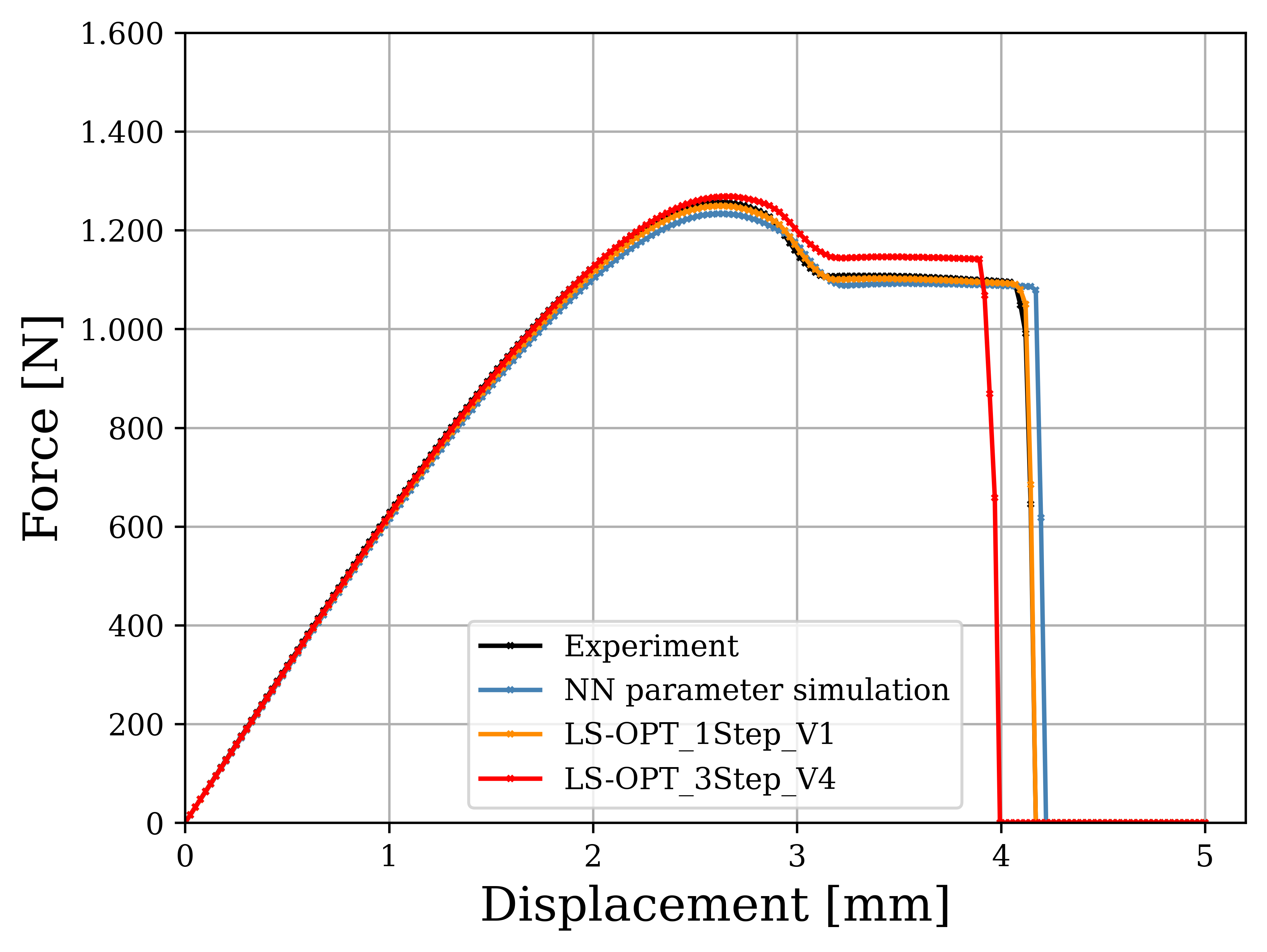

Supplement: Supplementary file 1 [file materials-15-00643-s001.zip › Supplementary_Material/SOC_NN_Pred_LSOPT_Complete/NN_Run_7/FD_Comparison_Tensile_Test_V2.png]

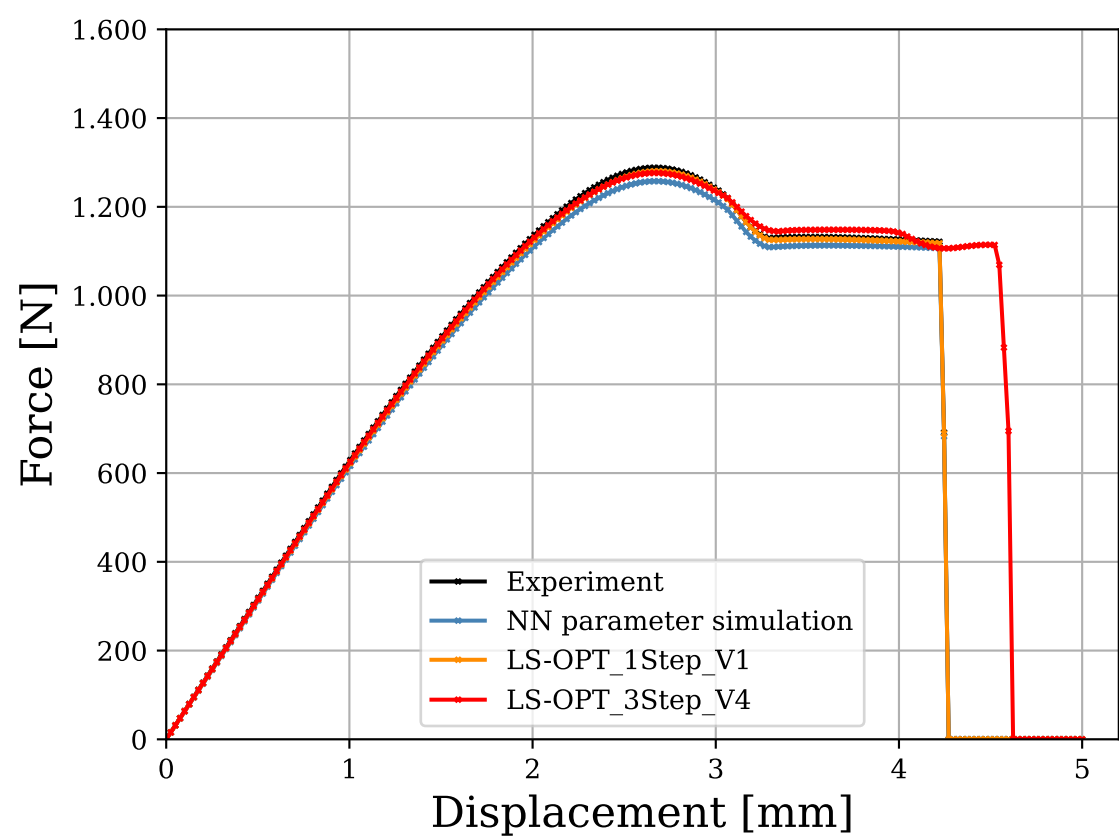

Supplement: Supplementary file 1 [file materials-15-00643-s001.zip › Supplementary_Material/SOC_NN_Pred_LSOPT_Complete/NN_Run_7/FD_Comparison_Tensile_Test_V3.pdf]

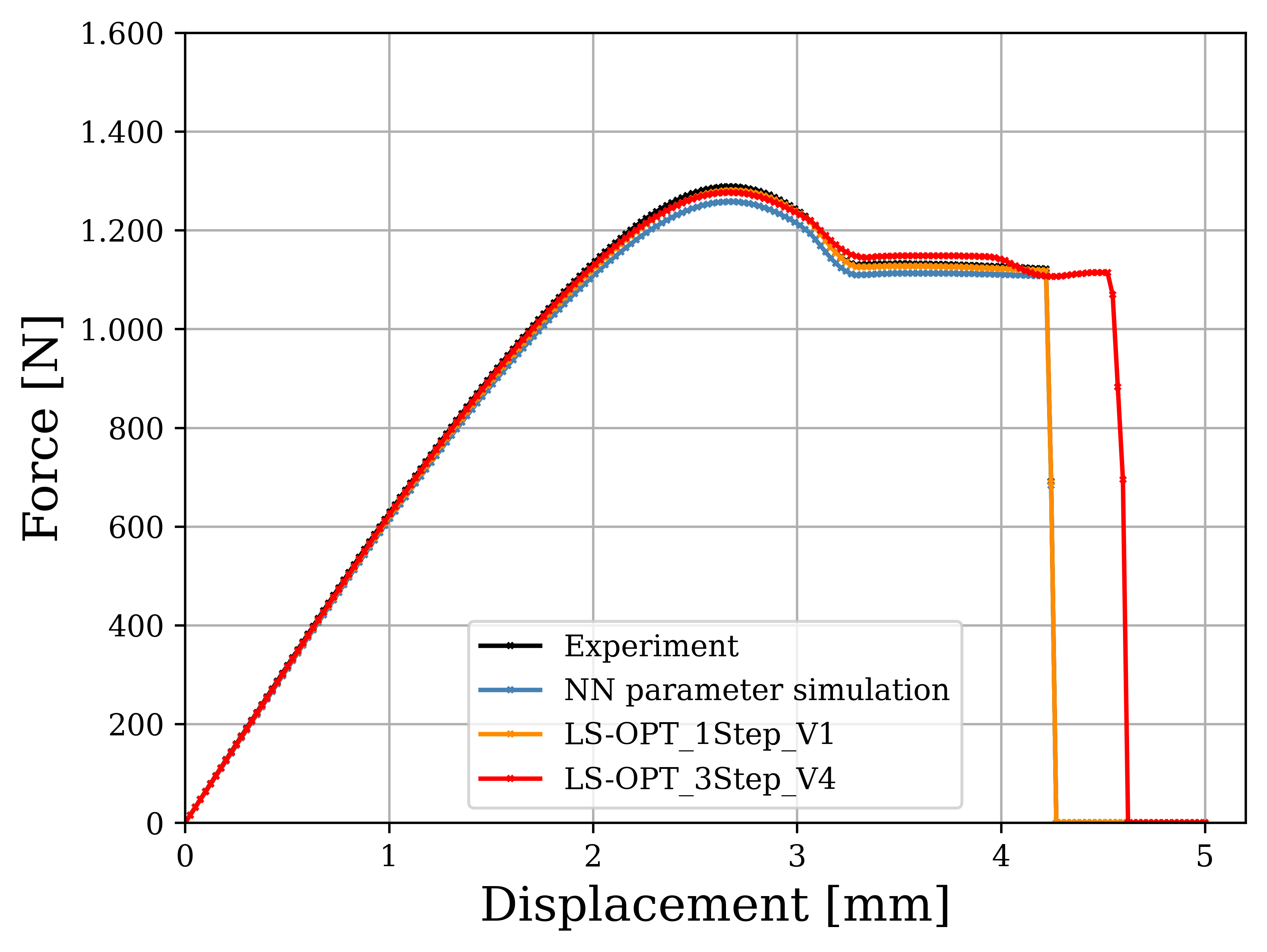

Supplement: Supplementary file 1 [file materials-15-00643-s001.zip › Supplementary_Material/SOC_NN_Pred_LSOPT_Complete/NN_Run_7/FD_Comparison_Tensile_Test_V3.png]

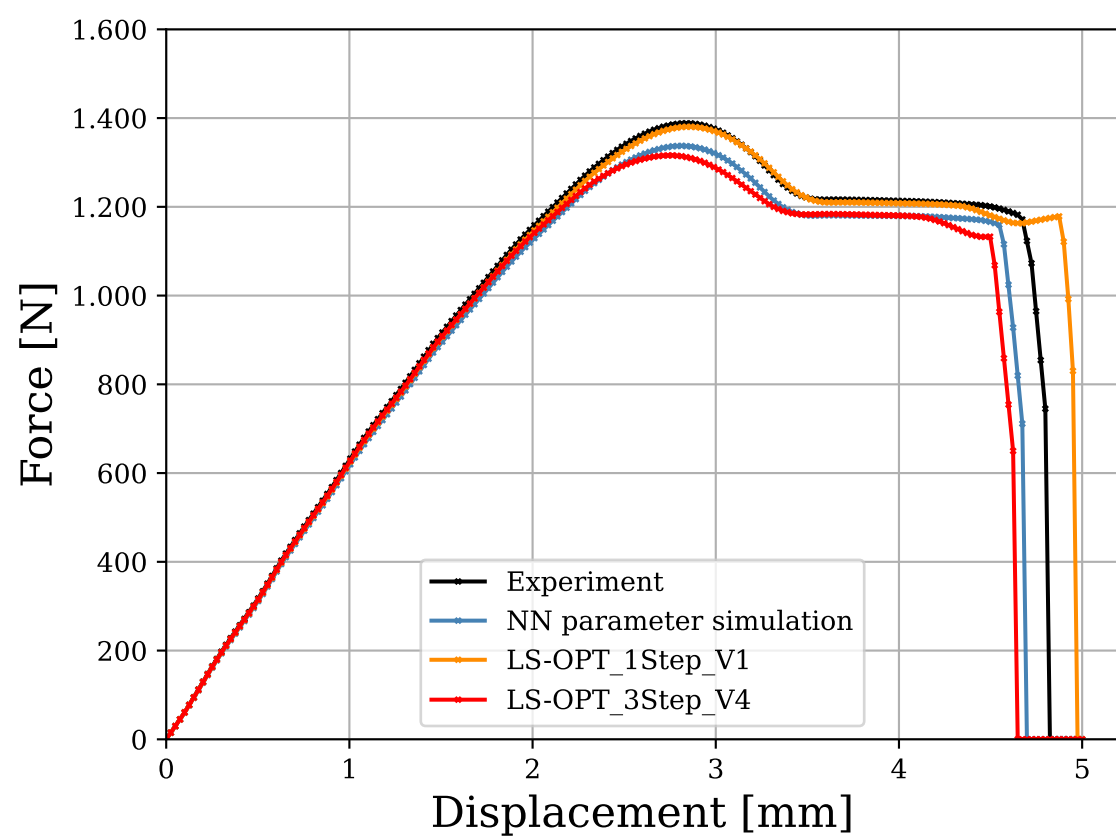

Supplement: Supplementary file 1 [file materials-15-00643-s001.zip › Supplementary_Material/SOC_NN_Pred_LSOPT_Complete/NN_Run_7/FD_Comparison_Tensile_Test_V4.pdf]

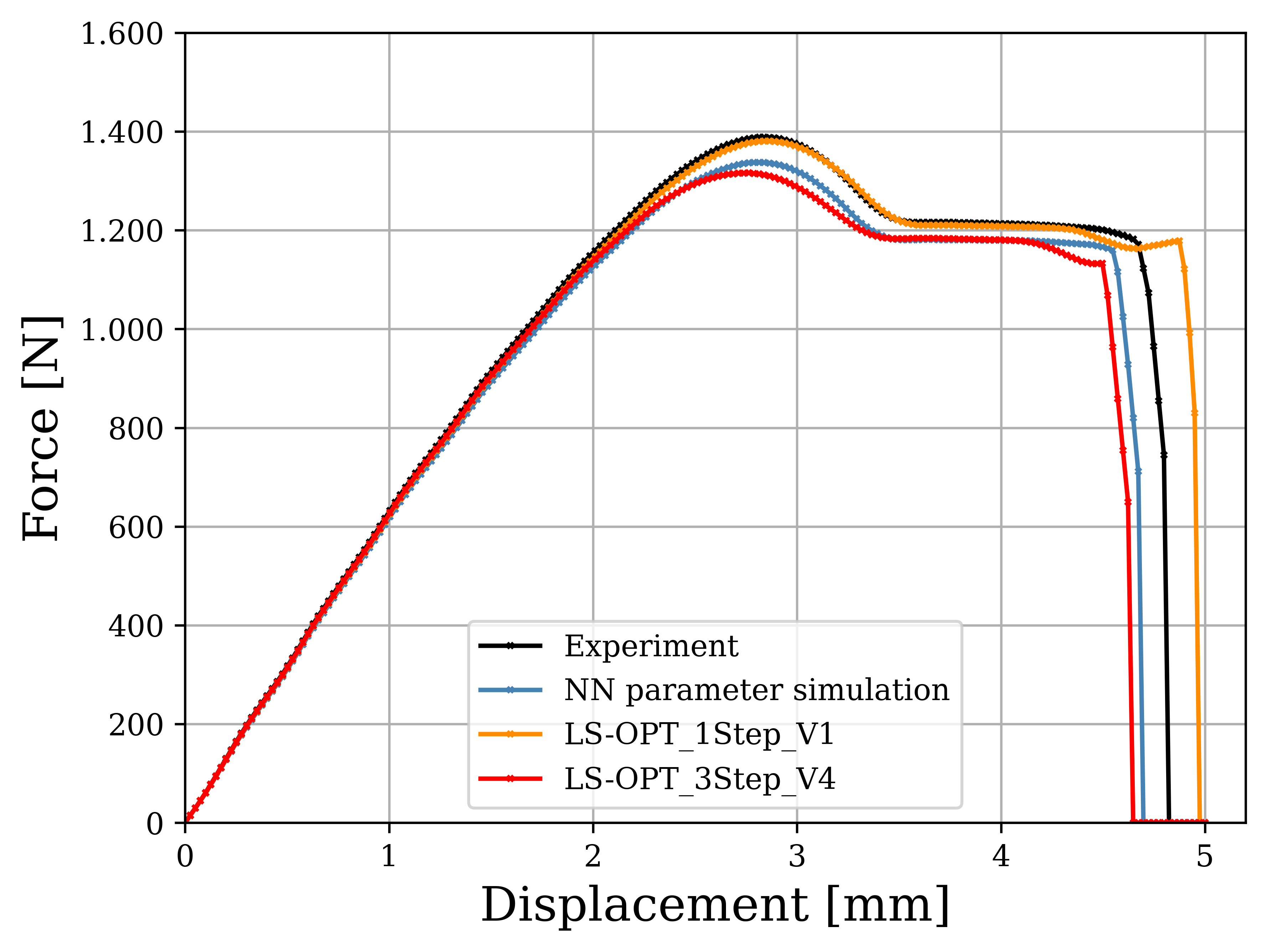

Supplement: Supplementary file 1 [file materials-15-00643-s001.zip › Supplementary_Material/SOC_NN_Pred_LSOPT_Complete/NN_Run_7/FD_Comparison_Tensile_Test_V4.png]

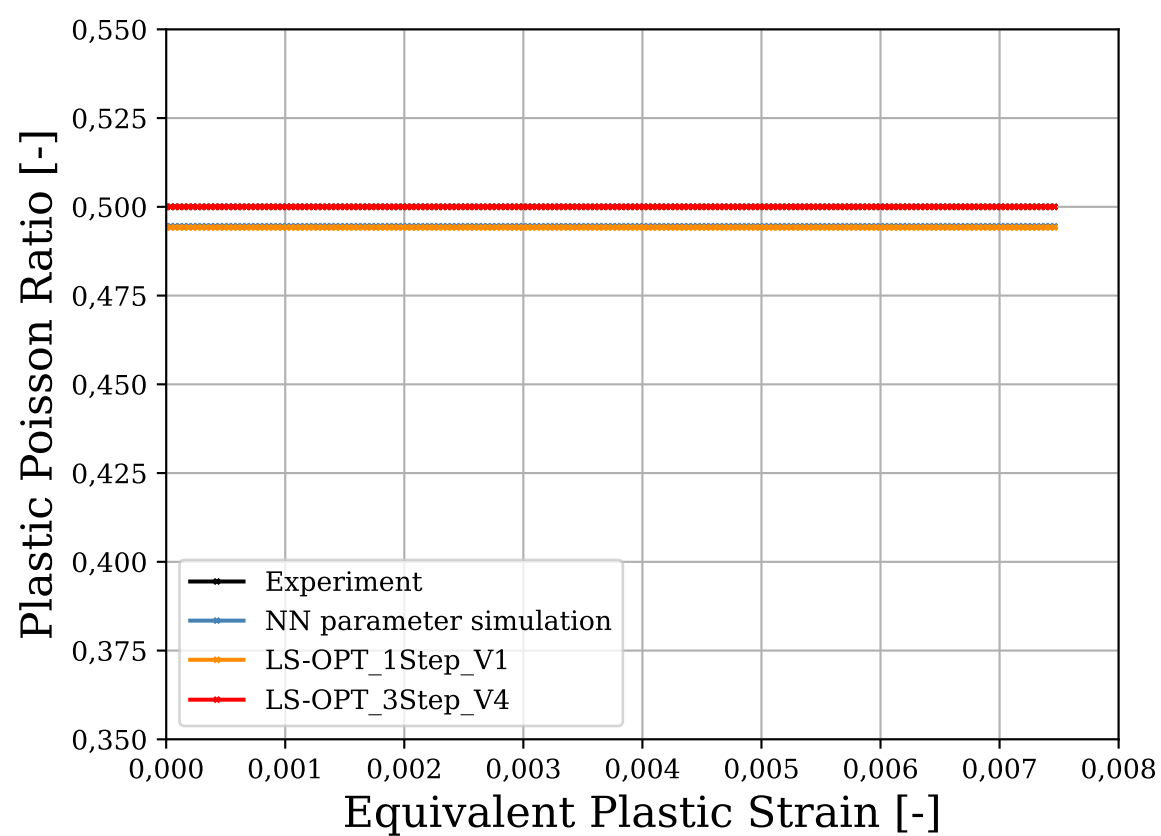

Supplement: Supplementary file 1 [file materials-15-00643-s001.zip › Supplementary_Material/SOC_NN_Pred_LSOPT_Complete/NN_Run_7/PE_Comparison_Compression_Test.pdf]

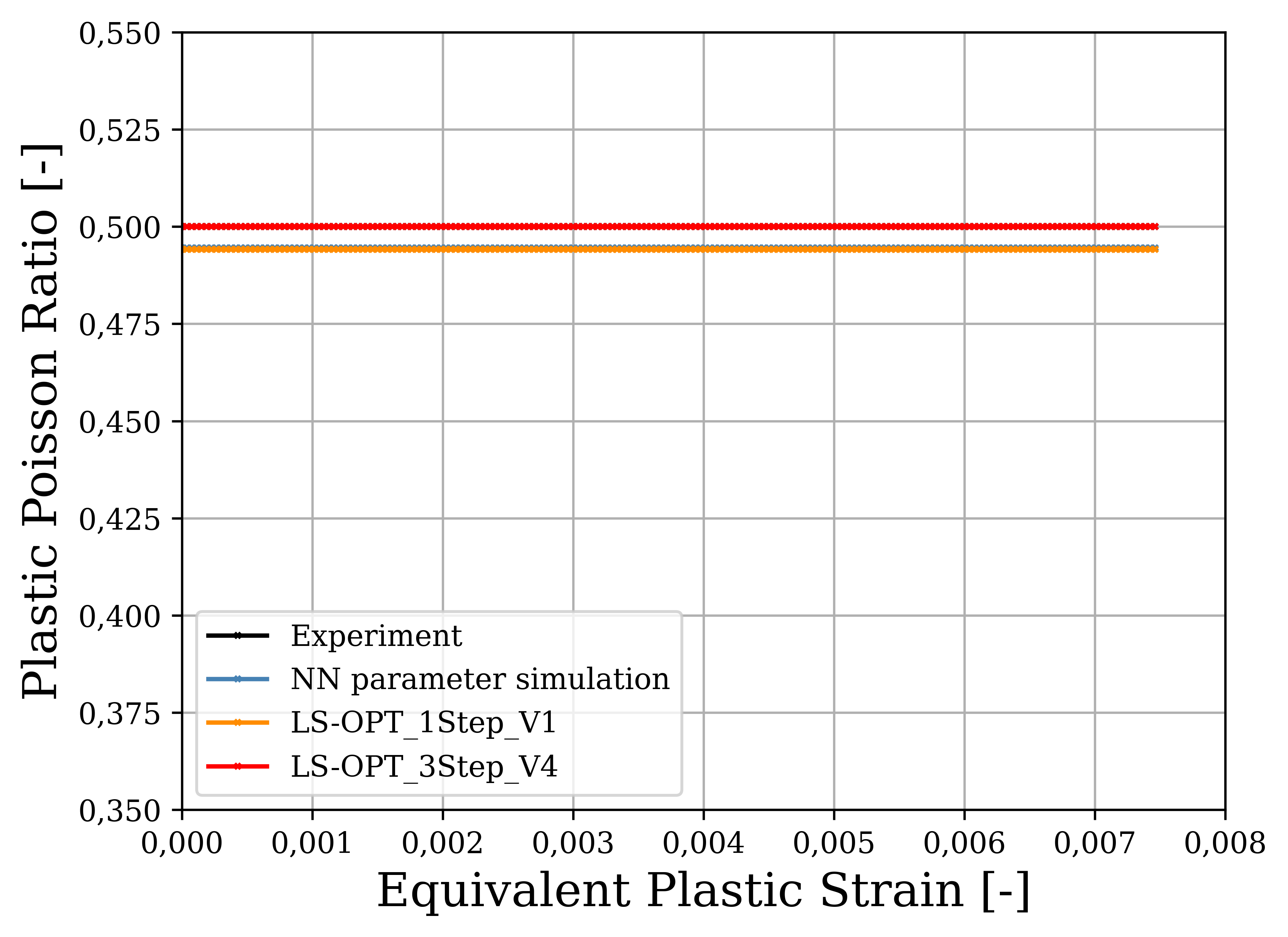

Supplement: Supplementary file 1 [file materials-15-00643-s001.zip › Supplementary_Material/SOC_NN_Pred_LSOPT_Complete/NN_Run_7/PE_Comparison_Compression_Test.png]
