# Supplementary figures and images for: Methodology for Neural Network-Based Material Card Calibration Using LS-DYNA MAT_187_SAMP-1 Considering Failure with GISSMO (part 1 of 2)
Source: Materials (Basel). 2022 Jan 15;15(2):643. doi: 10.3390/ma15020643 (PMC8778971; doi:10.3390/ma15020643)

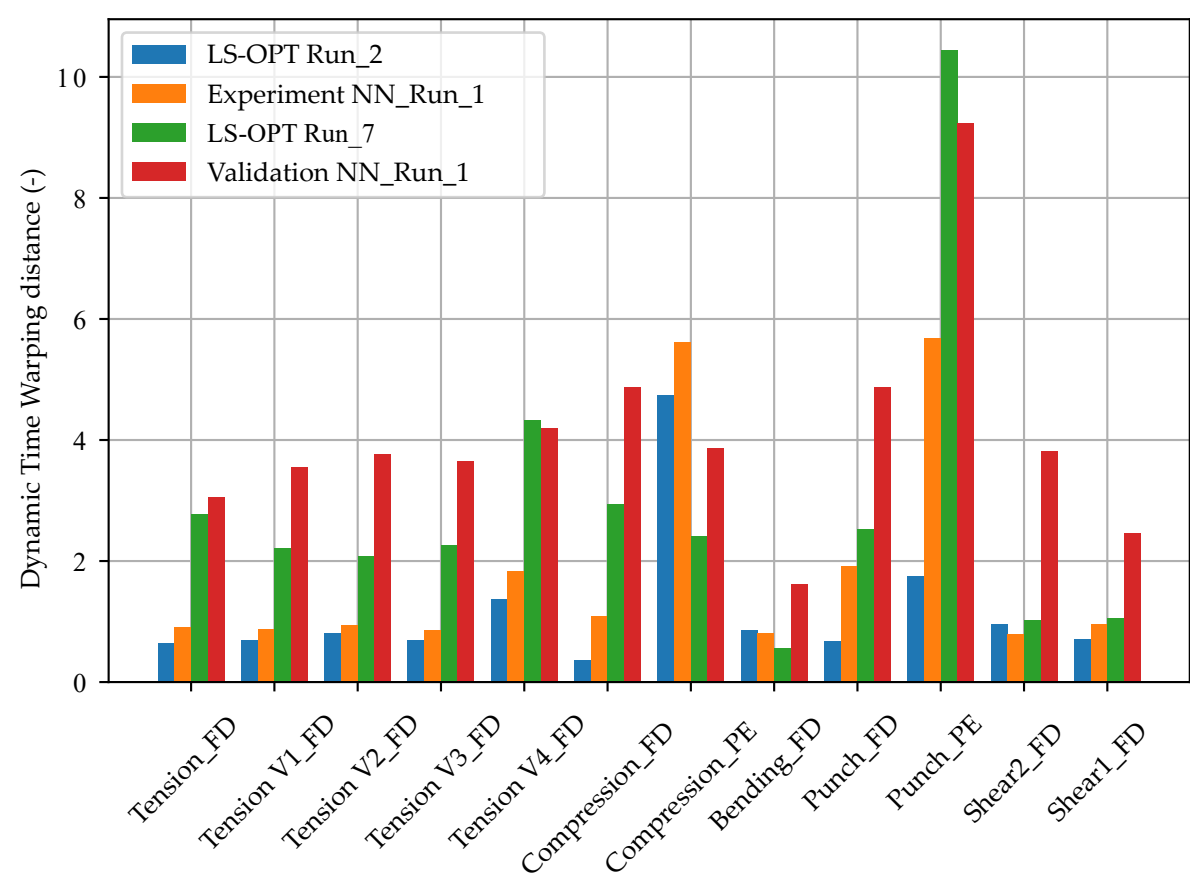

Supplement: Supplementary file 1 [file materials-15-00643-s001.zip › Supplementary_Material/DTW_Tests_SOC_GroupedBar_Complete/DTW_Exp_Pred_PSet_GroupedBar_Run1.pdf]

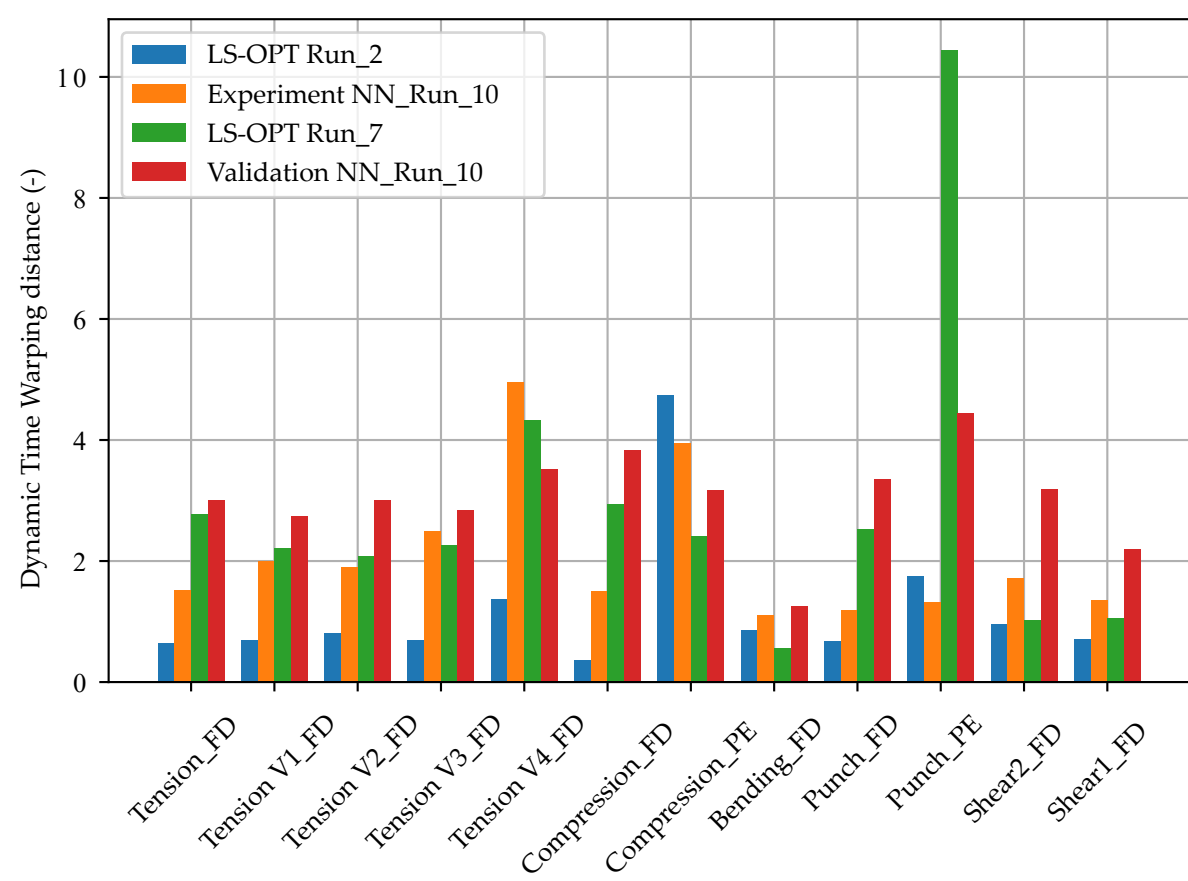

Supplement: Supplementary file 1 [file materials-15-00643-s001.zip › Supplementary_Material/DTW_Tests_SOC_GroupedBar_Complete/DTW_Exp_Pred_PSet_GroupedBar_Run10.pdf]

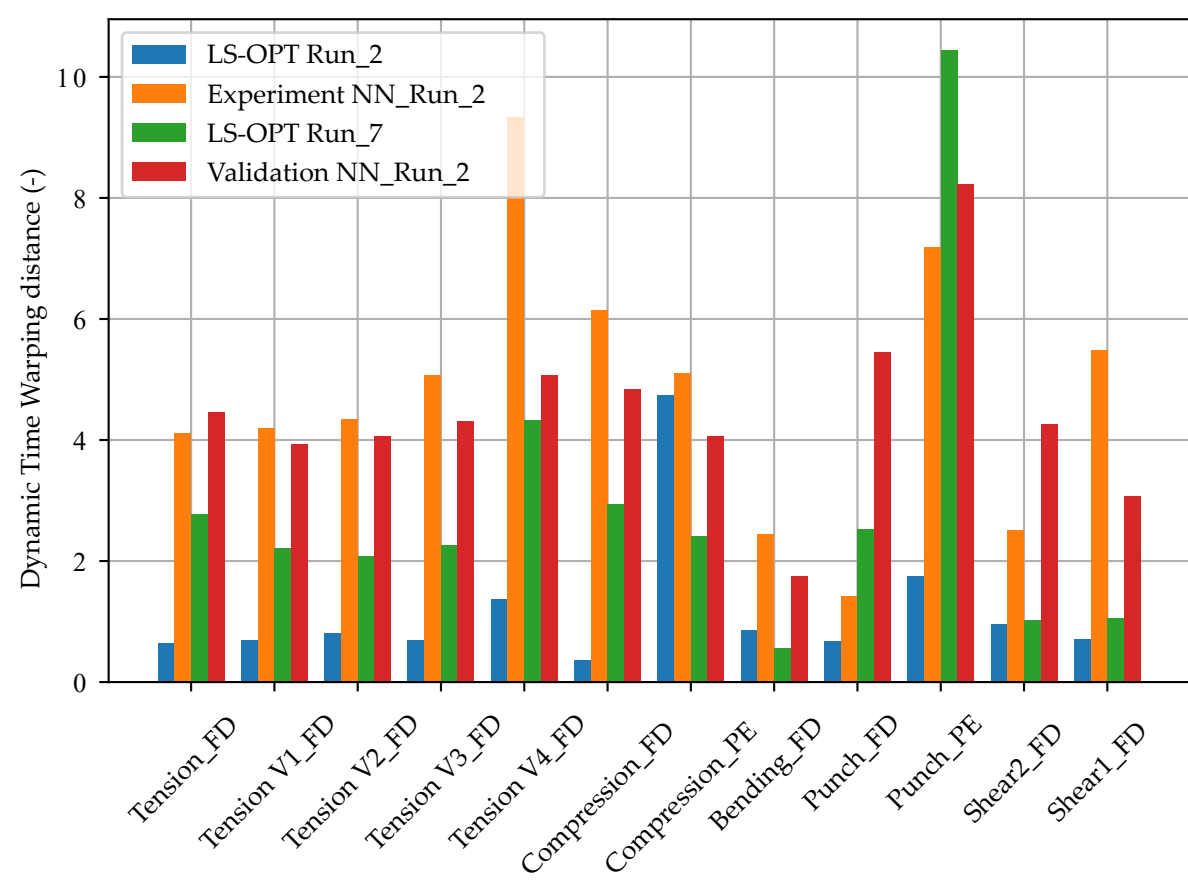

Supplement: Supplementary file 1 [file materials-15-00643-s001.zip › Supplementary_Material/DTW_Tests_SOC_GroupedBar_Complete/DTW_Exp_Pred_PSet_GroupedBar_Run2.pdf]

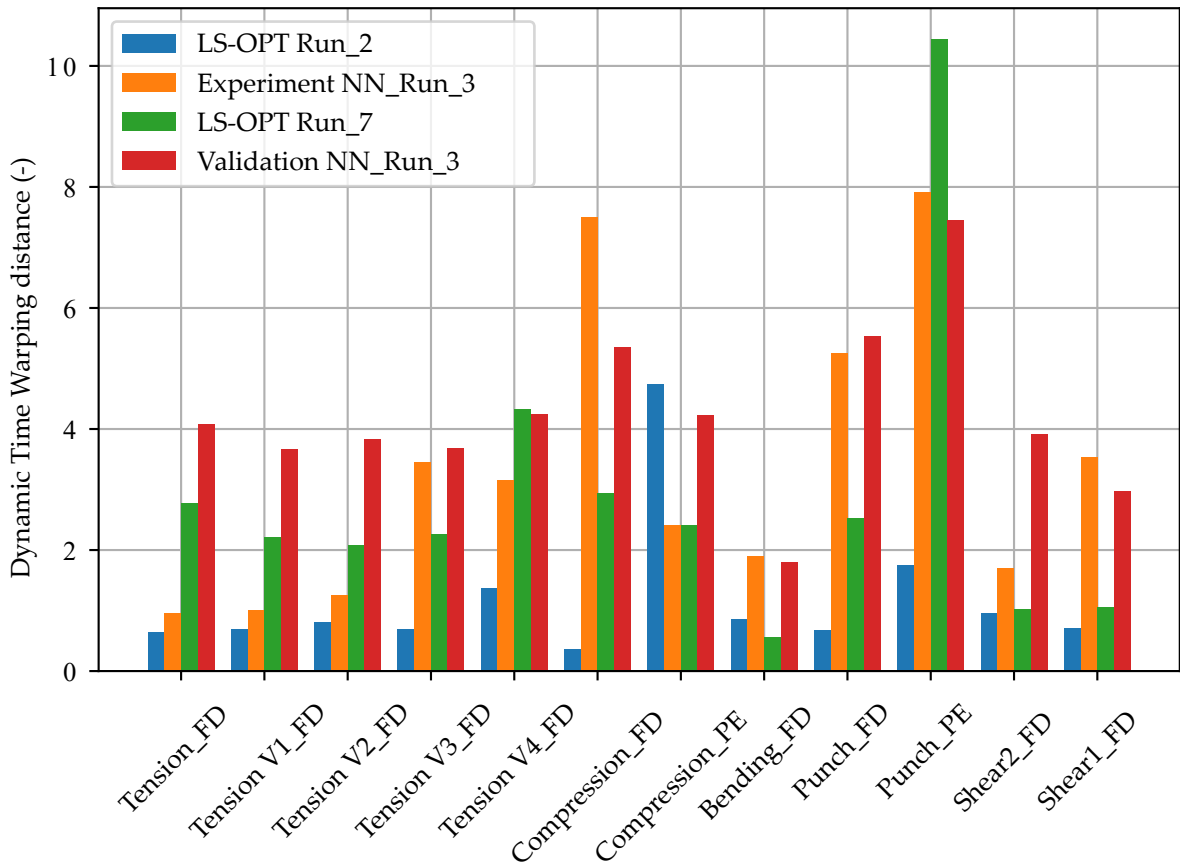

Supplement: Supplementary file 1 [file materials-15-00643-s001.zip › Supplementary_Material/DTW_Tests_SOC_GroupedBar_Complete/DTW_Exp_Pred_PSet_GroupedBar_Run3.pdf]

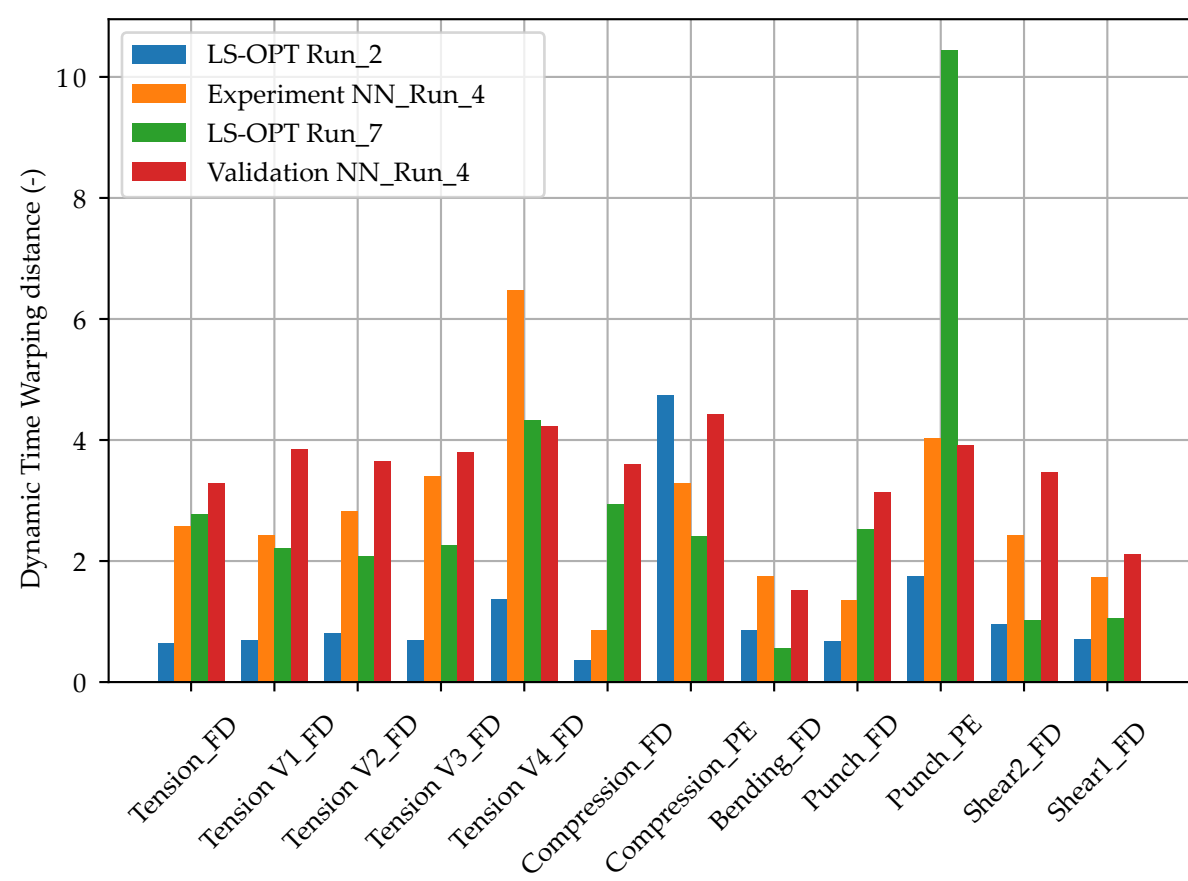

Supplement: Supplementary file 1 [file materials-15-00643-s001.zip › Supplementary_Material/DTW_Tests_SOC_GroupedBar_Complete/DTW_Exp_Pred_PSet_GroupedBar_Run4.pdf]

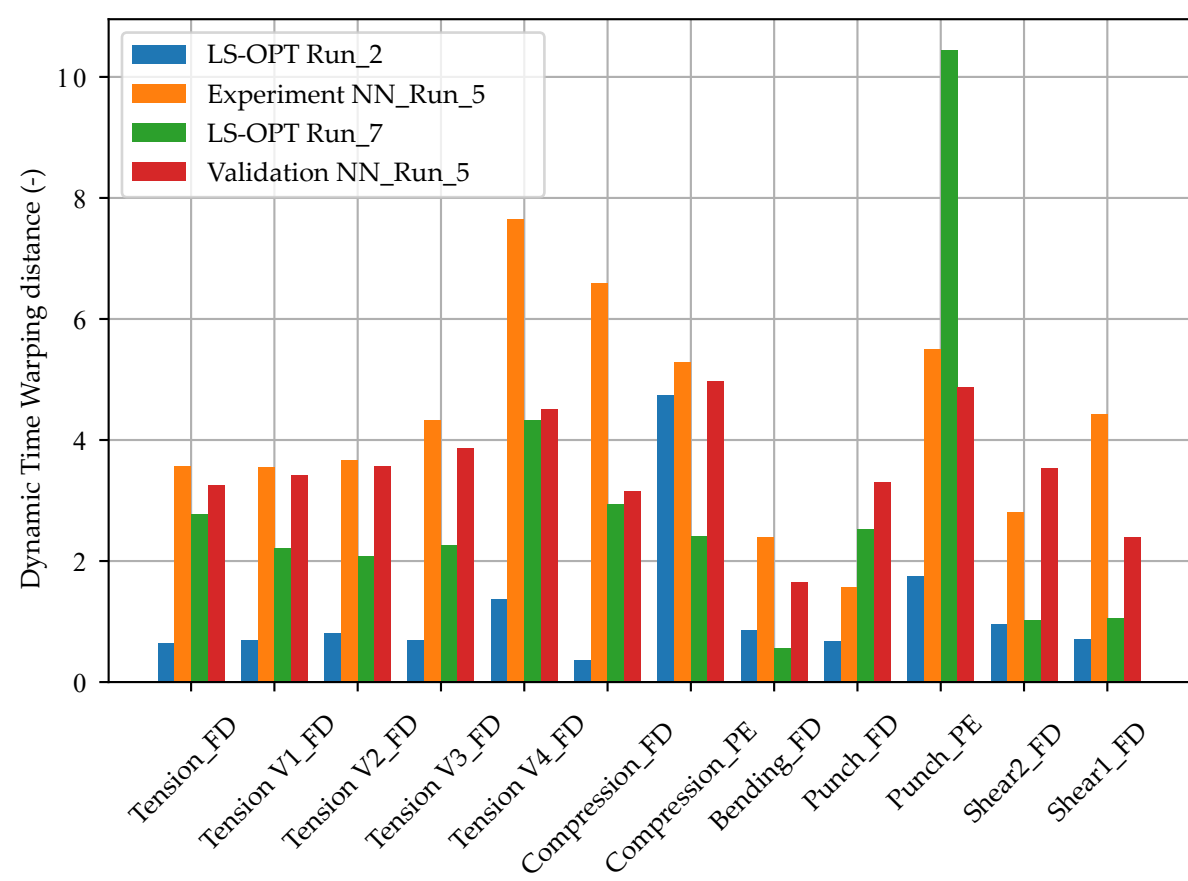

Supplement: Supplementary file 1 [file materials-15-00643-s001.zip › Supplementary_Material/DTW_Tests_SOC_GroupedBar_Complete/DTW_Exp_Pred_PSet_GroupedBar_Run5.pdf]

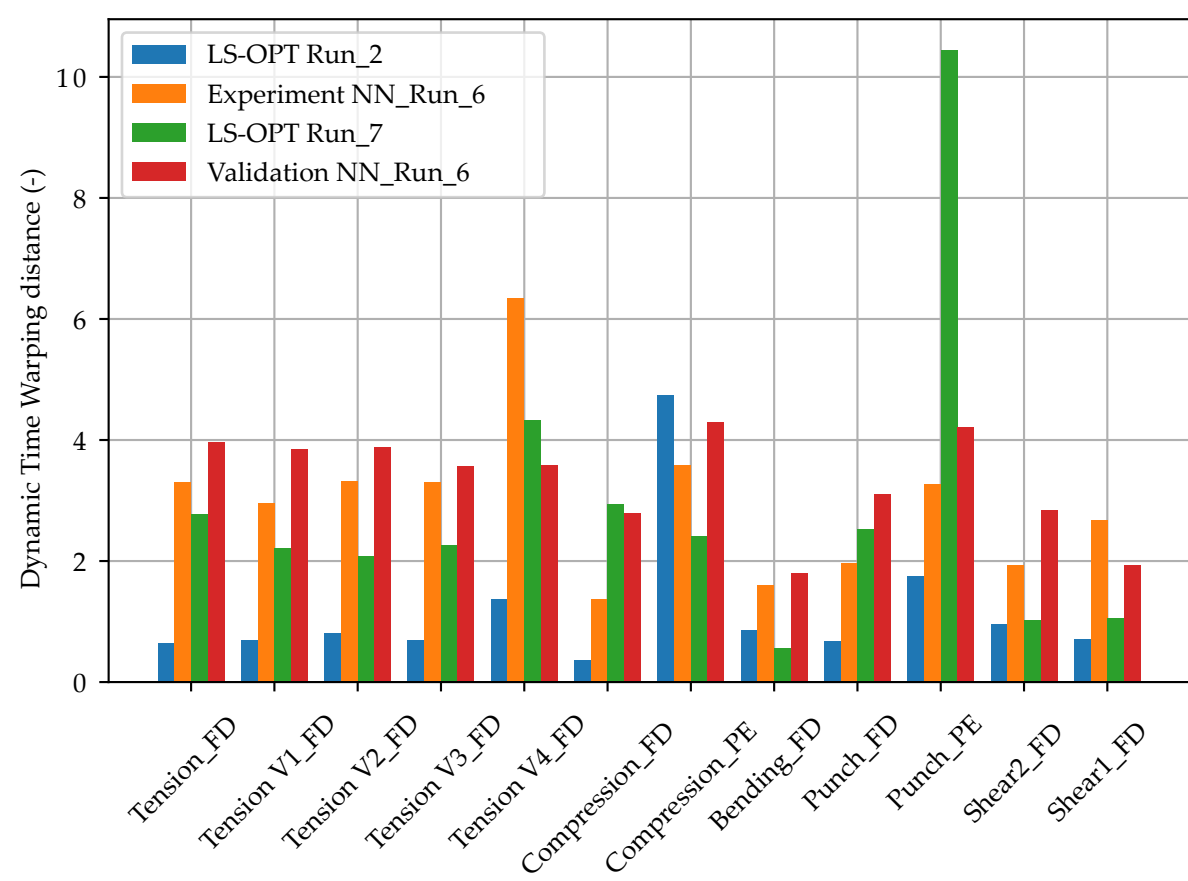

Supplement: Supplementary file 1 [file materials-15-00643-s001.zip › Supplementary_Material/DTW_Tests_SOC_GroupedBar_Complete/DTW_Exp_Pred_PSet_GroupedBar_Run6.pdf]

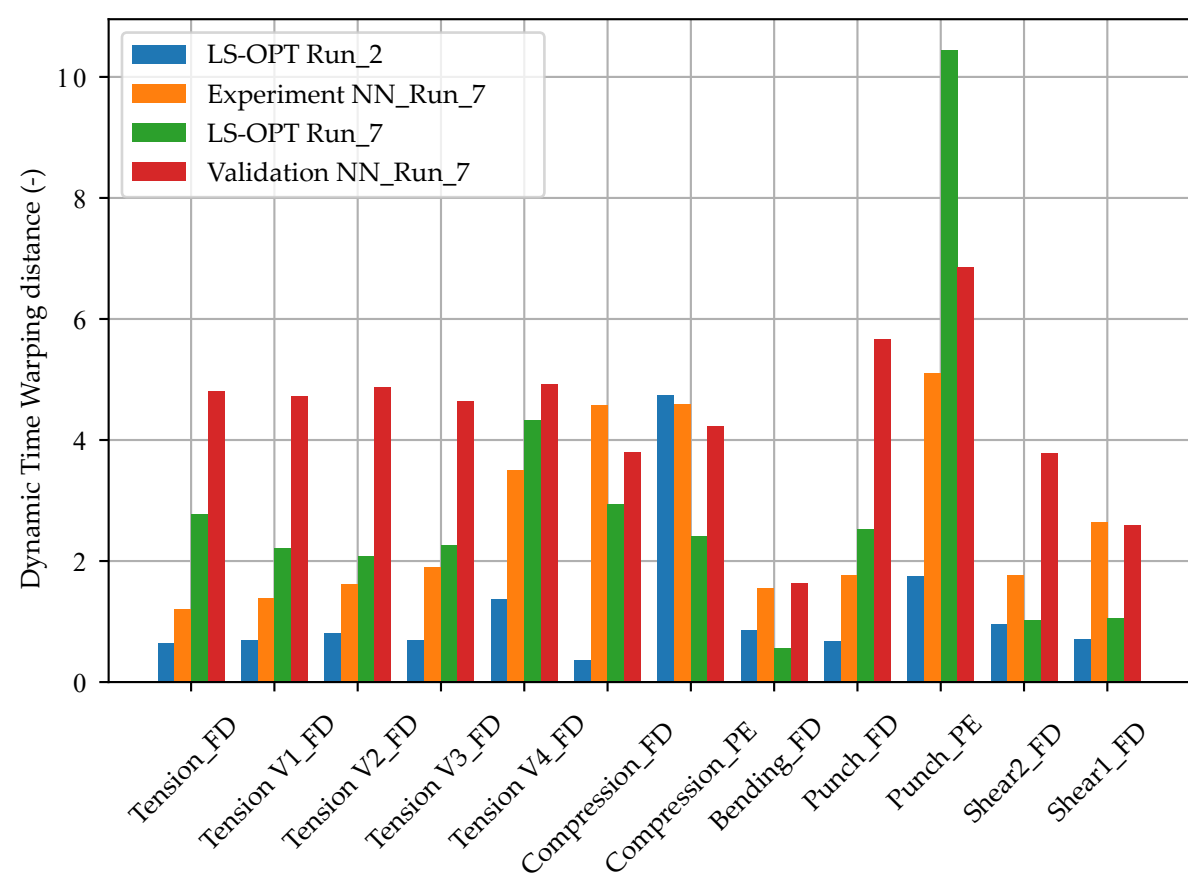

Supplement: Supplementary file 1 [file materials-15-00643-s001.zip › Supplementary_Material/DTW_Tests_SOC_GroupedBar_Complete/DTW_Exp_Pred_PSet_GroupedBar_Run7.pdf]

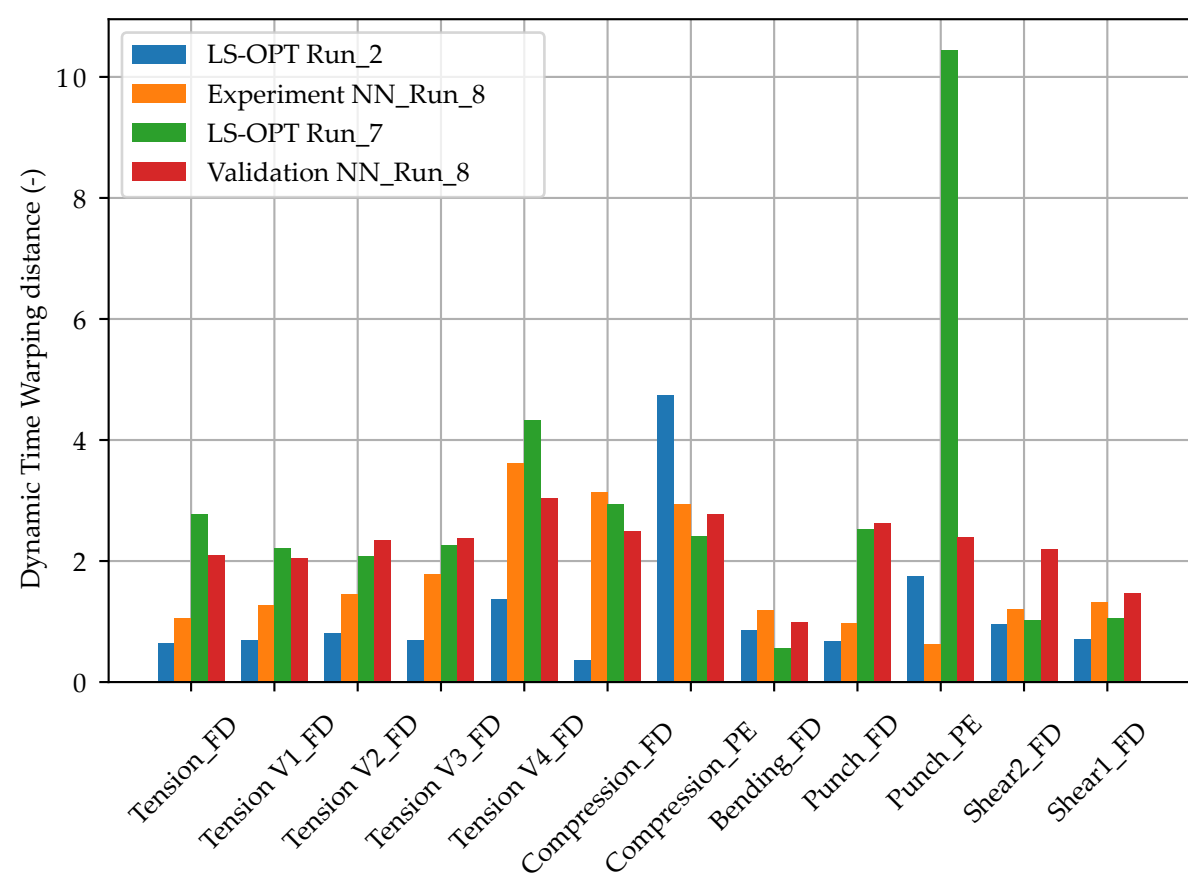

Supplement: Supplementary file 1 [file materials-15-00643-s001.zip › Supplementary_Material/DTW_Tests_SOC_GroupedBar_Complete/DTW_Exp_Pred_PSet_GroupedBar_Run8.pdf]

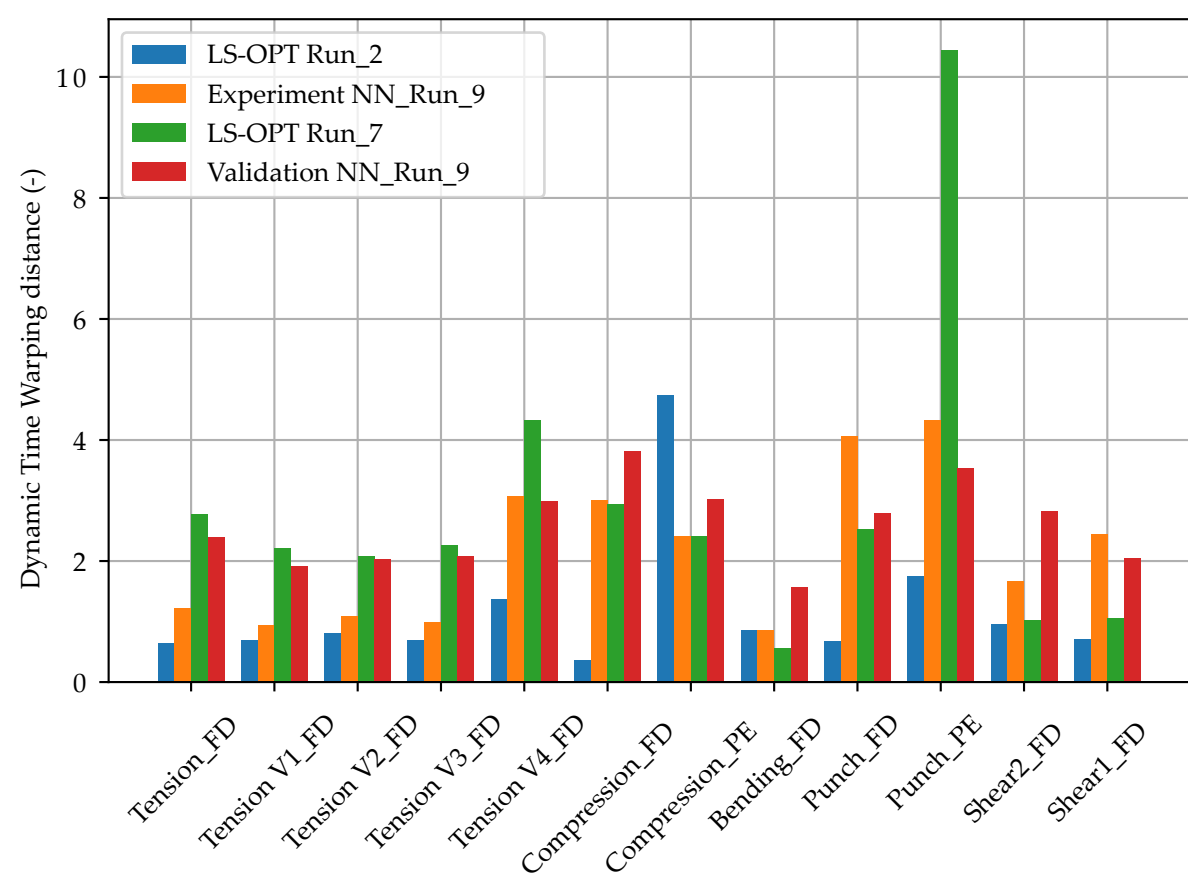

Supplement: Supplementary file 1 [file materials-15-00643-s001.zip › Supplementary_Material/DTW_Tests_SOC_GroupedBar_Complete/DTW_Exp_Pred_PSet_GroupedBar_Run9.pdf]

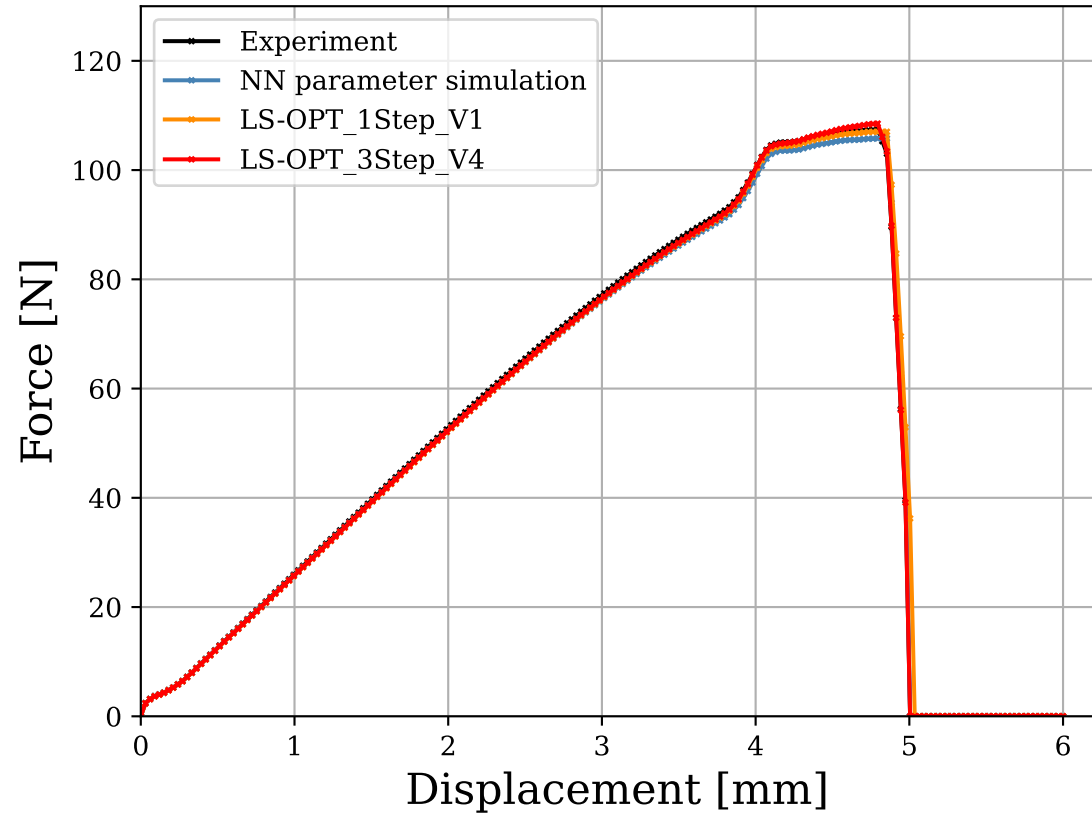

Supplement: Supplementary file 1 [file materials-15-00643-s001.zip › Supplementary_Material/SOC_NN_Pred_LSOPT_Complete/NN_Run_10/FD_Comparison_Bending_Test.pdf]

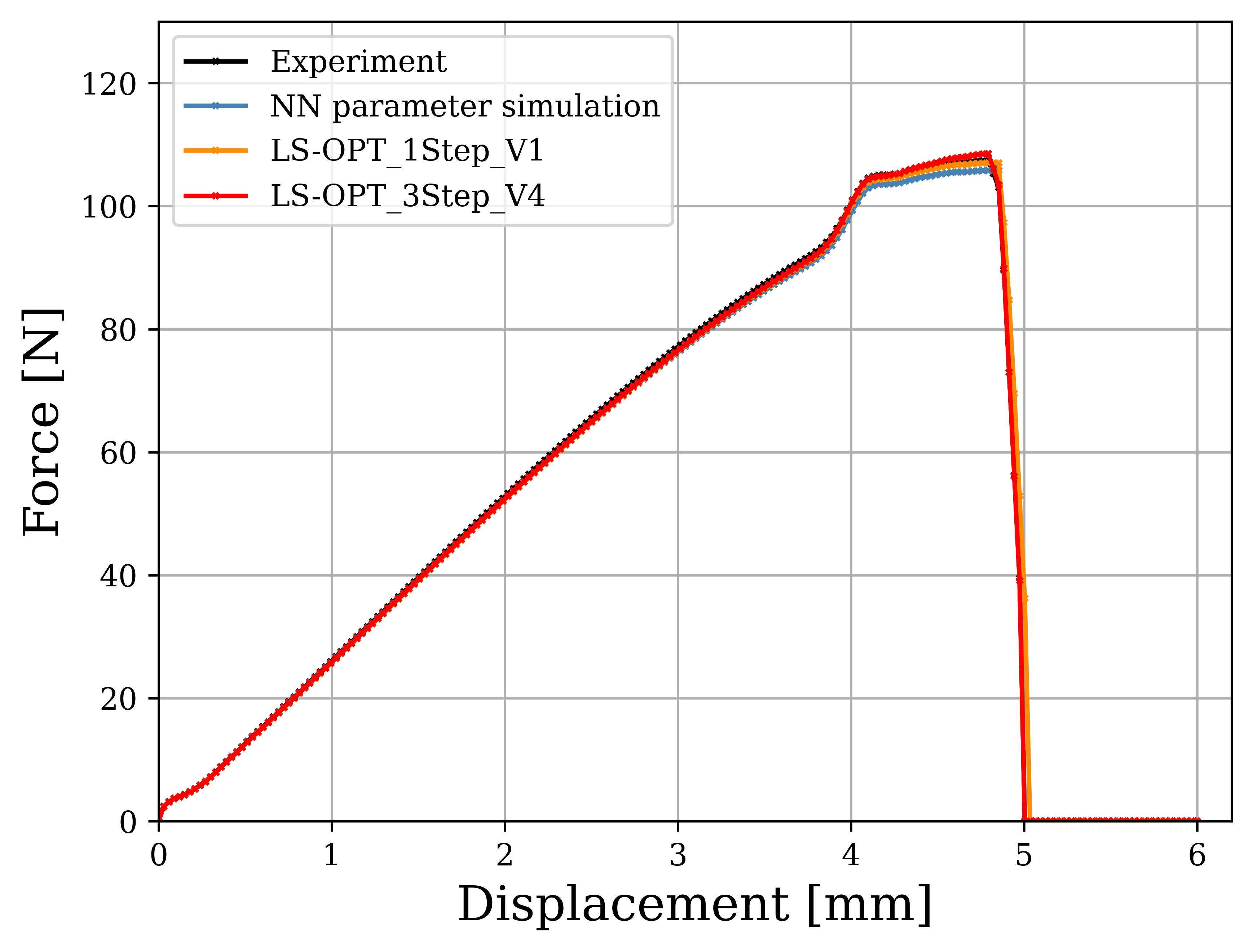

Supplement: Supplementary file 1 [file materials-15-00643-s001.zip › Supplementary_Material/SOC_NN_Pred_LSOPT_Complete/NN_Run_10/FD_Comparison_Bending_Test.png]

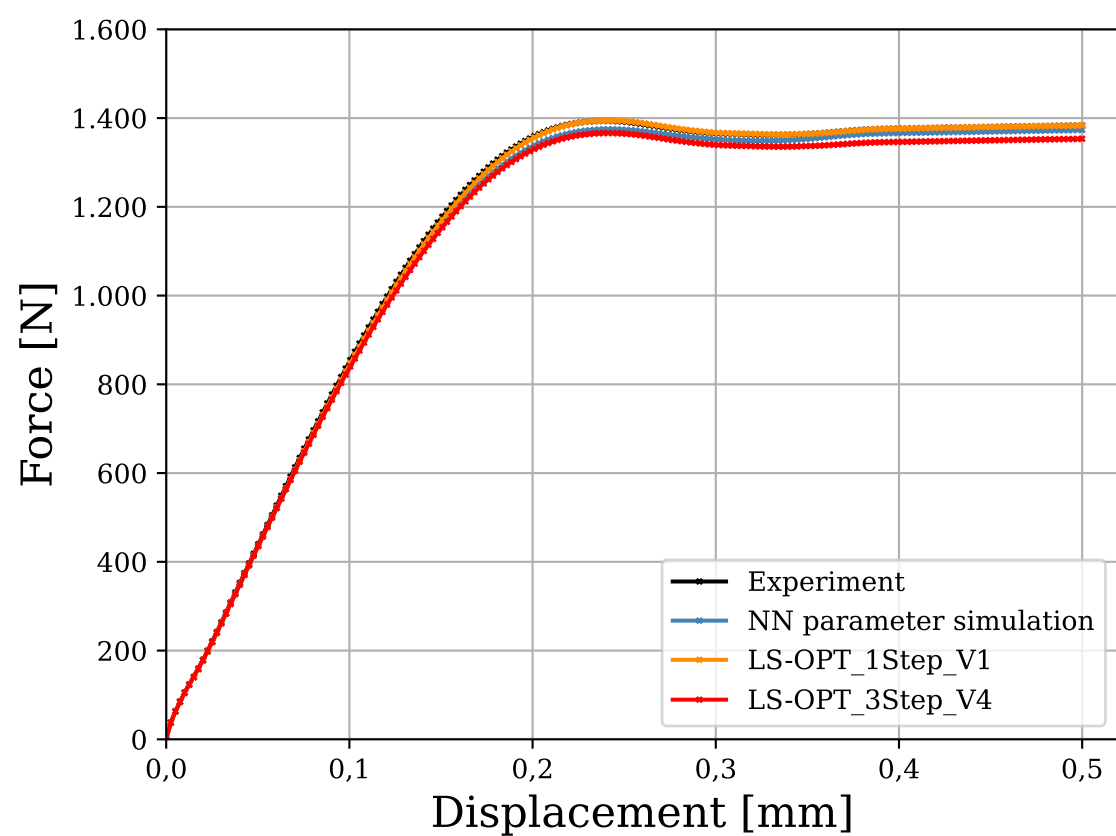

Supplement: Supplementary file 1 [file materials-15-00643-s001.zip › Supplementary_Material/SOC_NN_Pred_LSOPT_Complete/NN_Run_10/FD_Comparison_Compression_Test.pdf]

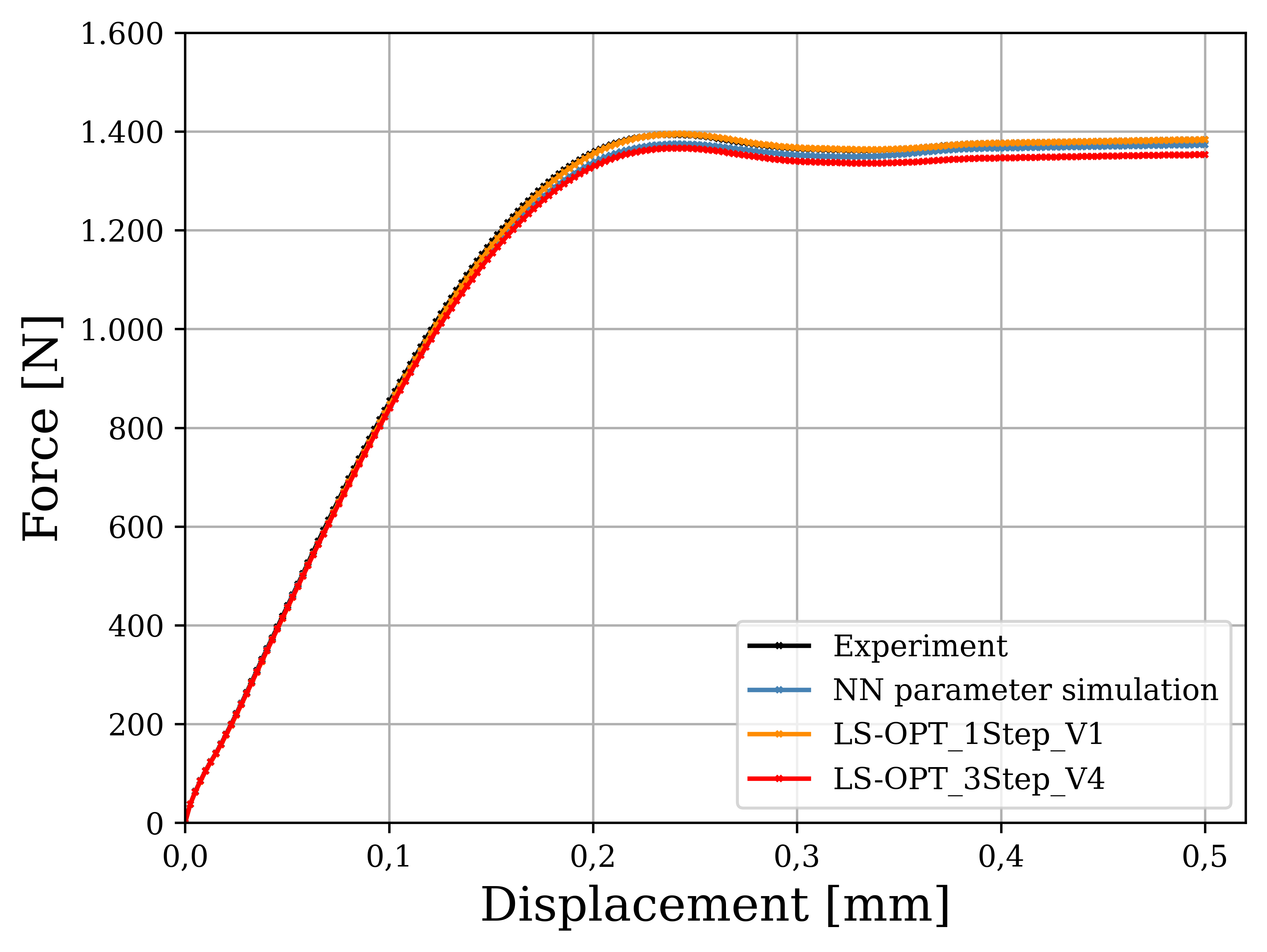

Supplement: Supplementary file 1 [file materials-15-00643-s001.zip › Supplementary_Material/SOC_NN_Pred_LSOPT_Complete/NN_Run_10/FD_Comparison_Compression_Test.png]

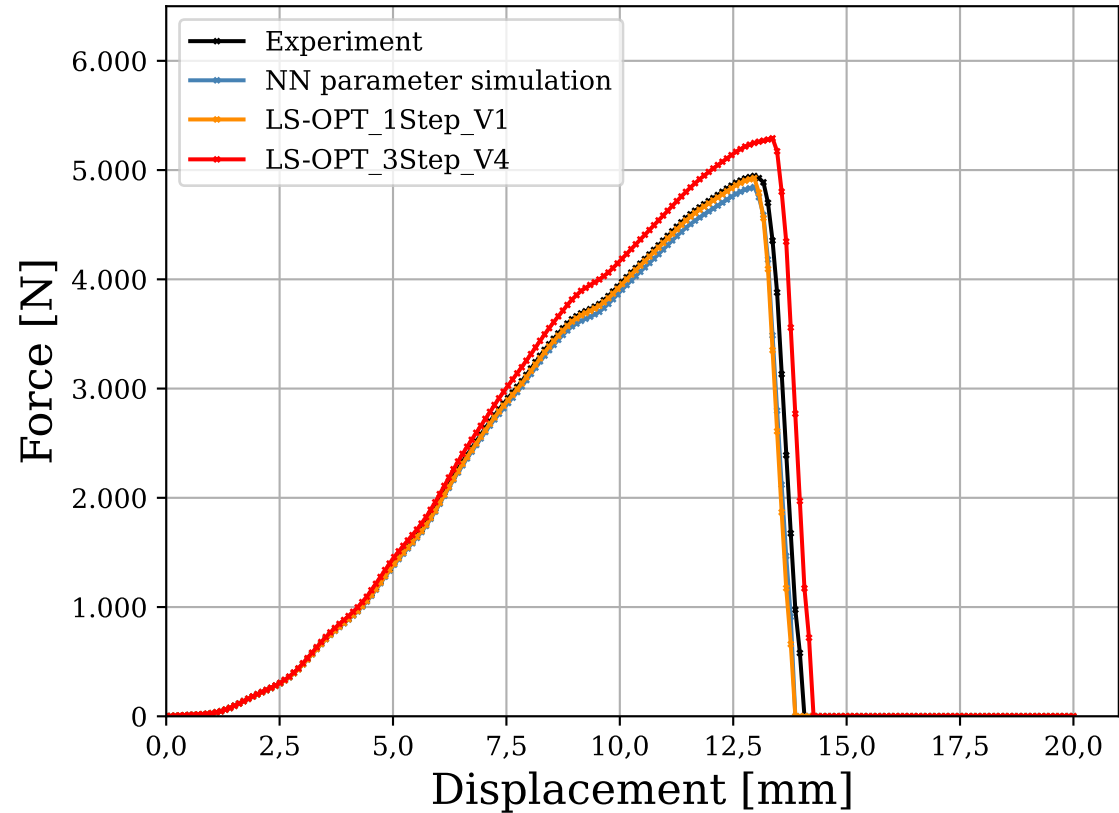

Supplement: Supplementary file 1 [file materials-15-00643-s001.zip › Supplementary_Material/SOC_NN_Pred_LSOPT_Complete/NN_Run_10/FD_Comparison_Punch_Test.pdf]

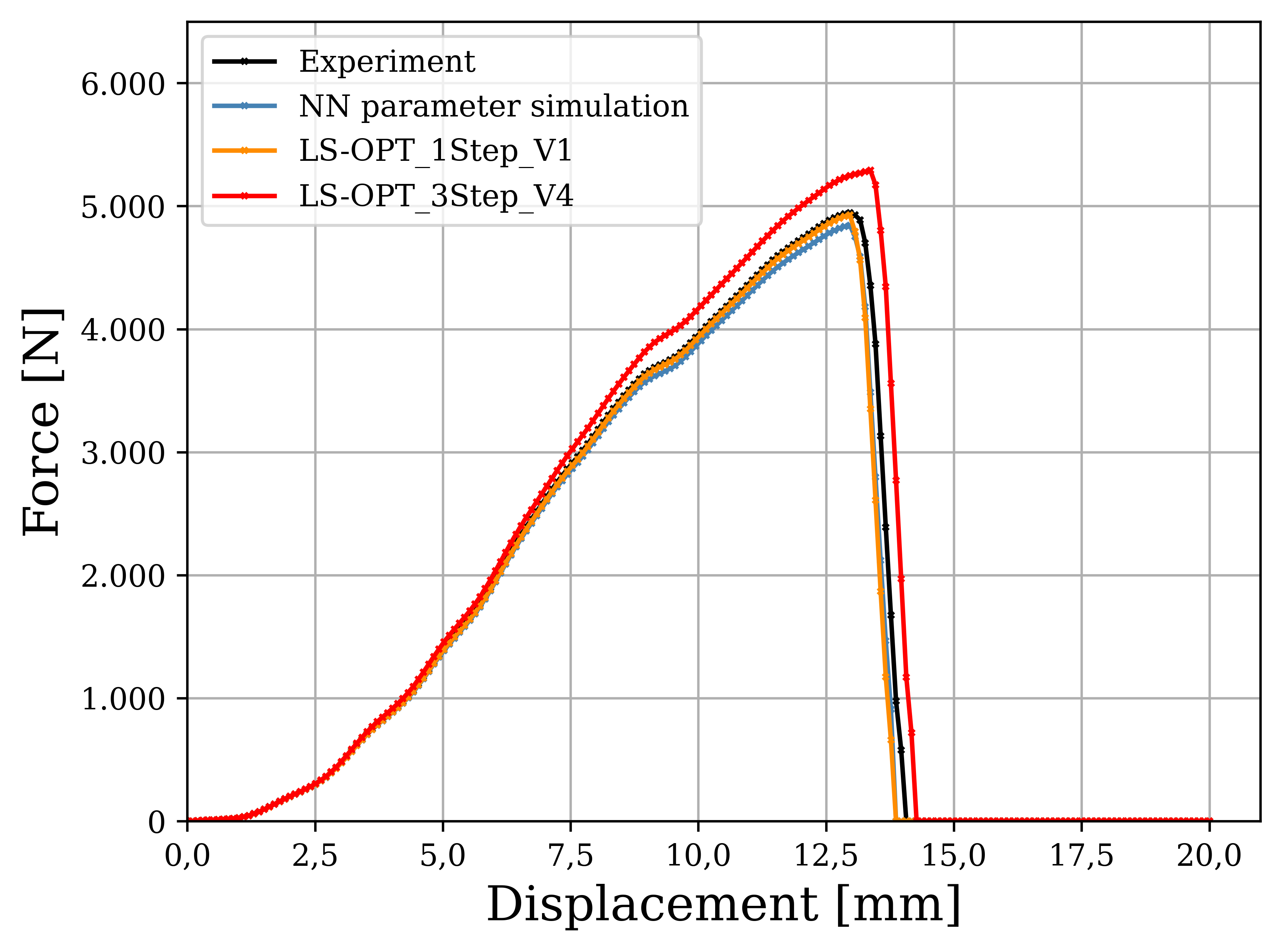

Supplement: Supplementary file 1 [file materials-15-00643-s001.zip › Supplementary_Material/SOC_NN_Pred_LSOPT_Complete/NN_Run_10/FD_Comparison_Punch_Test.png]

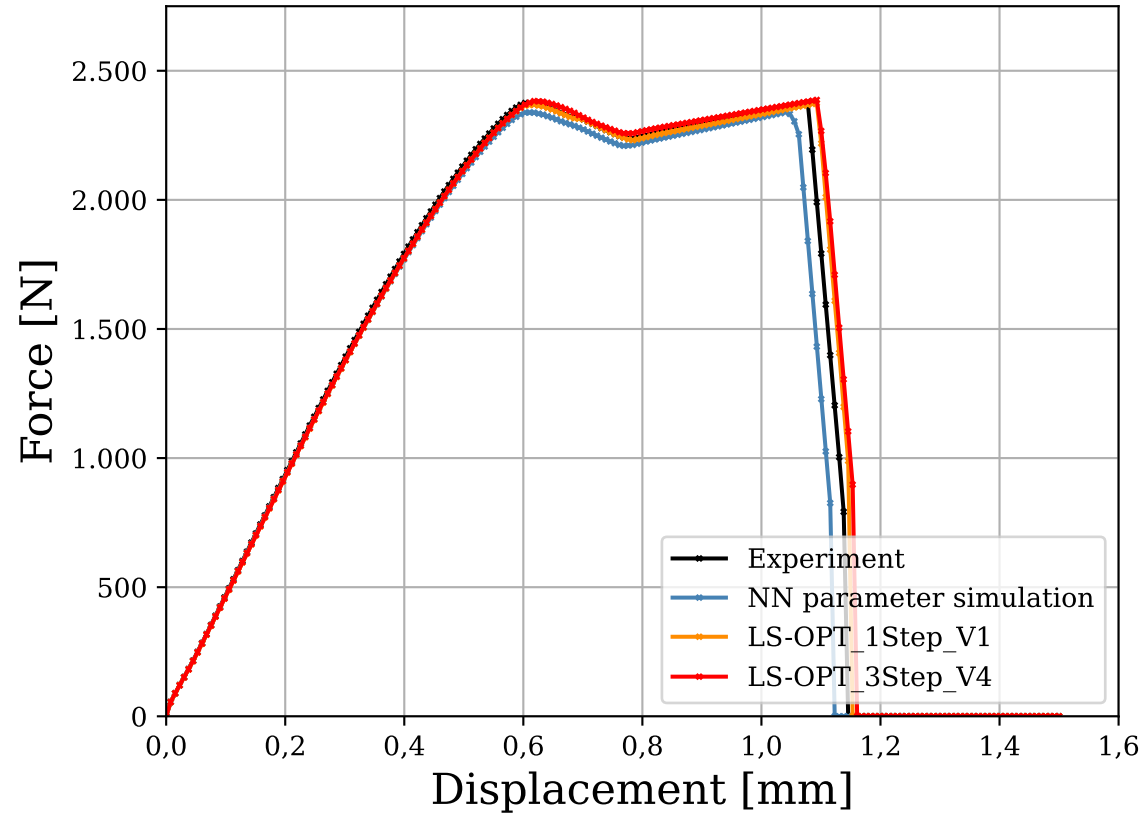

Supplement: Supplementary file 1 [file materials-15-00643-s001.zip › Supplementary_Material/SOC_NN_Pred_LSOPT_Complete/NN_Run_10/FD_Comparison_Shear_ASTM_Test.pdf]

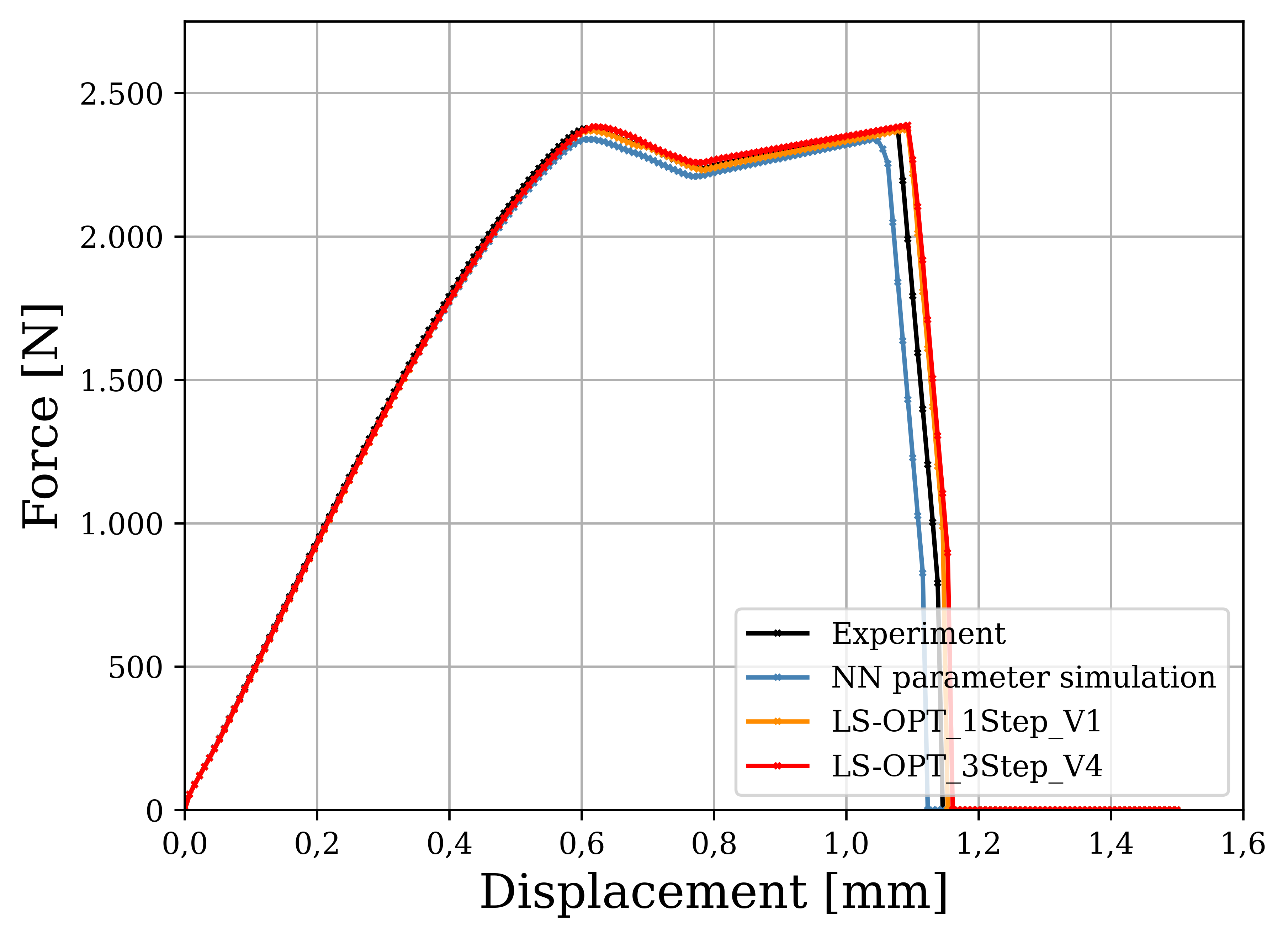

Supplement: Supplementary file 1 [file materials-15-00643-s001.zip › Supplementary_Material/SOC_NN_Pred_LSOPT_Complete/NN_Run_10/FD_Comparison_Shear_ASTM_Test.png]

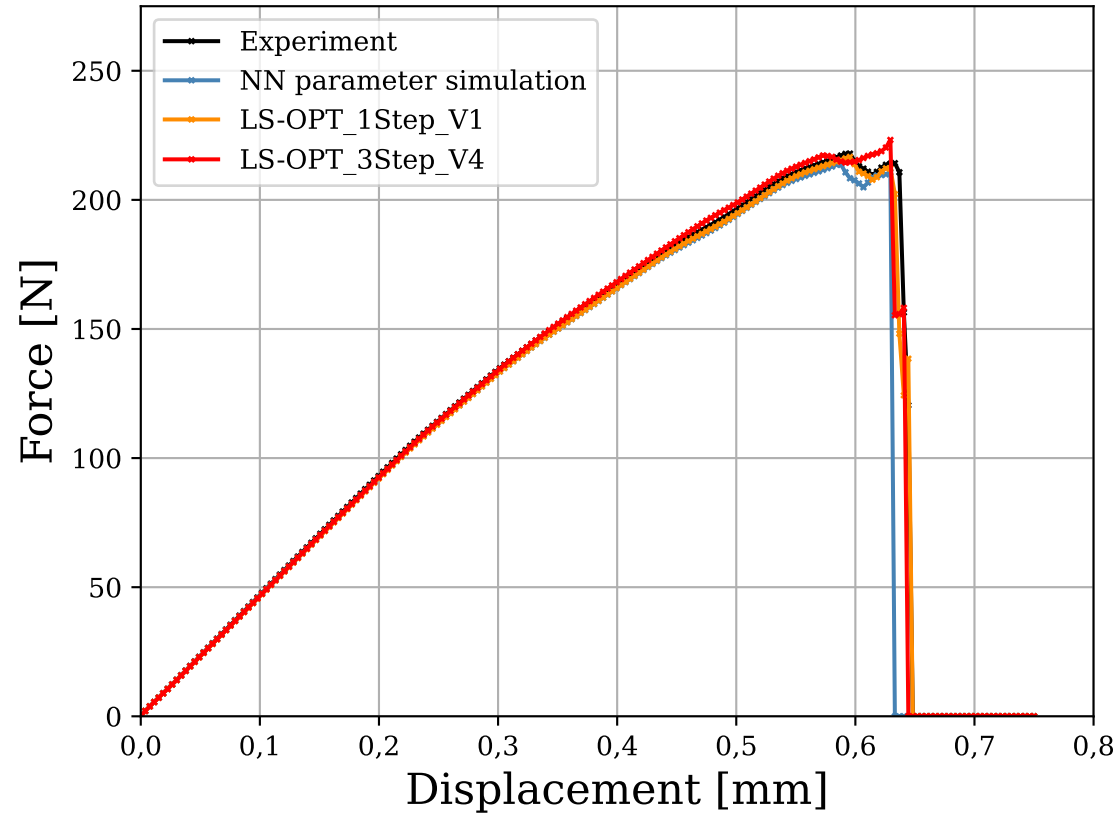

Supplement: Supplementary file 1 [file materials-15-00643-s001.zip › Supplementary_Material/SOC_NN_Pred_LSOPT_Complete/NN_Run_10/FD_Comparison_Shear_Dynamore_Test.pdf]

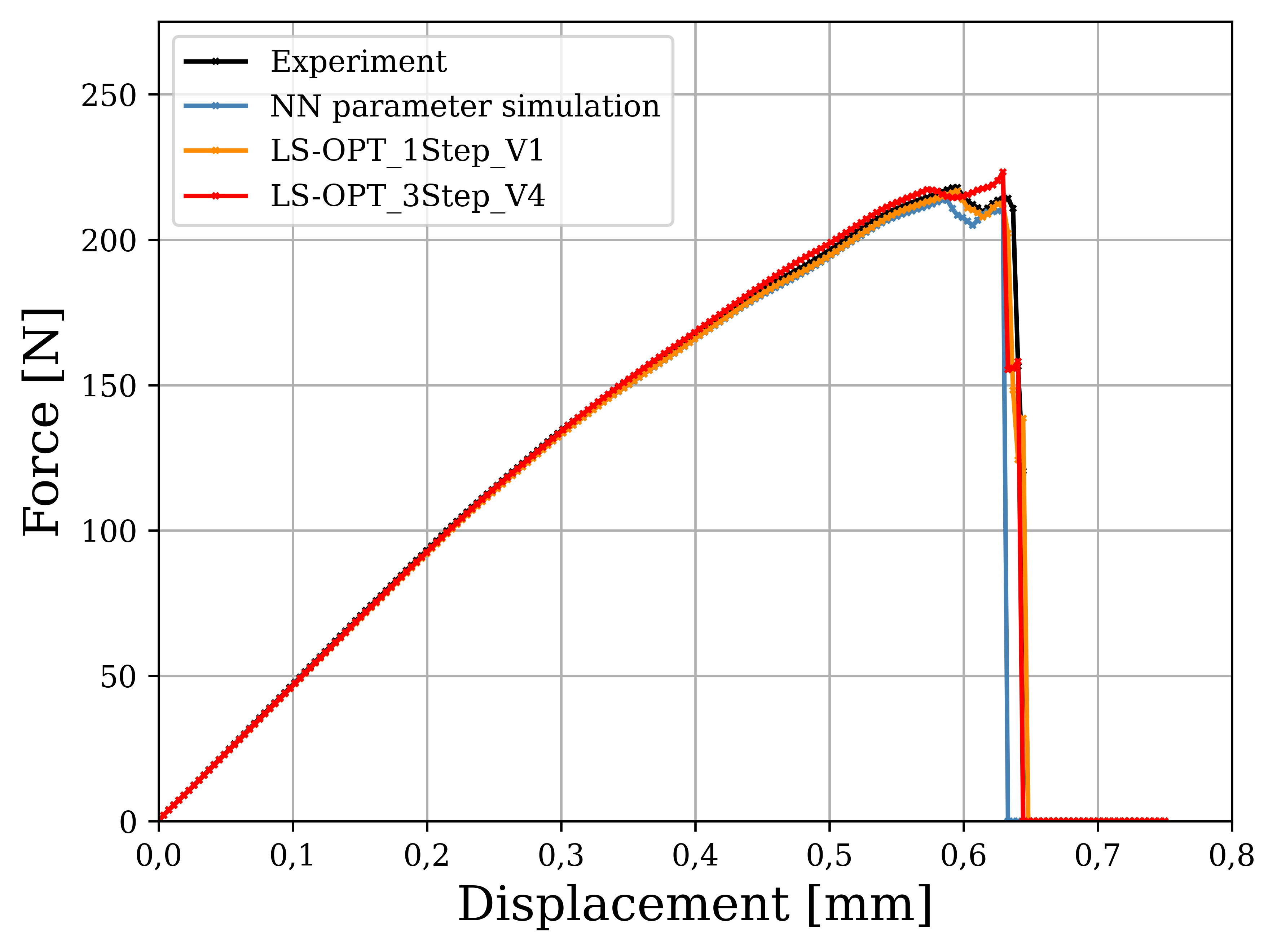

Supplement: Supplementary file 1 [file materials-15-00643-s001.zip › Supplementary_Material/SOC_NN_Pred_LSOPT_Complete/NN_Run_10/FD_Comparison_Shear_Dynamore_Test.png]

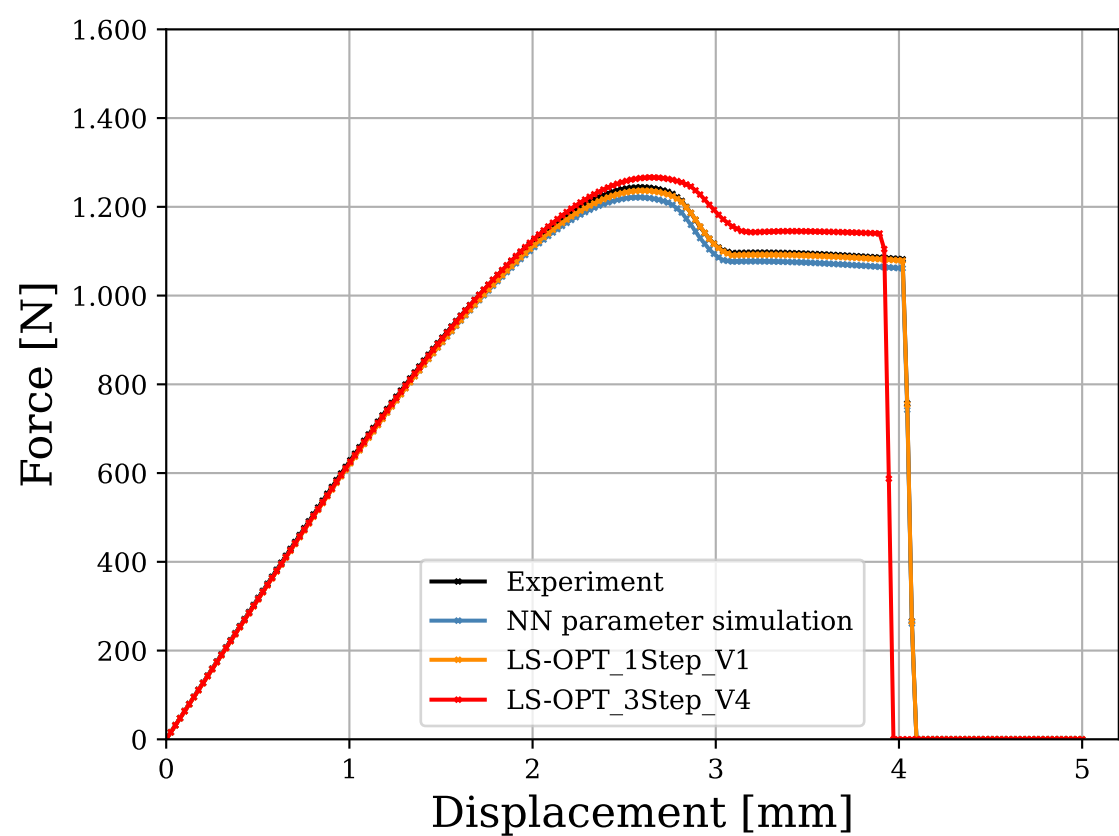

Supplement: Supplementary file 1 [file materials-15-00643-s001.zip › Supplementary_Material/SOC_NN_Pred_LSOPT_Complete/NN_Run_10/FD_Comparison_Tensile_Test.pdf]

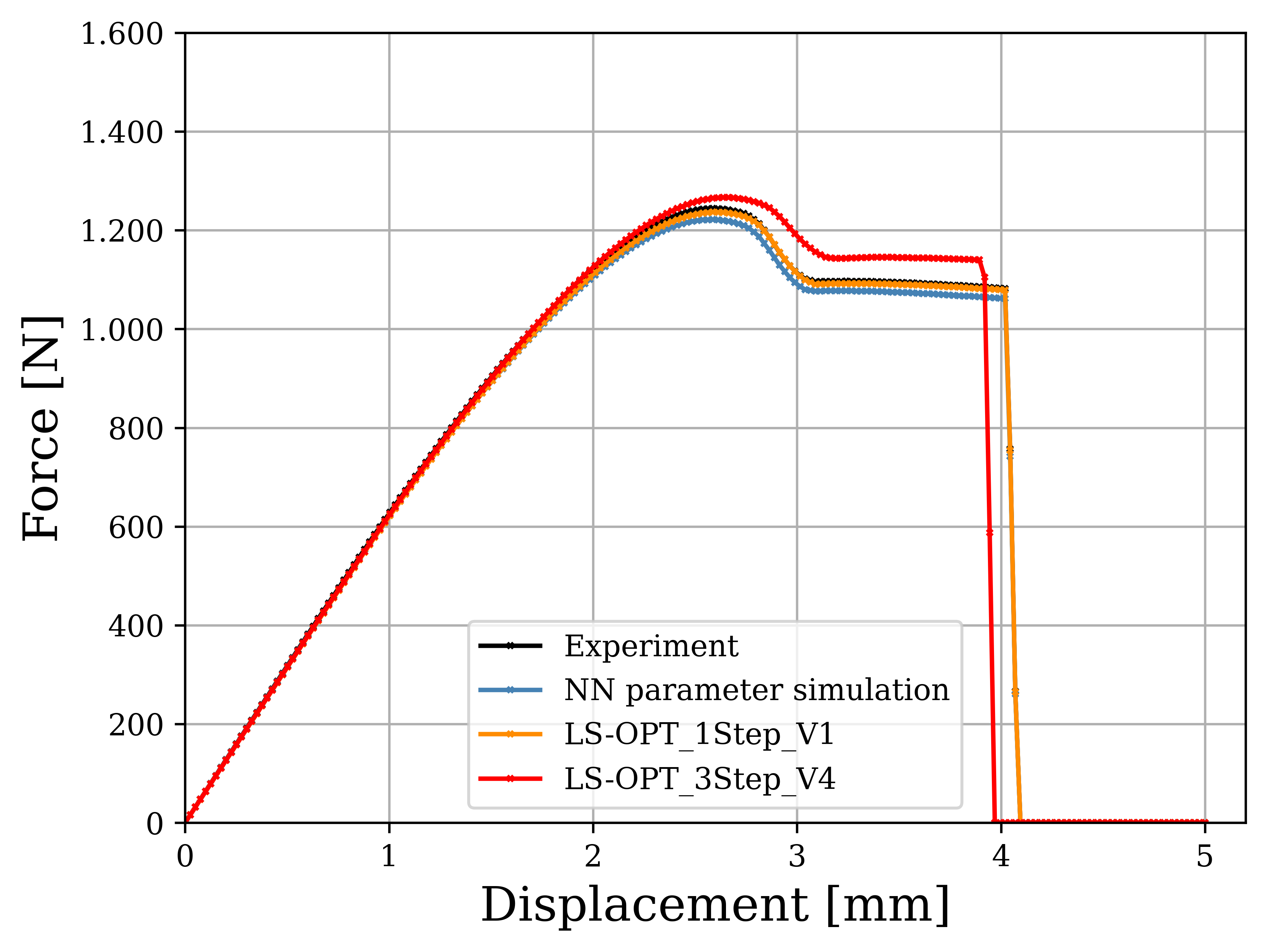

Supplement: Supplementary file 1 [file materials-15-00643-s001.zip › Supplementary_Material/SOC_NN_Pred_LSOPT_Complete/NN_Run_10/FD_Comparison_Tensile_Test.png]

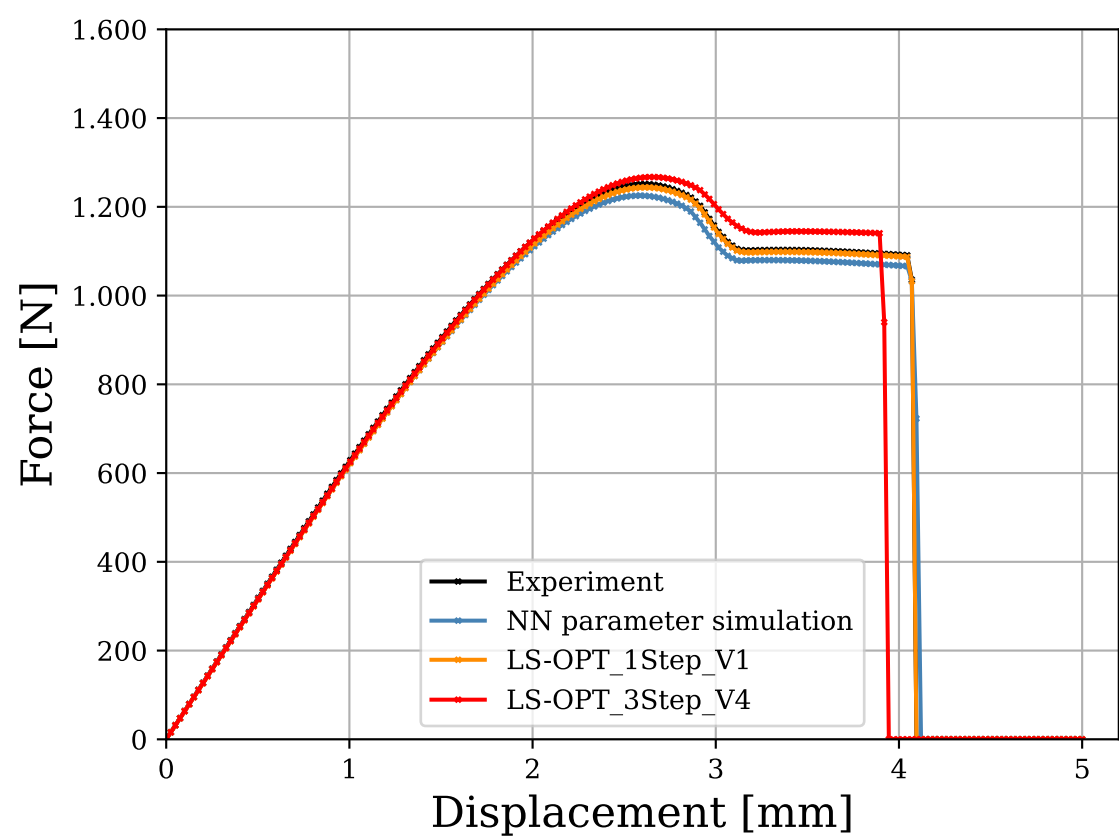

Supplement: Supplementary file 1 [file materials-15-00643-s001.zip › Supplementary_Material/SOC_NN_Pred_LSOPT_Complete/NN_Run_10/FD_Comparison_Tensile_Test_V1.pdf]

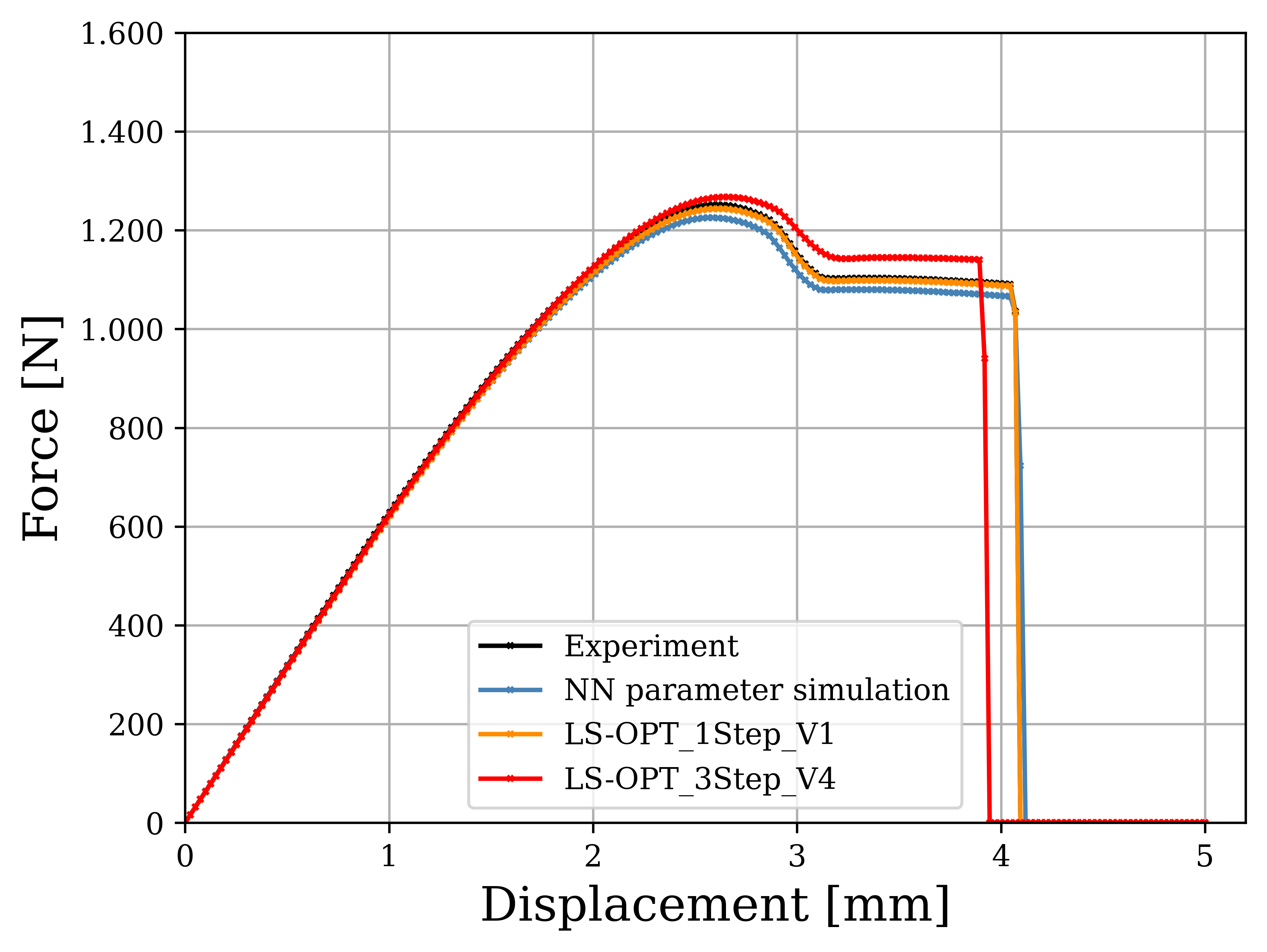

Supplement: Supplementary file 1 [file materials-15-00643-s001.zip › Supplementary_Material/SOC_NN_Pred_LSOPT_Complete/NN_Run_10/FD_Comparison_Tensile_Test_V1.png]

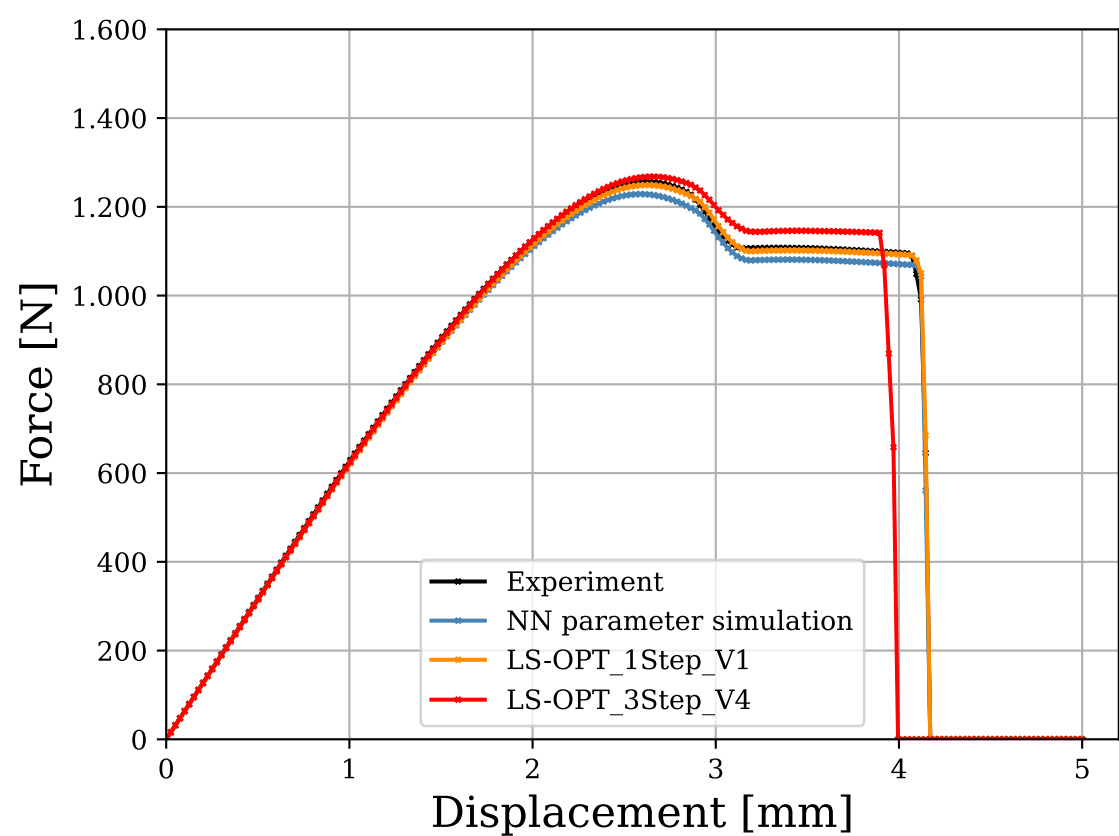

Supplement: Supplementary file 1 [file materials-15-00643-s001.zip › Supplementary_Material/SOC_NN_Pred_LSOPT_Complete/NN_Run_10/FD_Comparison_Tensile_Test_V2.pdf]

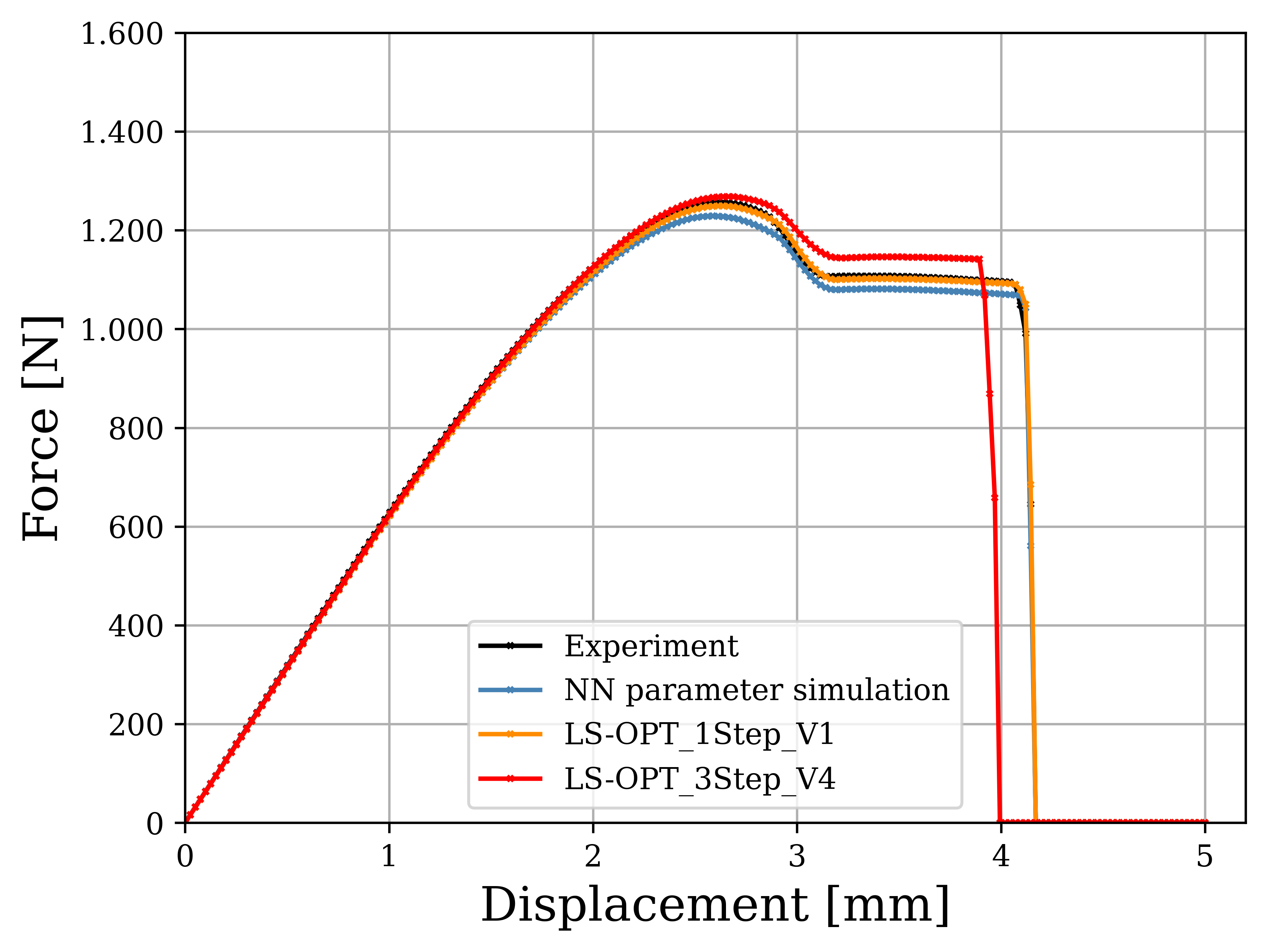

Supplement: Supplementary file 1 [file materials-15-00643-s001.zip › Supplementary_Material/SOC_NN_Pred_LSOPT_Complete/NN_Run_10/FD_Comparison_Tensile_Test_V2.png]

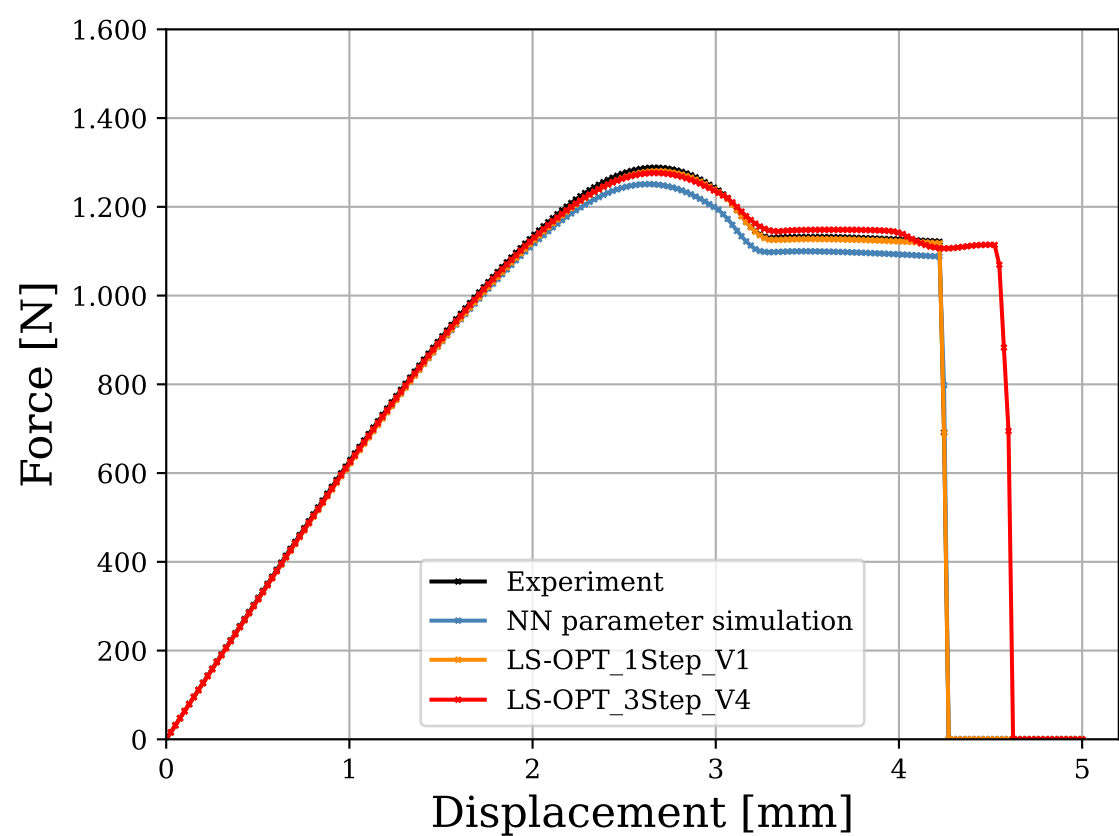

Supplement: Supplementary file 1 [file materials-15-00643-s001.zip › Supplementary_Material/SOC_NN_Pred_LSOPT_Complete/NN_Run_10/FD_Comparison_Tensile_Test_V3.pdf]

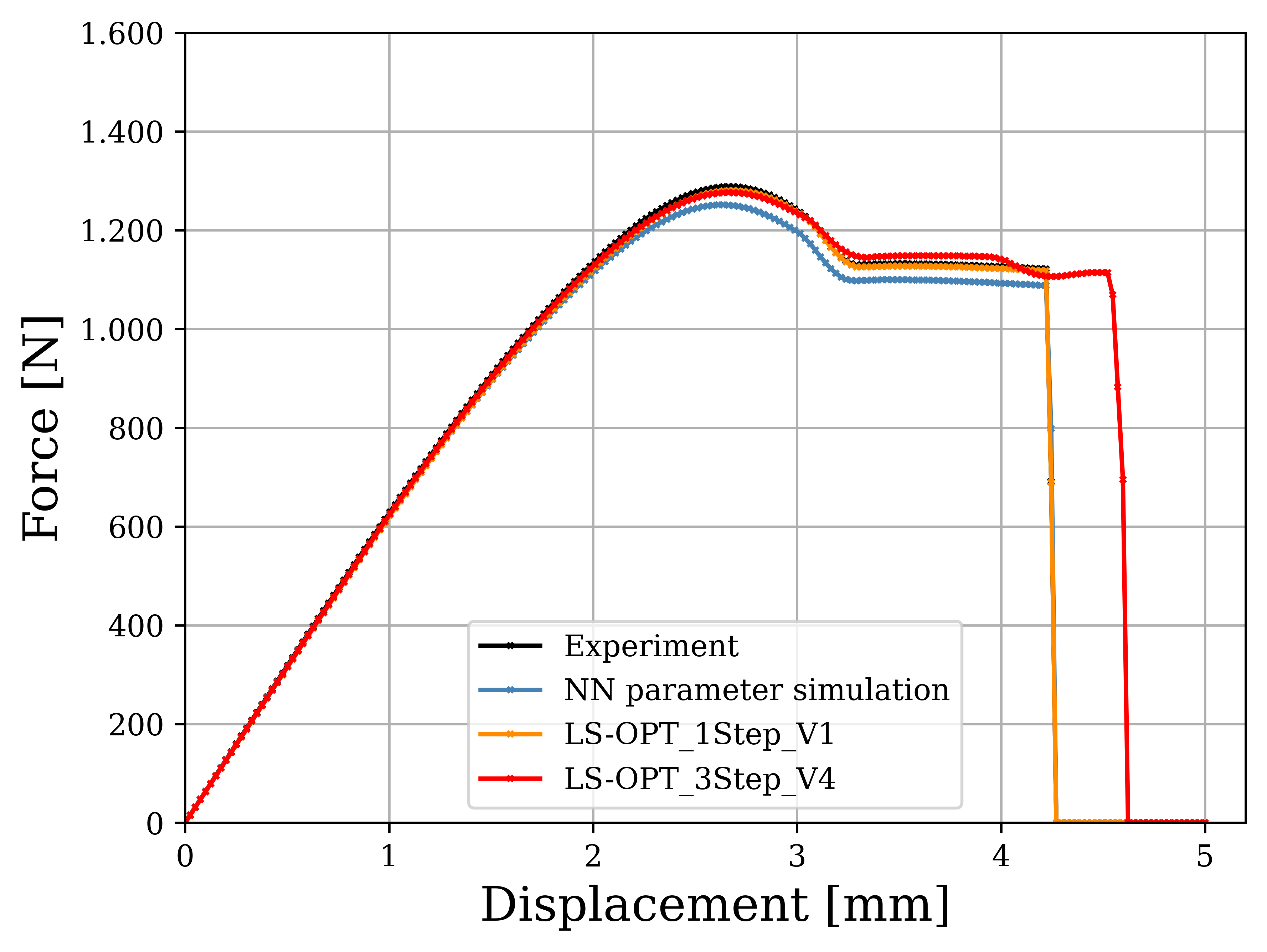

Supplement: Supplementary file 1 [file materials-15-00643-s001.zip › Supplementary_Material/SOC_NN_Pred_LSOPT_Complete/NN_Run_10/FD_Comparison_Tensile_Test_V3.png]

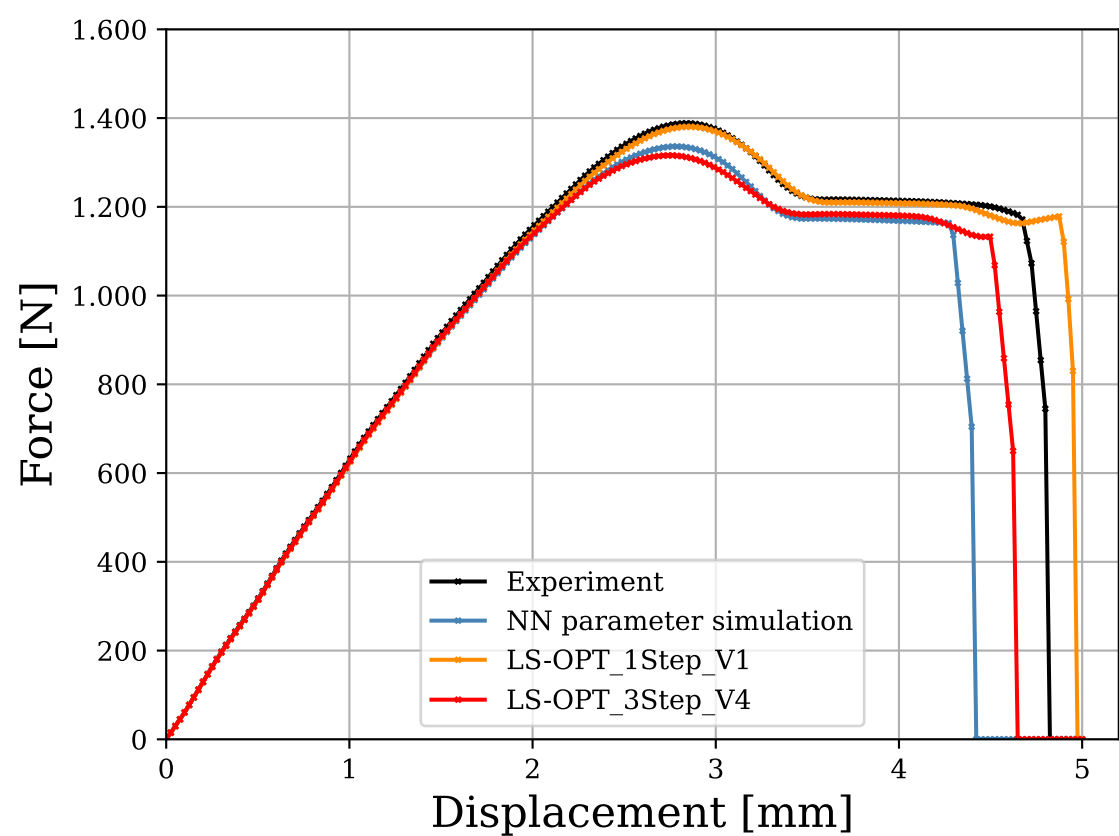

Supplement: Supplementary file 1 [file materials-15-00643-s001.zip › Supplementary_Material/SOC_NN_Pred_LSOPT_Complete/NN_Run_10/FD_Comparison_Tensile_Test_V4.pdf]

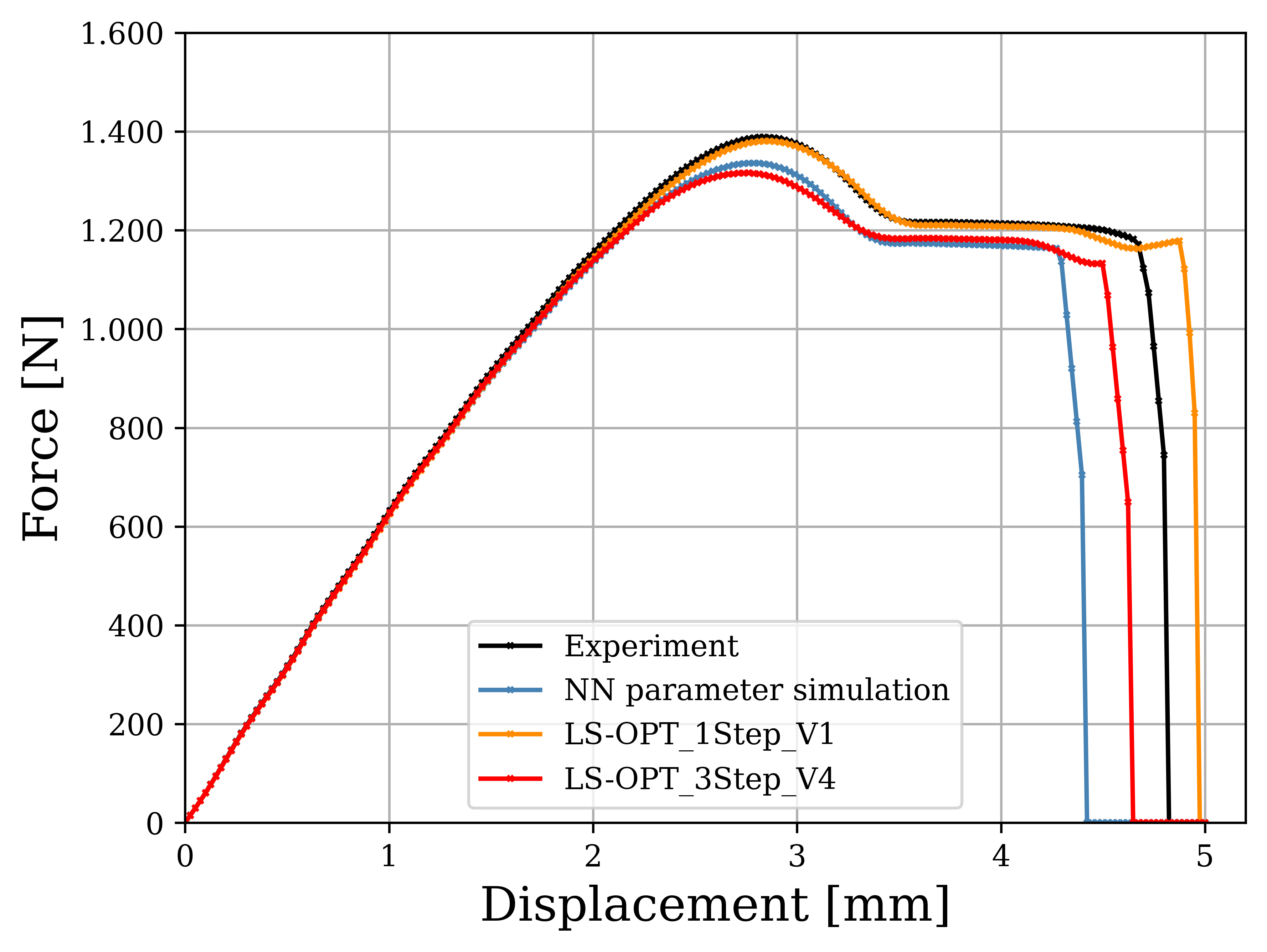

Supplement: Supplementary file 1 [file materials-15-00643-s001.zip › Supplementary_Material/SOC_NN_Pred_LSOPT_Complete/NN_Run_10/FD_Comparison_Tensile_Test_V4.png]

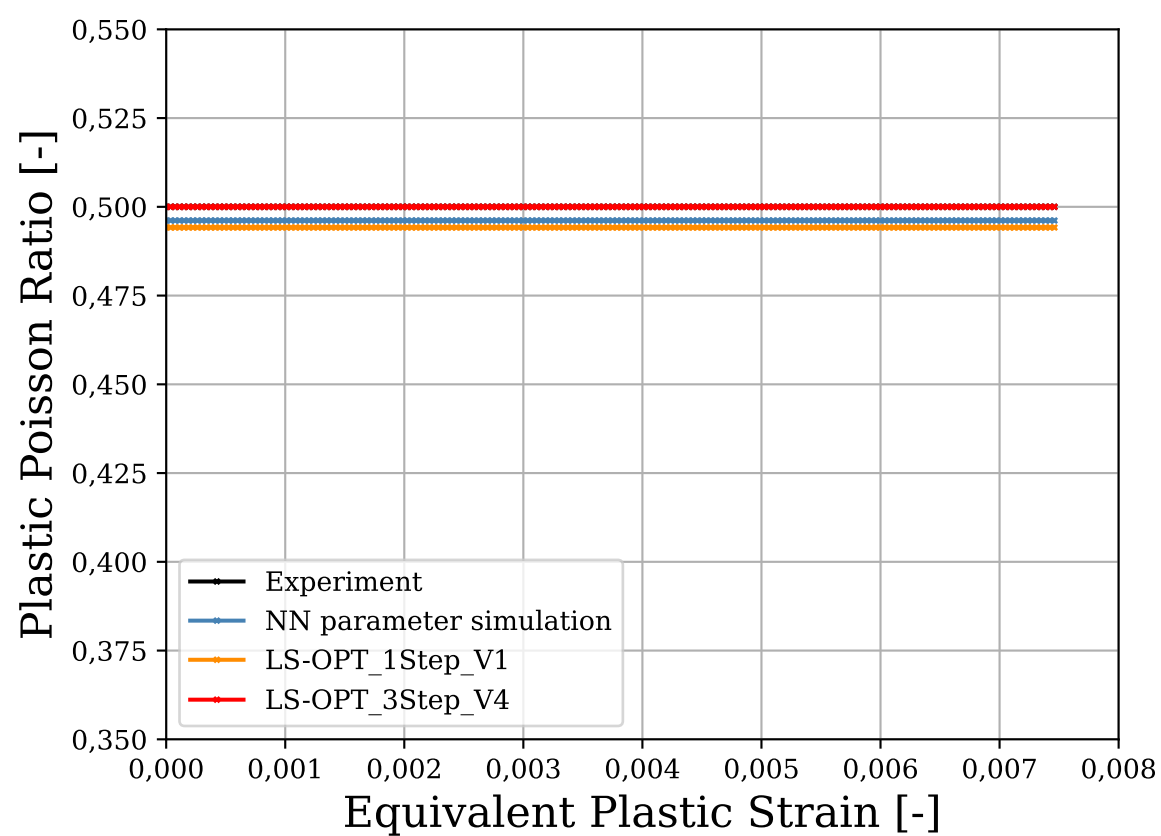

Supplement: Supplementary file 1 [file materials-15-00643-s001.zip › Supplementary_Material/SOC_NN_Pred_LSOPT_Complete/NN_Run_10/PE_Comparison_Compression_Test.pdf]

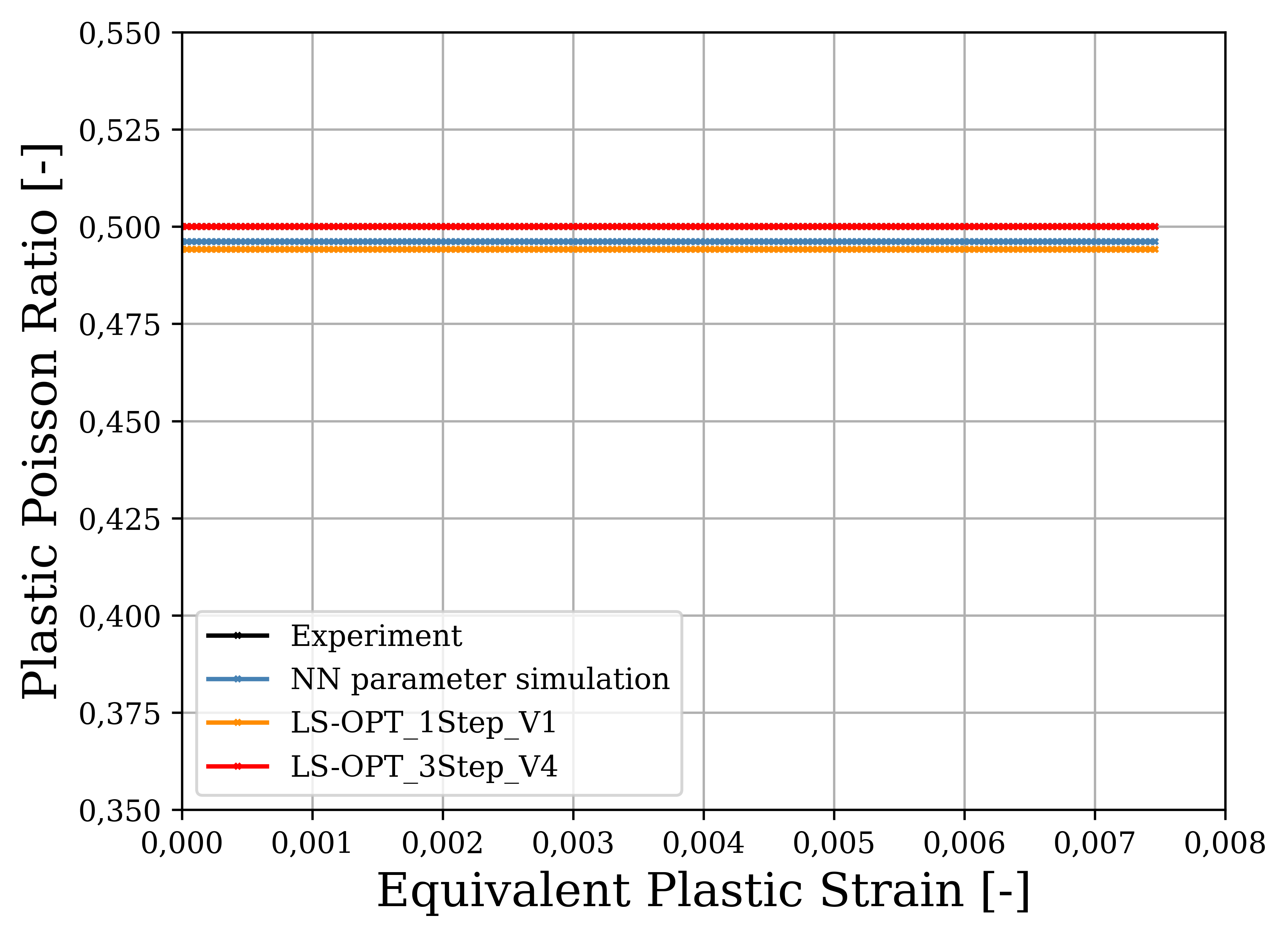

Supplement: Supplementary file 1 [file materials-15-00643-s001.zip › Supplementary_Material/SOC_NN_Pred_LSOPT_Complete/NN_Run_10/PE_Comparison_Compression_Test.png]

Plastic Poisson Ratio [-]

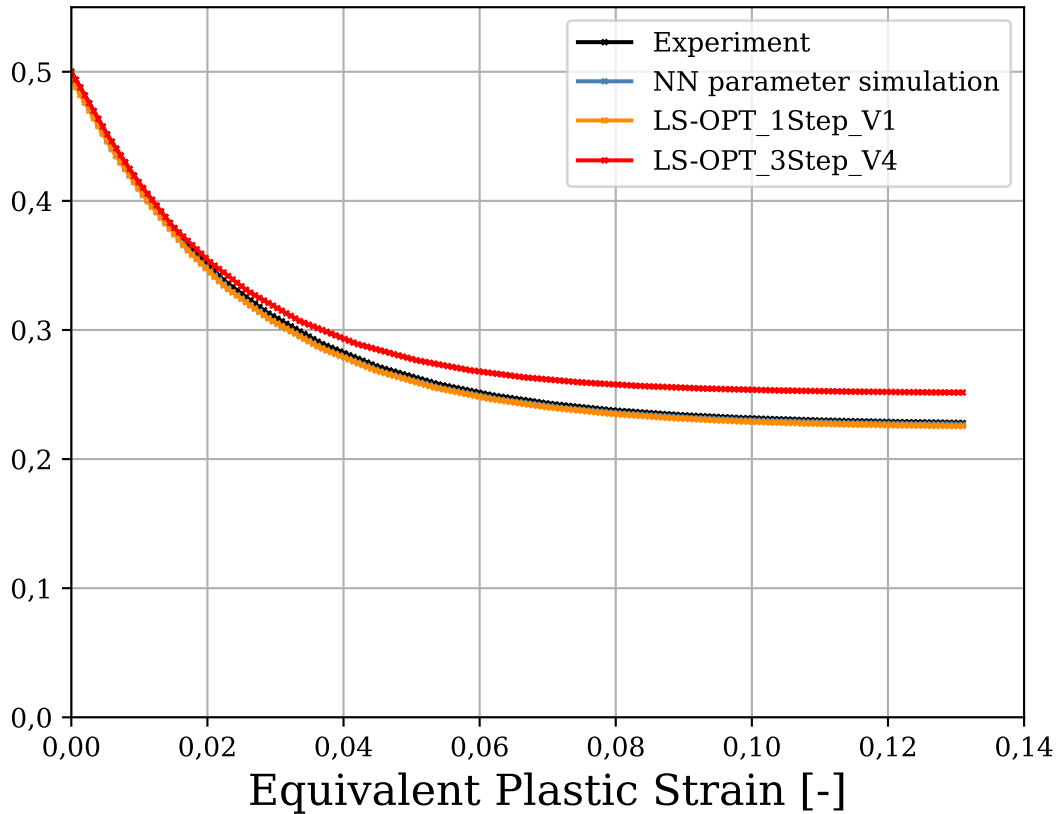

Supplement: Supplementary file 1 [file materials-15-00643-s001.zip › Supplementary_Material/SOC_NN_Pred_LSOPT_Complete/NN_Run_10/PE_Comparison_Punch_Test.pdf]

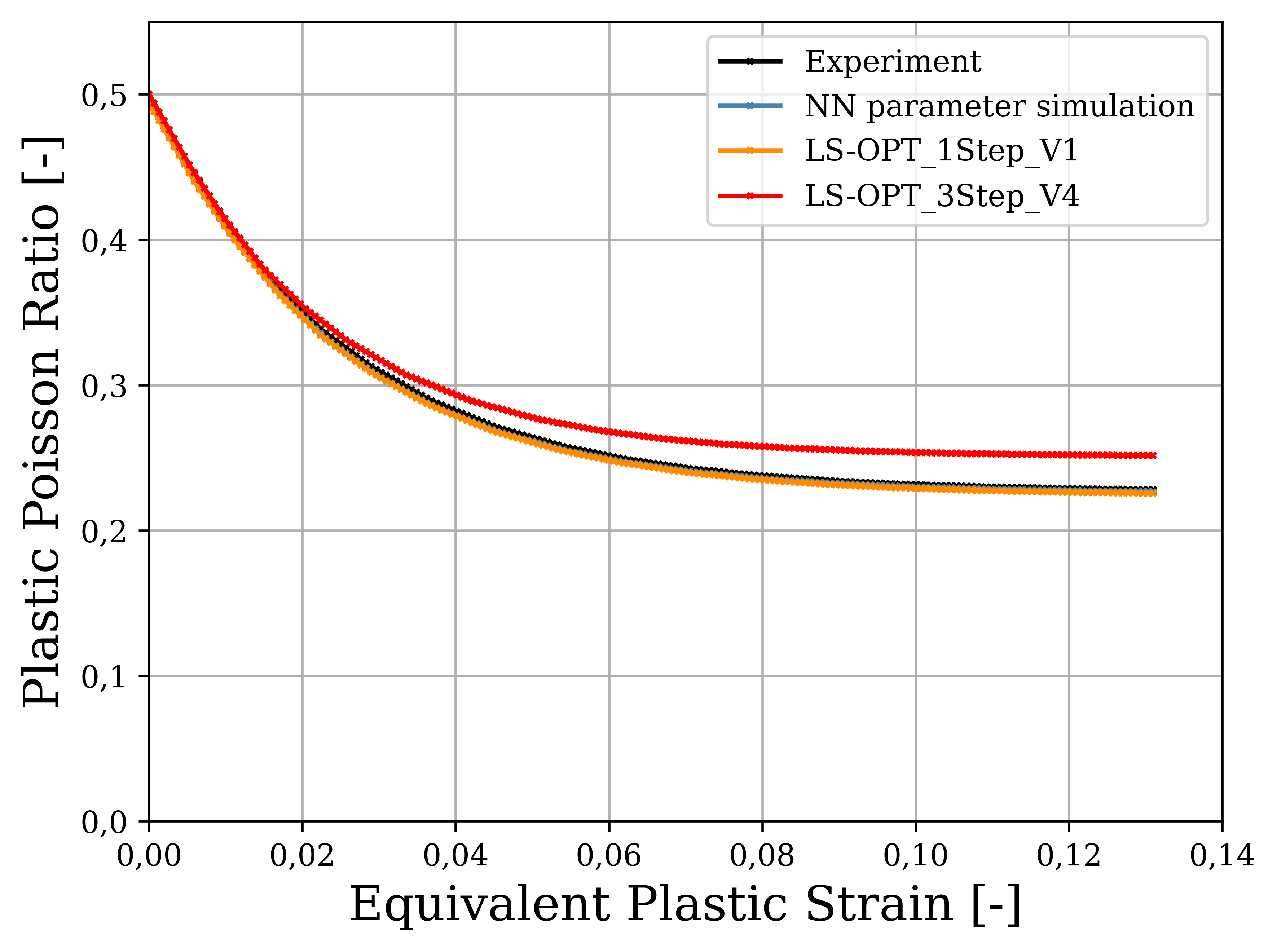

Supplement: Supplementary file 1 [file materials-15-00643-s001.zip › Supplementary_Material/SOC_NN_Pred_LSOPT_Complete/NN_Run_10/PE_Comparison_Punch_Test.png]

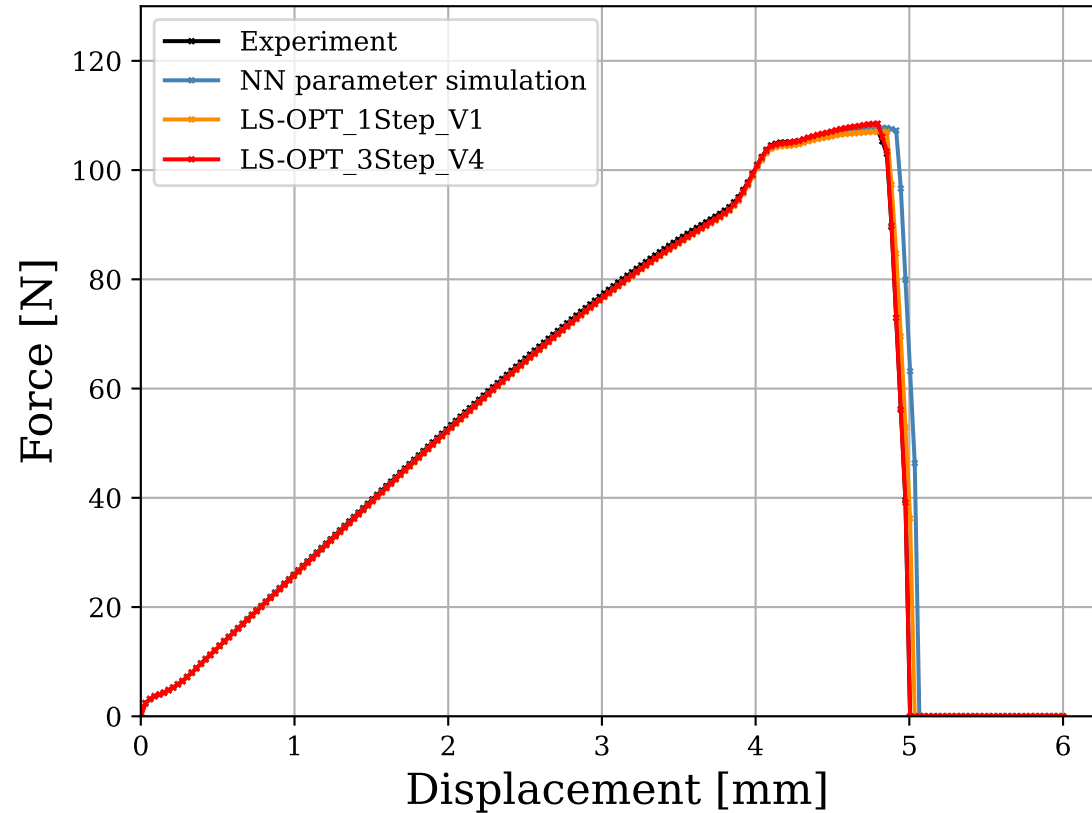

Supplement: Supplementary file 1 [file materials-15-00643-s001.zip › Supplementary_Material/SOC_NN_Pred_LSOPT_Complete/NN_Run_1/FD_Comparison_Bending_Test.pdf]

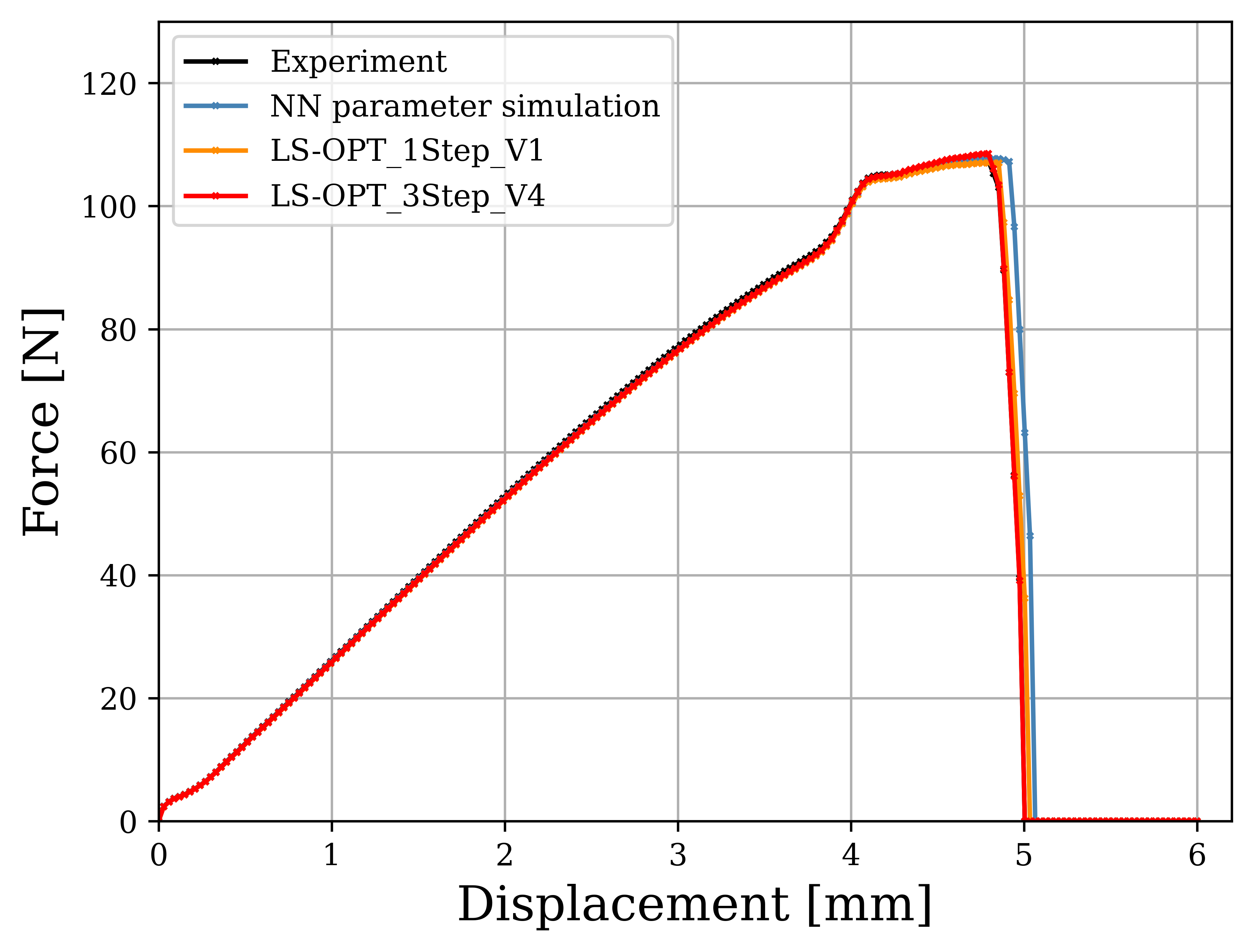

Supplement: Supplementary file 1 [file materials-15-00643-s001.zip › Supplementary_Material/SOC_NN_Pred_LSOPT_Complete/NN_Run_1/FD_Comparison_Bending_Test.png]

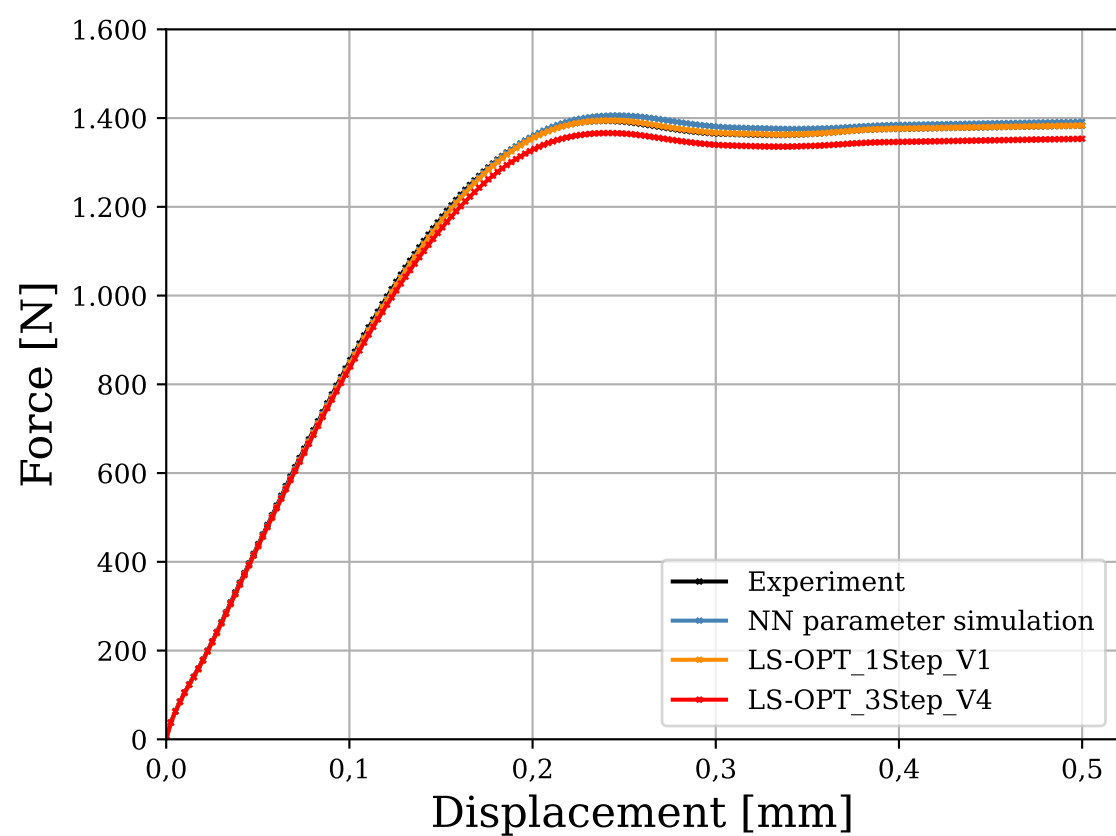

Supplement: Supplementary file 1 [file materials-15-00643-s001.zip › Supplementary_Material/SOC_NN_Pred_LSOPT_Complete/NN_Run_1/FD_Comparison_Compression_Test.pdf]

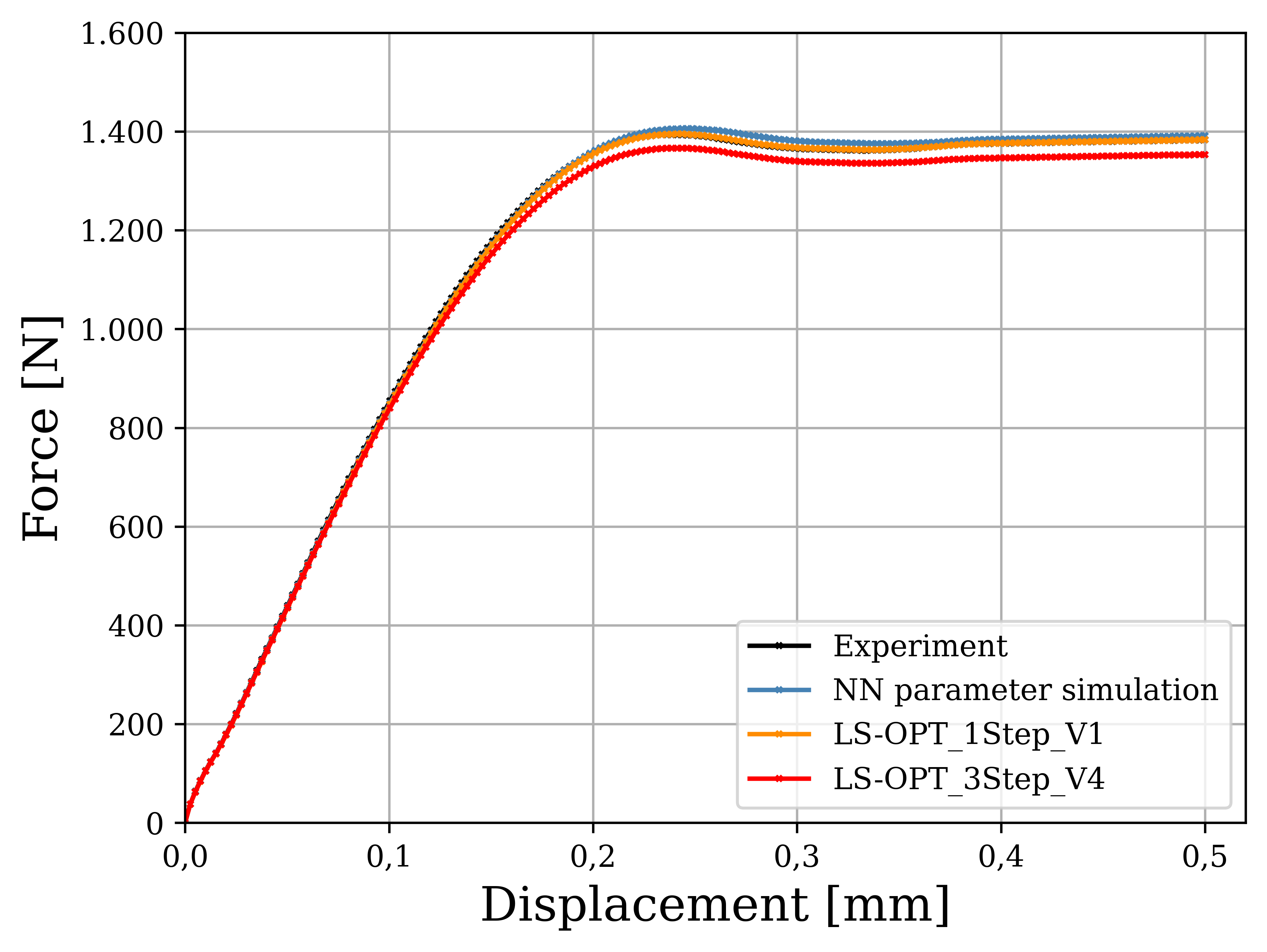

Supplement: Supplementary file 1 [file materials-15-00643-s001.zip › Supplementary_Material/SOC_NN_Pred_LSOPT_Complete/NN_Run_1/FD_Comparison_Compression_Test.png]

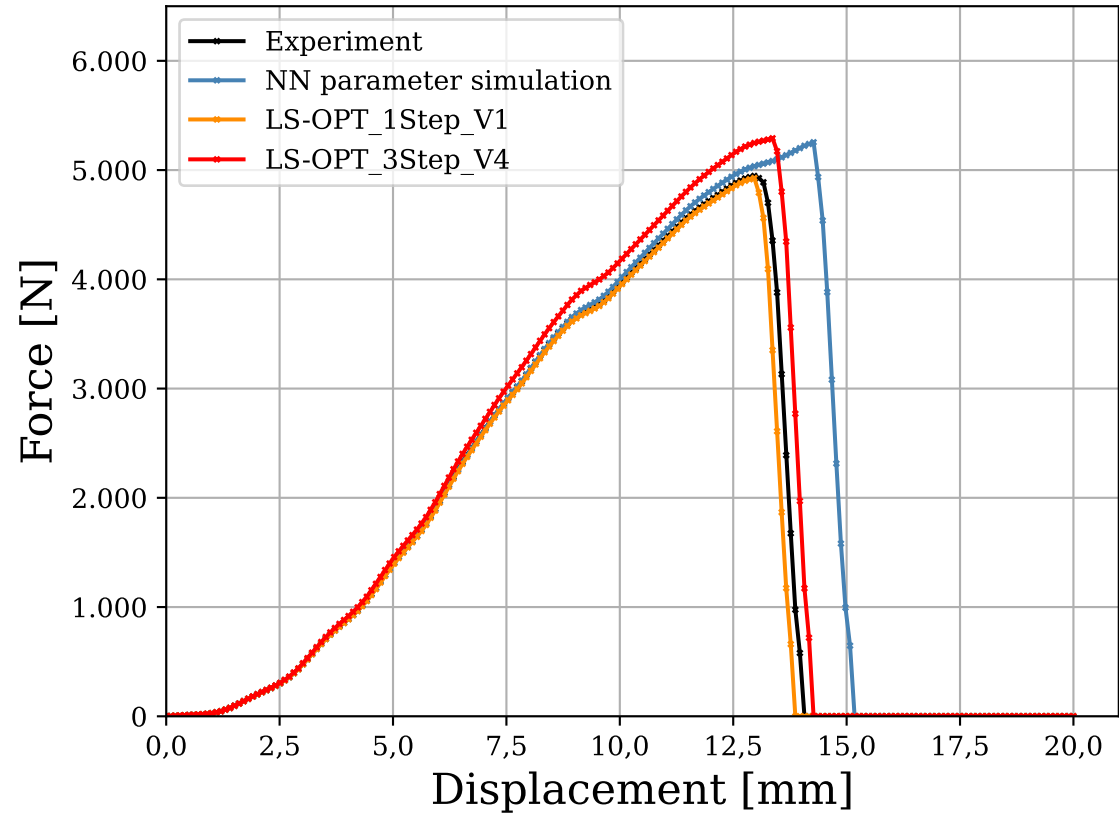

Supplement: Supplementary file 1 [file materials-15-00643-s001.zip › Supplementary_Material/SOC_NN_Pred_LSOPT_Complete/NN_Run_1/FD_Comparison_Punch_Test.pdf]

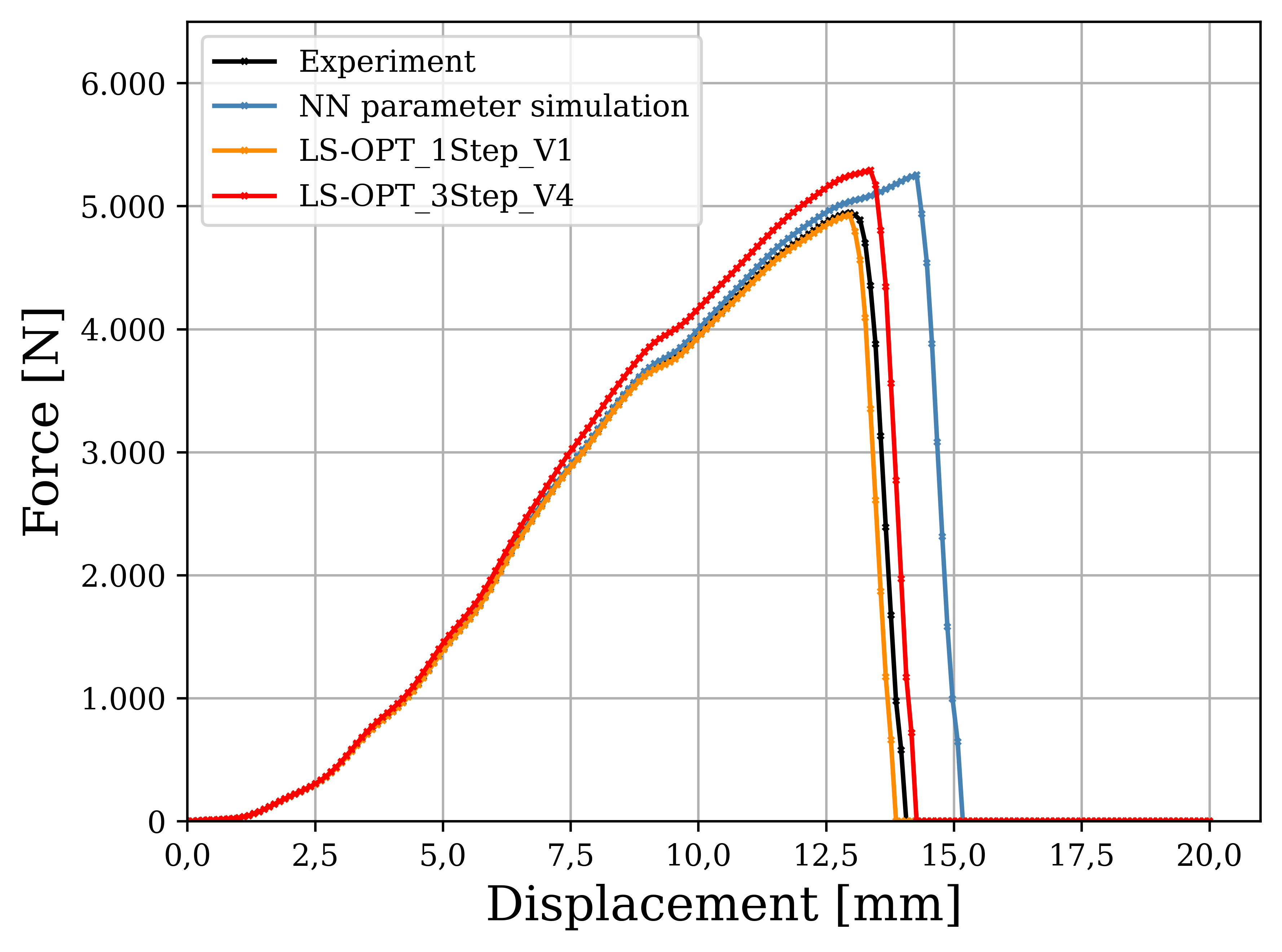

Supplement: Supplementary file 1 [file materials-15-00643-s001.zip › Supplementary_Material/SOC_NN_Pred_LSOPT_Complete/NN_Run_1/FD_Comparison_Punch_Test.png]

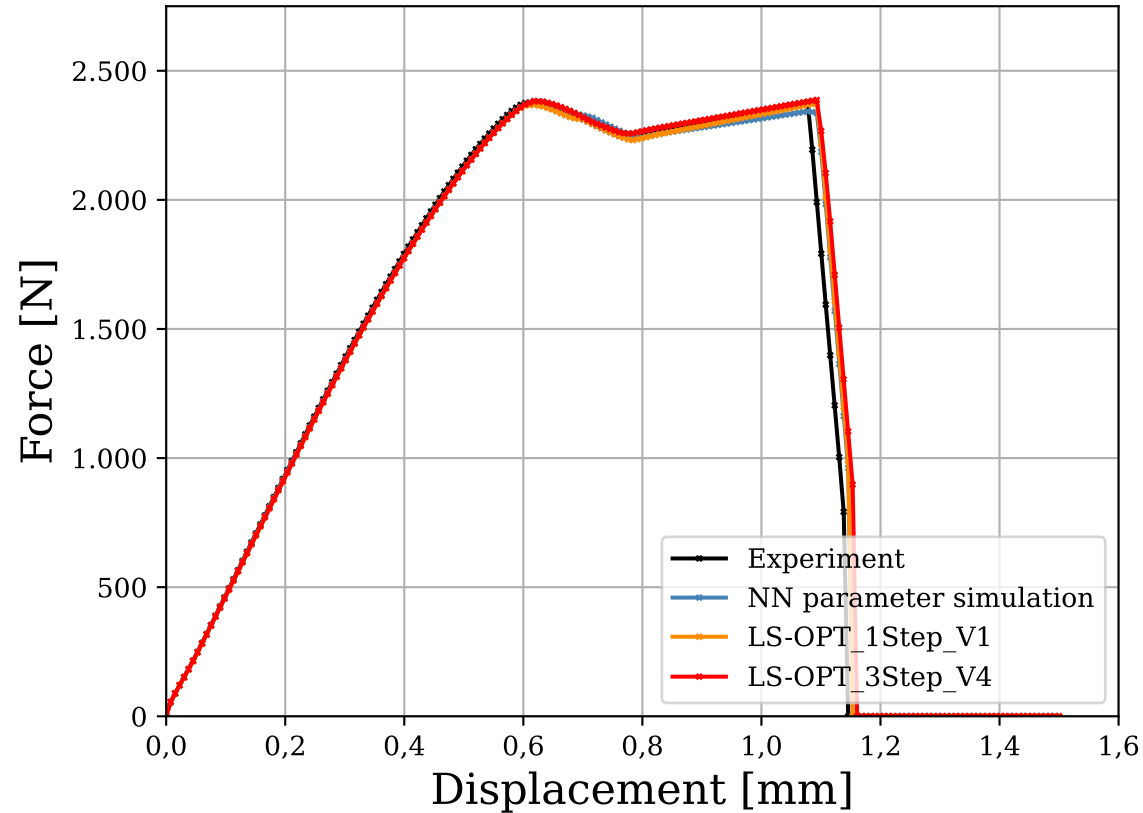

Supplement: Supplementary file 1 [file materials-15-00643-s001.zip › Supplementary_Material/SOC_NN_Pred_LSOPT_Complete/NN_Run_1/FD_Comparison_Shear_ASTM_Test.pdf]

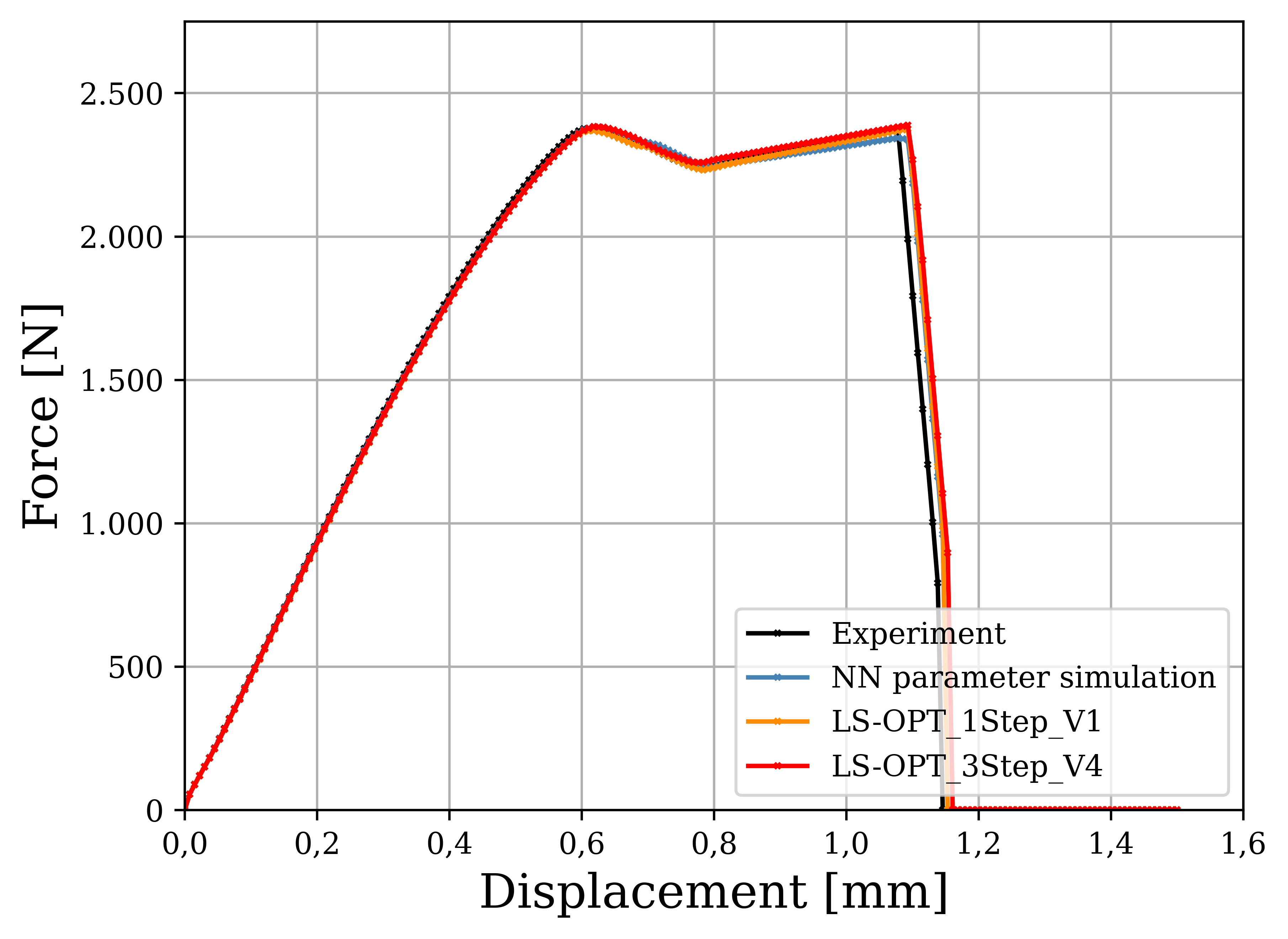

Supplement: Supplementary file 1 [file materials-15-00643-s001.zip › Supplementary_Material/SOC_NN_Pred_LSOPT_Complete/NN_Run_1/FD_Comparison_Shear_ASTM_Test.png]

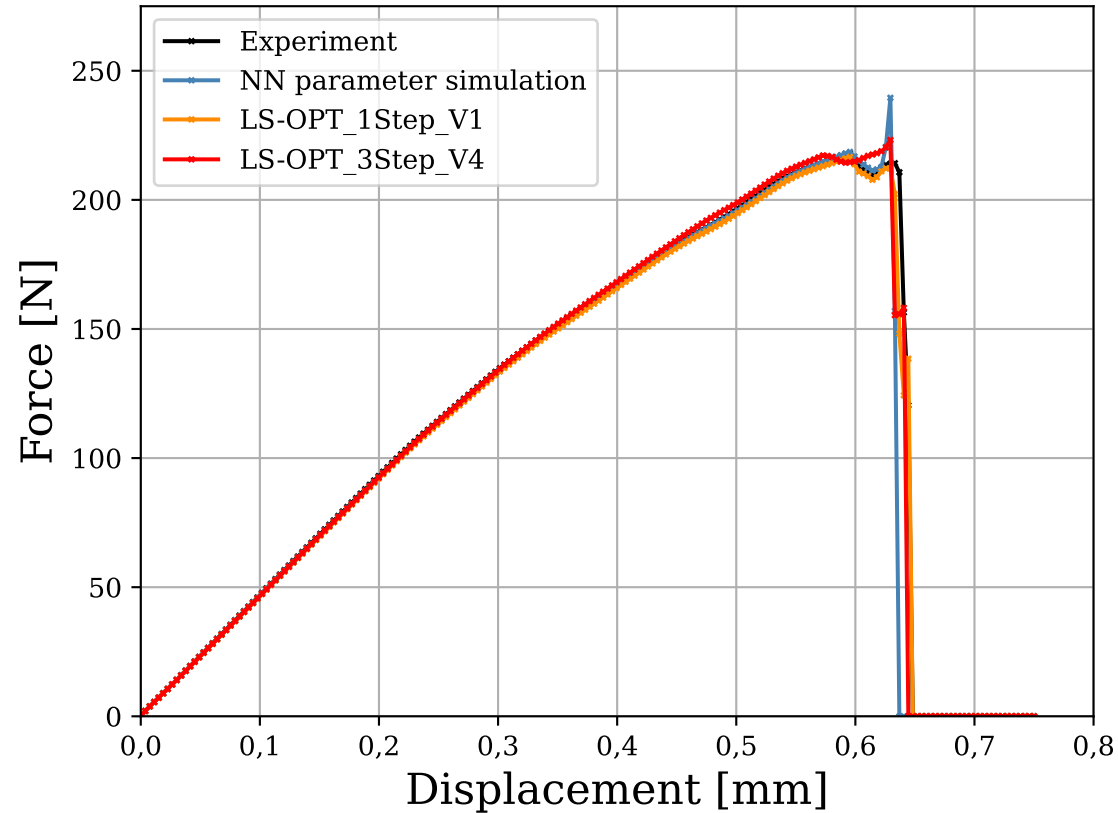

Supplement: Supplementary file 1 [file materials-15-00643-s001.zip › Supplementary_Material/SOC_NN_Pred_LSOPT_Complete/NN_Run_1/FD_Comparison_Shear_Dynamore_Test.pdf]

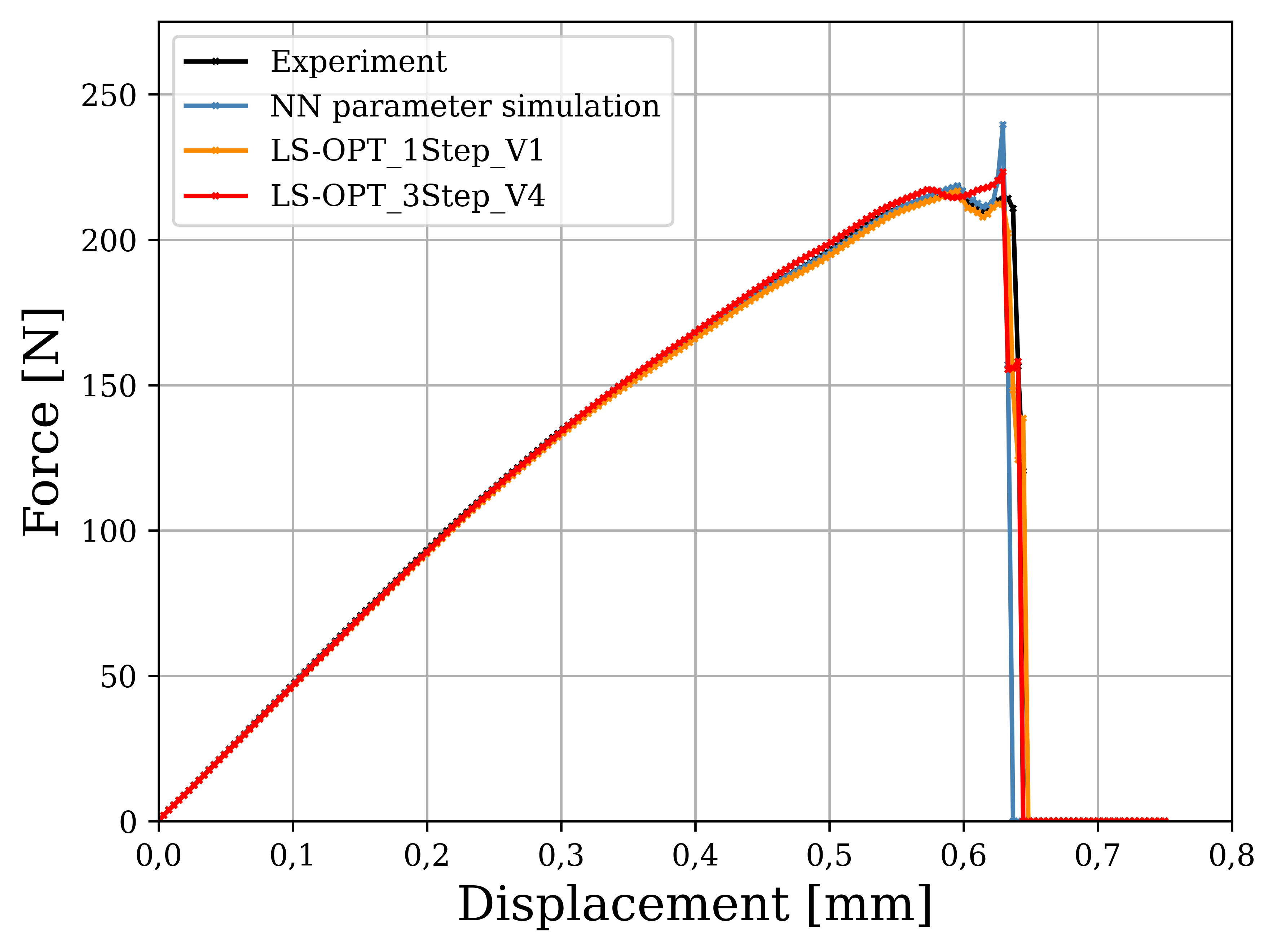

Supplement: Supplementary file 1 [file materials-15-00643-s001.zip › Supplementary_Material/SOC_NN_Pred_LSOPT_Complete/NN_Run_1/FD_Comparison_Shear_Dynamore_Test.png]

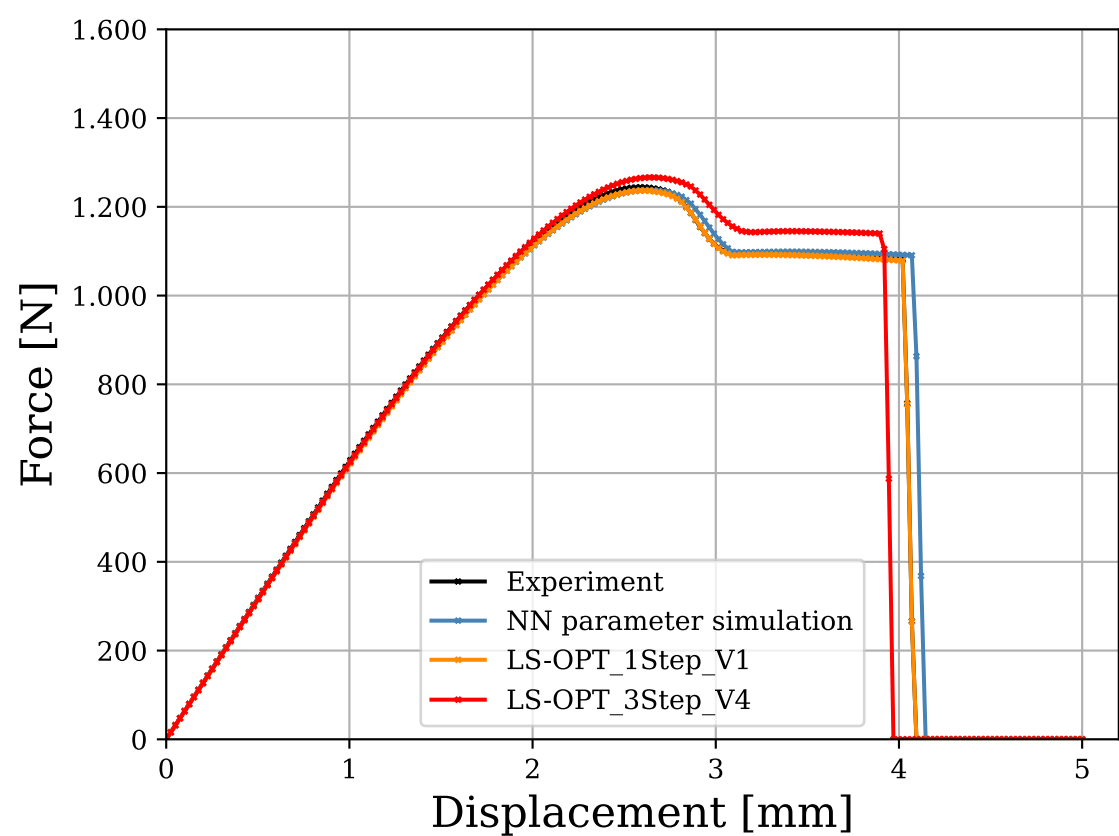

Supplement: Supplementary file 1 [file materials-15-00643-s001.zip › Supplementary_Material/SOC_NN_Pred_LSOPT_Complete/NN_Run_1/FD_Comparison_Tensile_Test.pdf]

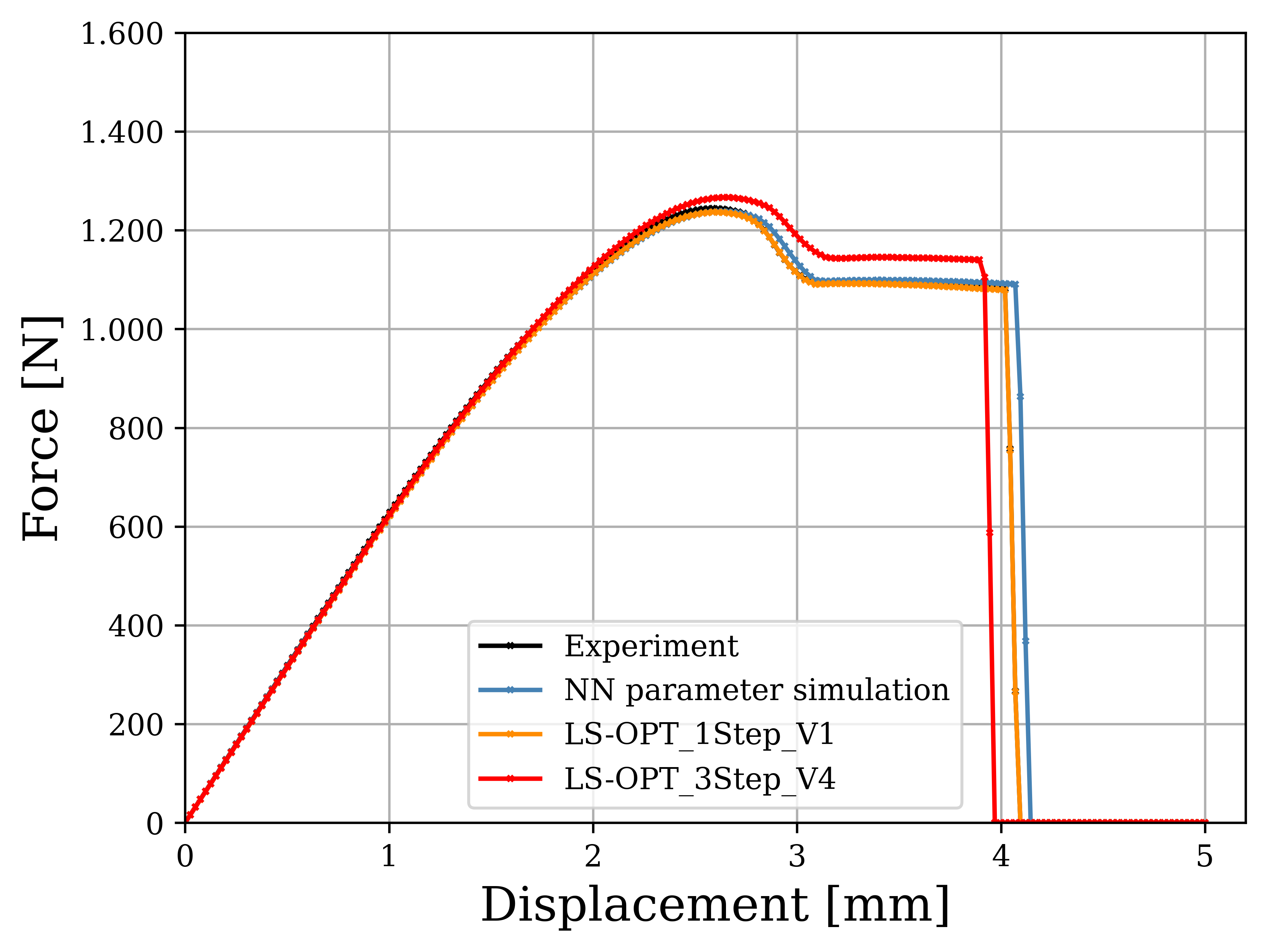

Supplement: Supplementary file 1 [file materials-15-00643-s001.zip › Supplementary_Material/SOC_NN_Pred_LSOPT_Complete/NN_Run_1/FD_Comparison_Tensile_Test.png]

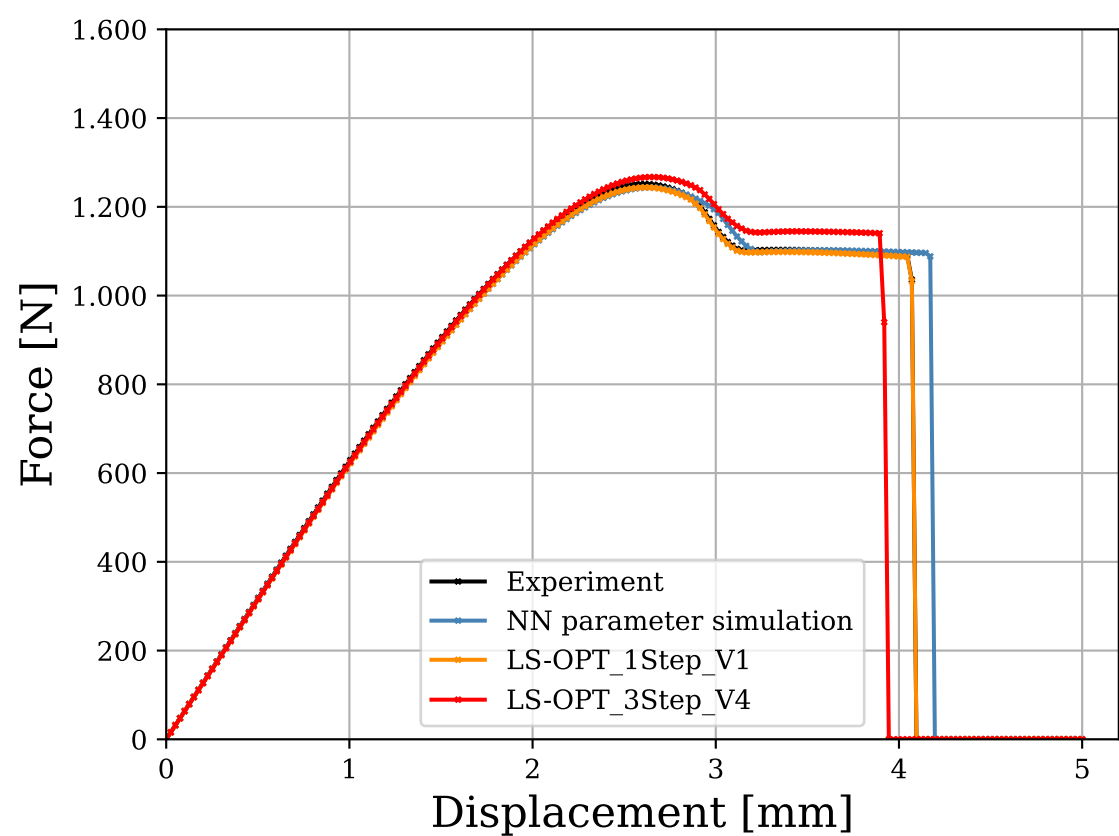

Supplement: Supplementary file 1 [file materials-15-00643-s001.zip › Supplementary_Material/SOC_NN_Pred_LSOPT_Complete/NN_Run_1/FD_Comparison_Tensile_Test_V1.pdf]

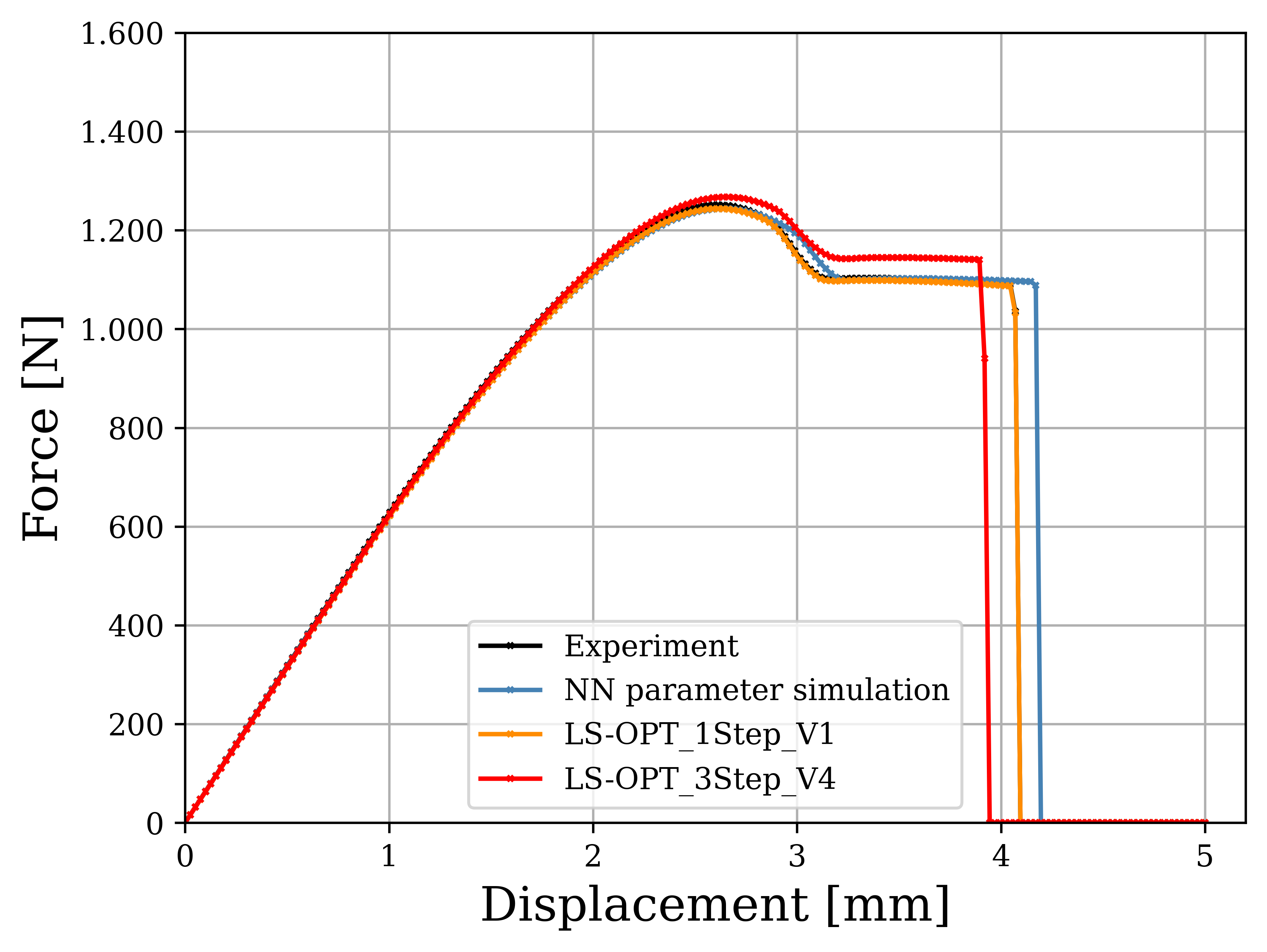

Supplement: Supplementary file 1 [file materials-15-00643-s001.zip › Supplementary_Material/SOC_NN_Pred_LSOPT_Complete/NN_Run_1/FD_Comparison_Tensile_Test_V1.png]

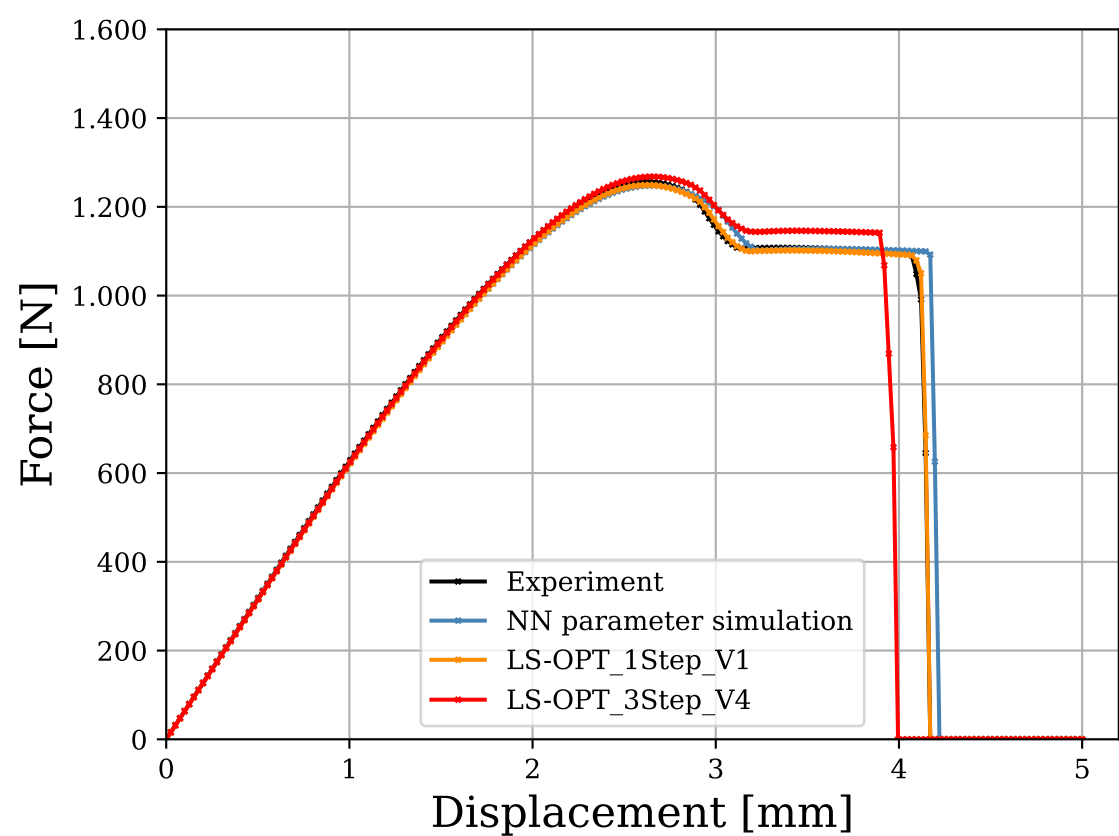

Supplement: Supplementary file 1 [file materials-15-00643-s001.zip › Supplementary_Material/SOC_NN_Pred_LSOPT_Complete/NN_Run_1/FD_Comparison_Tensile_Test_V2.pdf]

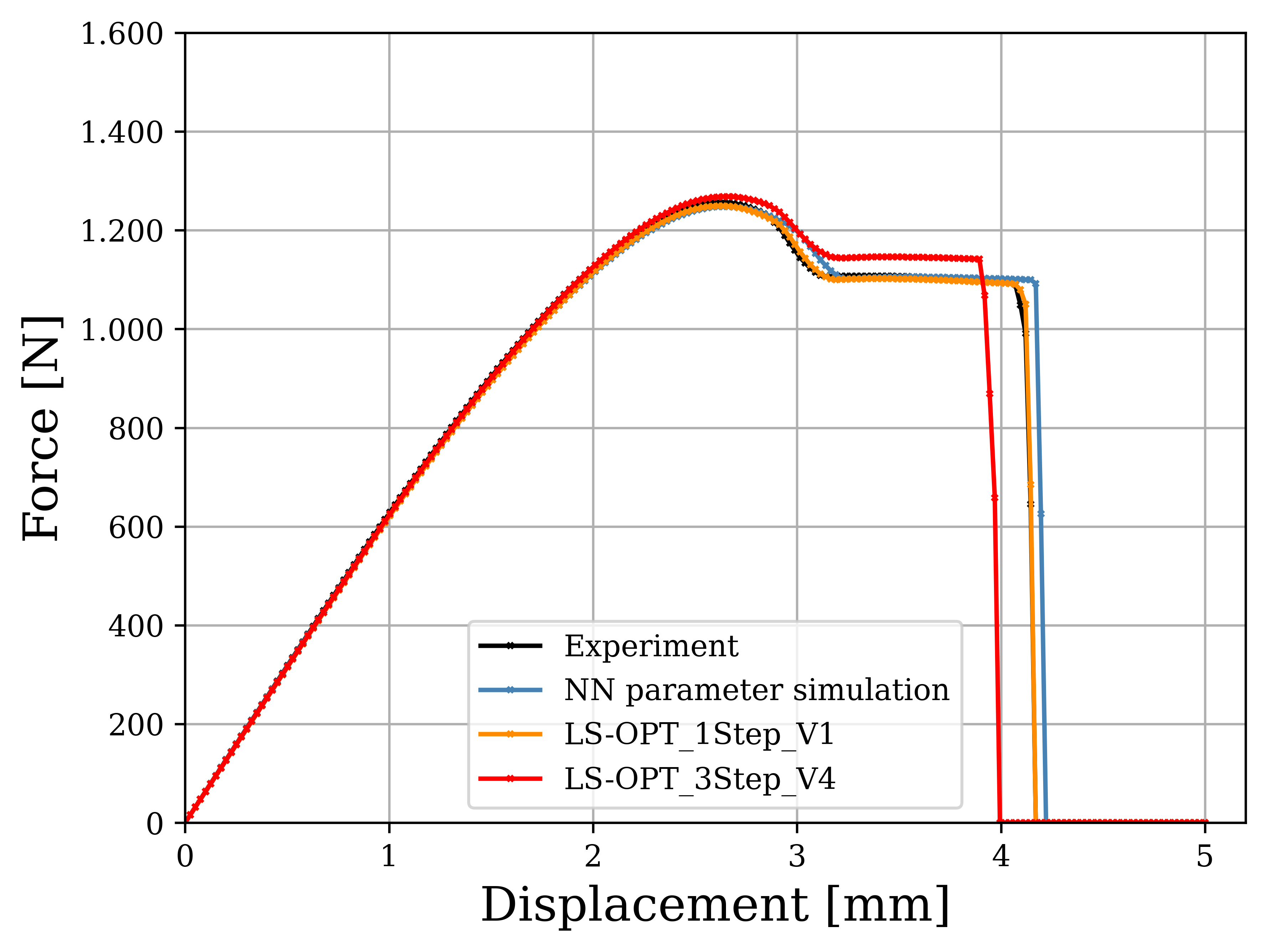

Supplement: Supplementary file 1 [file materials-15-00643-s001.zip › Supplementary_Material/SOC_NN_Pred_LSOPT_Complete/NN_Run_1/FD_Comparison_Tensile_Test_V2.png]

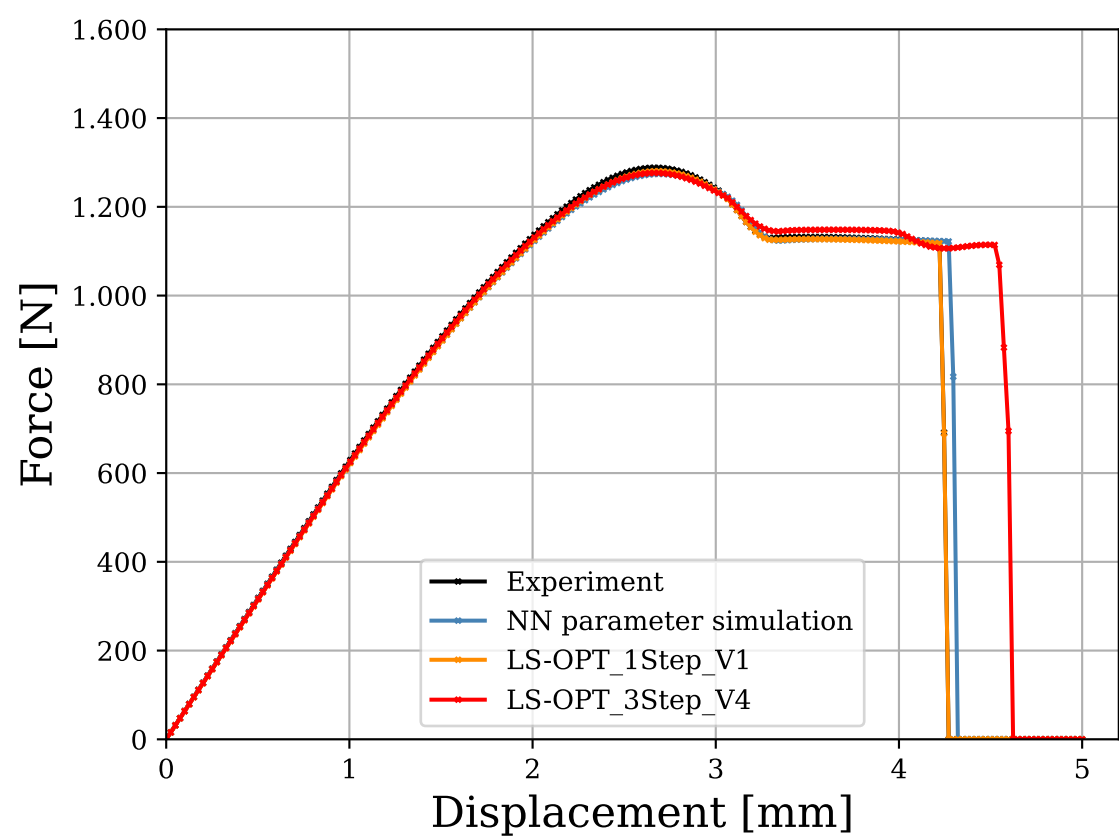

Supplement: Supplementary file 1 [file materials-15-00643-s001.zip › Supplementary_Material/SOC_NN_Pred_LSOPT_Complete/NN_Run_1/FD_Comparison_Tensile_Test_V3.pdf]

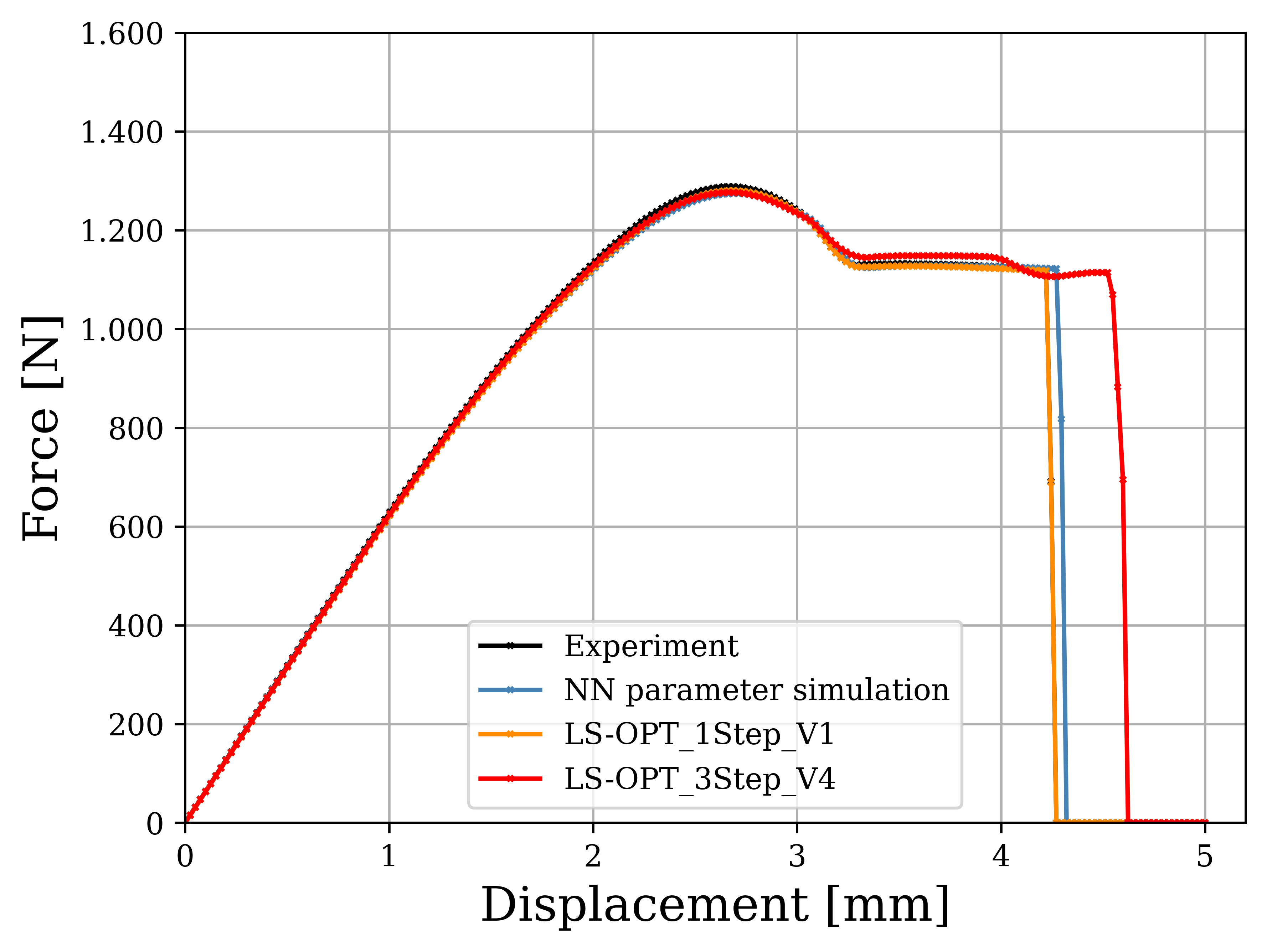

Supplement: Supplementary file 1 [file materials-15-00643-s001.zip › Supplementary_Material/SOC_NN_Pred_LSOPT_Complete/NN_Run_1/FD_Comparison_Tensile_Test_V3.png]

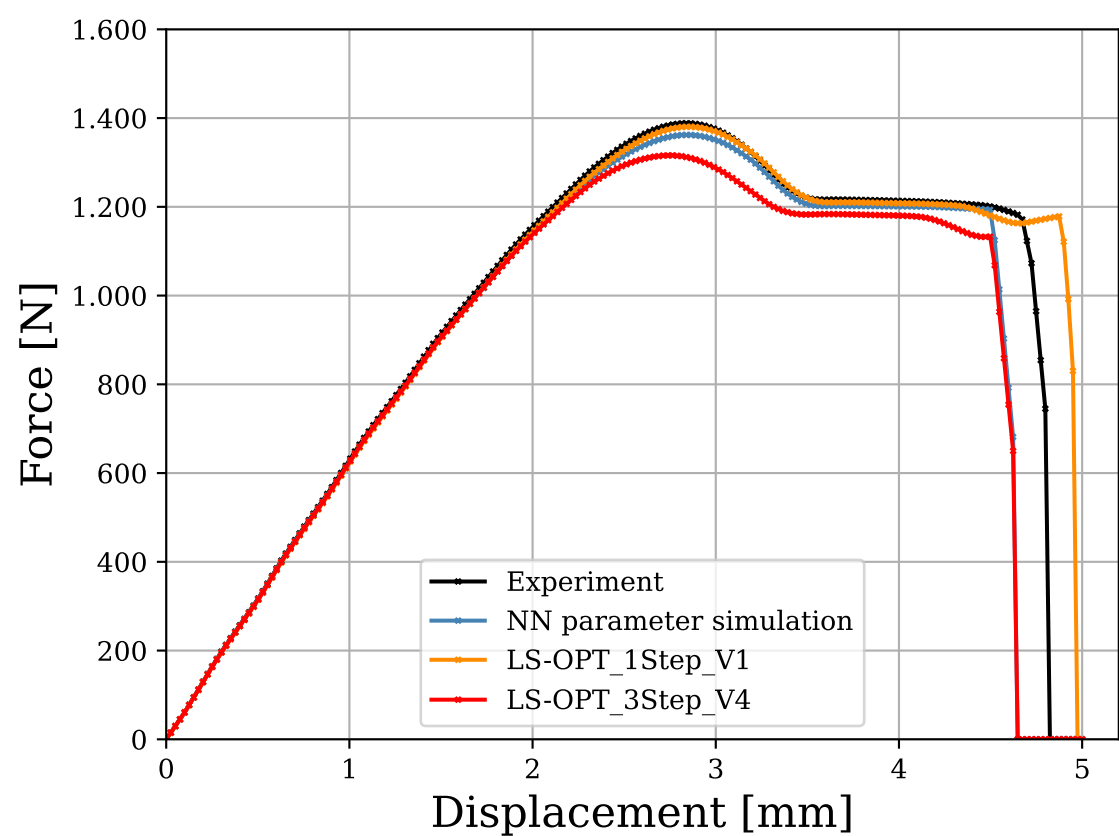

Supplement: Supplementary file 1 [file materials-15-00643-s001.zip › Supplementary_Material/SOC_NN_Pred_LSOPT_Complete/NN_Run_1/FD_Comparison_Tensile_Test_V4.pdf]

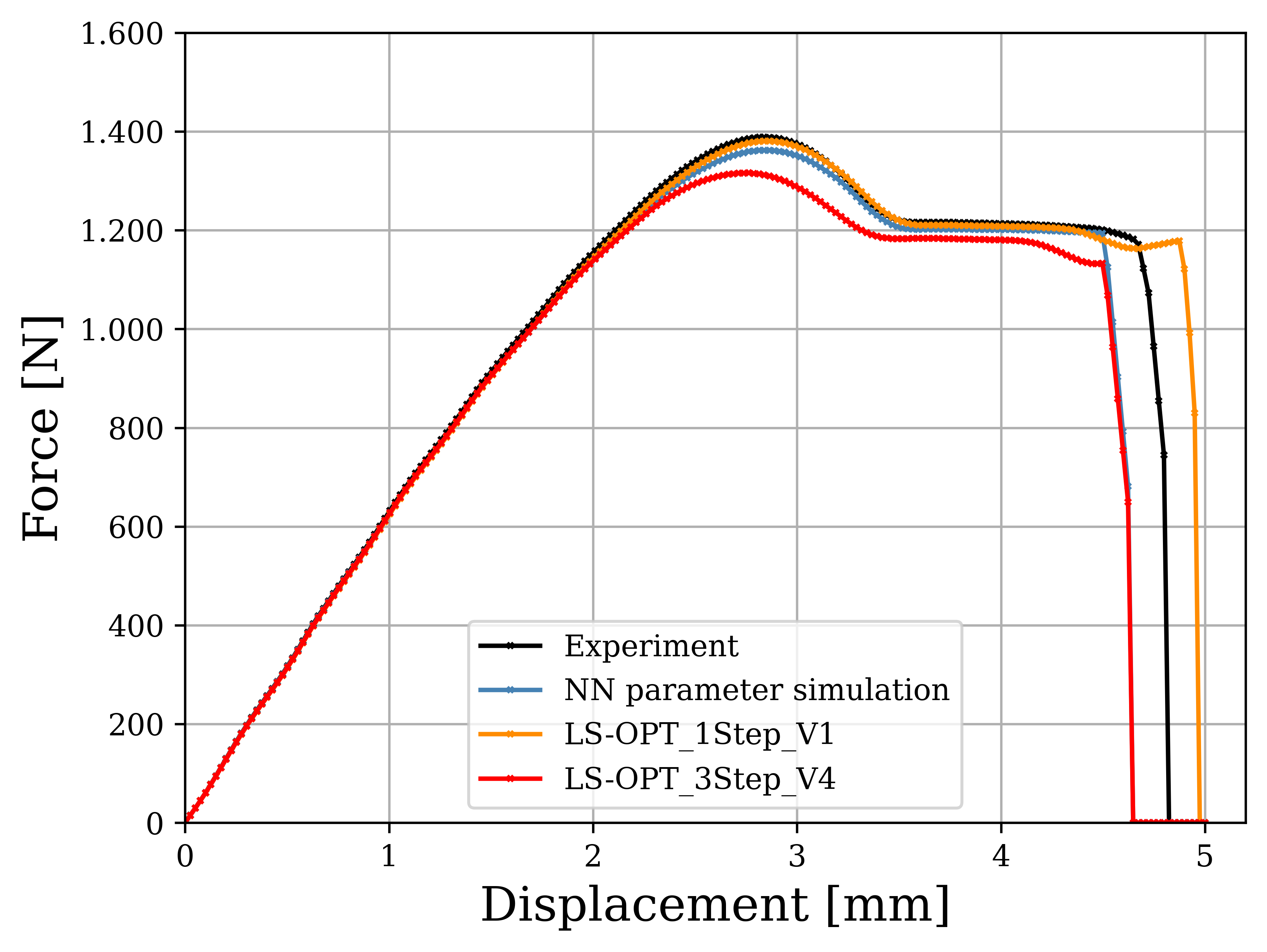

Supplement: Supplementary file 1 [file materials-15-00643-s001.zip › Supplementary_Material/SOC_NN_Pred_LSOPT_Complete/NN_Run_1/FD_Comparison_Tensile_Test_V4.png]

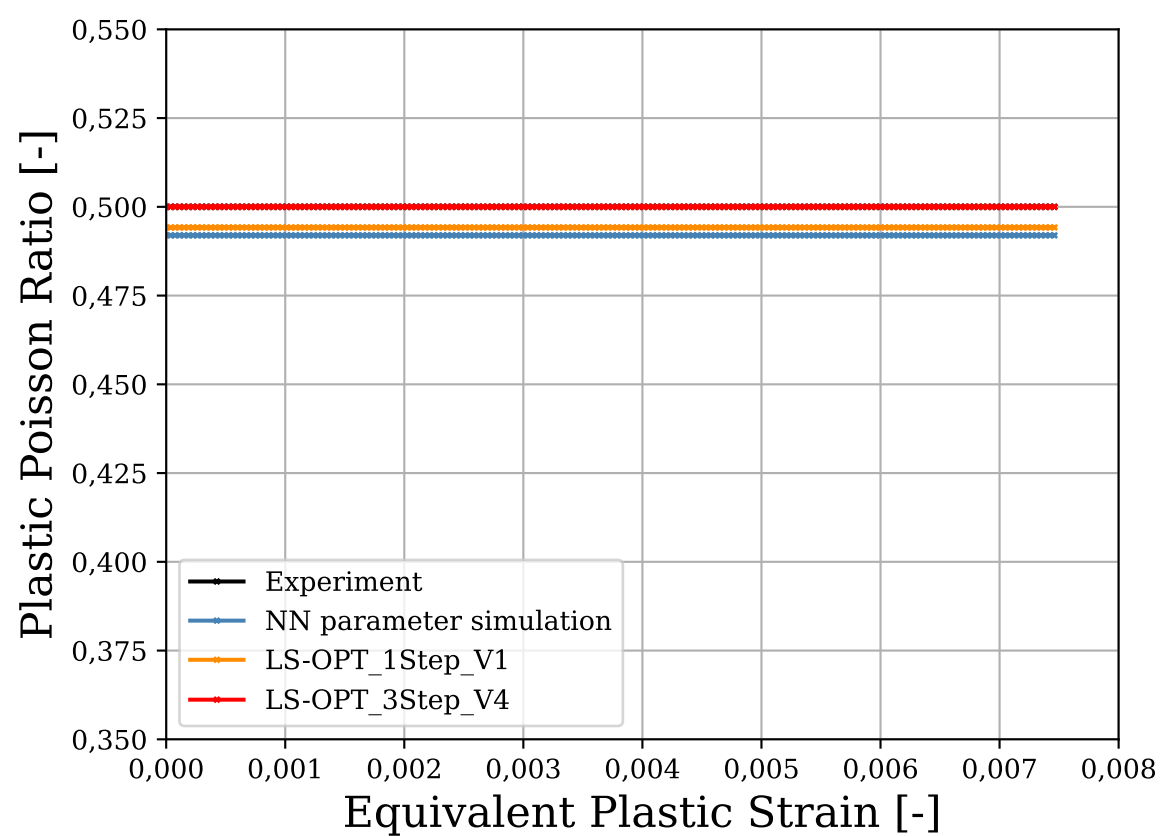

Supplement: Supplementary file 1 [file materials-15-00643-s001.zip › Supplementary_Material/SOC_NN_Pred_LSOPT_Complete/NN_Run_1/PE_Comparison_Compression_Test.pdf]

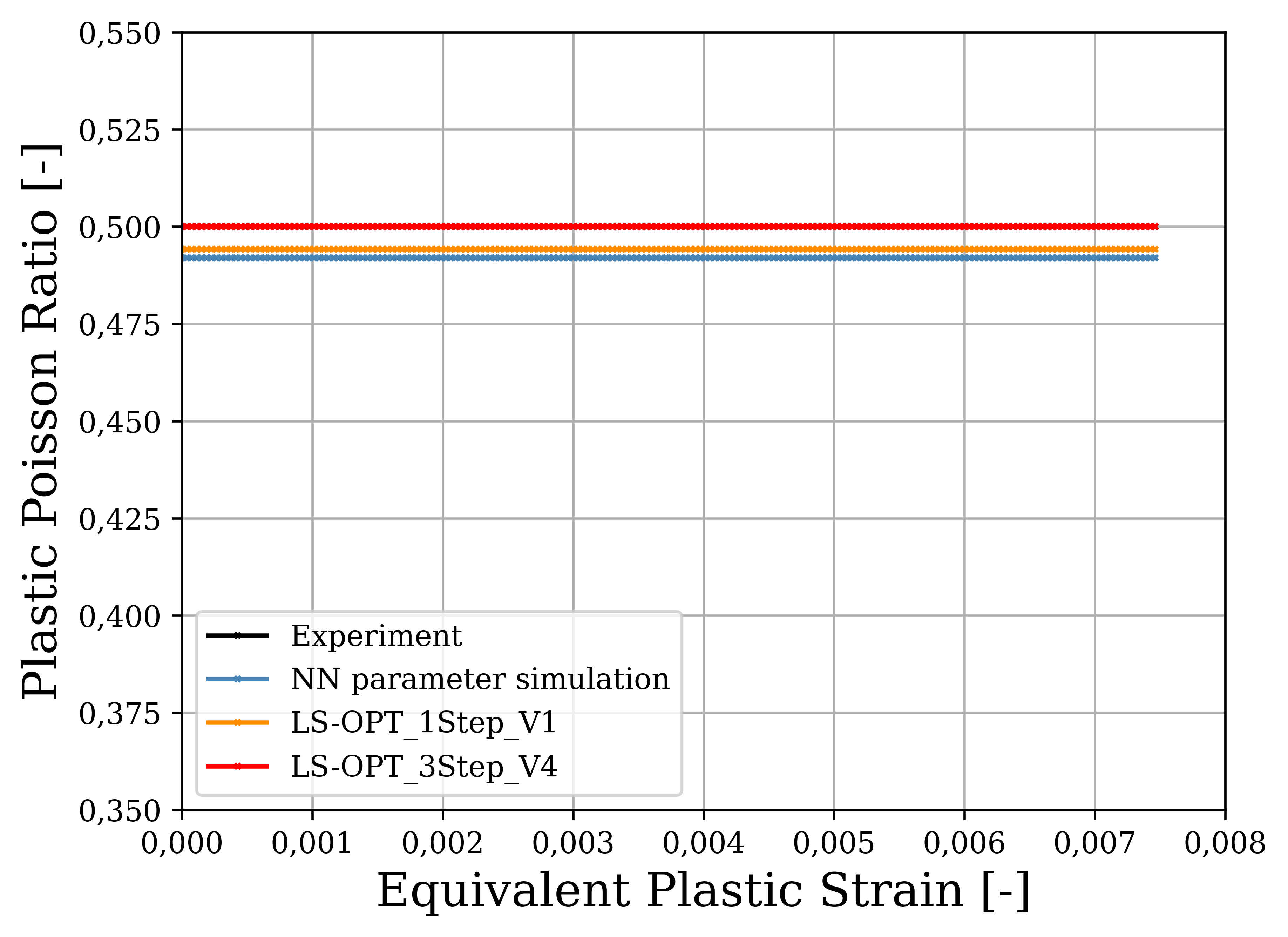

Supplement: Supplementary file 1 [file materials-15-00643-s001.zip › Supplementary_Material/SOC_NN_Pred_LSOPT_Complete/NN_Run_1/PE_Comparison_Compression_Test.png]

Plastic Poisson Ratio [-]

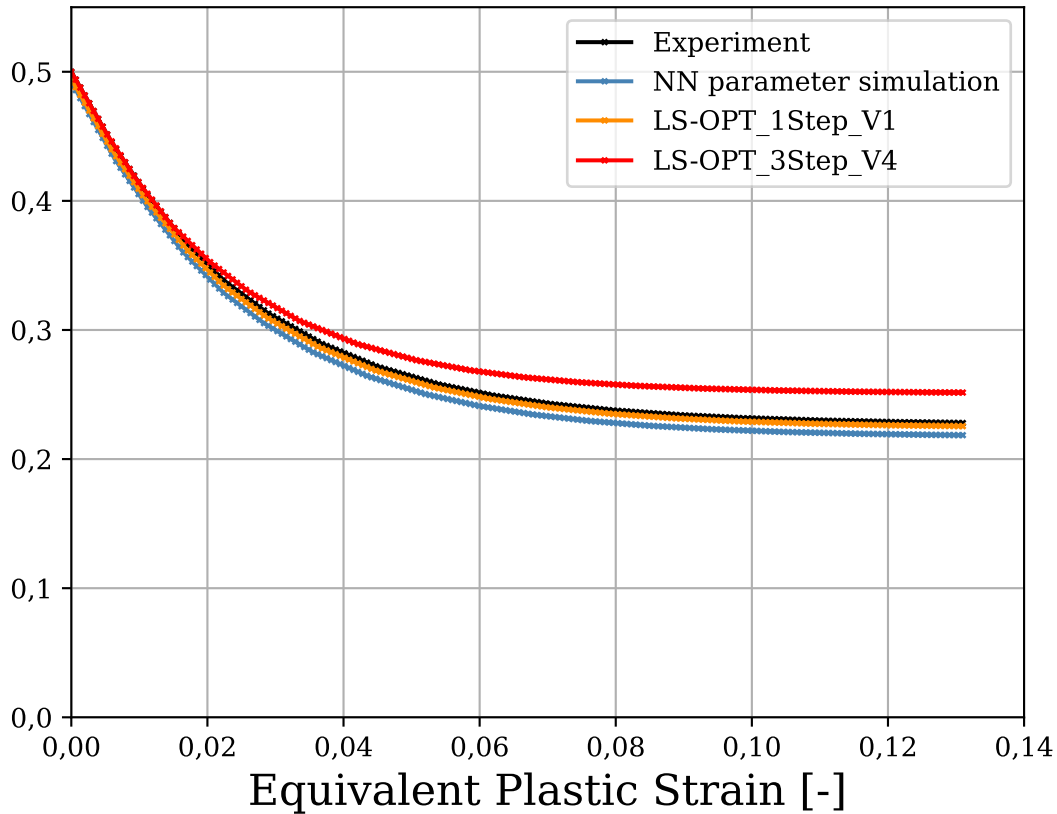

Supplement: Supplementary file 1 [file materials-15-00643-s001.zip › Supplementary_Material/SOC_NN_Pred_LSOPT_Complete/NN_Run_1/PE_Comparison_Punch_Test.pdf]

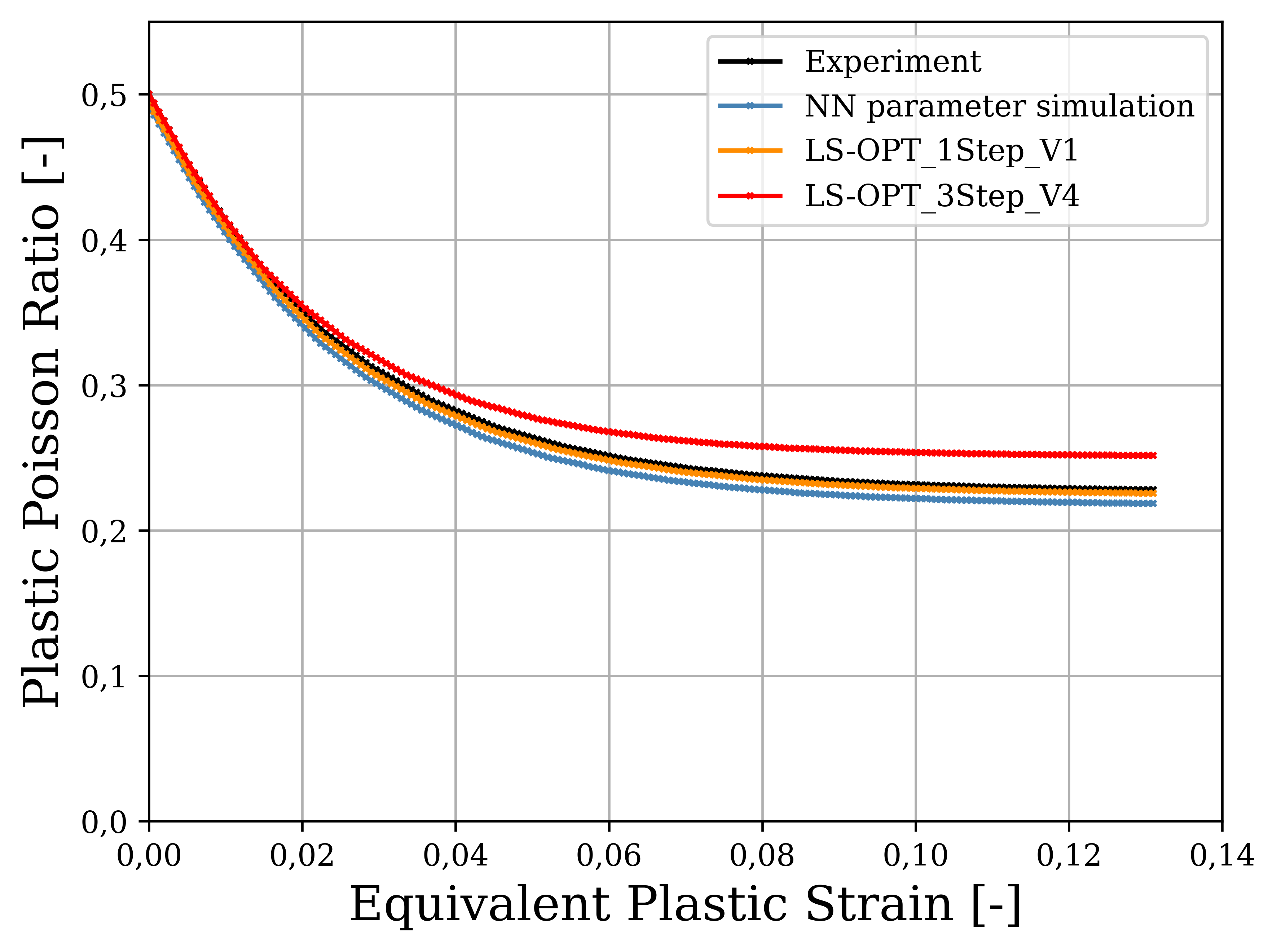

Supplement: Supplementary file 1 [file materials-15-00643-s001.zip › Supplementary_Material/SOC_NN_Pred_LSOPT_Complete/NN_Run_1/PE_Comparison_Punch_Test.png]

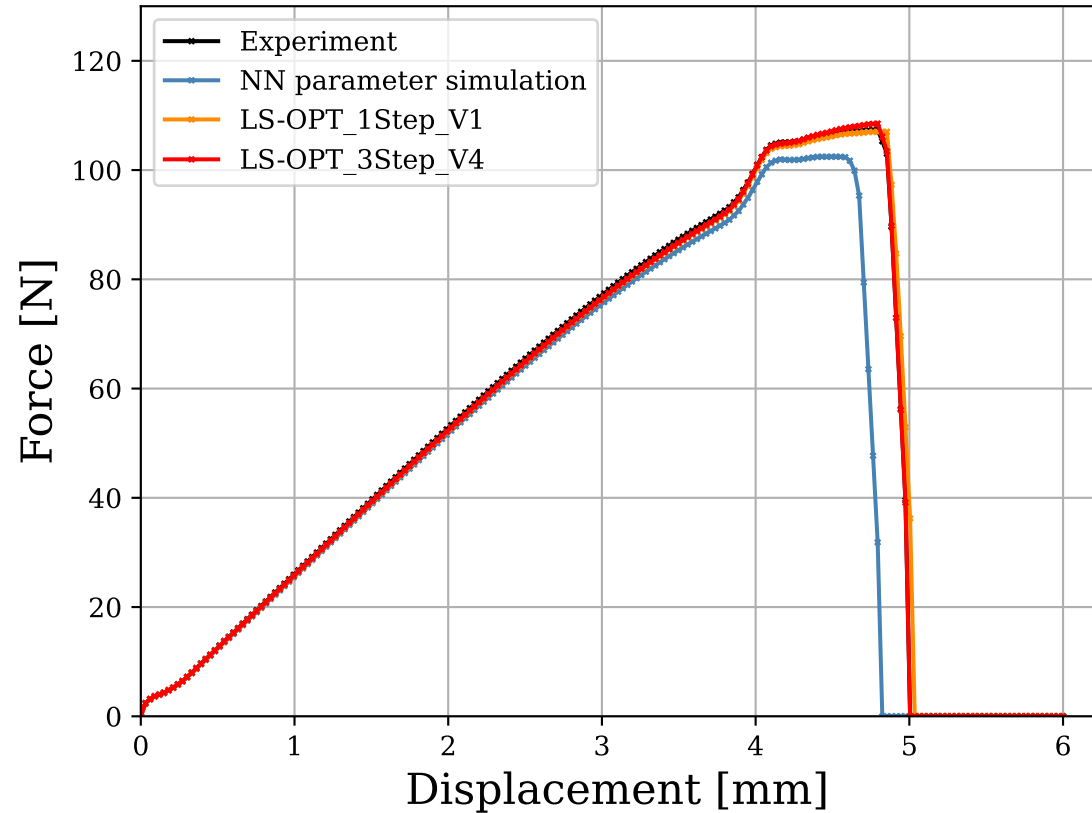

Supplement: Supplementary file 1 [file materials-15-00643-s001.zip › Supplementary_Material/SOC_NN_Pred_LSOPT_Complete/NN_Run_2/FD_Comparison_Bending_Test.pdf]

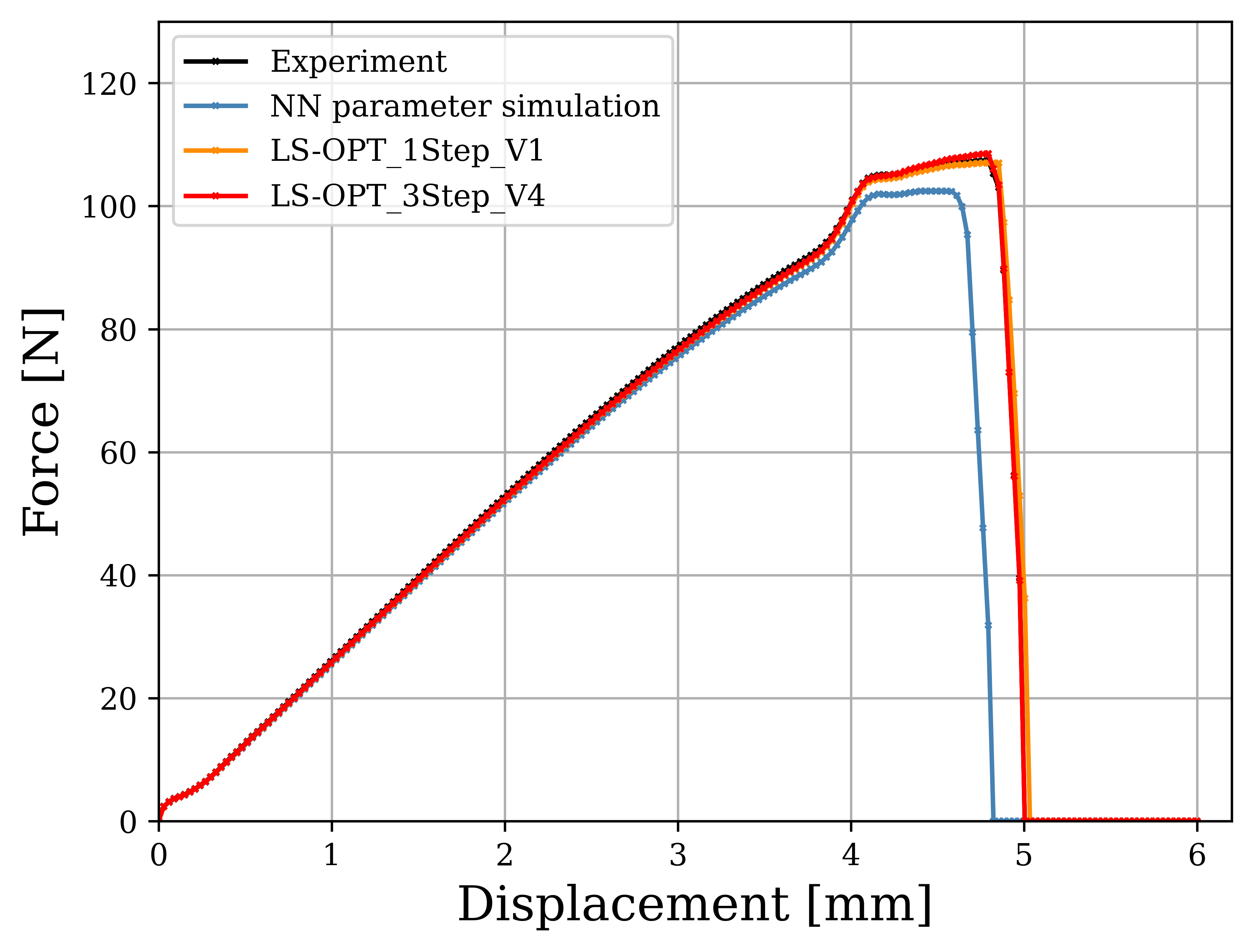

Supplement: Supplementary file 1 [file materials-15-00643-s001.zip › Supplementary_Material/SOC_NN_Pred_LSOPT_Complete/NN_Run_2/FD_Comparison_Bending_Test.png]

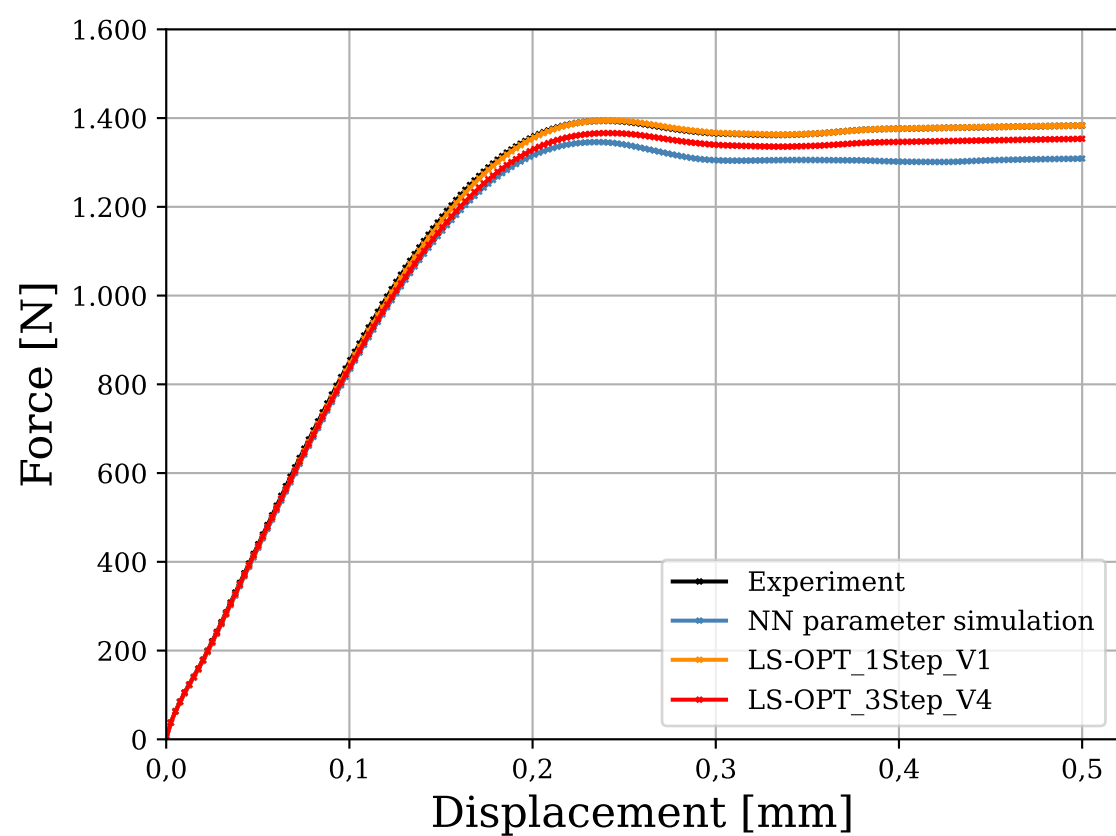

Supplement: Supplementary file 1 [file materials-15-00643-s001.zip › Supplementary_Material/SOC_NN_Pred_LSOPT_Complete/NN_Run_2/FD_Comparison_Compression_Test.pdf]

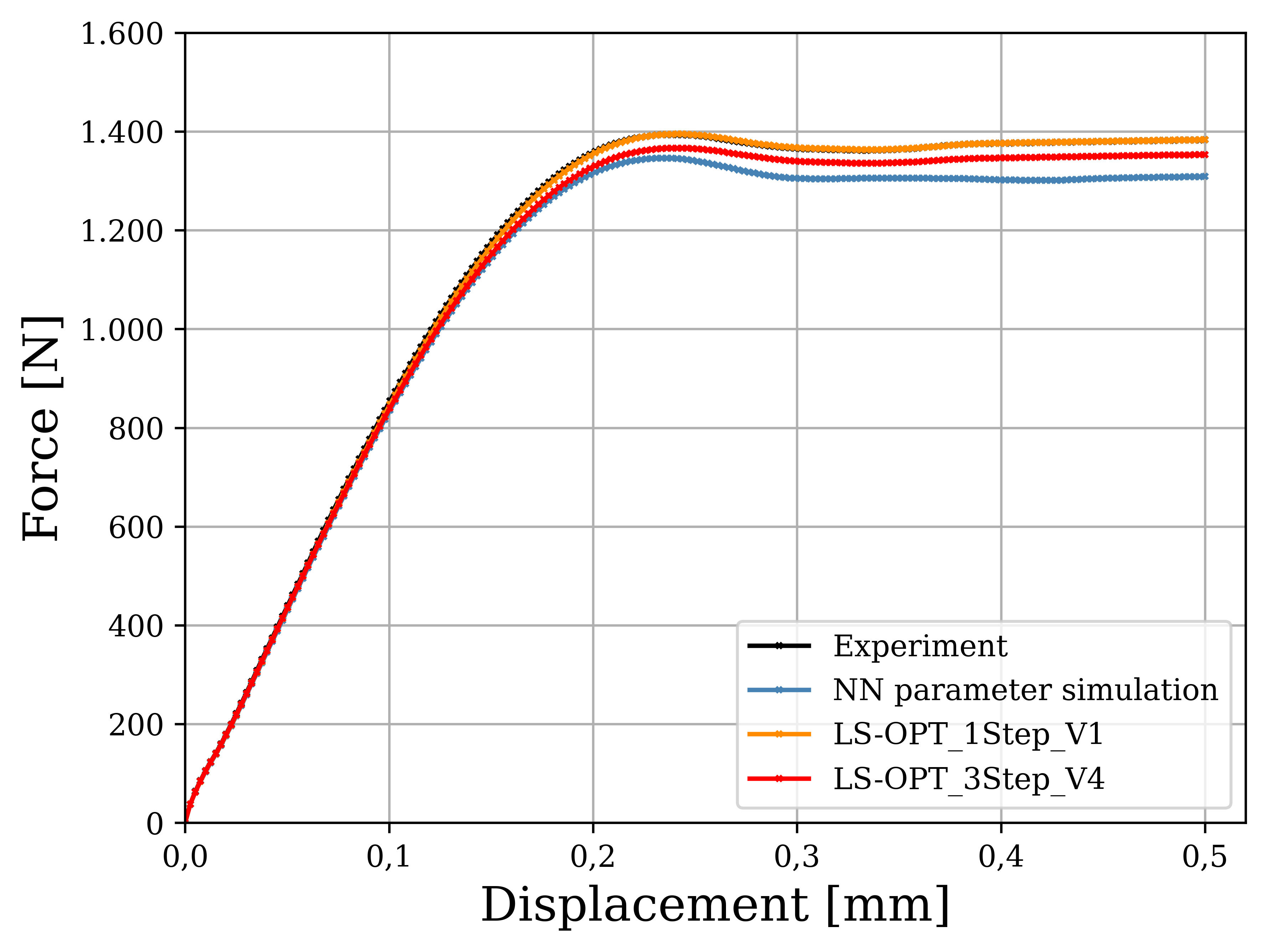

Supplement: Supplementary file 1 [file materials-15-00643-s001.zip › Supplementary_Material/SOC_NN_Pred_LSOPT_Complete/NN_Run_2/FD_Comparison_Compression_Test.png]

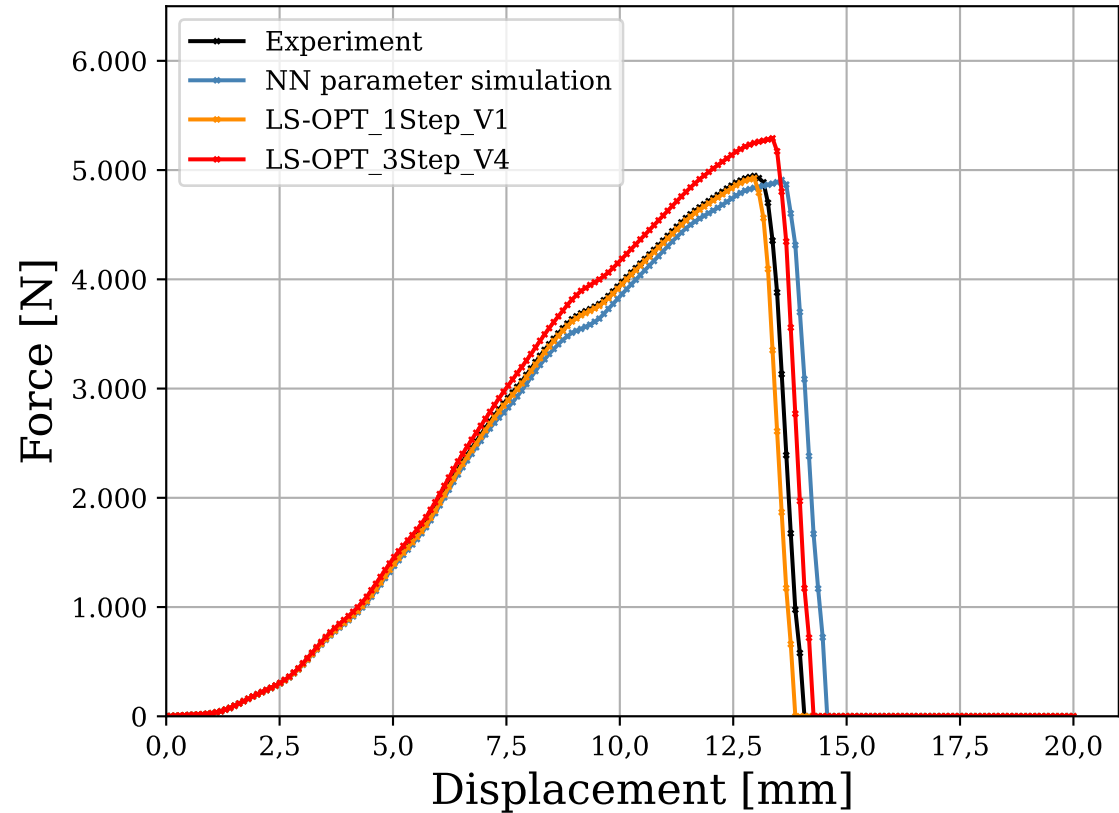

Supplement: Supplementary file 1 [file materials-15-00643-s001.zip › Supplementary_Material/SOC_NN_Pred_LSOPT_Complete/NN_Run_2/FD_Comparison_Punch_Test.pdf]

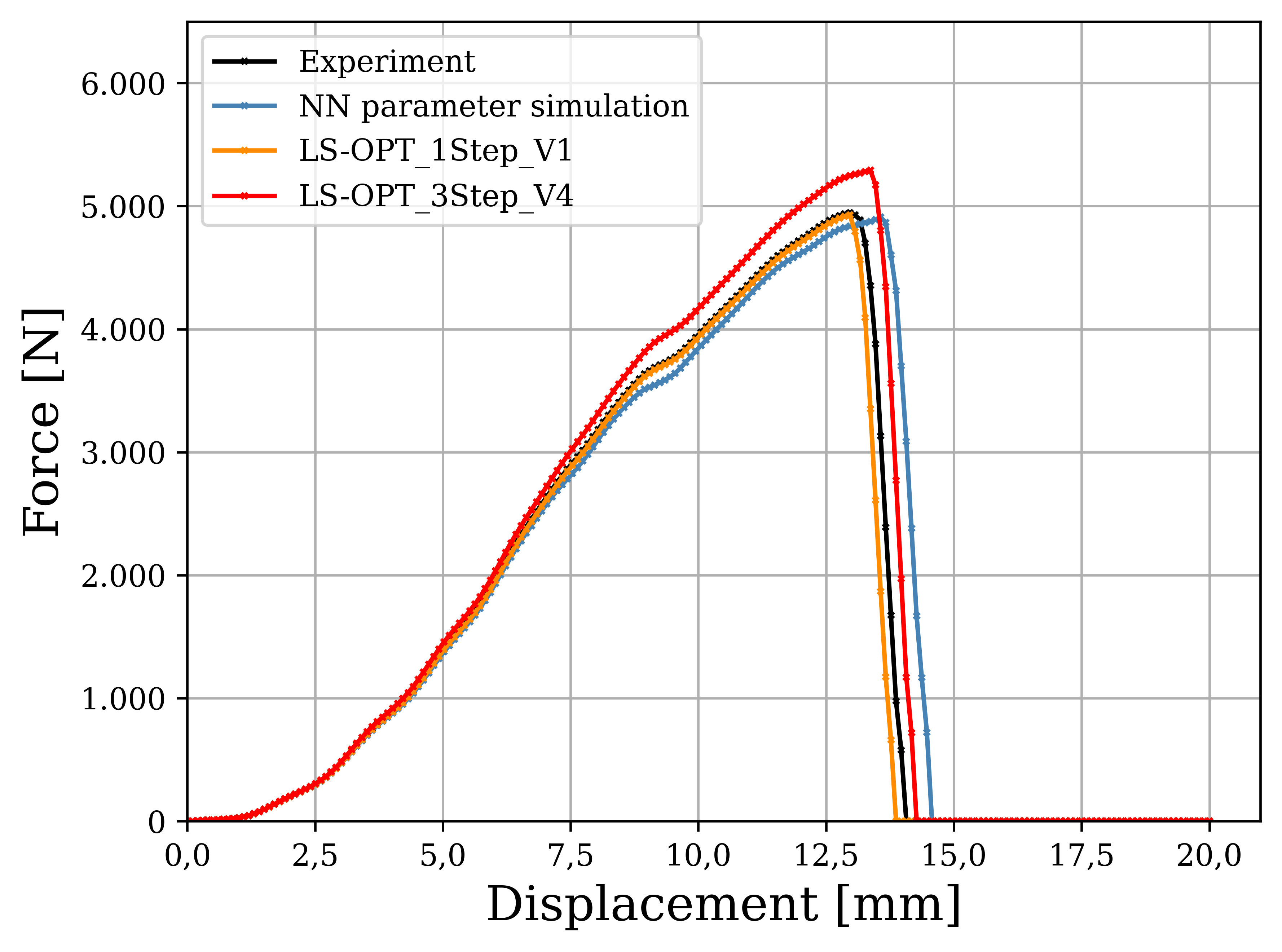

Supplement: Supplementary file 1 [file materials-15-00643-s001.zip › Supplementary_Material/SOC_NN_Pred_LSOPT_Complete/NN_Run_2/FD_Comparison_Punch_Test.png]

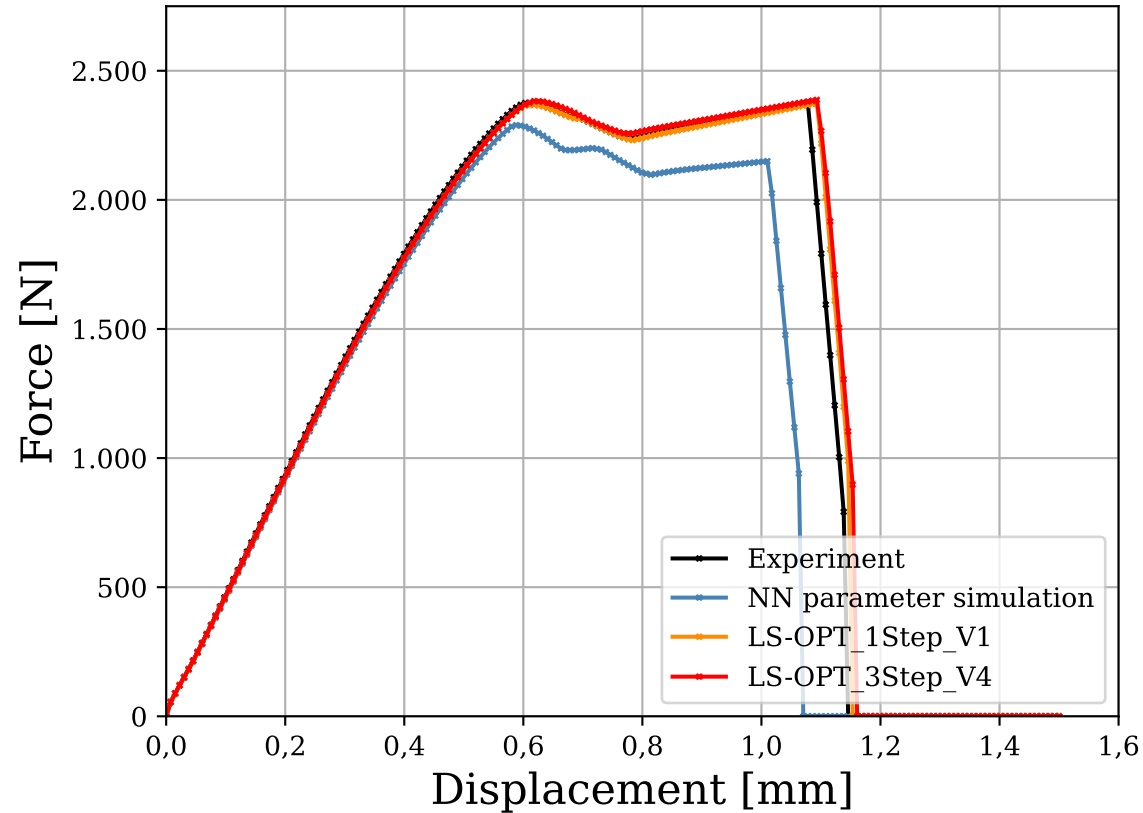

Supplement: Supplementary file 1 [file materials-15-00643-s001.zip › Supplementary_Material/SOC_NN_Pred_LSOPT_Complete/NN_Run_2/FD_Comparison_Shear_ASTM_Test.pdf]

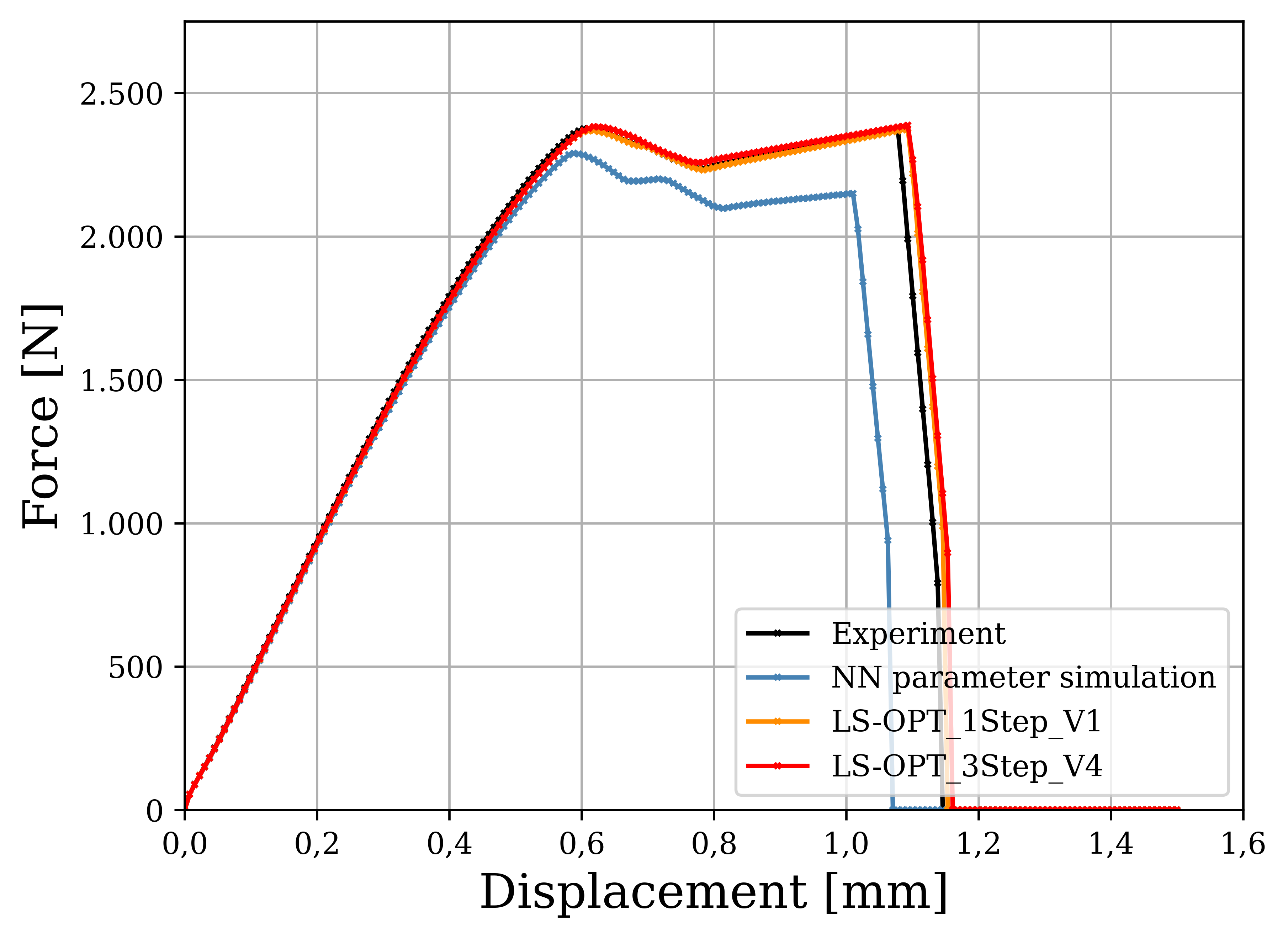

Supplement: Supplementary file 1 [file materials-15-00643-s001.zip › Supplementary_Material/SOC_NN_Pred_LSOPT_Complete/NN_Run_2/FD_Comparison_Shear_ASTM_Test.png]

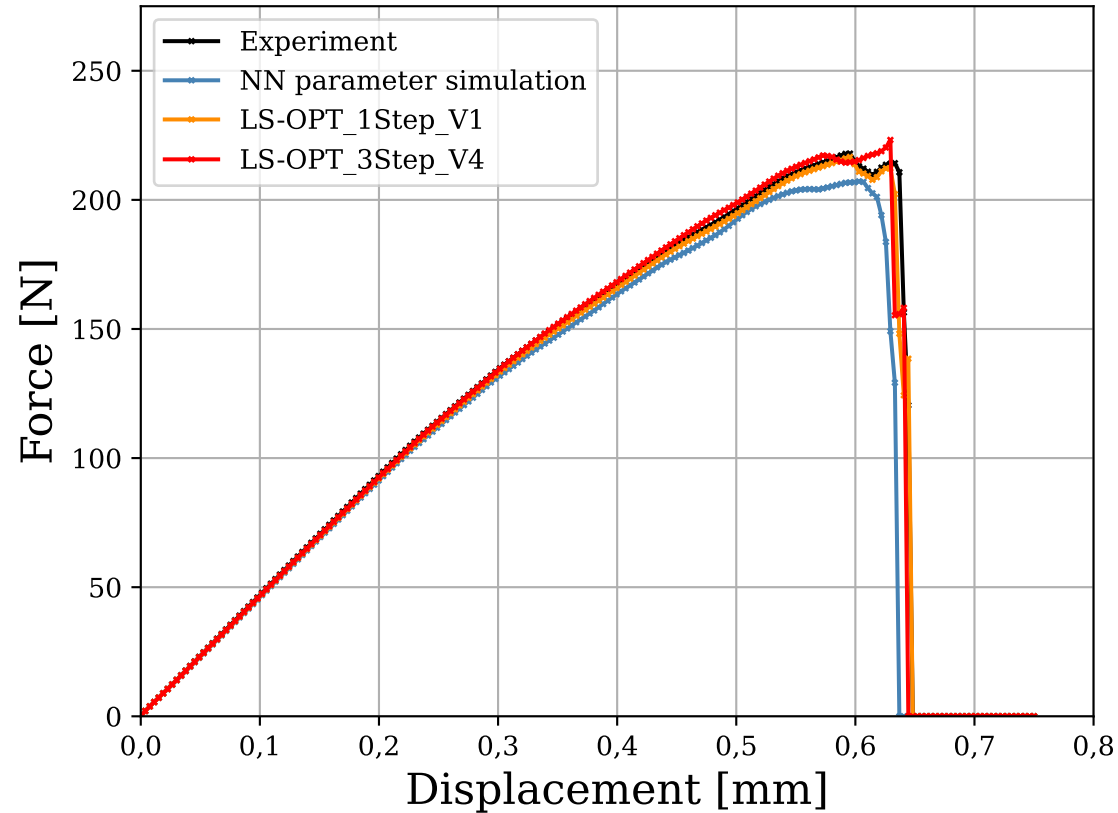

Supplement: Supplementary file 1 [file materials-15-00643-s001.zip › Supplementary_Material/SOC_NN_Pred_LSOPT_Complete/NN_Run_2/FD_Comparison_Shear_Dynamore_Test.pdf]

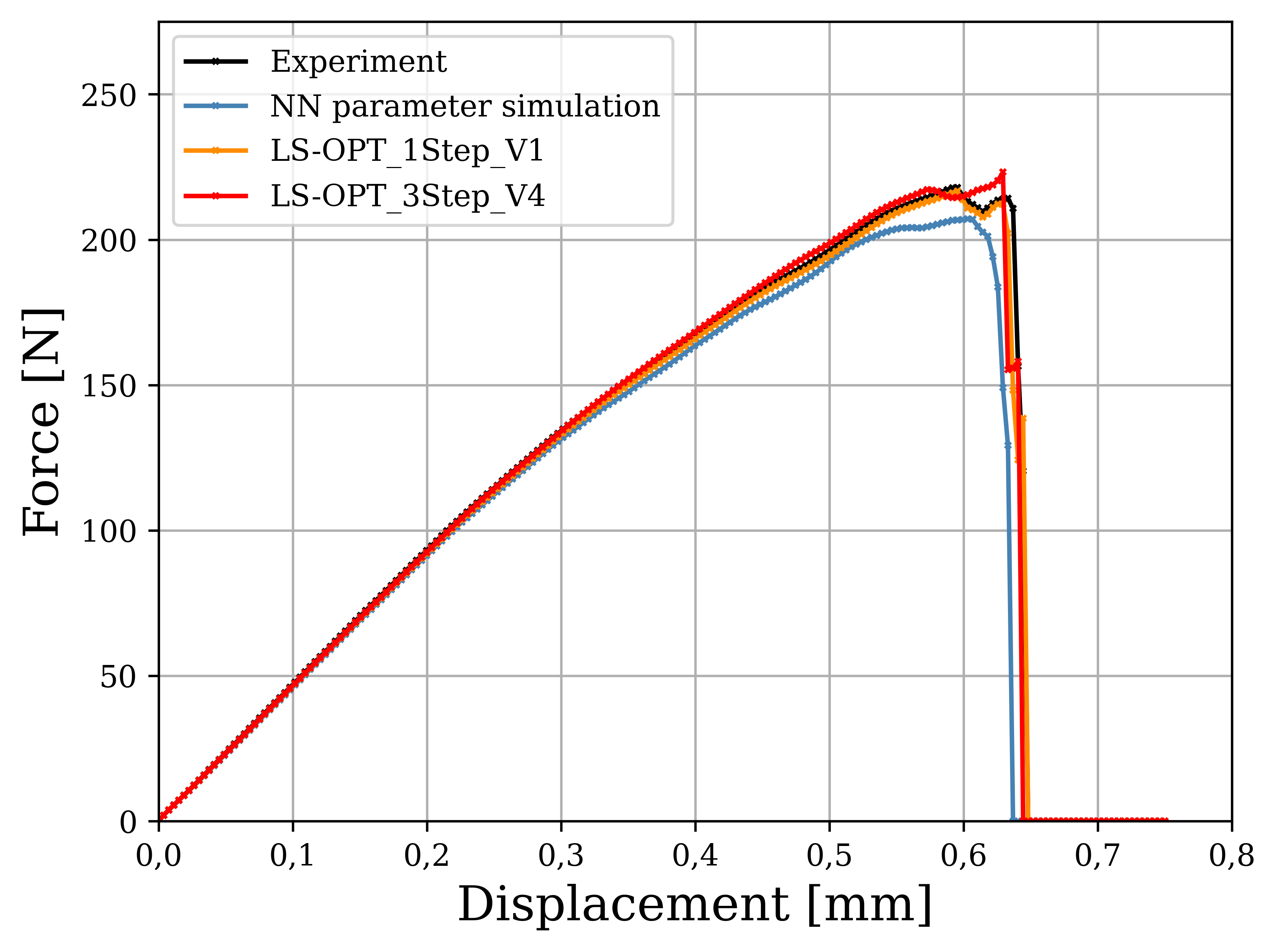

Supplement: Supplementary file 1 [file materials-15-00643-s001.zip › Supplementary_Material/SOC_NN_Pred_LSOPT_Complete/NN_Run_2/FD_Comparison_Shear_Dynamore_Test.png]

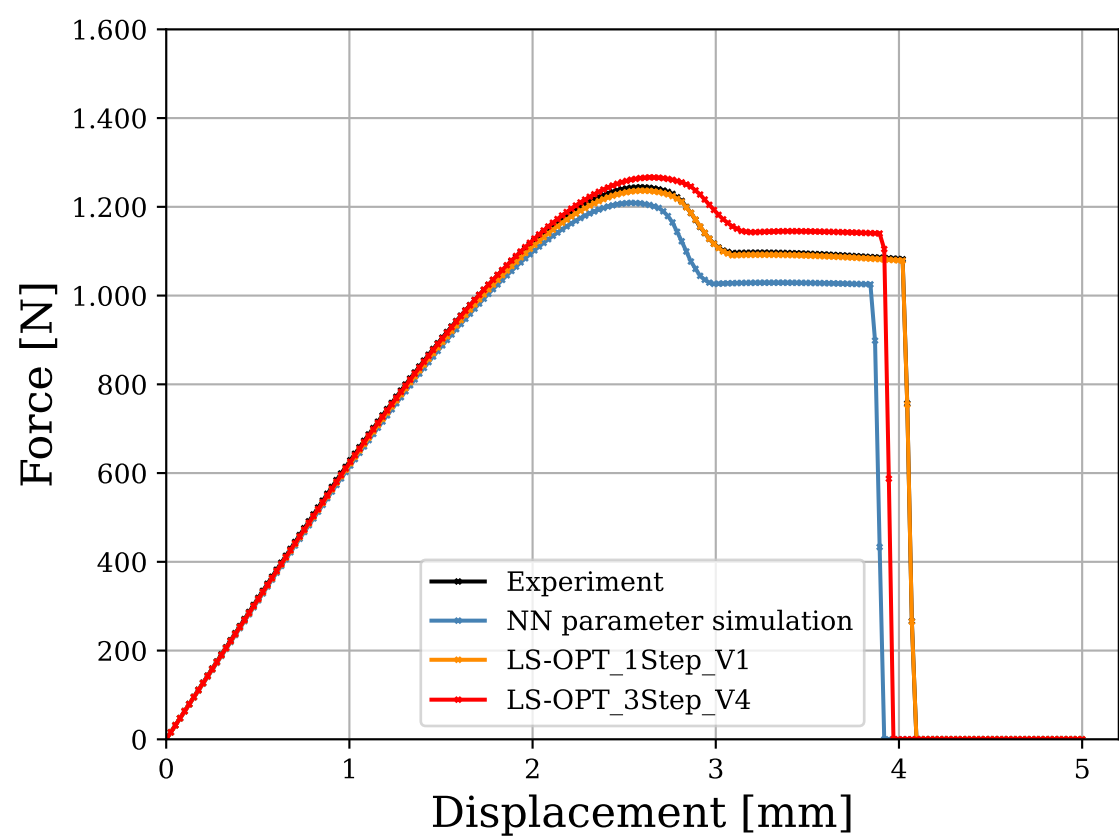

Supplement: Supplementary file 1 [file materials-15-00643-s001.zip › Supplementary_Material/SOC_NN_Pred_LSOPT_Complete/NN_Run_2/FD_Comparison_Tensile_Test.pdf]

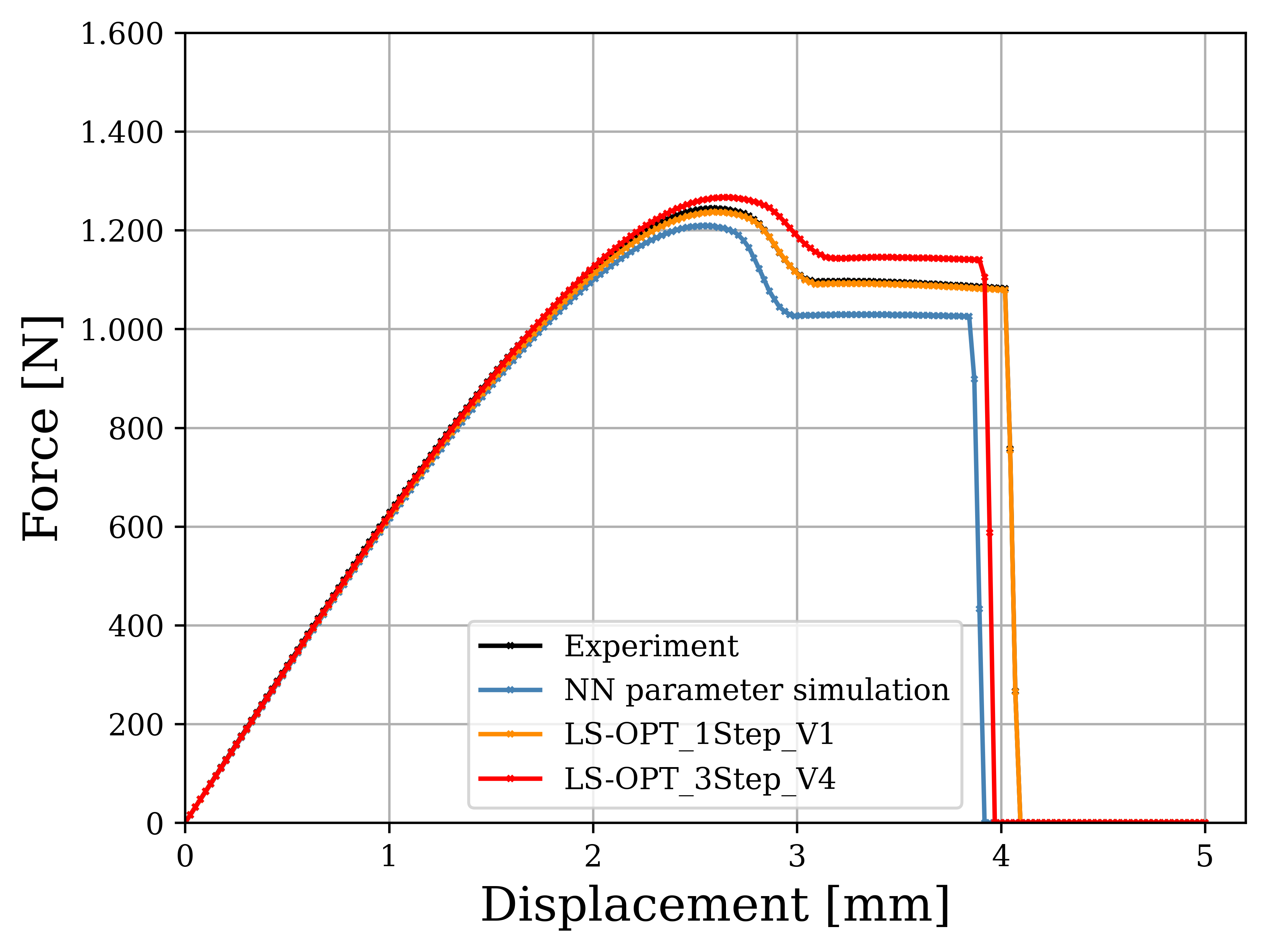

Supplement: Supplementary file 1 [file materials-15-00643-s001.zip › Supplementary_Material/SOC_NN_Pred_LSOPT_Complete/NN_Run_2/FD_Comparison_Tensile_Test.png]

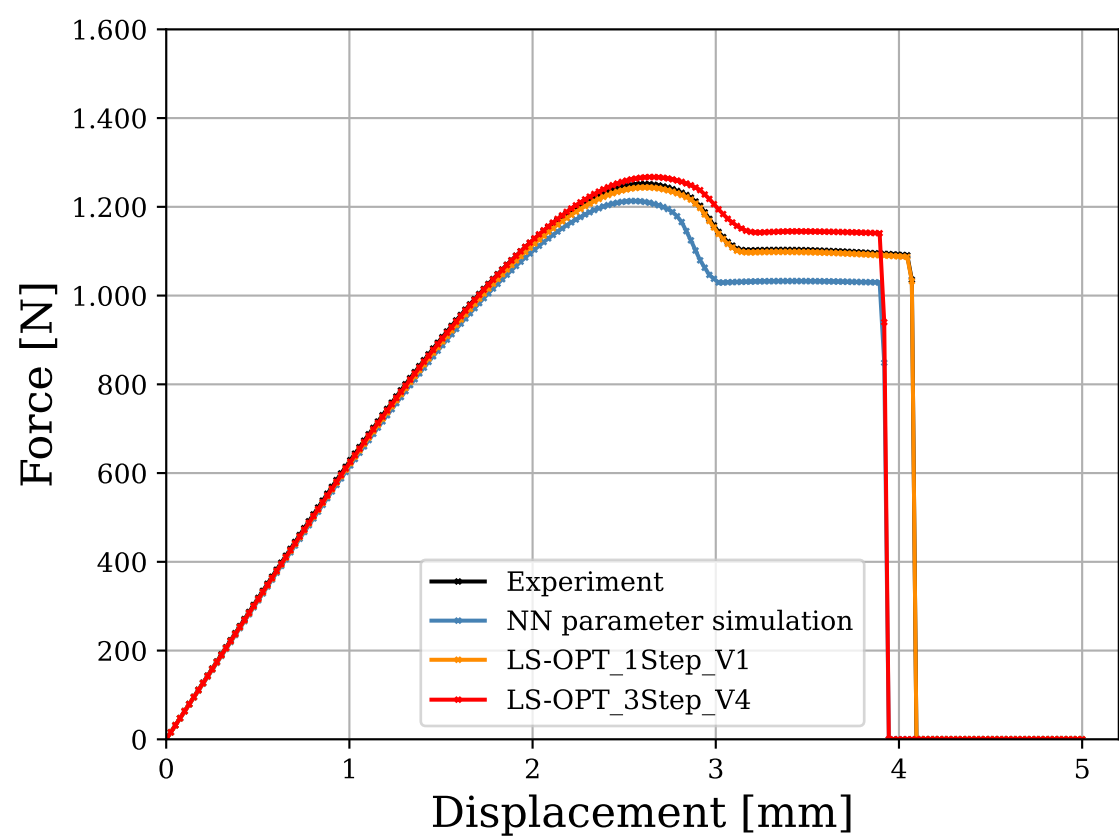

Supplement: Supplementary file 1 [file materials-15-00643-s001.zip › Supplementary_Material/SOC_NN_Pred_LSOPT_Complete/NN_Run_2/FD_Comparison_Tensile_Test_V1.pdf]

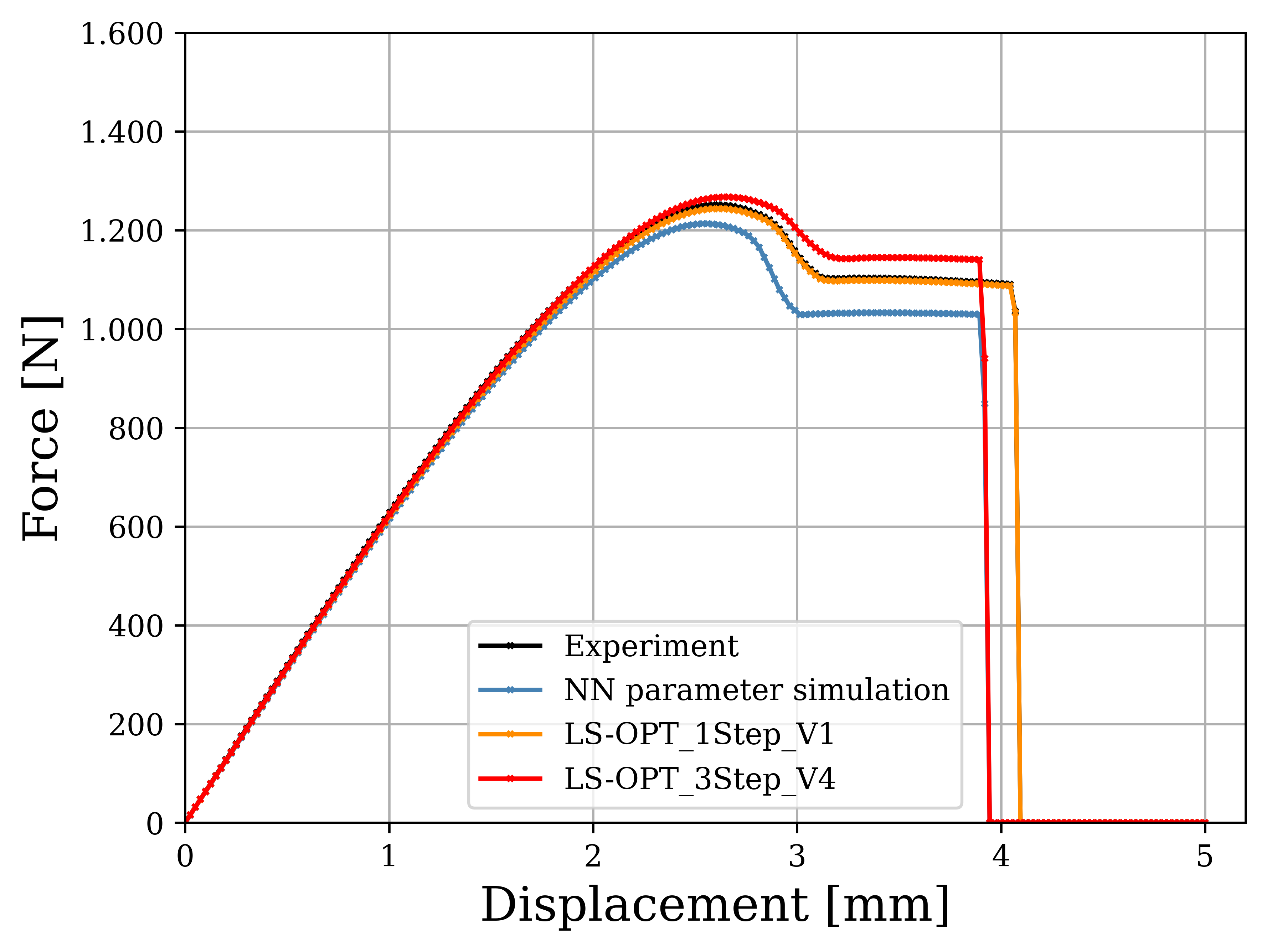

Supplement: Supplementary file 1 [file materials-15-00643-s001.zip › Supplementary_Material/SOC_NN_Pred_LSOPT_Complete/NN_Run_2/FD_Comparison_Tensile_Test_V1.png]

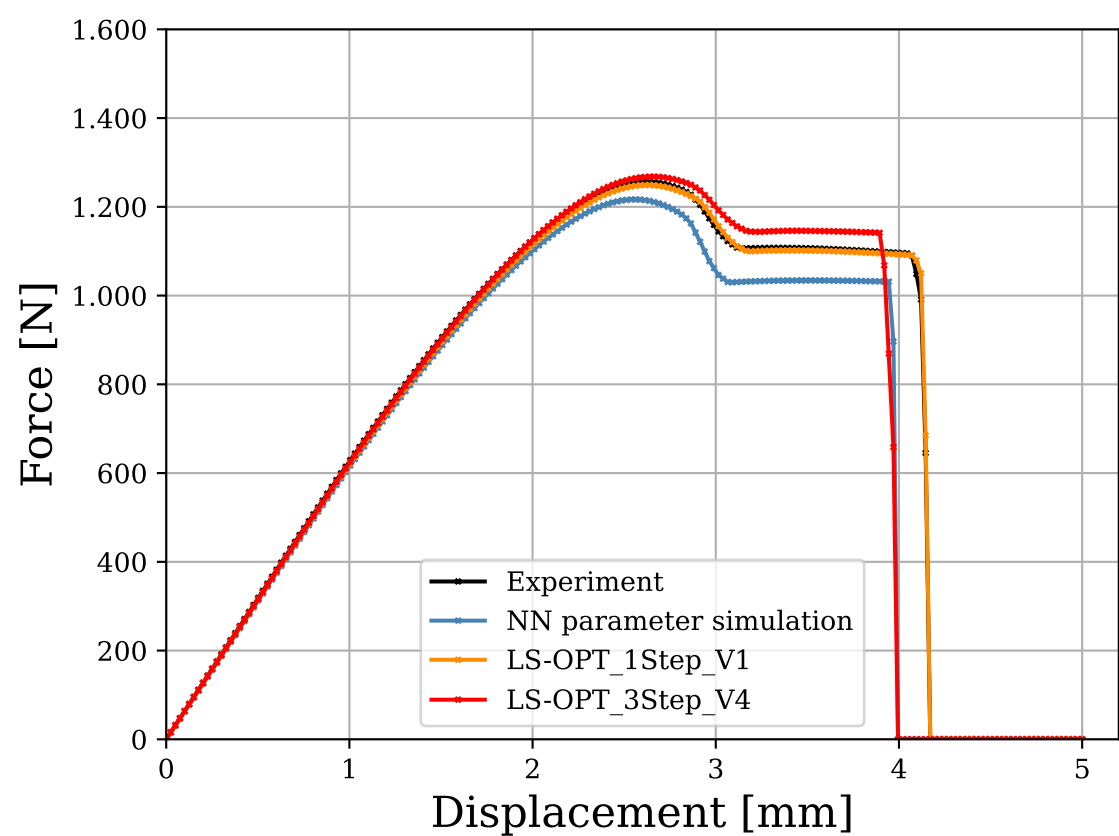

Supplement: Supplementary file 1 [file materials-15-00643-s001.zip › Supplementary_Material/SOC_NN_Pred_LSOPT_Complete/NN_Run_2/FD_Comparison_Tensile_Test_V2.pdf]

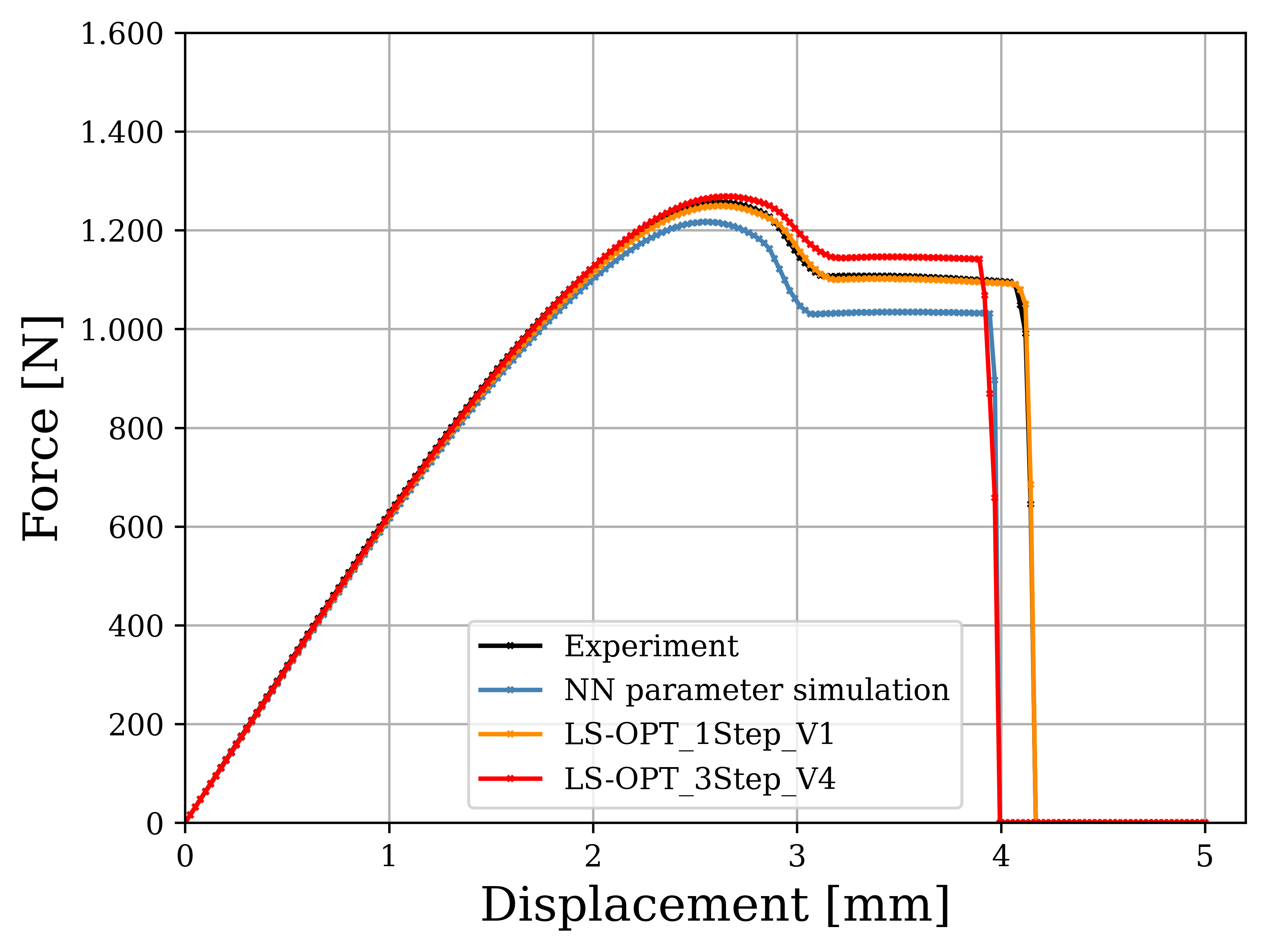

Supplement: Supplementary file 1 [file materials-15-00643-s001.zip › Supplementary_Material/SOC_NN_Pred_LSOPT_Complete/NN_Run_2/FD_Comparison_Tensile_Test_V2.png]

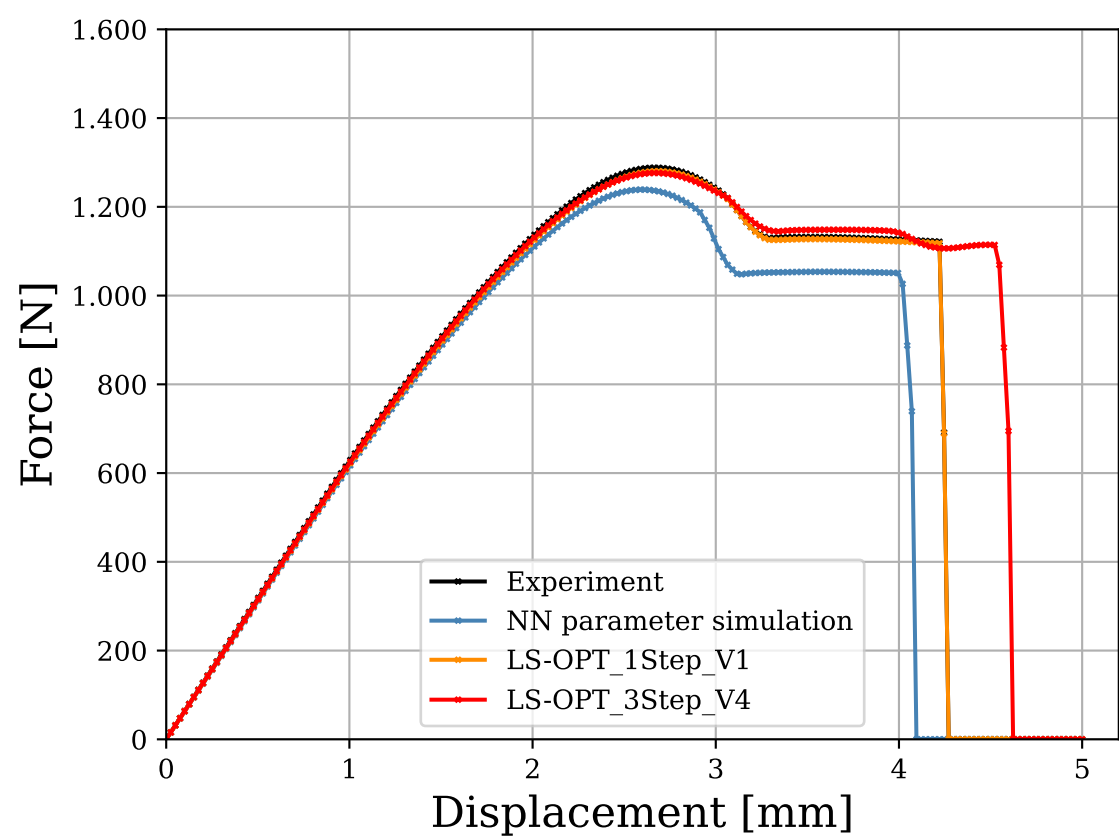

Supplement: Supplementary file 1 [file materials-15-00643-s001.zip › Supplementary_Material/SOC_NN_Pred_LSOPT_Complete/NN_Run_2/FD_Comparison_Tensile_Test_V3.pdf]

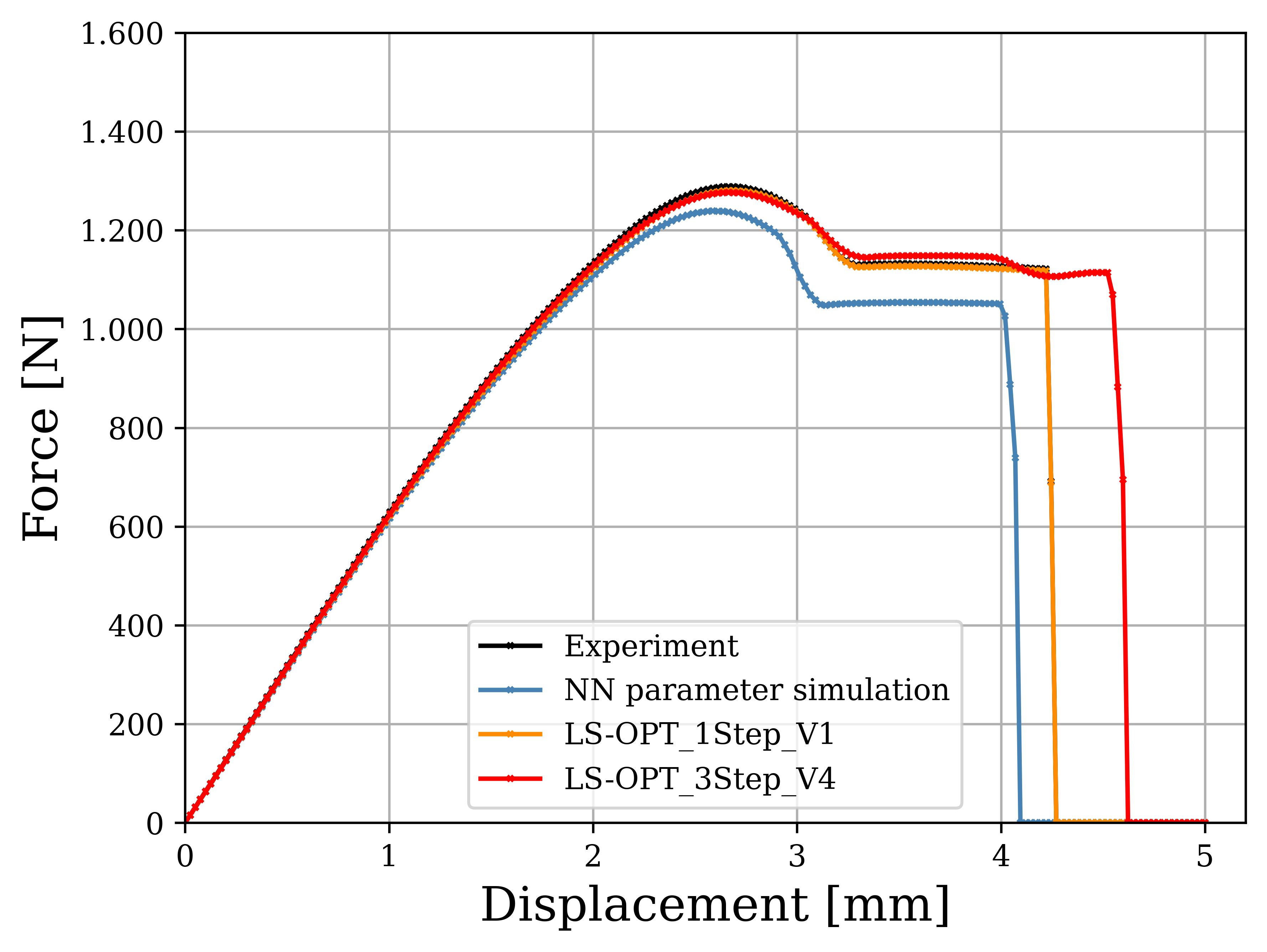

Supplement: Supplementary file 1 [file materials-15-00643-s001.zip › Supplementary_Material/SOC_NN_Pred_LSOPT_Complete/NN_Run_2/FD_Comparison_Tensile_Test_V3.png]

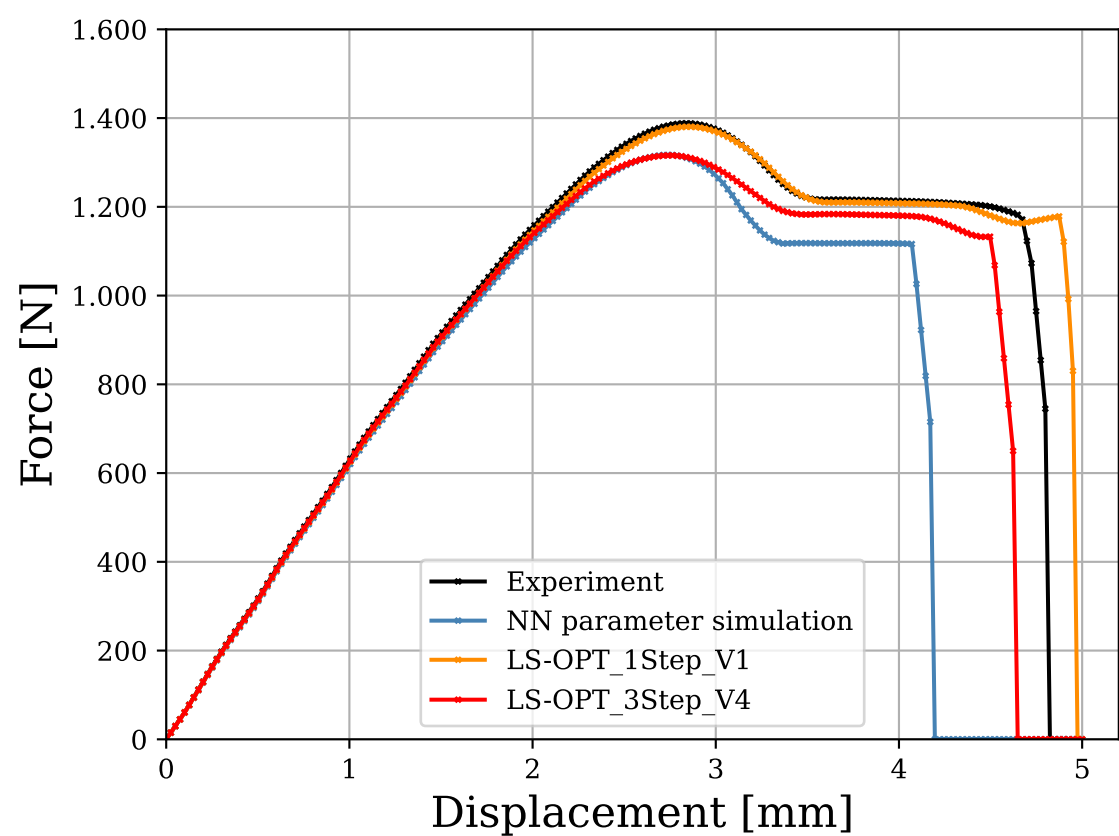

Supplement: Supplementary file 1 [file materials-15-00643-s001.zip › Supplementary_Material/SOC_NN_Pred_LSOPT_Complete/NN_Run_2/FD_Comparison_Tensile_Test_V4.pdf]

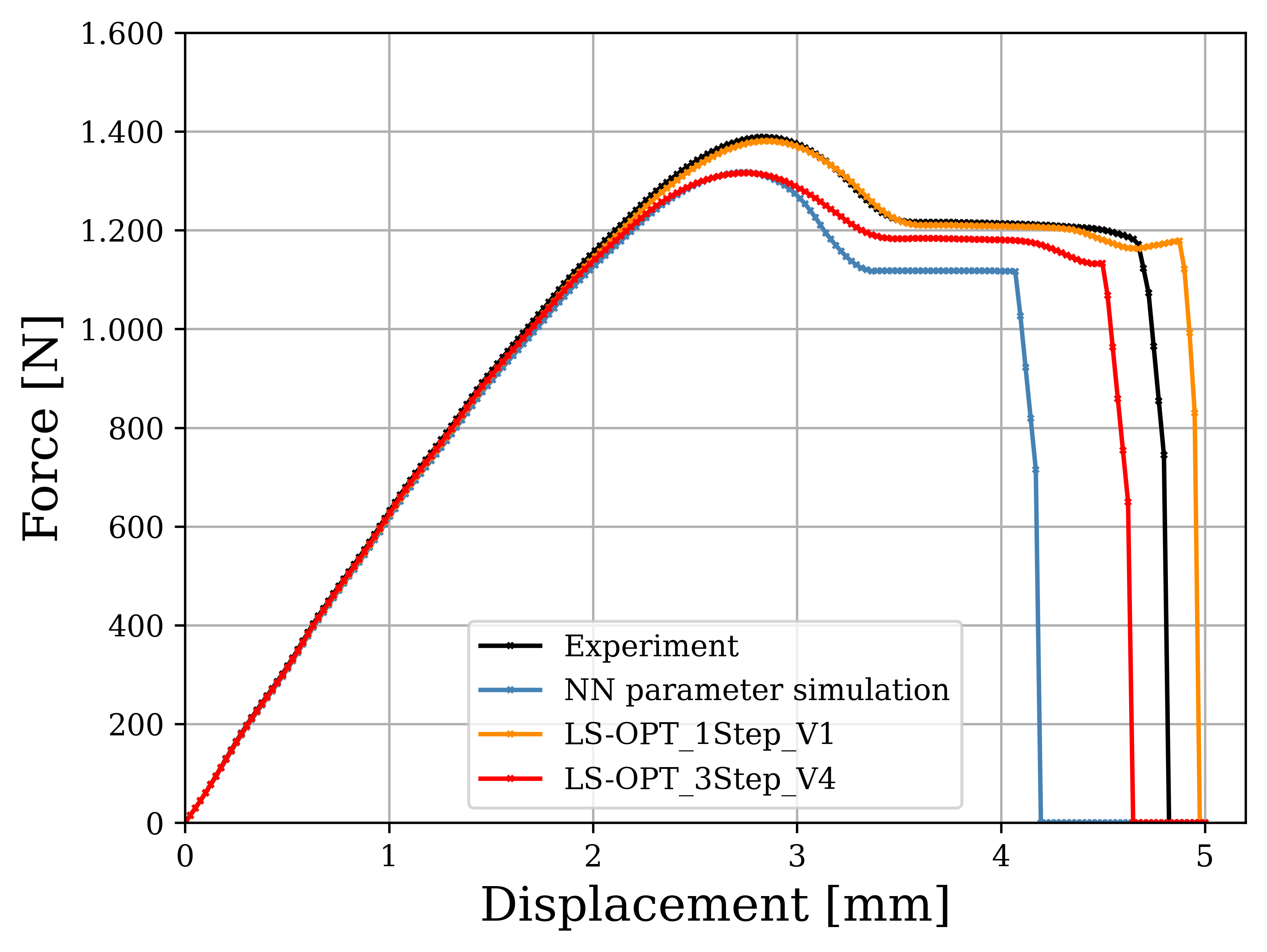

Supplement: Supplementary file 1 [file materials-15-00643-s001.zip › Supplementary_Material/SOC_NN_Pred_LSOPT_Complete/NN_Run_2/FD_Comparison_Tensile_Test_V4.png]

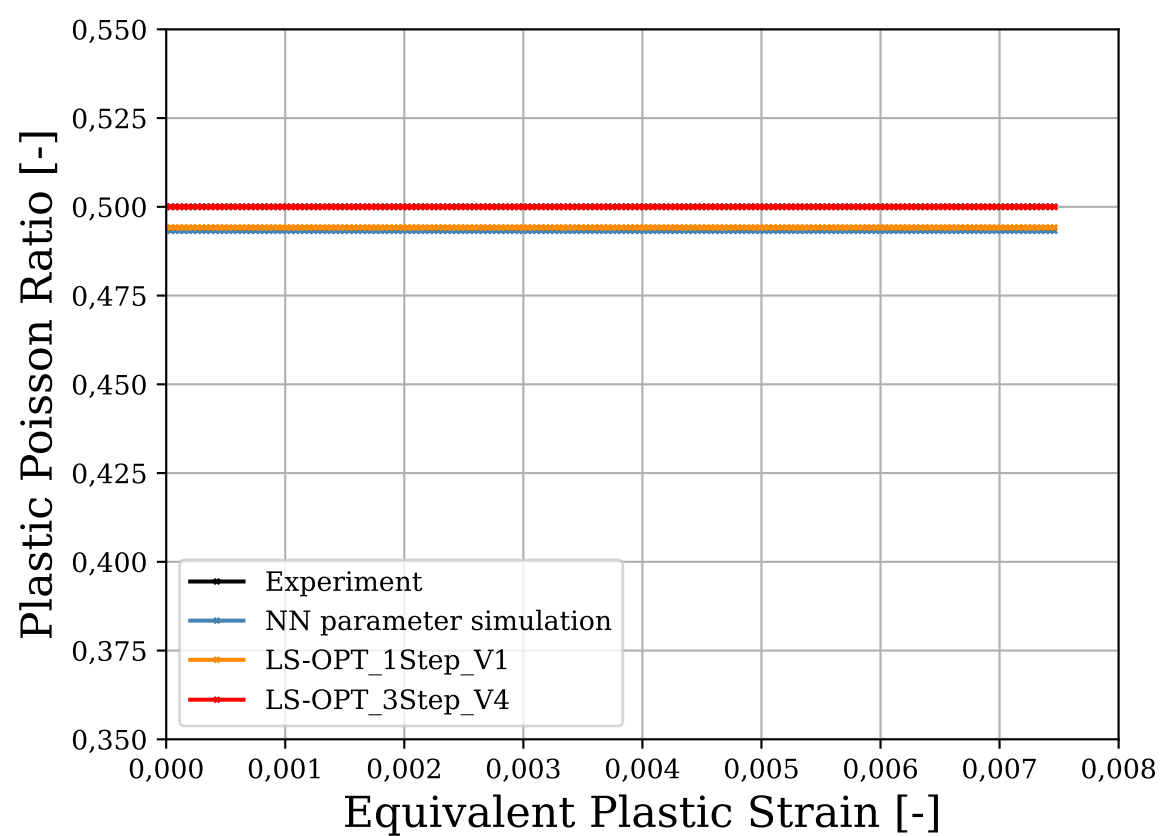

Supplement: Supplementary file 1 [file materials-15-00643-s001.zip › Supplementary_Material/SOC_NN_Pred_LSOPT_Complete/NN_Run_2/PE_Comparison_Compression_Test.pdf]

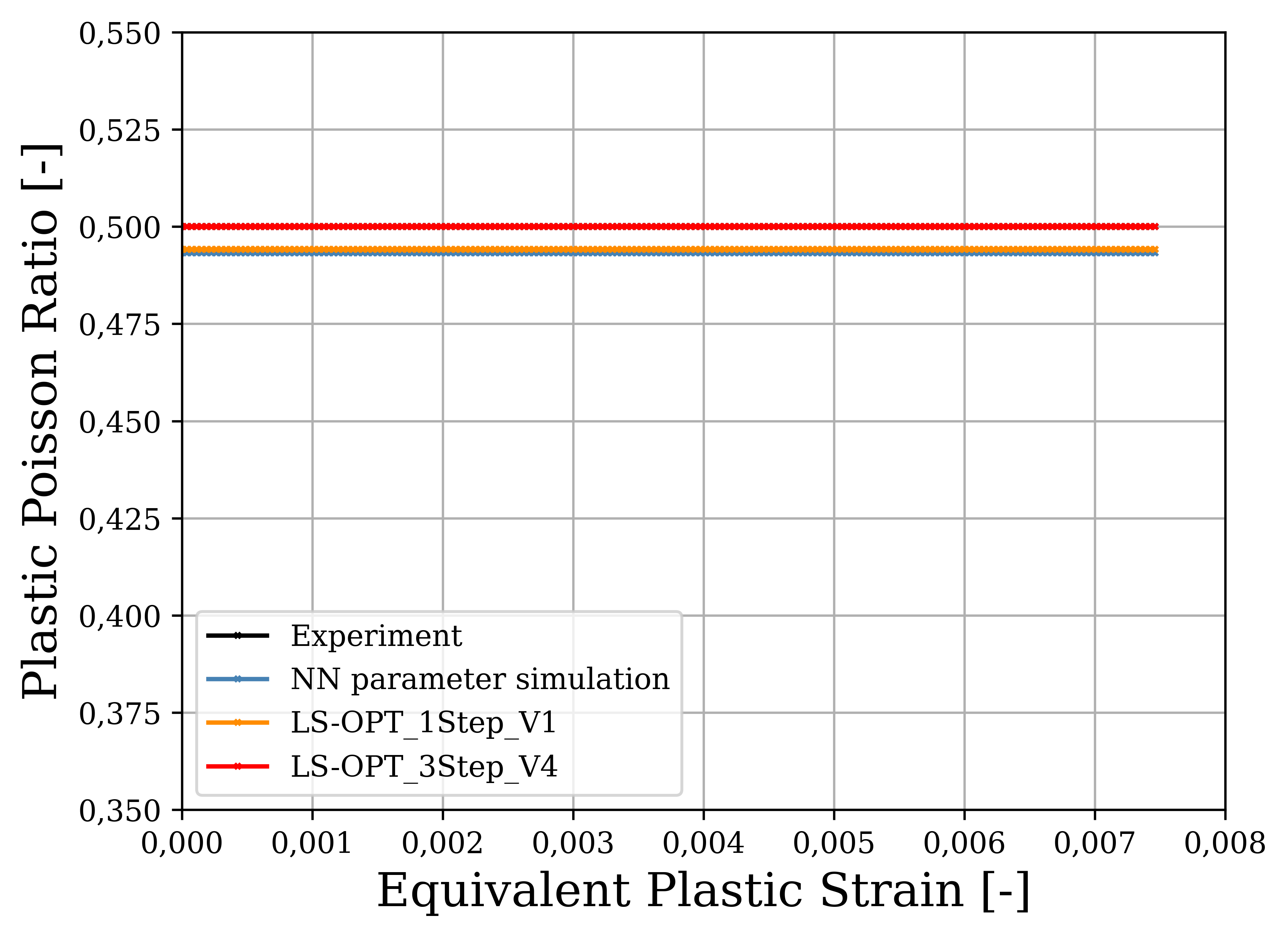

Supplement: Supplementary file 1 [file materials-15-00643-s001.zip › Supplementary_Material/SOC_NN_Pred_LSOPT_Complete/NN_Run_2/PE_Comparison_Compression_Test.png]

Plastic Poisson Ratio [-]

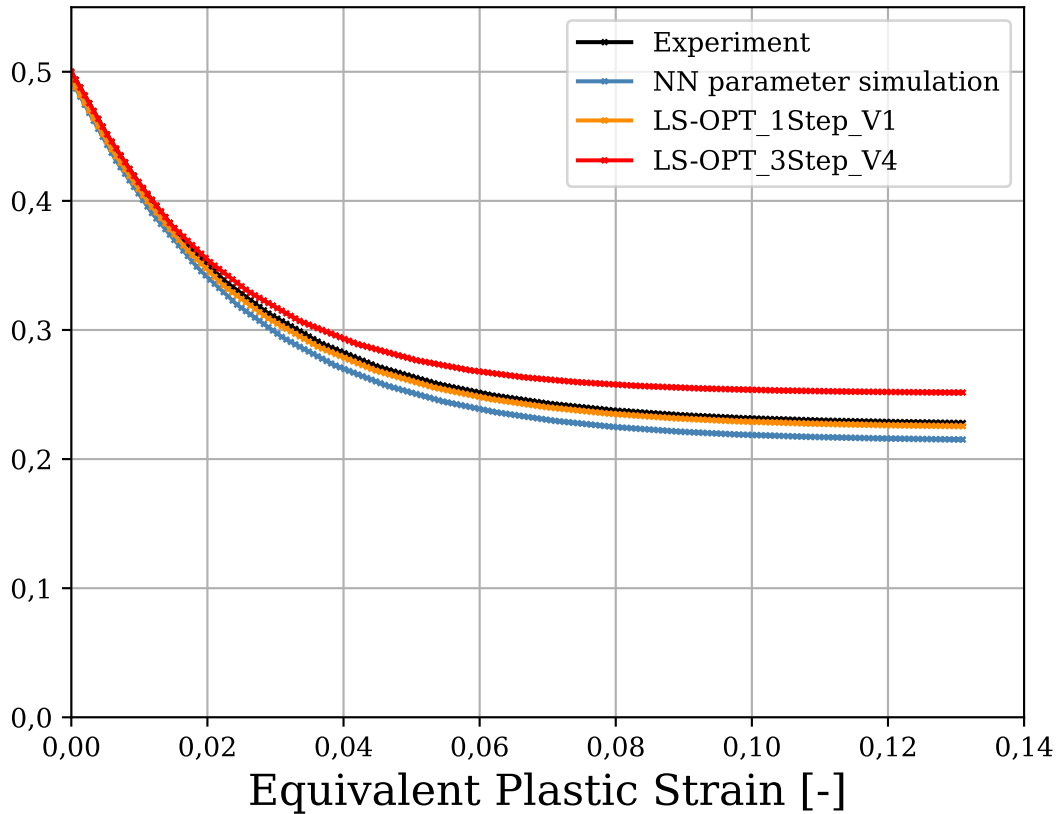

Supplement: Supplementary file 1 [file materials-15-00643-s001.zip › Supplementary_Material/SOC_NN_Pred_LSOPT_Complete/NN_Run_2/PE_Comparison_Punch_Test.pdf]

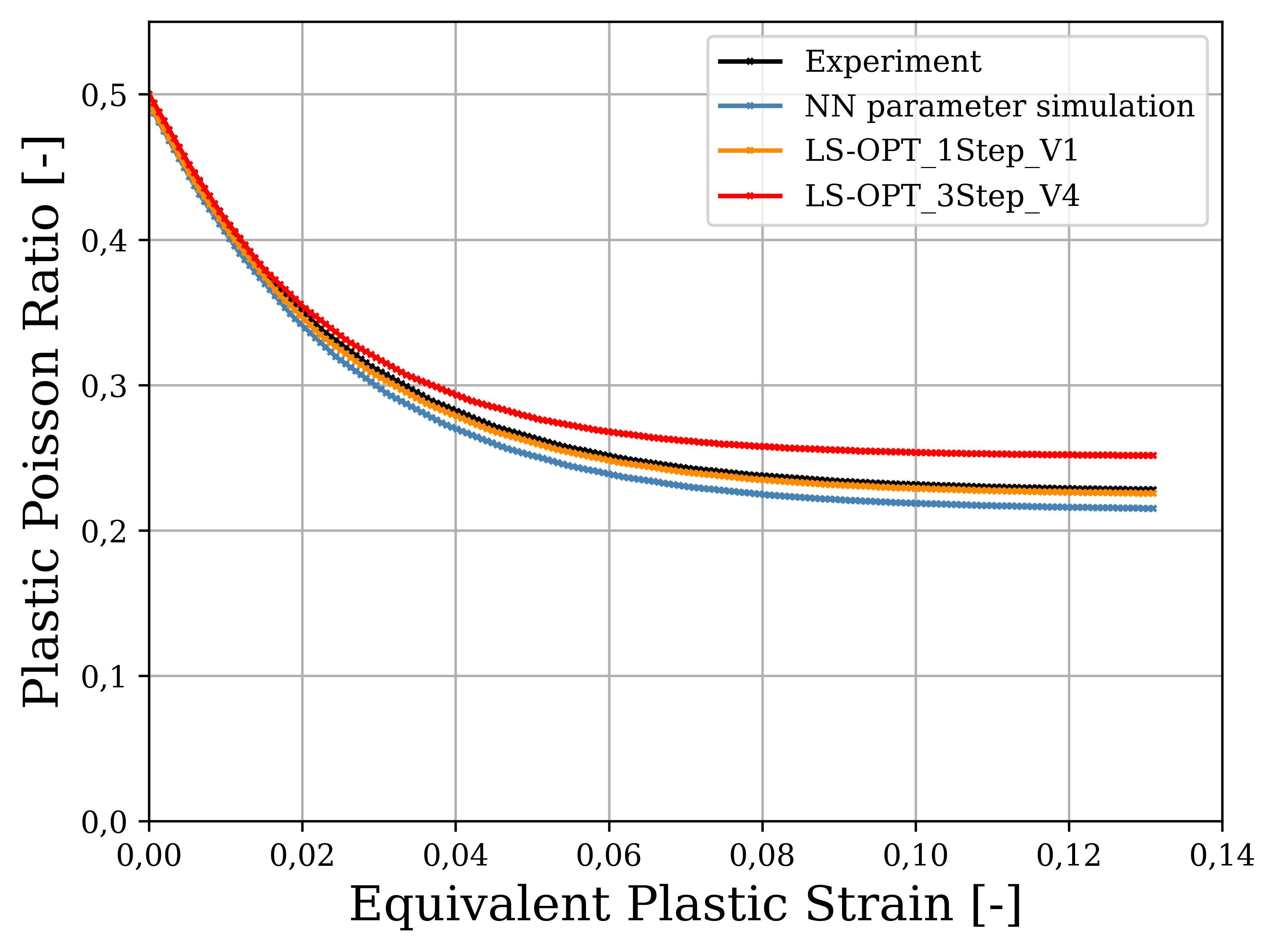

Supplement: Supplementary file 1 [file materials-15-00643-s001.zip › Supplementary_Material/SOC_NN_Pred_LSOPT_Complete/NN_Run_2/PE_Comparison_Punch_Test.png]

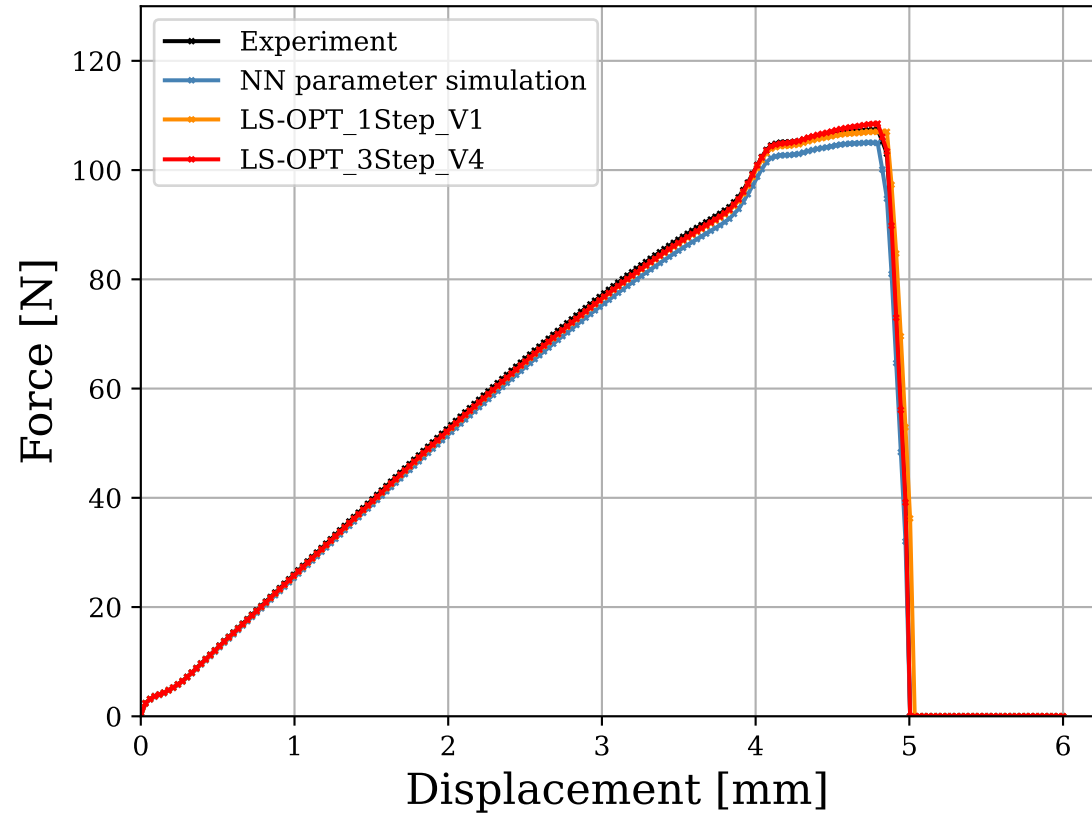

Supplement: Supplementary file 1 [file materials-15-00643-s001.zip › Supplementary_Material/SOC_NN_Pred_LSOPT_Complete/NN_Run_3/FD_Comparison_Bending_Test.pdf]

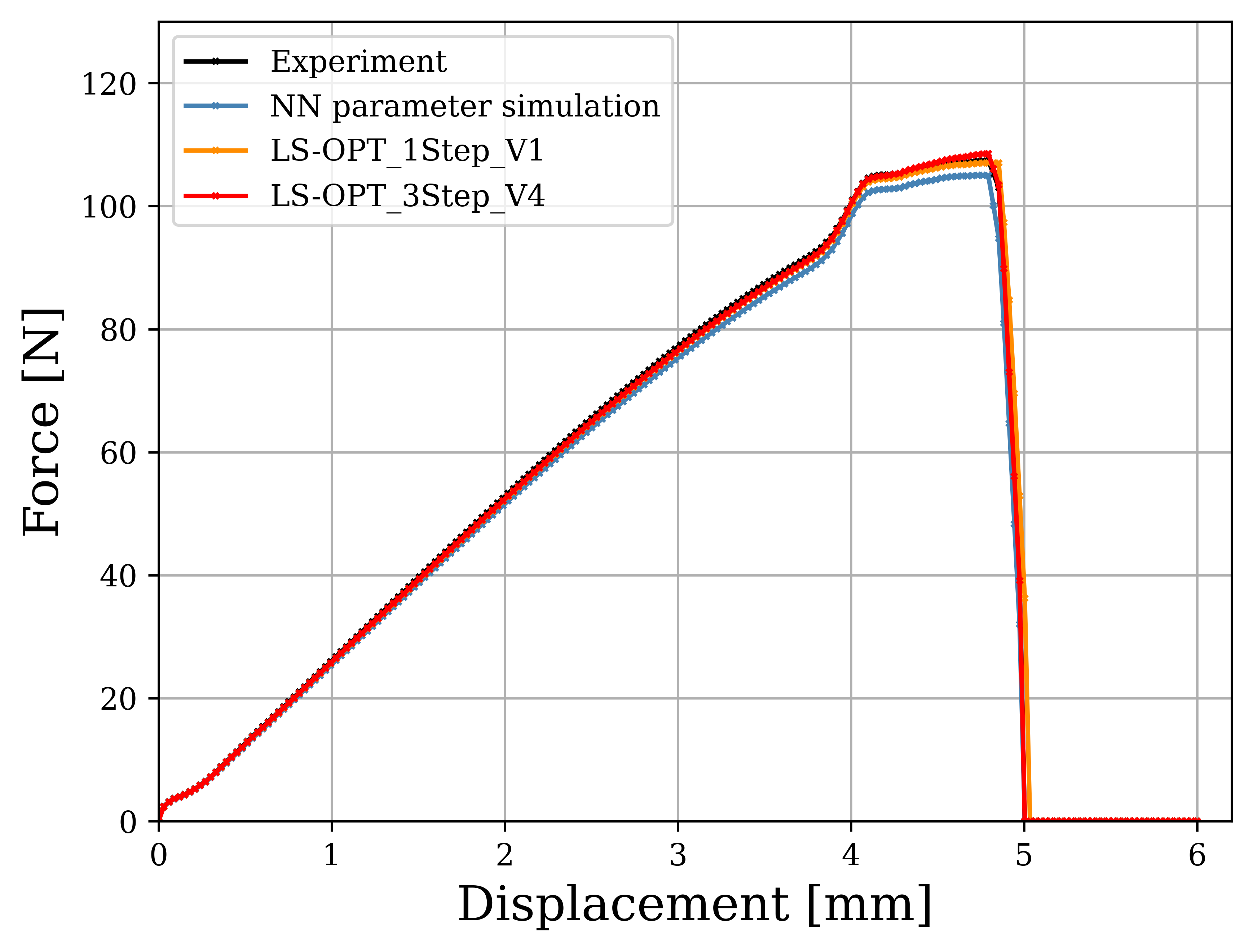

Supplement: Supplementary file 1 [file materials-15-00643-s001.zip › Supplementary_Material/SOC_NN_Pred_LSOPT_Complete/NN_Run_3/FD_Comparison_Bending_Test.png]

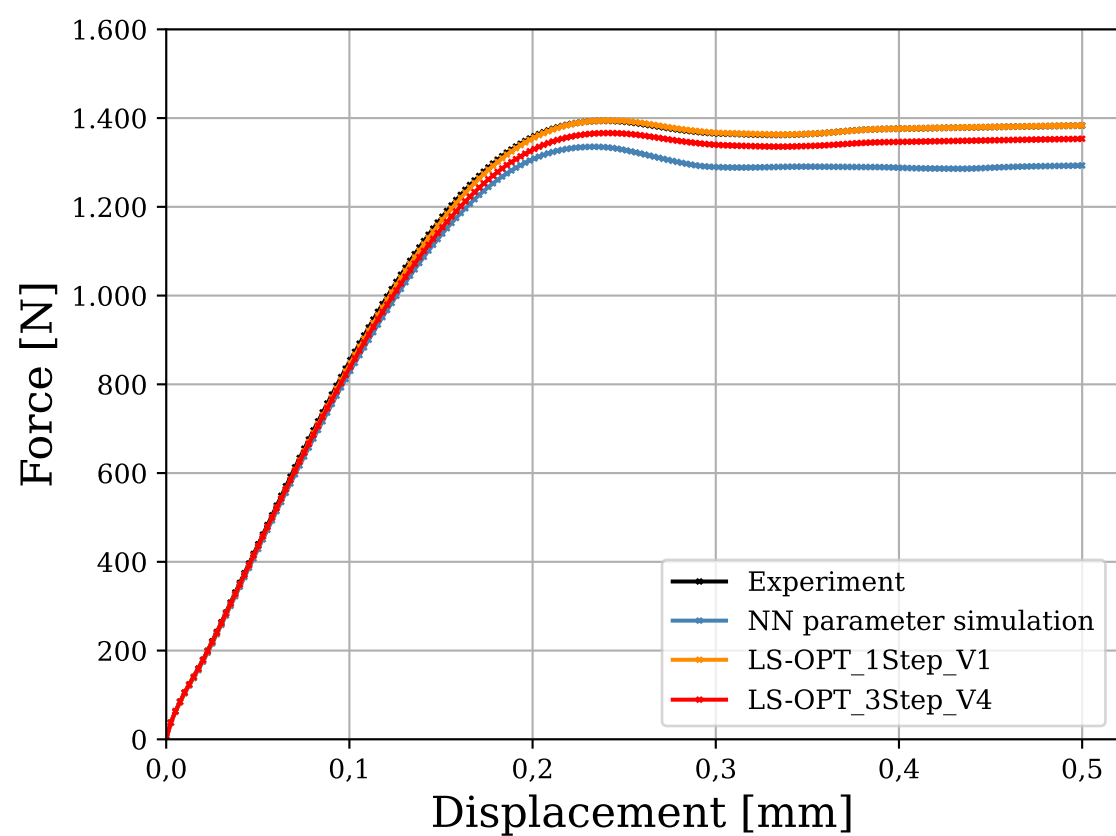

Supplement: Supplementary file 1 [file materials-15-00643-s001.zip › Supplementary_Material/SOC_NN_Pred_LSOPT_Complete/NN_Run_3/FD_Comparison_Compression_Test.pdf]

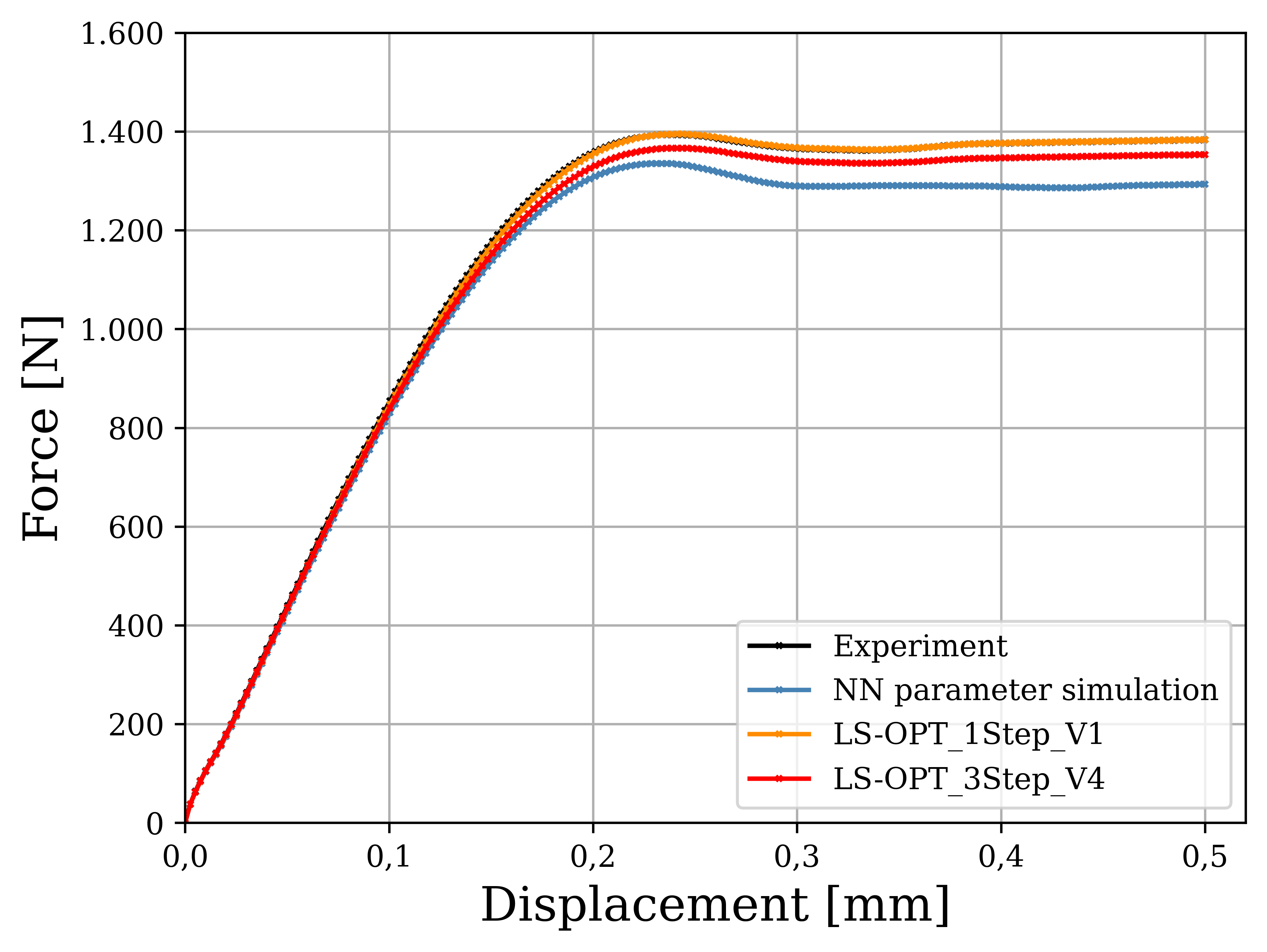

Supplement: Supplementary file 1 [file materials-15-00643-s001.zip › Supplementary_Material/SOC_NN_Pred_LSOPT_Complete/NN_Run_3/FD_Comparison_Compression_Test.png]

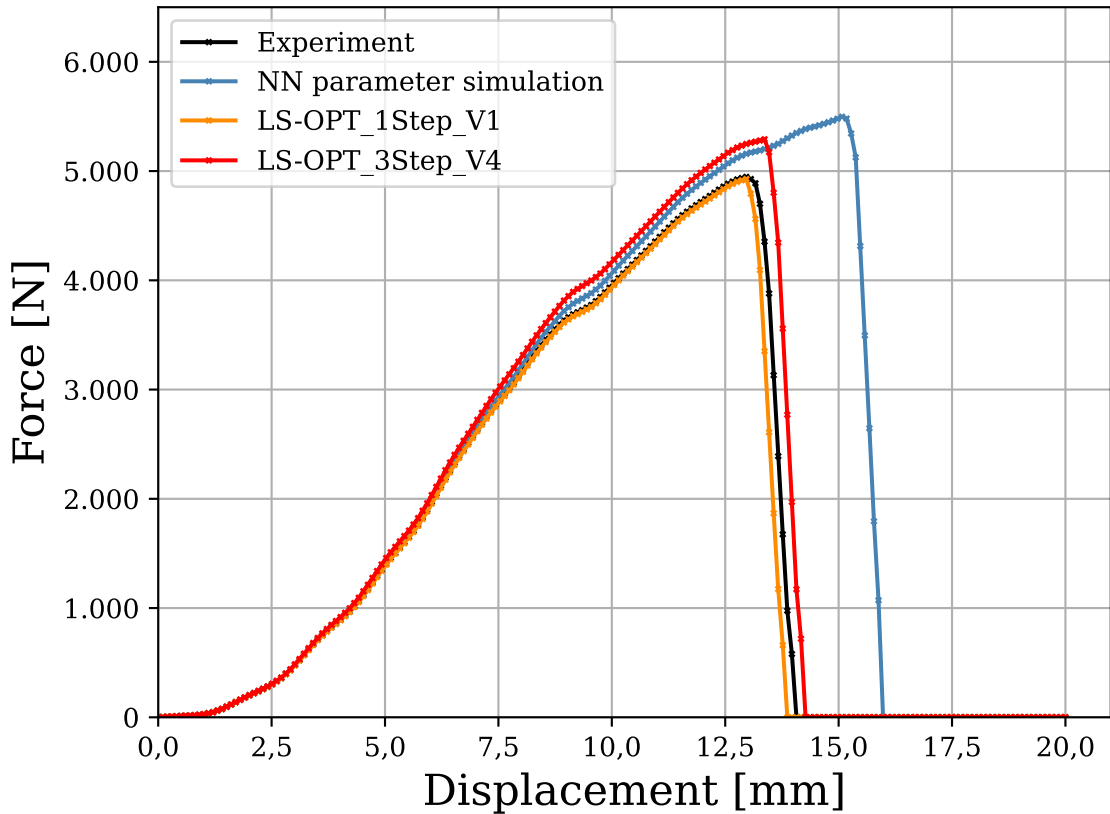

Supplement: Supplementary file 1 [file materials-15-00643-s001.zip › Supplementary_Material/SOC_NN_Pred_LSOPT_Complete/NN_Run_3/FD_Comparison_Punch_Test.pdf]

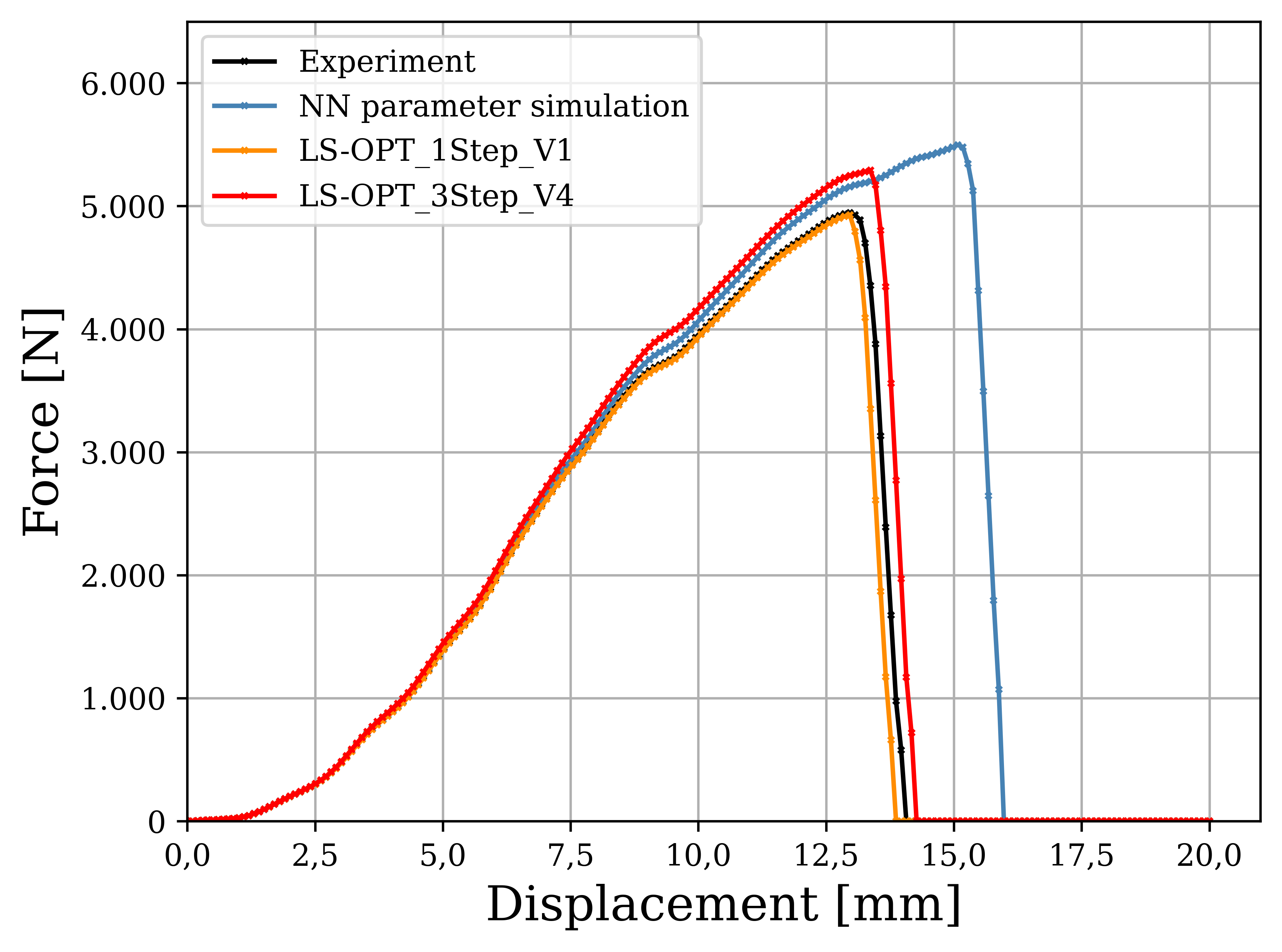

Supplement: Supplementary file 1 [file materials-15-00643-s001.zip › Supplementary_Material/SOC_NN_Pred_LSOPT_Complete/NN_Run_3/FD_Comparison_Punch_Test.png]

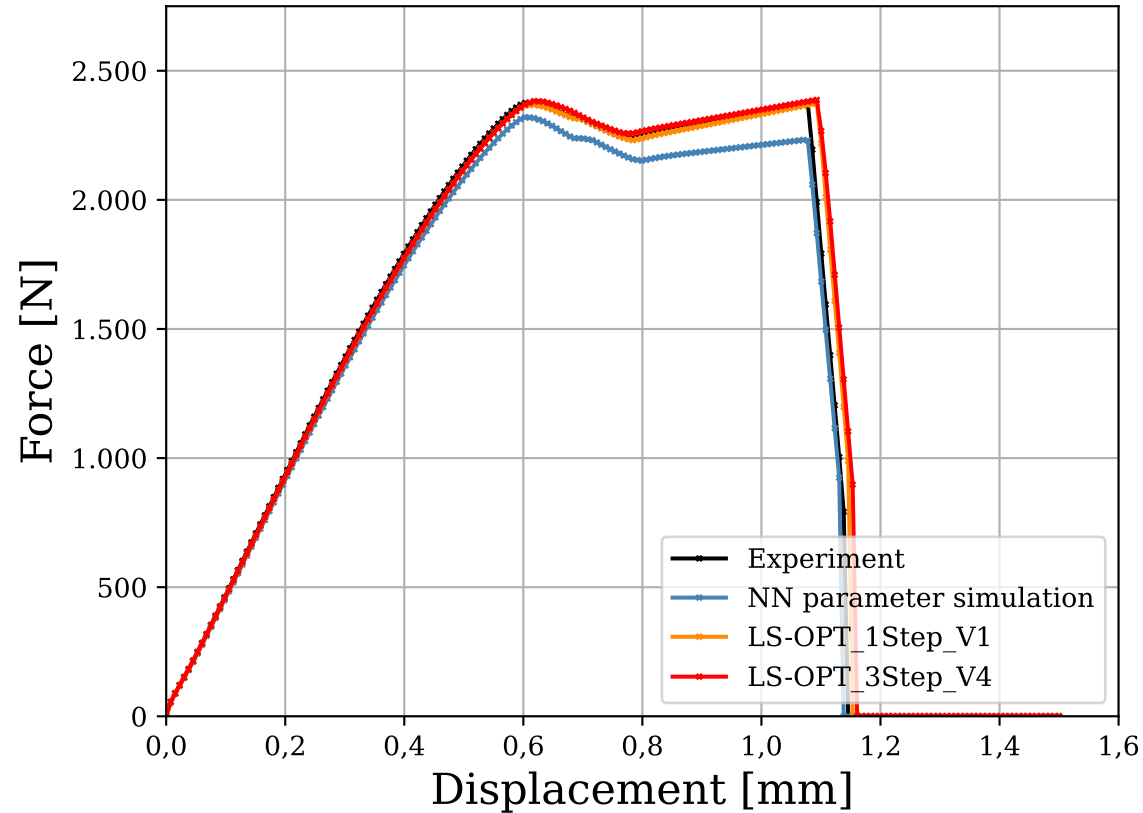

Supplement: Supplementary file 1 [file materials-15-00643-s001.zip › Supplementary_Material/SOC_NN_Pred_LSOPT_Complete/NN_Run_3/FD_Comparison_Shear_ASTM_Test.pdf]

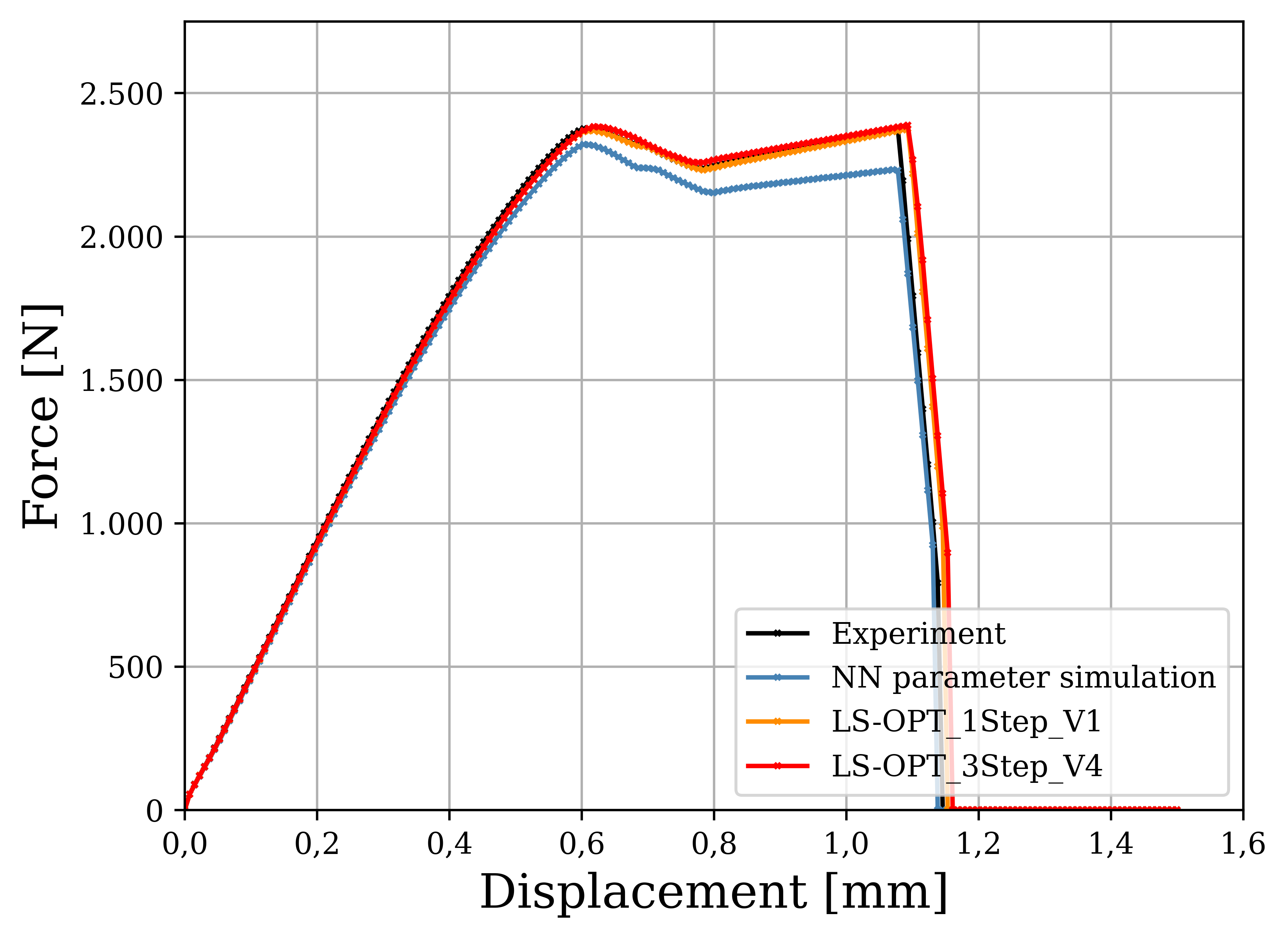

Supplement: Supplementary file 1 [file materials-15-00643-s001.zip › Supplementary_Material/SOC_NN_Pred_LSOPT_Complete/NN_Run_3/FD_Comparison_Shear_ASTM_Test.png]

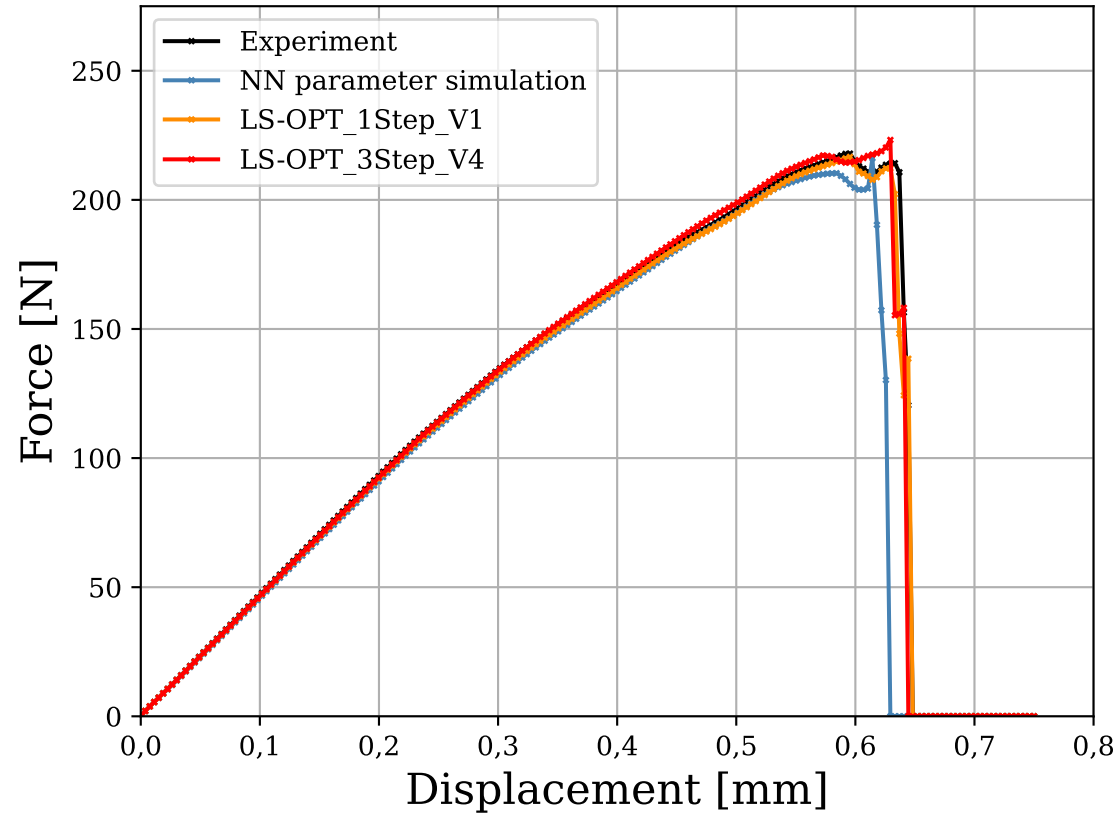

Supplement: Supplementary file 1 [file materials-15-00643-s001.zip › Supplementary_Material/SOC_NN_Pred_LSOPT_Complete/NN_Run_3/FD_Comparison_Shear_Dynamore_Test.pdf]

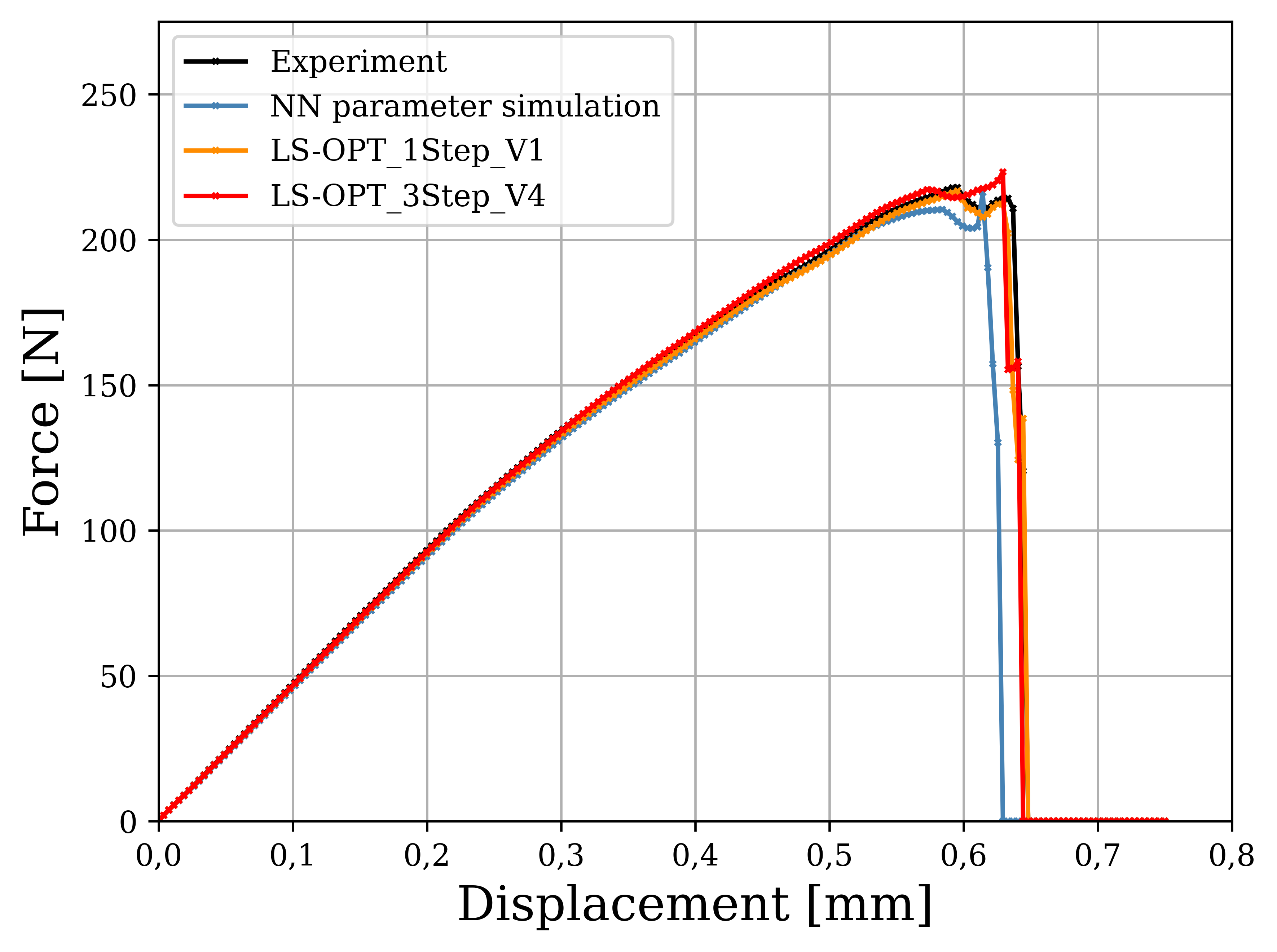

Supplement: Supplementary file 1 [file materials-15-00643-s001.zip › Supplementary_Material/SOC_NN_Pred_LSOPT_Complete/NN_Run_3/FD_Comparison_Shear_Dynamore_Test.png]

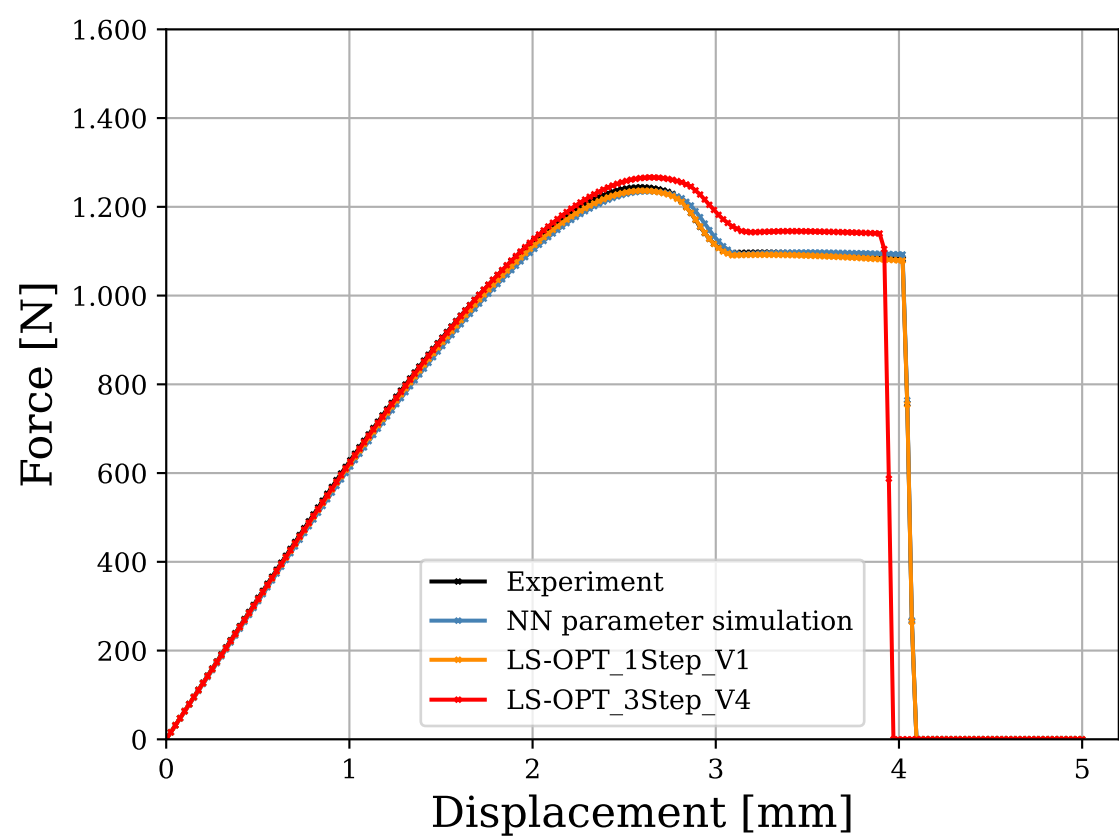

Supplement: Supplementary file 1 [file materials-15-00643-s001.zip › Supplementary_Material/SOC_NN_Pred_LSOPT_Complete/NN_Run_3/FD_Comparison_Tensile_Test.pdf]

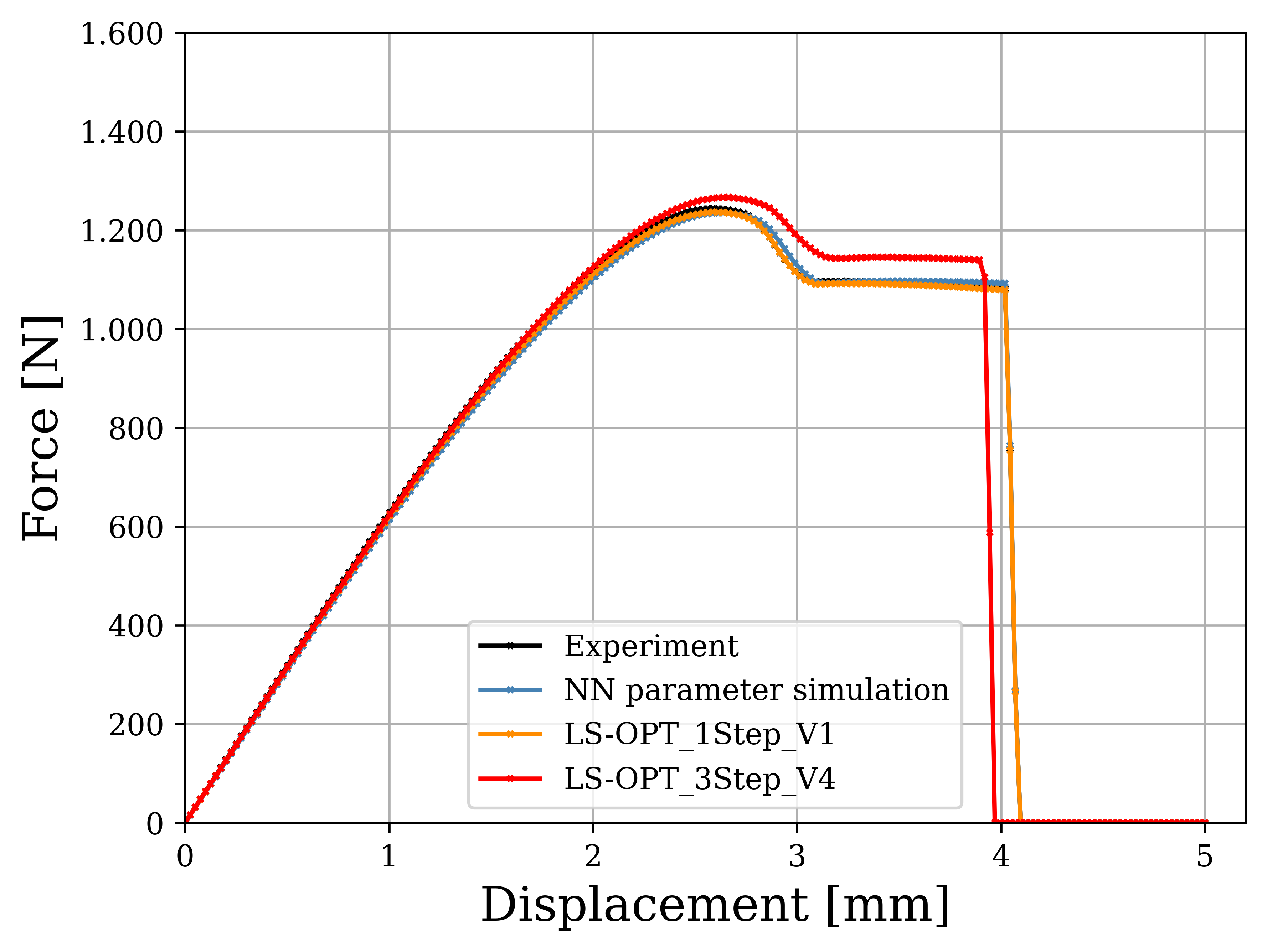

Supplement: Supplementary file 1 [file materials-15-00643-s001.zip › Supplementary_Material/SOC_NN_Pred_LSOPT_Complete/NN_Run_3/FD_Comparison_Tensile_Test.png]

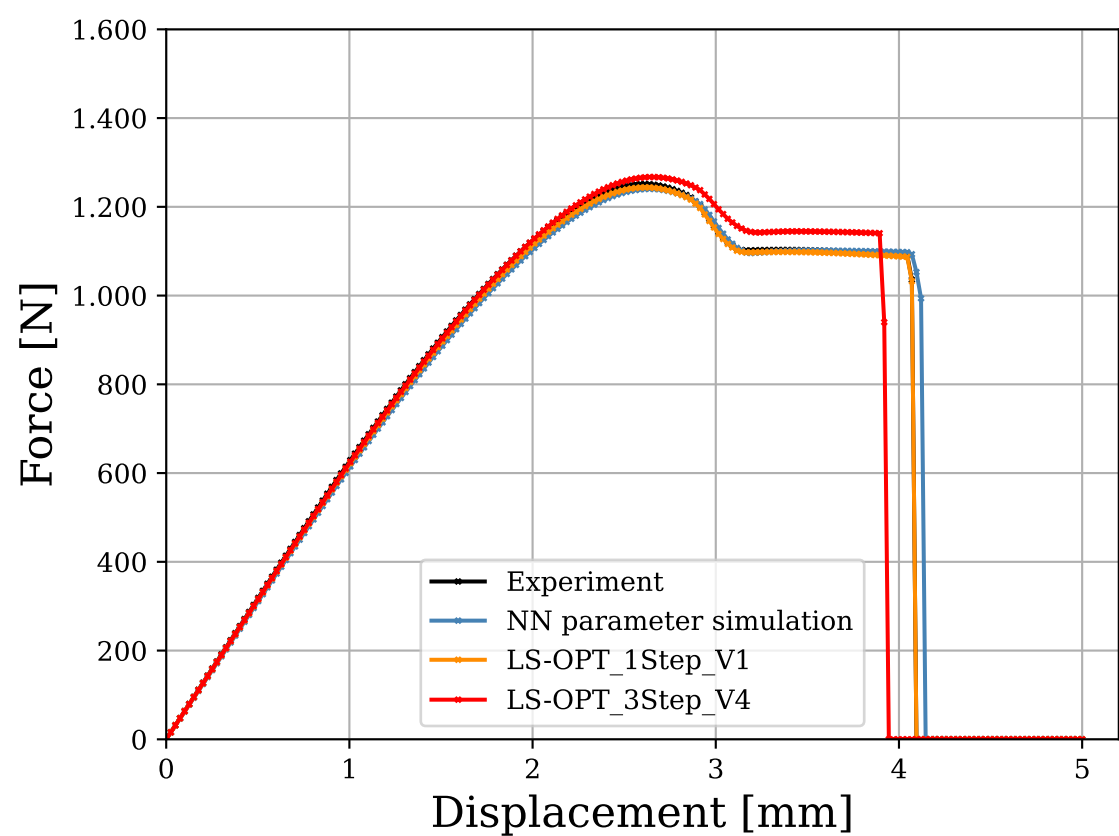

Supplement: Supplementary file 1 [file materials-15-00643-s001.zip › Supplementary_Material/SOC_NN_Pred_LSOPT_Complete/NN_Run_3/FD_Comparison_Tensile_Test_V1.pdf]

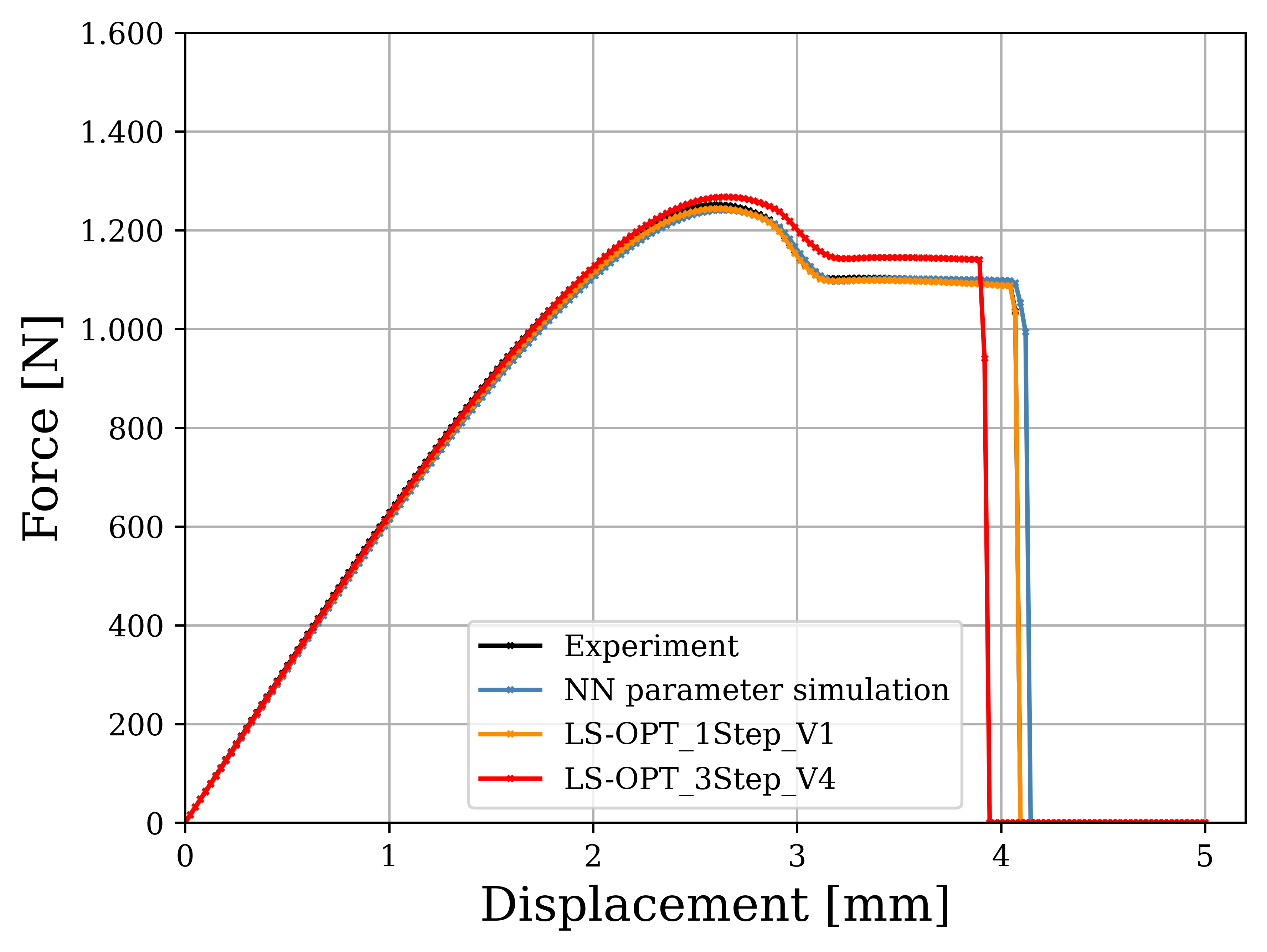

Supplement: Supplementary file 1 [file materials-15-00643-s001.zip › Supplementary_Material/SOC_NN_Pred_LSOPT_Complete/NN_Run_3/FD_Comparison_Tensile_Test_V1.png]

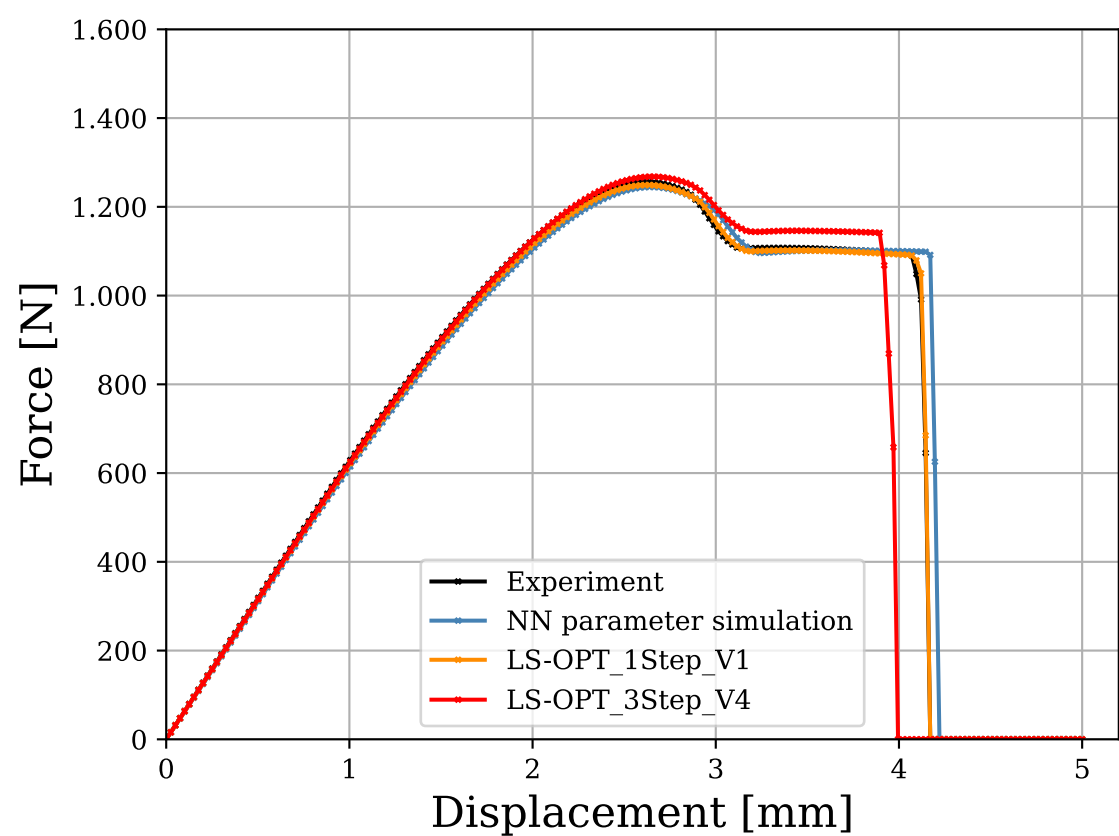

Supplement: Supplementary file 1 [file materials-15-00643-s001.zip › Supplementary_Material/SOC_NN_Pred_LSOPT_Complete/NN_Run_3/FD_Comparison_Tensile_Test_V2.pdf]

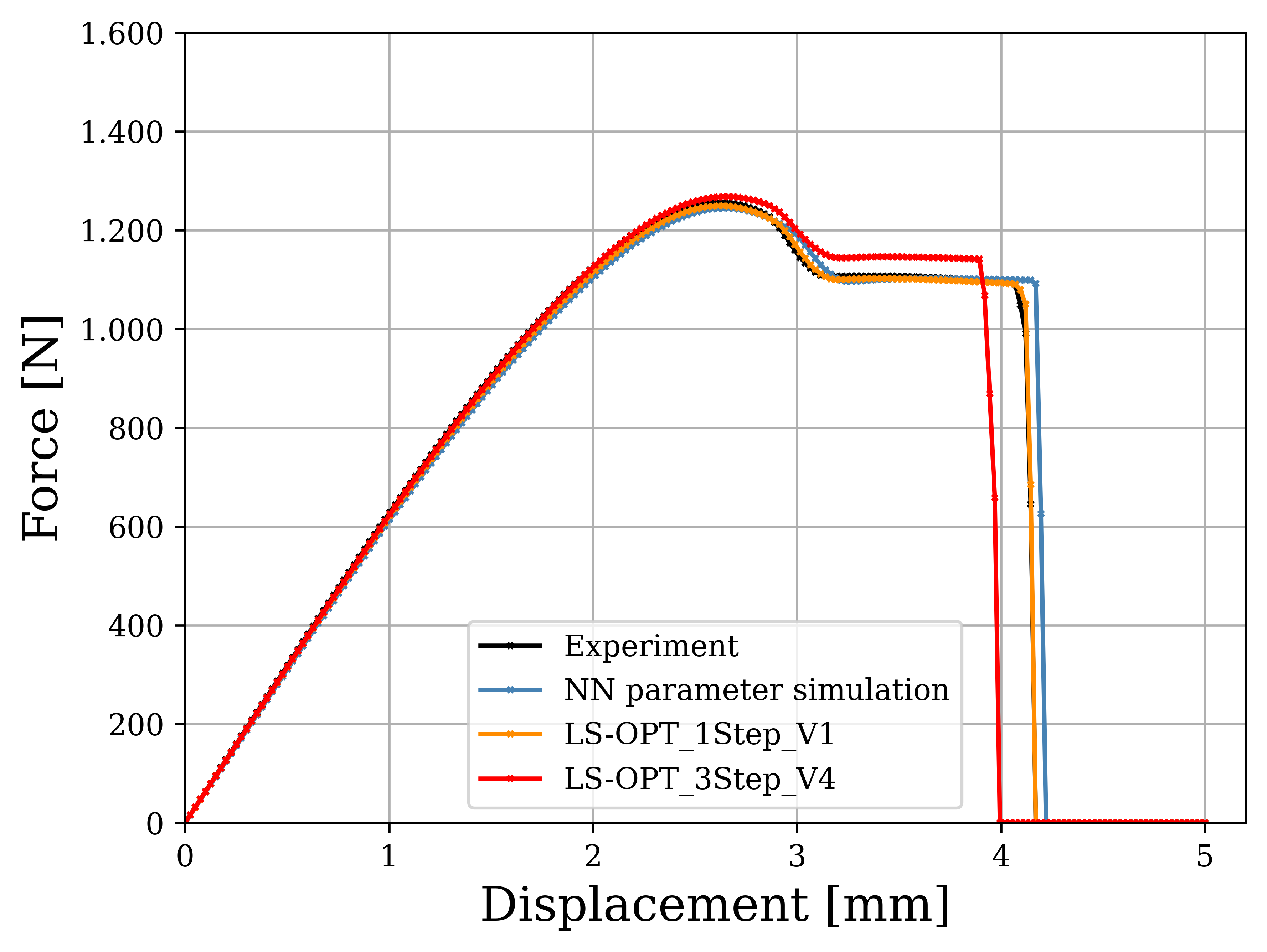

Supplement: Supplementary file 1 [file materials-15-00643-s001.zip › Supplementary_Material/SOC_NN_Pred_LSOPT_Complete/NN_Run_3/FD_Comparison_Tensile_Test_V2.png]

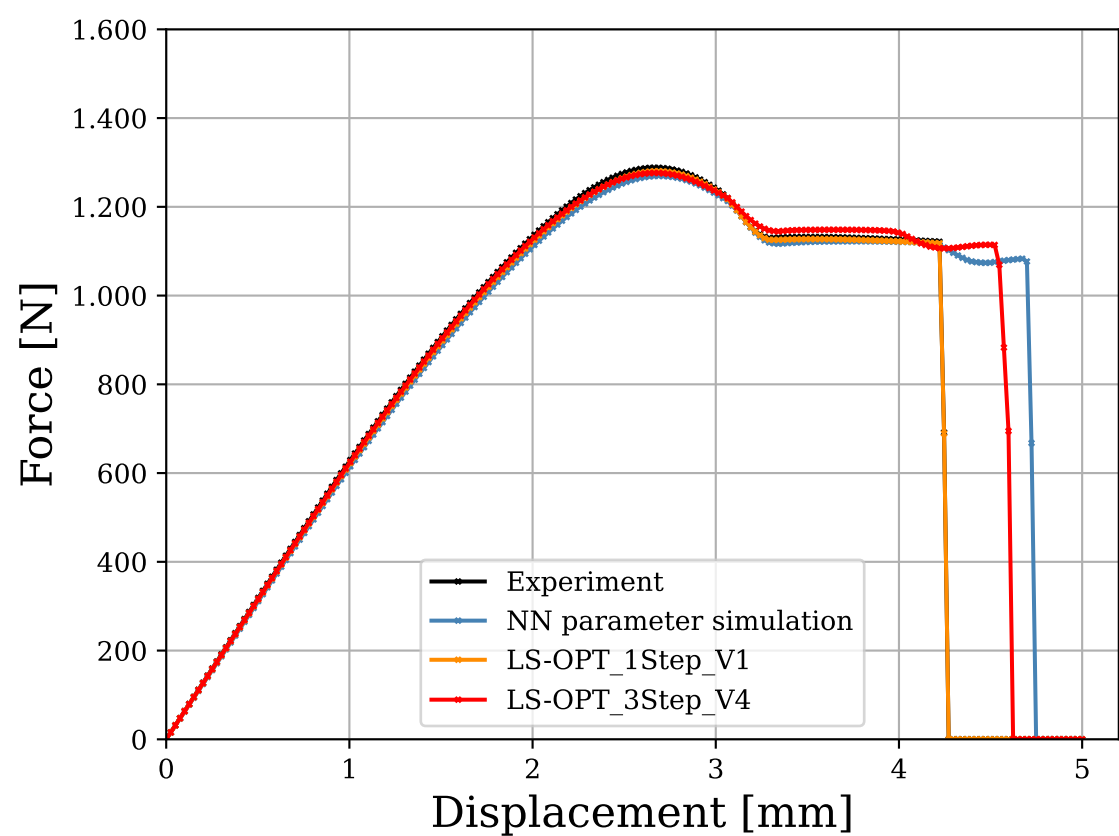

Supplement: Supplementary file 1 [file materials-15-00643-s001.zip › Supplementary_Material/SOC_NN_Pred_LSOPT_Complete/NN_Run_3/FD_Comparison_Tensile_Test_V3.pdf]

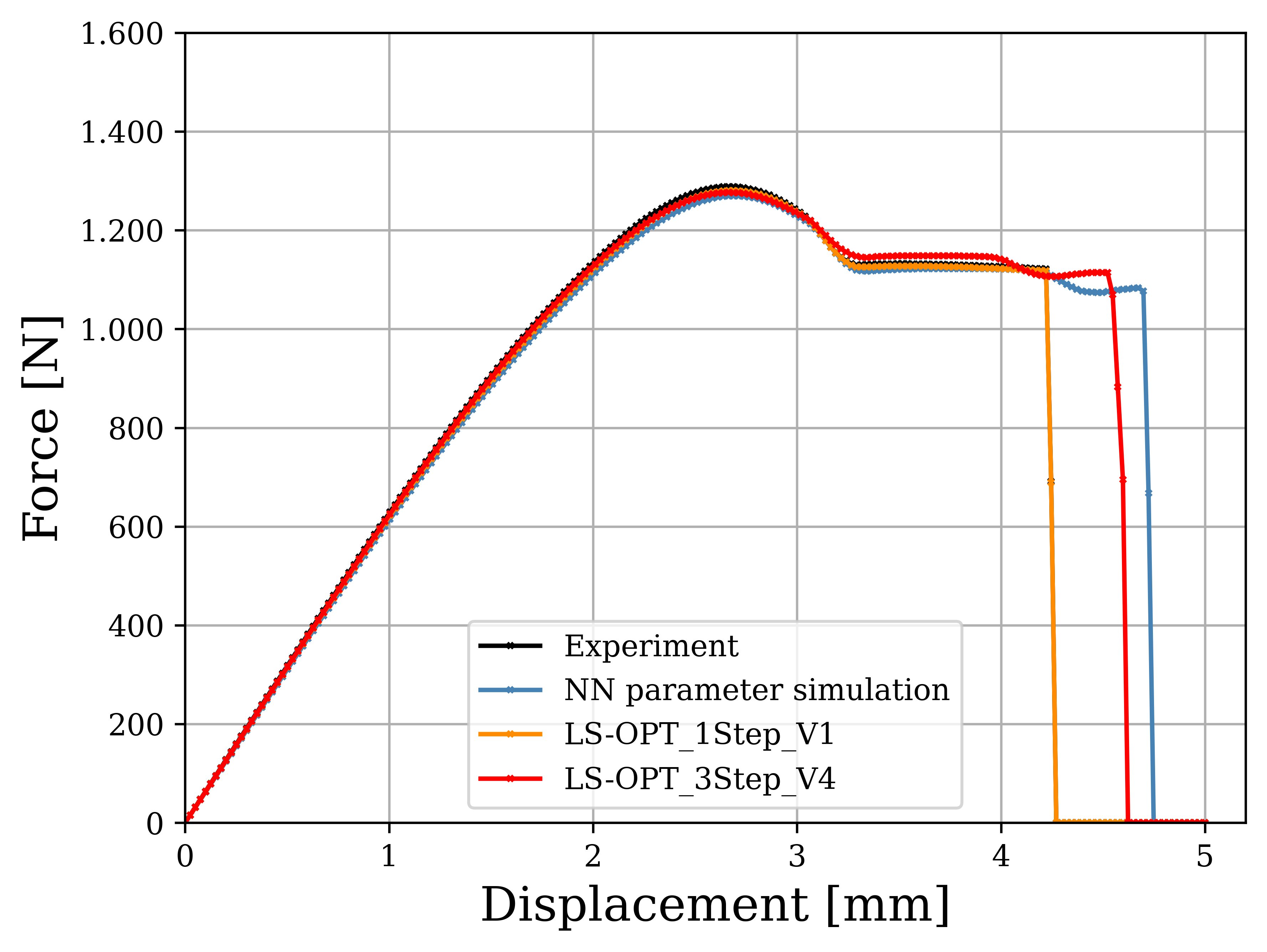

Supplement: Supplementary file 1 [file materials-15-00643-s001.zip › Supplementary_Material/SOC_NN_Pred_LSOPT_Complete/NN_Run_3/FD_Comparison_Tensile_Test_V3.png]
