# Supplementary material for: Methodology for Neural Network-Based Material Card Calibration Using LS-DYNA MAT_187_SAMP-1 Considering Failure with GISSMO
Source: Materials (Basel). 2022 Jan 15;15(2):643. doi: 10.3390/ma15020643 (PMC8778971; doi:10.3390/ma15020643)
Supplement: Supplementary file 1 [file materials-15-00643-s001.zip › Supplementary_Material/SOC_NN_Pred_LSOPT_Complete/NN_Run_8/PE_Comparison_Compression_Test.pdf]

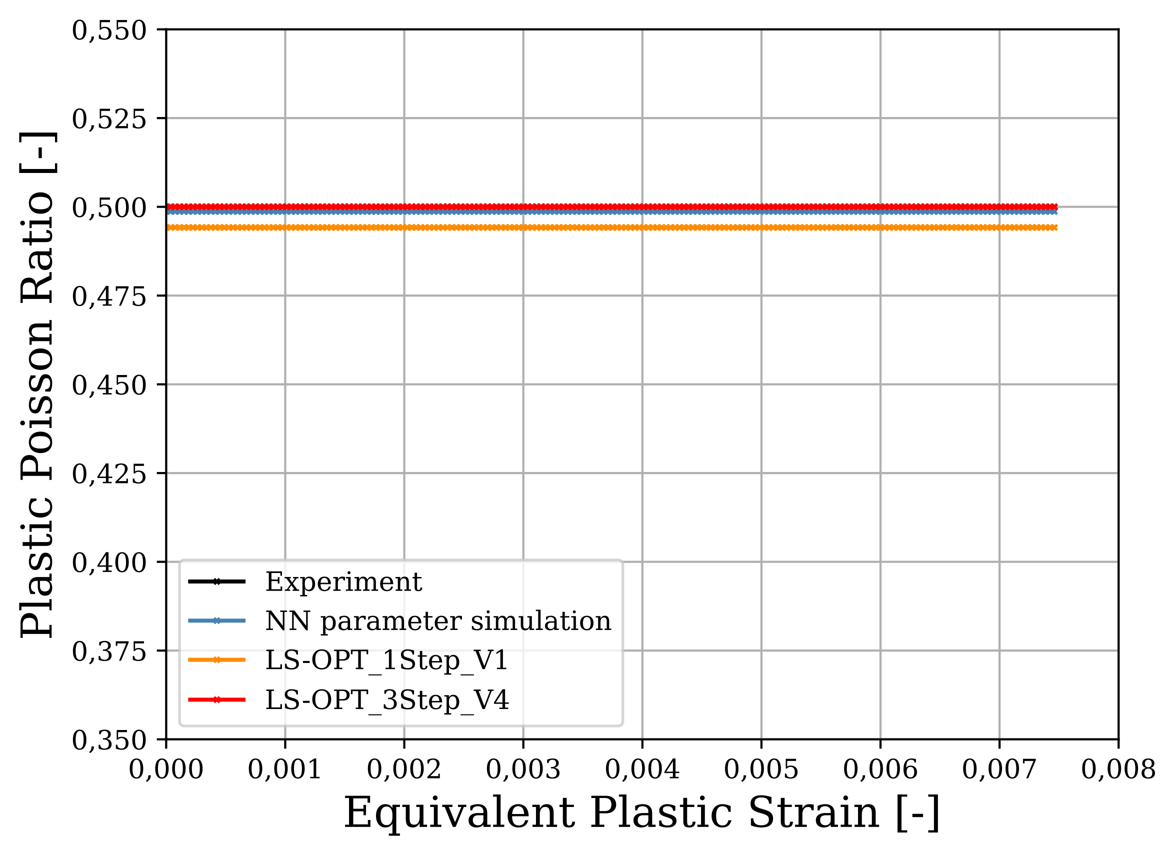

Plastic Poisson Ratio [-]
0,550
0,525
0,500
0,475
0,450
0,425
0,400
0,375
0,350
0,000
0,001
0,002
0,003
0,004
0,005
0,006
0,007
0,008
Equivalent Plastic Strain [-]
Experiment
NN parameter simulation
LS-OPT_1Step_V1
LS-OPT_3Step_V4
