# Supplementary material for: Methodology for Neural Network-Based Material Card Calibration Using LS-DYNA MAT_187_SAMP-1 Considering Failure with GISSMO
Source: Materials (Basel). 2022 Jan 15;15(2):643. doi: 10.3390/ma15020643 (PMC8778971; doi:10.3390/ma15020643)
Supplement: Supplementary file 1 [file materials-15-00643-s001.zip › Supplementary_Material/SOC_NN_Pred_LSOPT_Complete/NN_Run_9/FD_Comparison_Shear_ASTM_Test.pdf]

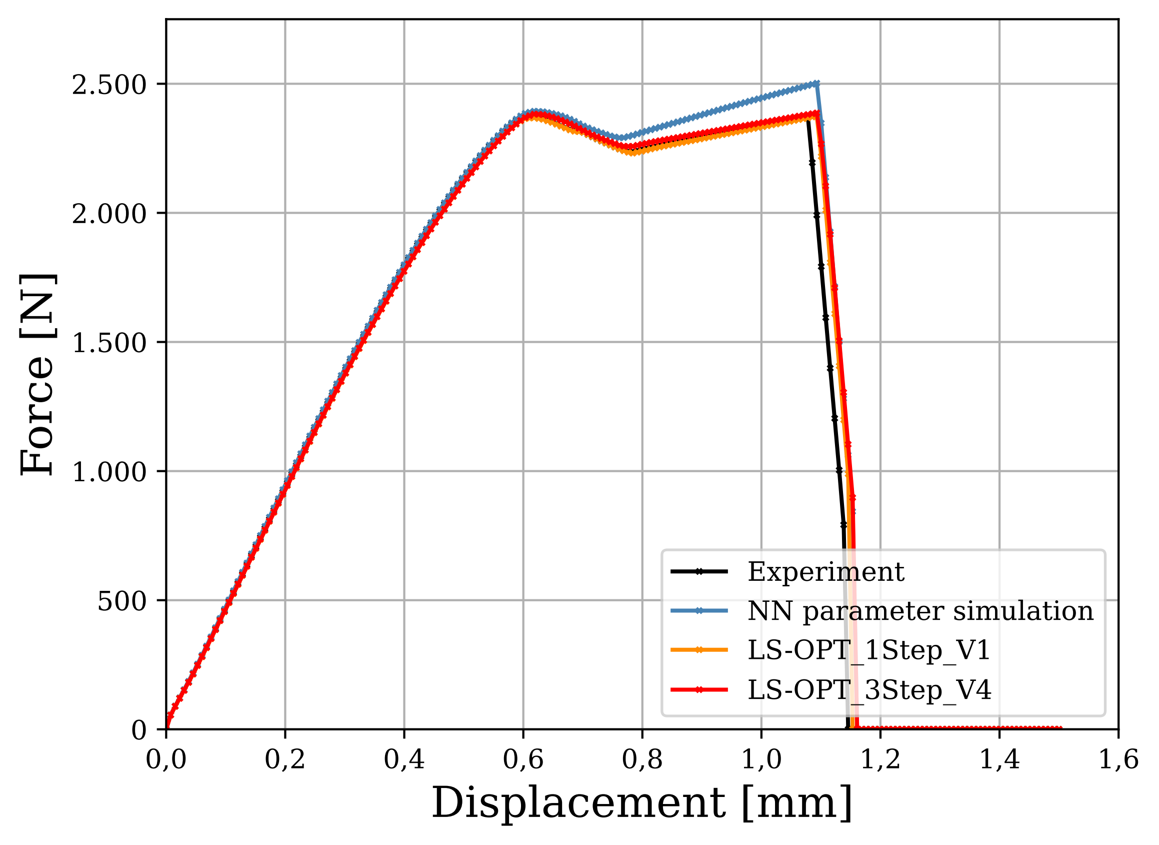
Force [N]
2.500
2.000
1.500
1.000
500
0
0,0
0,2
0,4
0,6
0,8
1,0
1,2
1,4
1,6
Displacement [mm]
Experiment
NN parameter simulation
LS-OPT_1Step_V1
LS-OPT_3Step_V4
