# Supplementary material for: Methodology for Neural Network-Based Material Card Calibration Using LS-DYNA MAT_187_SAMP-1 Considering Failure with GISSMO
Source: Materials (Basel). 2022 Jan 15;15(2):643. doi: 10.3390/ma15020643 (PMC8778971; doi:10.3390/ma15020643)
Supplement: Supplementary file 1 [file materials-15-00643-s001.zip › Supplementary_Material/SOC_NN_Pred_LSOPT_Complete/NN_Run_9/FD_Comparison_Shear_Dynamore_Test.pdf]

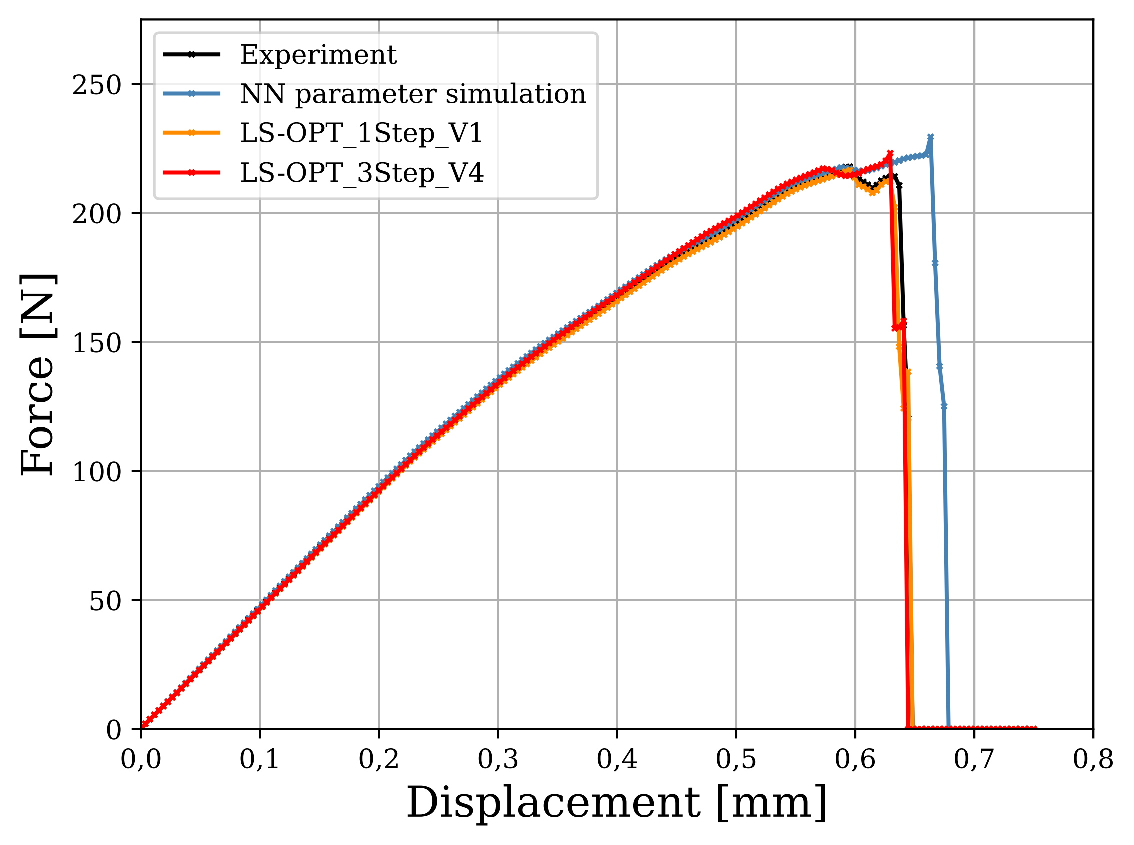
Experiment
NN parameter simulation
LS-OPT_1Step_V1
LS-OPT_3Step_V4
Force [N]
Displacement [mm]
0
50
100
150
200
250
0,0
0,1
0,2
0,3
0,4
0,5
0,6
0,7
0,8
