# Supplementary material for: Methodology for Neural Network-Based Material Card Calibration Using LS-DYNA MAT_187_SAMP-1 Considering Failure with GISSMO
Source: Materials (Basel). 2022 Jan 15;15(2):643. doi: 10.3390/ma15020643 (PMC8778971; doi:10.3390/ma15020643)
Supplement: Supplementary file 1 [file materials-15-00643-s001.zip › Supplementary_Material/SOC_NN_Pred_LSOPT_Complete/NN_Run_9/FD_Comparison_Tensile_Test_V3.pdf]

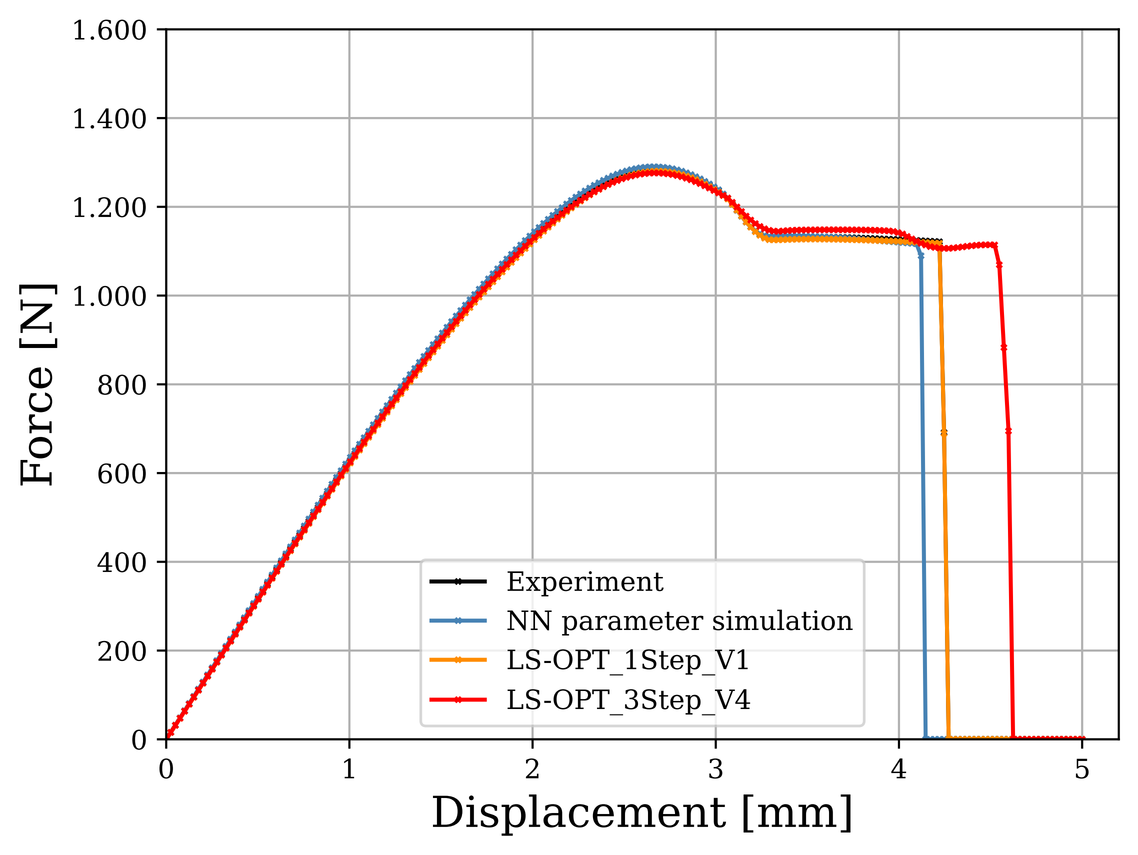

1.600
1.400
1.200
1.000
800
600
400
200
0
Force [N]
0
1
2
3
4
5
Displacement [mm]
Experiment
NN parameter simulation
LS-OPT_1Step_V1
LS-OPT_3Step_V4
