# Supplementary material for: Methodology for Neural Network-Based Material Card Calibration Using LS-DYNA MAT_187_SAMP-1 Considering Failure with GISSMO
Source: Materials (Basel). 2022 Jan 15;15(2):643. doi: 10.3390/ma15020643 (PMC8778971; doi:10.3390/ma15020643)
Supplement: Supplementary file 1 [file materials-15-00643-s001.zip › Supplementary_Material/SOC_Range_Sets/1/FD_Comparison_Punch_Range.pdf]

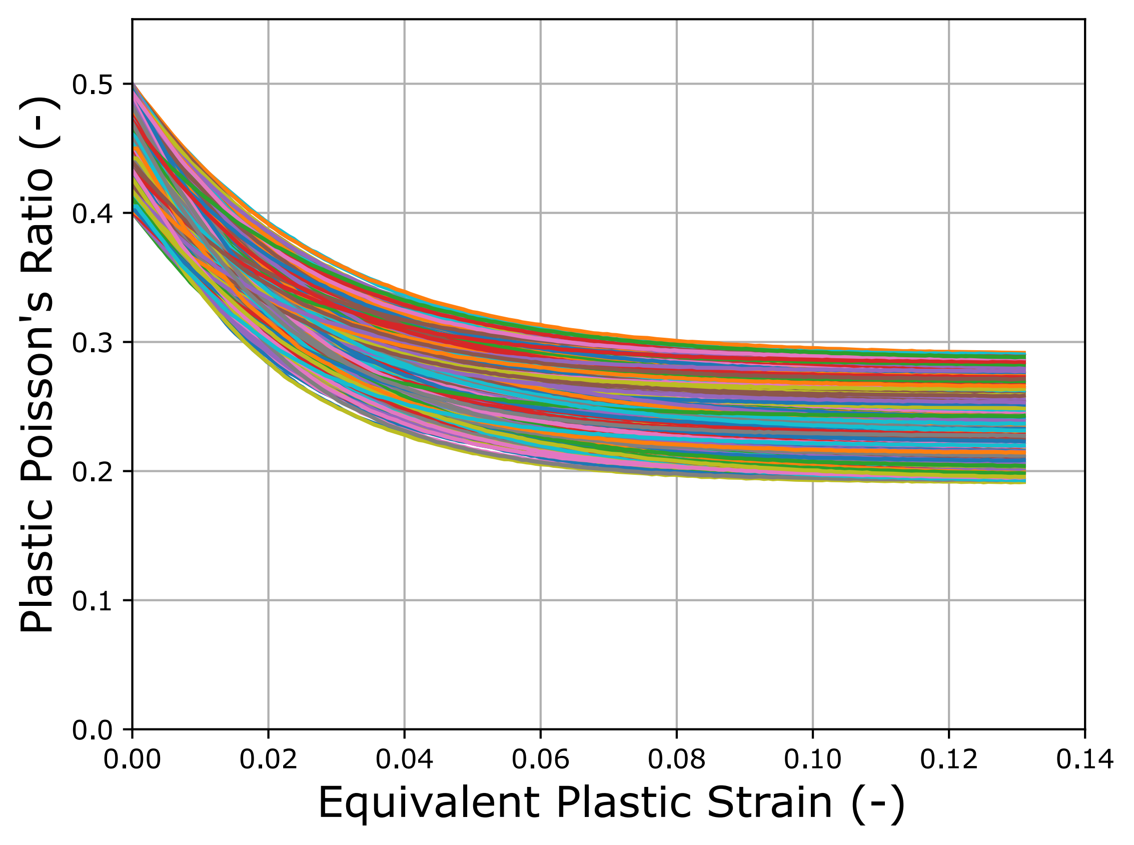

Plastic Poisson's Ratio (-)
0.0
0.1
0.2
0.3
0.4
0.5
0.00
0.02
0.04
0.06
0.08
0.10
0.12
0.14
Equivalent Plastic Strain (-)
