# Supplementary material for: Methodology for Neural Network-Based Material Card Calibration Using LS-DYNA MAT_187_SAMP-1 Considering Failure with GISSMO
Source: Materials (Basel). 2022 Jan 15;15(2):643. doi: 10.3390/ma15020643 (PMC8778971; doi:10.3390/ma15020643)
Supplement: Supplementary file 1 [file materials-15-00643-s001.zip › Supplementary_Material/SOC_Range_Sets/1/FD_Comparison_Shear_astm_Range.pdf]

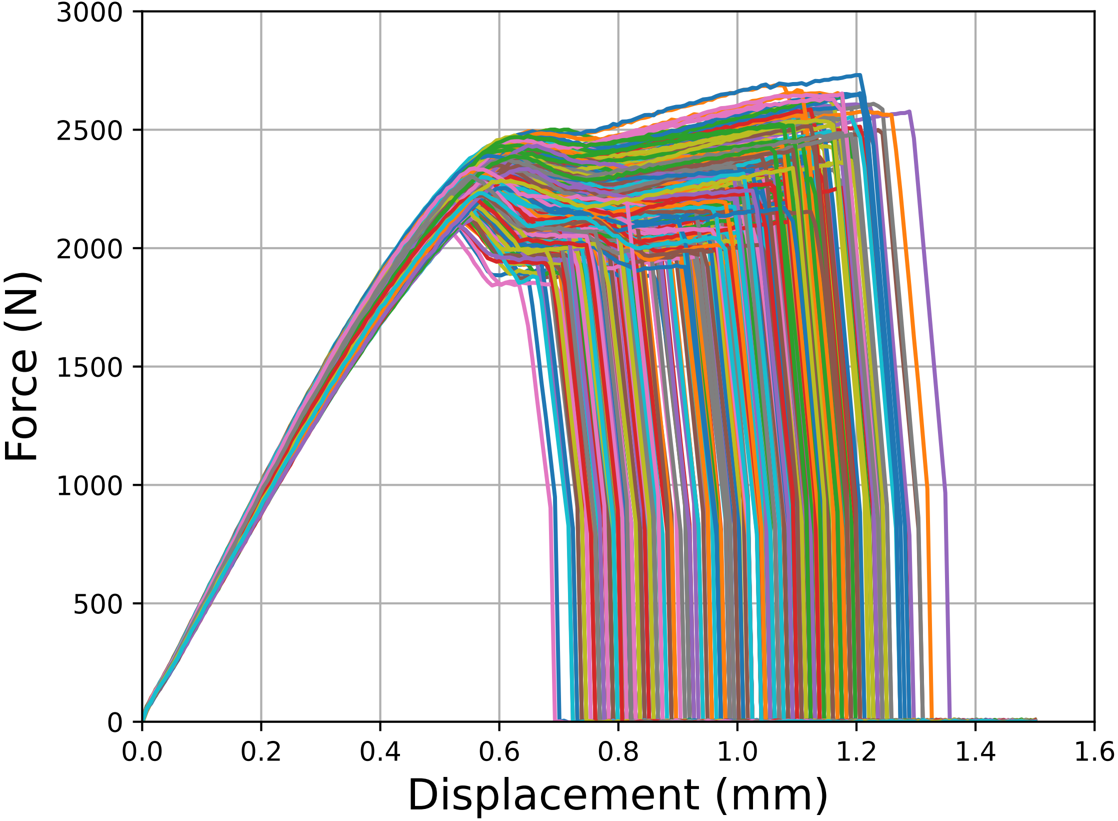

Force (N)
3000
2500
2000
1500
1000
500
0
0.0
0.2
0.4
0.6
0.8
1.0
1.2
1.4
1.6
Displacement (mm)
