# Supplementary material for: Methodology for Neural Network-Based Material Card Calibration Using LS-DYNA MAT_187_SAMP-1 Considering Failure with GISSMO
Source: Materials (Basel). 2022 Jan 15;15(2):643. doi: 10.3390/ma15020643 (PMC8778971; doi:10.3390/ma15020643)
Supplement: Supplementary file 1 [file materials-15-00643-s001.zip › Supplementary_Material/SOC_Range_Sets/2/FD_Comparison_PPR_Compression_Range.pdf]

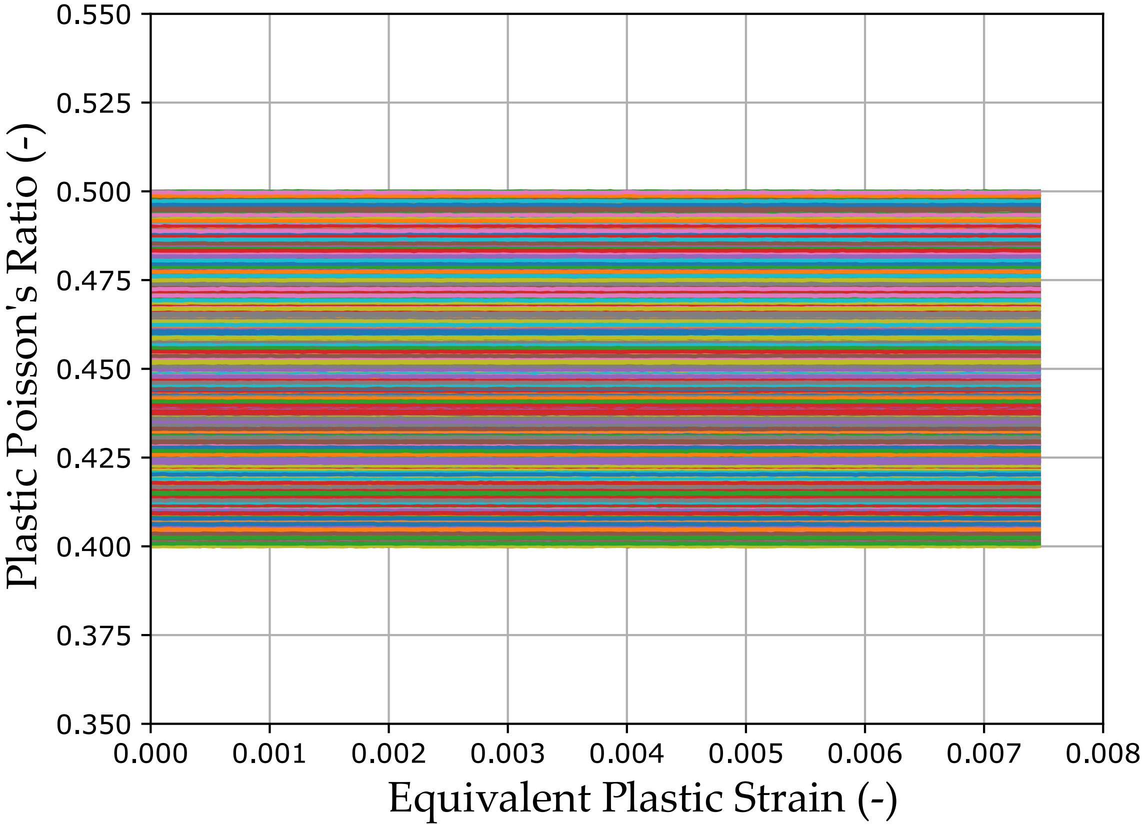

Plastic Poisson's Ratio (-)
0.550
0.525
0.500
0.475
0.450
0.425
0.400
0.375
0.350
0.000
0.001
0.002
0.003
0.004
0.005
0.006
0.007
0.008
Equivalent Plastic Strain (-)
