# Supplementary material for: Methodology for Neural Network-Based Material Card Calibration Using LS-DYNA MAT_187_SAMP-1 Considering Failure with GISSMO
Source: Materials (Basel). 2022 Jan 15;15(2):643. doi: 10.3390/ma15020643 (PMC8778971; doi:10.3390/ma15020643)
Supplement: Supplementary file 1 [file materials-15-00643-s001.zip › Supplementary_Material/SOC_Range_Sets/2/FD_Comparison_Punch_Range.pdf]

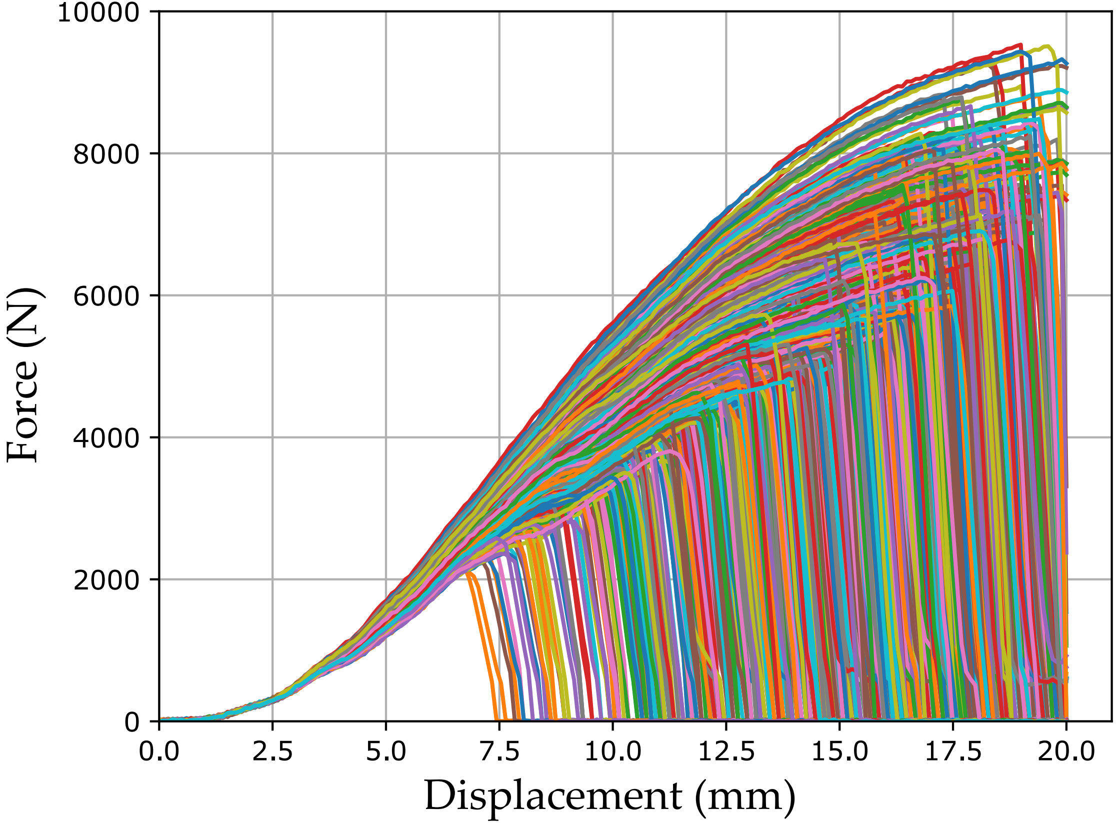

Force (N)
10000
8000
6000
4000
2000
0
0.0
2.5
5.0
7.5
10.0
12.5
15.0
17.5
20.0
Displacement (mm)
