# Supplementary material for: Methodology for Neural Network-Based Material Card Calibration Using LS-DYNA MAT_187_SAMP-1 Considering Failure with GISSMO
Source: Materials (Basel). 2022 Jan 15;15(2):643. doi: 10.3390/ma15020643 (PMC8778971; doi:10.3390/ma15020643)
Supplement: Supplementary file 1 [file materials-15-00643-s001.zip › Supplementary_Material/SOC_Range_Sets/2/FD_Comparison_Tensile_V1_Range.pdf]

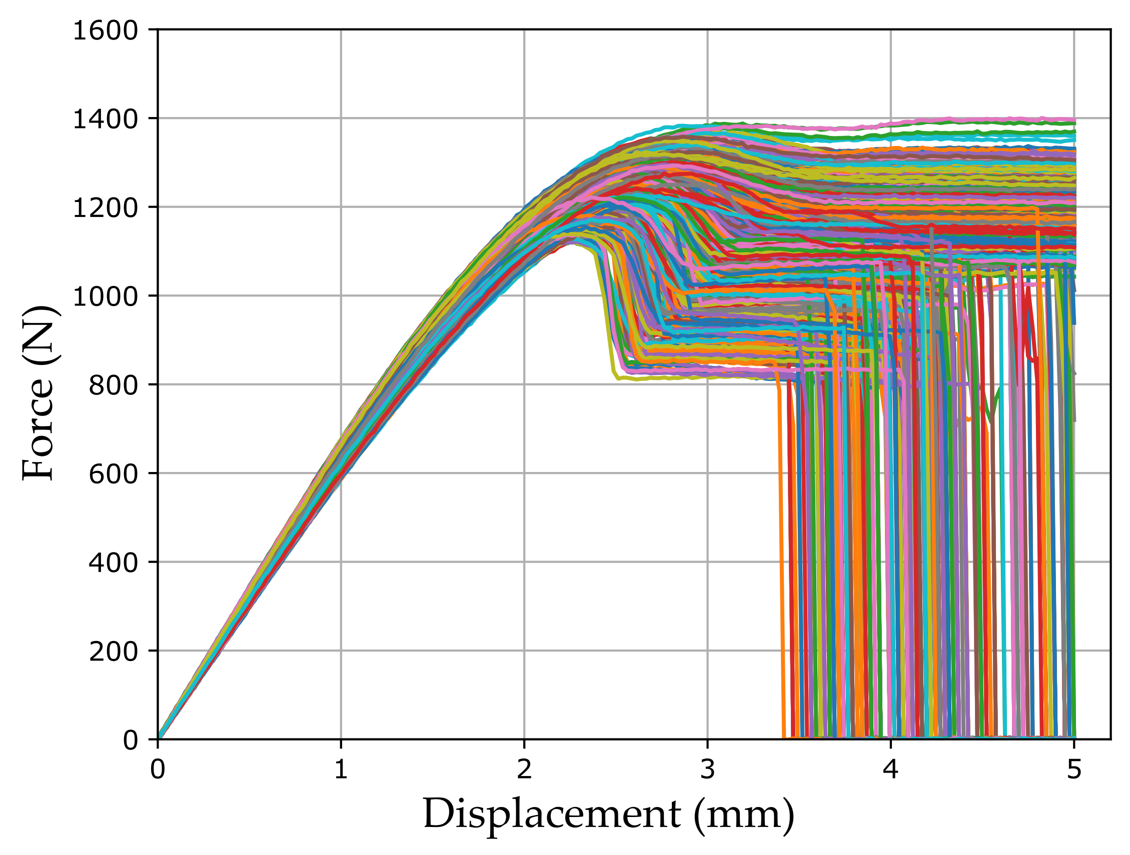

1600
1400
1200
1000
800
600
400
200
0
Force (N)
0
1
2
3
4
5
Displacement (mm)
