# Supplementary material for: Methodology for Neural Network-Based Material Card Calibration Using LS-DYNA MAT_187_SAMP-1 Considering Failure with GISSMO
Source: Materials (Basel). 2022 Jan 15;15(2):643. doi: 10.3390/ma15020643 (PMC8778971; doi:10.3390/ma15020643)
Supplement: Supplementary file 1 [file materials-15-00643-s001.zip › Supplementary_Material/SOC_Range_Sets/3/FD_Comparison_Bending_Range.pdf]

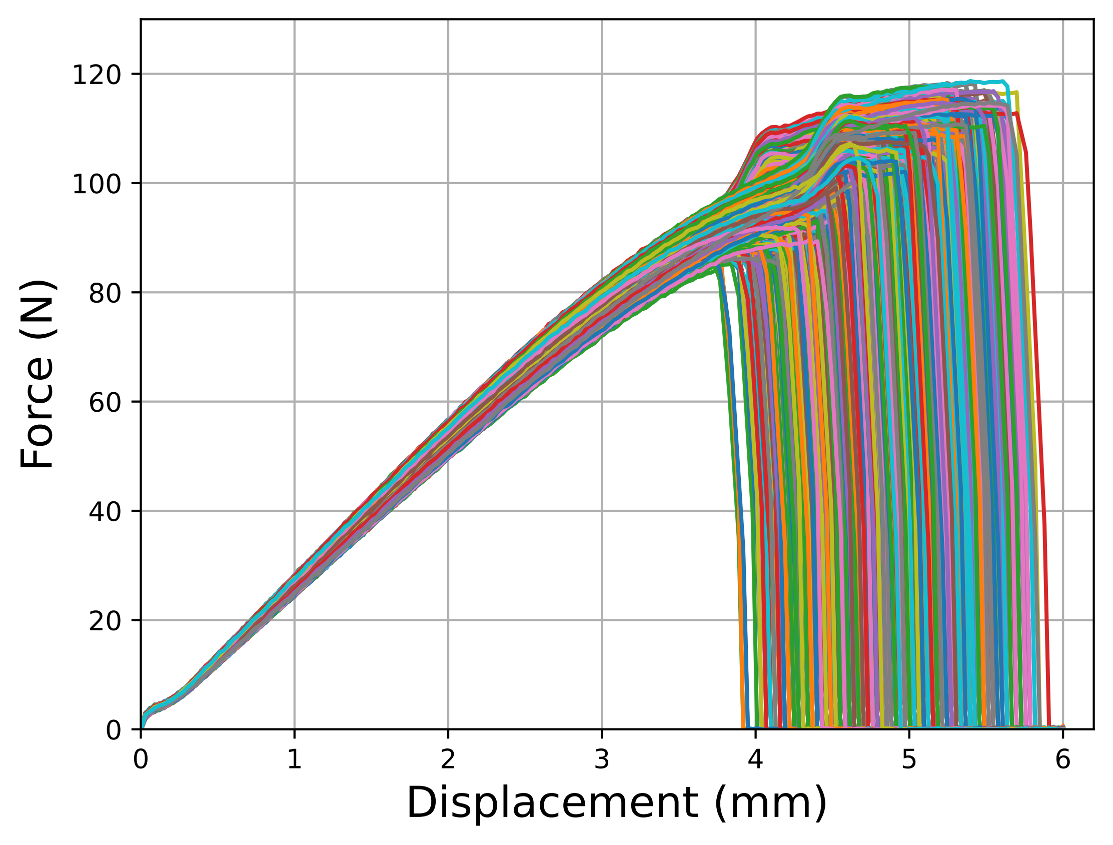

Force (N)
Displacement (mm)
0
20
40
60
80
100
120
0
1
2
3
4
5
6
